# A practical method to control spatiotemporal confounding in environmental impact studies

**DOI:** 10.1016/j.mex.2018.07.003

**Published:** 2018-07-05

**Authors:** Rezvan Hatami

**Affiliations:** Department of Ecology, Environment and Evolution, La Trobe University, Bundoora, 3086, Victoria, Australia

**Keywords:** Causal modelling technique, Spatiotemporal confounding, Natural variation, Structural equation modelling (SEM), Bayesian Networks (BNs)

## Abstract

Separating natural spatiotemporal variation from the impact of human activities has long been a challenge in environmental impact studies. To overcome this problem, a causal modelling method based on spatiotemporal data, integrated with existing statistical methods such as multivariate redundancy analysis, multiple regression and, ordination was used for inferring causal effects of wastewater on biotic ecosystems. The causal modelling techniques were structural equation modelling (SEM) and Bayesian Networks (BNs); SEM, with the help of statistical analysis, was used for building deterministic models while the composite hypothesis underlying the models was checked based on the principle of BNs. Both spatial and temporal variations were considered in the design of the study so that spatiotemporal confounding could be controlled by adjusting for ‘time’ and ‘distance’ in the models. This improved the external validity of the models, so they could be used for predicting the effect of interventions, e.g. manipulating the discharge loads. This could be possible where time-varying variables such as quantity of discharge effluent were included in the models. Models can be used for prediction the effect of an intervention in situations understood as causal. Thus, the causal structure of composite hypotheses of the study was tested using both local and global tests.

## Graphical abstract

**Figure:**
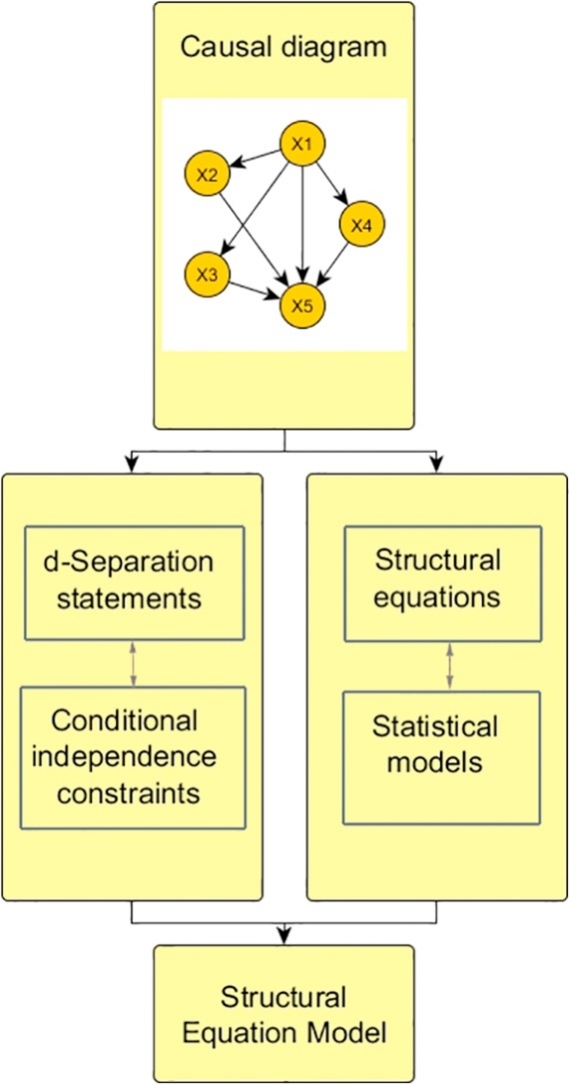

Specifications Table

**Table:** 

Subject area	*Environmental Science*
More specific subject area	*Environmental impact studies*
Method name	*Causal modelling technique*
Name and reference of original method	[18,19,22]
Resource availability	*links to data and R codes*

## Method details

The statistical analysis for causal modelling follows the approach used by Pearl [22] and Paul and Anderson [19]. All of the analysis was done using R version 3.2.2 [23]. The main packages used for data analysis and building graphs included ‘vegan’ [15], ‘Car’ [3], ‘lattice’ [24], ‘ecodist’ [6], ‘BiodiversityR’ [10], ‘caret’ [11], and ‘stats’ (lines 6–20 in R). The application of the methodology introduced in this paper was tested in a study conducted by Hatami [8].The list of all packages used in this study and all of the codes written in R can be found in Supplement 1.

### Building a causal diagram

**Fig. 1:**
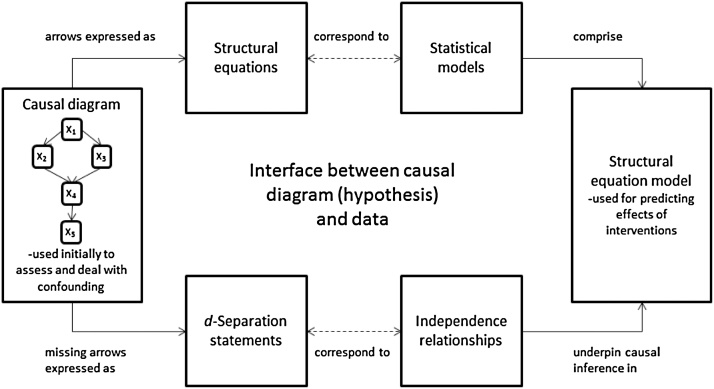
The process of graph theoretical structural equation modelling [17].

Graph theoretical structural equation modelling (SEM) was combined with the principles of Bayesian Networks (BNs) to control for natural spatiotemporal confounding while checking for causal relationships between abiotic and biotic variables. Graph theoretical SEM is used to translate a causal diagram into structural equation models, and to test causal models. This process is summarised in Fig. 1 from Paul, Rokahr [17]. As the linchpin of the causal process is a causal diagram, the first step was to build a causal diagram. Causal diagrams are a form of Directed Acyclic Graphs (DAGs) that present causal assumptions visually, and consist of nodes or vertices, arrows and missing arrows [2,25]. Arrows in a causal diagram represent causal relationships between variables [25], and missing arrows show the assumption of no direct causal relationship between two variables [2]. By including ‘time’ and ‘distance’ in the causal diagram, it depicts the variation in biotic communities along the stream (distance) over time due to natural and anthropogenic activities. Natural variability is related to changes that are imposed to the river by natural events including floods, substratum and habitat disruption, and seasonal changes in light and temperature. The impact of human activities includes the introduction of pollutants such as nutrients and sediments into the river; for example, discharges from treatment plants, industrial plants, mining activities, and fish farms [1]. After building a causal diagram, the next step was to use SEM for building a list of structural equations entailed in the arrows, and to utilise the principle of BNs for writing a set of *d*-separation statements represented in missing arrows. *d*-Separation statements are graphically equivalent to conditional independence relationships, and structural equations are used as a guide for building statistical models [22,25].

### Exploratory analyses

A series of exploratory analyses was first undertaken for visual inspection of systematic patterns in the data. That is, any trend or pattern in the data was explored by examining the graphs representing the data variation across time or with distance. All the measured environmental variables were plotted against distance (lines 35–271 in R code, Supplementary Online Material). A community table can be constructed using the *vegemite()* function to display variation of macroinvertebrate abundances along sites (line 275 in R). To continue with exploratory data analysis, the main Principal Co-ordinate (PCO) axes need to be derived from macroinvertebrate abundance data. For doing so, the *Vegdist()* function was used to produce a Bray-Curtis dissimilarity matrix of square-root transformed abundance data. This was followed by using function *cmdscale()* to perform a principal co-ordinate analysis (PCoA) in order to convert the samples-by-species data matrix into a smaller matrix of PCO scores, so that the PCO scores represent a measure of the state of the macroinvertebrate community at a point in time and space (lines 277–290 in R). After extracting the PCO axes, it was important to reveal the nontrivial PCOs.

**Fig. 2:**
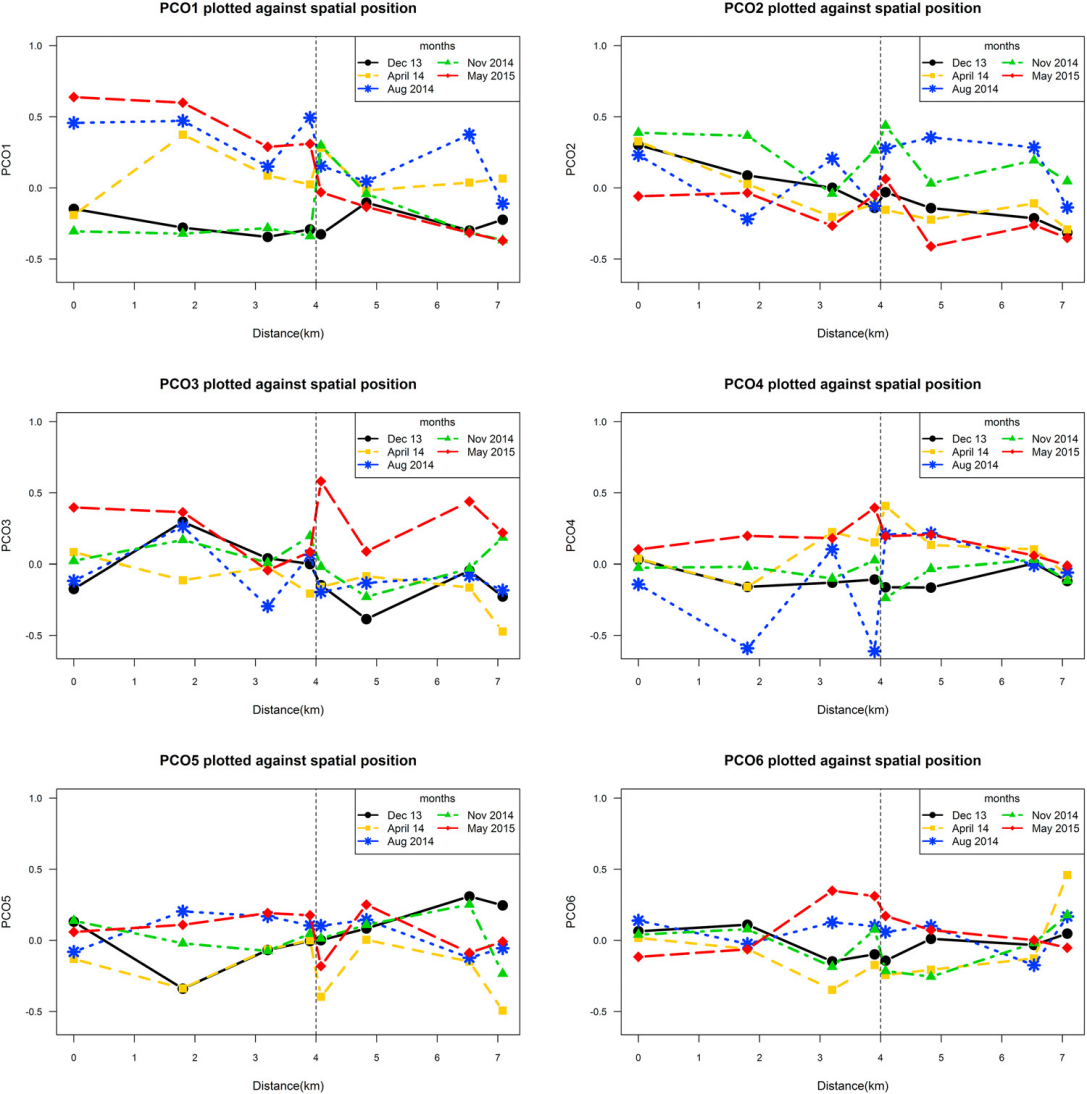
The first six PCO axesplotted against distance (space) [8].

Following Paul and Anderson [19], four diagnostic methods were employed to identify potentially nontrivial PCO axes that contain systematic patterns in community composition. The first method was the broken stick method, which uses the broken stick distribution [4,13]. Frontier’s model assumes that if the total variance in a multivariate dataset is divided at random among all components, the expected distribution of the eigenvalues can be assumed to follow a broken-stick distribution. PCOs with eigenvalues exceeding the broken stick values are assumed to exhibit patterns. The second method was the bootstrap approach, which re-samples observations, the rows of a site (row) × species (column) matrix, with replacement and estimates 95% confidence intervals for the distribution of eigenvalues from a PCO of the bootstrap samples. Where the confidence intervals did not overlap between pairs of successive eigenvalues, the eigenvalues were considered different. PCOs with these eigenvalues were considered to be nontrivial, potentially having structure worthy of interpretation [9]. The third method involved destroying the inter-correlations among the original species and building a null model of randomness by randomly and independently permuting the values in each column vector corresponding to each species in the original matrix. The next step was to construct the 95% confidence interval for the empirical distribution of each of the ordered PCO eigenvalues calculated under the null model of randomness. Any of the originally observed eigenvalues that exceeded this interval were assumed to be nontrivial. The fourth method computed the variation explained by each ranked PCO as the percentage of the variation accounted for by the remaining ranked PCOs rather than as a percentage of the total. Finally, a scree plot was used to plot the percent variation explained by each PCO against the rank order of each PCO (lines 292–441 in R). The first few PCO axes reveal any useful information about the macroinvertebrate community and the remaining axes usually contain random taxa fluctuations that can be considered “noise” [5,13]. For example, from Fig. 2 derived from Hatami [8], and from the results, the first two PCOs contained most of the information and the rest of the PCOs did not seem to show much systematic pattern.

The next stage of the exploratory analysis was to explore spatiotemporal patterns in the nontrivial PCO axes. All of the nontrivial PCO axes were plotted against time and spatial positions (lines 443–618 in R codes). Based on the spatiotemporal patterns observed in PCO axes and in environmental variables, PCOs were fitted and modelled as a function of each of the environmental variables, in combination with other variables and their interactions (lines 656–765). The exploratory analyses, and in particular building spatiotemporal plots of PCO axes, provide useful insight to choose the functional forms in the statistical models.

### Model building and testing

For model building and testing, a combination of principles of structural equation modelling and Bayesian network was employed following Pearl [22] and Paul and Anderson [20]. After exploratory analysis and visual inspection of the data, the process of structural equation modelling started with translating the relationships among variables in the causal diagram into a series of structural equations [22,25]. Every structural equation consists of a set of environmental variables (parents) in the causal diagram that directly determine the value of the variable of interest (child), plus the error due to the omitted factors [22]. The hypothesis underlying each arrow in the causal diagram was checked by testing the structural equations. The process of building the models required statistical models to be fitted and checked for each of the structural equations derived from a causal diagram [16,17]. A list of all structural equations derived from a causal diagram can be found in the link research paper [8]. In that list, the first structural equation was related to modelling macroinvertebrate community composition (calculated as PCO scores) as a function of its direct causes, i.e. all the environmental variables that macroinvertebrate community (as a response variable) received arrows from them in the causal diagram. In addition to building statistical models related to the structural equations, it was also important to build a spatiotemporal model for each variable.

In order to control for spatial and temporal confounding, PCO axes were modelled as a function of time, distance, effluent and their interactions. For doing so, first, a binary variable was created using *ifelse()* function to represent ‘effluent’ in the models. All the sites located upstream of a discharge point were assigned value 1, and all the downstream sites had the value of 0 (line 770–771 in R). For model building and testing of macroinvertebrate multivariate data, the *capscale()* function was employed to perform a distance-based Redundancy Analysis (dbRDA) on a Bray-Curtis dissimilarity matrix of square-root transformed abundance data [12,17,19] (lines 770–920 in R). Predicted values for the first six PCO axes, calculated from the spatiotemporal model, were plotted against distance to visually illustrate the degree of agreement between predicted and observed values (lines 921–1044 in R). To examine if any pattern of observed values remained unexplained by the model, residuals from the spatiotemporal model were plotted against distance and time (lines 1046–1078 in R).

A series of analyses were done for every nontrivial PCO to find a set of variables that could explain the spatiotemporal variation of macroinvertebrate community composition. This started with using scatter plot matrices to assess the evidence of correlations between macroinvertebrate community composition i.e. PCO scores of the chosen axes, land use, and environmental variables. Then, the relationships between all six nontrivial axes and every environmental variable were examined using linear regression. This was followed by multiple regression to examine how a combination of environmental variables and their interactions might be related to the PCO axes (lines 1080–2485 in R). Finally, dbRDA was performed to build a model of PCO scores as a function of a set of environmental variables that could account for most of their spatiotemporal variations. The *capscale()* function with the argument of *condition*, was used to check whether the environmental variables in dbRDA model could explain the spatiotemporal variations in PCOs. The residuals were checked visually and tested for any spatiotemporal patterns remained unexplained in dbRDA model after conditioning on the environmental variables (lines 2488–2570 in R). A mantel correlogram was computed using the *mgram*() function to examine spatial multivariate autocorrelation, and to check the assumption underpinning the permutation test that the errors were independent.

After finding environmental variables accounting for the spatiotemporal variations in macroinvertebrate structure, it was important to explore which taxa the PCO axes were represented for. To do so, the spatial variations of macroinvertebrate taxa and their association with PCO axes were checked. In addition, the relationships between PCO axes and several biotic indices such as Shannon diversity index, EPT taxon richness,^1^ EPT number, and SIGNAL^2^ index were examined using regression and Spearman correlation tests (Lines 2573–3405 in R). The changes in macroinvertebrate community composition across variations in PCOs were visualised in heat maps to illustrate the taxa responsible for this observed pattern (lines 2985–3075 in R).

The spearman correlations were used, in a series of scatter plot matrices, to decide which land use types were related to the variations of environmental variables and needed to be included in the models (lines 3408–3637 in R). Statistical analyses such as multiple regression were performed using the *lm()* function to fit the models for the environmental variables. The analysis of variance table and a summary of the results were produced using *anova()* and *summary()* functions. The assumptions underpinning the tests used for building the models and performing hypothesis tests were checked using diagnostic plots. Function *avPlots()* was also used to check the partial regression plots for diagnosing the fit of the models.

### Testing conditional independence constraints

After testing the existence of the direct relations included in the SEM, the conditional independencies needed to be checked. A graphical criterion called the *d*-separation criterion [21] is used to translate the missing arrows in the causal diagram into *d*-separation statements, which are graphical counterparts of the conditional independence relationships [25]. *d*-Separation is used to read the conditional independencies from DAGs, or a causal diagram, by providing sufficient conditions for two variables to be probabilistically independent upon conditioning on having knowledge about their parents [22,25]. That is, under this condition, known as a Markov condition, every two variables in the causal diagram can be probabilistically independent, given the state of their immediate causes [27]. Conditional independencies are about the independence of two variables conditional on the behaviour of other variables. The structural equation models explained in the previous sections revealed the statistical association between variables which related to arrows in causal diagram. Conditional independencies, which are representing themselves as missing arrows on the causal diagram, are about the constraints imposed by the structural equations on the data-generating process. Therefore, they are useful means to test the causal inferences made from data [22].

The process of producing and testing the conditional independence constraints can be done in several steps. This started with generating a list of conditional independence relationships. First, the causal diagram was revised by removing all the arrows between variables that did not show any statistical association. Another important difference between the initial causal diagram and the revised causal diagram in the application of this method from Hatami [8] was the addition of bidirectional arrows and the latent variable ‘treatment plant operation’ to the latter. Bidirectional arrows between the discharge variables implied that they were correlated because of their possible latent common cause, i.e. their marginal independence must result from a common cause [18,22]. The causal hypotheses underlying the arrows left in the revised causal diagram were written in the form of a DAG. The function *DAG()* was used to produce an adjacency matrix of a DAG, which is a square (1,0) matrix, with order equal to the number of nodes of the revised causal diagram. If there is an arrow between two variables, this function produces value 1 in position (i,j) in the matrix, and a zero if there is not any arrow [26]. This adjacency matrix of a DAG was used as an argument for *basiSet()* function to generate a basis set for conditional independencies (lines 5744–5770 in R). The number of conditional independencies derived from the revised causal diagram can be obtained by calculating the number of elements in the basis set, using this formula:

$$The\;number\;of\;elements\;in\;the\;basis\;set=\frac{V!}{2(v-2)!}-A$$

Where V is the number of variables, and A is the number of arrows in the revised causal diagram [26]. This helps to ensure if the number of generated conditional independencies was the same as those expected based on the number of nodes and missing arrows in the revised causal diagram. In addition, this was checked by using the function *graph.adjacency()* to graph the adjacency matrix and see if it was the same as the revised causal diagram.

To start testing the conditional independencies, they were written in the form of an equivalent set of *d*-separation statements. *d*-Separation statements are in the form of ‘X *des* Y|Z’ which means X is *d*-separated from Y, conditional on Z. A series of statistical tests were conducted to test the testable *d*-separation statements. The *capscale()* function with the argument *condition* was used to test if conditioning on a new variable could add more information to the dbRDA model. It was also tested if there were any variation remained in the residuals that could be explained by adding a variable to the model or not. For testing conditional independencies related to the environmental variables, nested regression models were compared using *anova()* function to test the hypothesis if a term should be removed or included in the model. In addition to checking the hypothesis underlying each missing arrow using the local tests (lines 5760–6617 in R), all independency relationships were tested simultaneously using a global test, called Fisher’s C test (lines 6623–6626 in R). The C-statistic and *p*-value of the global test were used to confirm the causal structure of the revised causal diagram by checking the composite hypothesis entailed in the set of conditional independence constraints [25,26]. If the results of local and global tests confirm the structure of composite hypotheses entailed in the causal diagram, then the models are known as causal and can be used for predicting the effect of interventions and counterfactual analysis. The given causal models can be used to predict the effect of a potential intervention; for example, to estimate the concentration of nutrients in the river if the corresponding discharge loads in effluent are divided by half [17]. Water quality variables can be predicted when an imaginary or counterfactual situation is imposed on the models. An application of counterfactual analysis in a risk assessment study can be found in Hatami [7], where the concentrations of pollutants were manipulated to answer management questions, e.g. what would have the response of macroinvertebrate communities in the stream been had there not been any effluent discharged to the river. Testing and revising of the models were done in an iterative process based on the results of local and global hypothesis tests, partial regression test, and cross validation test. During the process of model selection and revising the models, a combination of statistical tests and causal consideration was used. That is, statistical tests were used for model selection, and decision-making about which variables to include in the model were guided by causal considerations based on literature. Several packages, and in particular ‘ggm’ package [14], were used for the process of generating and testing of conditional independencies (lines 5730–5738 in R).

## Footnotes

1 The number of taxa belonging to sensitive orders Plecoptera, Trichoptera, and Ephemeroptera

2 Stream Invertebrate Grade Number – Average Level
