# Supplementary material for: A practical method to control spatiotemporal confounding in environmental impact studies
Source: MethodsX. 2018 Jul 5;5:710–6. doi: 10.1016/j.mex.2018.07.003 (PMC6070679; doi:10.1016/j.mex.2018.07.003)
Supplement: Supplementary file 3 [file mmc3.docx]

##Set working directory according to the filepath where data files are stored

setwd()

getwd()

###Load packages######

library(vegan)

library(car)

library(lattice)

library(ecodist)

library(BiodiversityR)

library(latticeExtra)

library(WhatIf)

library(bnlearn)

#########

library(Hmisc)

library(car)

library(bnlearn)

library(pspearman)

library(Rgraphviz)

library("Rgraphviz")

###Read data########

wangbug<-read.csv("wangbug.csv",header=TRUE,nrows=40)

View(wangbug)

edit(wangbug)

dim(wangbug)

wangenv<-read.csv("wangenv.csv",header=TRUE,nrows=40)

View(wangenv)

edit(wangenv)

diversity<-read.csv("diversity.csv",header=TRUE,nrows=40)

edit(diversity)

## Exploratory data analysis#########

## Environmental variables graphs

#********#nitrogen and phosphate graph#********#

tiff(file="nutrients.tif",width=14,height=14,units="in",pointsize = 12,bg ="transparent",res=800,compression="lzw")

par(mfrow=c(3,2), mar=c(0.1,0.1,0.1,0.1),cex=0.6,cex.axis=0.8,las=1)

par(mar=c(5,5,4,2),cex=0.9)

plot(wangenv[wangenv$day==1,"dist"],wangenv[wangenv$day==1,"tn"],type = "b",ylim=c(0,3.5),pch=19,cex=1.5,xlab="Distance(km)",ylab="TN(mg/L)",main="a) Total Nitrogen plotted against spatial position",lwd=3,lty=1)

points(wangenv[wangenv$day==126,"dist"],wangenv[wangenv$day==126,"tn"],type="b",col="gold2",pch=15,lwd=3,cex=1.5,lty=2)

points(wangenv[wangenv$day==260,"dist"],wangenv[wangenv$day==260,"tn"],type="b",col="blue",pch=8,cex=1.5,lwd=3,lty=3)

points(wangenv[wangenv$day==336,"dist"],wangenv[wangenv$day==336,"tn"],type="b",col="green3",pch=17,cex=1.5,lwd=3,lty=4)

points(wangenv[wangenv$day==518,"dist"],wangenv[wangenv$day==518,"tn"],type="b",col="red",pch=18,cex=2,lwd=3,lty=5)

legend("topright",inset=c(0,0),legend=c("Dec 13", "April 14", "Aug 2014", "Nov 2014","May 2015"),lty=c(1,5),pch=c(19,15,8,17,18),lwd=2,col=c("black","gold2","blue","green3","red"),ncol=2,horiz=FALSE,cex=0.9,title="months")

abline(v=4,lty=2)

par(mar=c(5,5,4,2),cex=0.9)

plot(wangenv[wangenv$day==1,"dist"],wangenv[wangenv$day==1,"no2"],type = "b",ylim=c(0,.7),pch=19,cex=1.5,xlab="Distance(km)",ylab="NO2(mg/L)",main="b) Nitrite plotted against spatial position",lwd=3,lty=1)

points(wangenv[wangenv$day==126,"dist"],wangenv[wangenv$day==126,"no2"],type="b",col="gold2",pch=15,lwd=3,cex=1.5,lty=2)

points(wangenv[wangenv$day==260,"dist"],wangenv[wangenv$day==260,"no2"],type="b",col="blue",pch=8,cex=1.5,lwd=3,lty=3)

points(wangenv[wangenv$day==336,"dist"],wangenv[wangenv$day==336,"no2"],type="b",col="green3",pch=17,cex=1.5,lwd=3,lty=4)

points(wangenv[wangenv$day==518,"dist"],wangenv[wangenv$day==518,"no2"],type="b",col="red",pch=18,cex=2,lwd=3,lty=5)

legend("topright",inset=c(0,0),legend=c("Dec 13", "April 14", "Aug 2014", "Nov 2014","May 2015"),lty=c(1,5),pch=c(19,15,8,17,18),lwd=2,col=c("black","gold2","blue","green3","red"),ncol=2,horiz=FALSE,cex=0.9,title="months")

abline(v=4,lty=2)

par(mar=c(5,5,4,2),cex=0.9)

plot(wangenv[wangenv$day==1,"dist"],wangenv[wangenv$day==1,"no3"],type = "b",ylim=c(0,.7),pch=19,cex=1.5,xlab="Distance(km)",ylab="NO3(mg/L)",main="c) Nitrate plotted against spatial position",lwd=3,lty=1)

points(wangenv[wangenv$day==126,"dist"],wangenv[wangenv$day==126,"no3"],type="b",col="gold2",pch=15,lwd=3,cex=1.5,lty=2)

points(wangenv[wangenv$day==260,"dist"],wangenv[wangenv$day==260,"no3"],type="b",col="blue",pch=8,cex=1.5,lwd=3,lty=3)

points(wangenv[wangenv$day==336,"dist"],wangenv[wangenv$day==336,"no3"],type="b",col="green3",pch=17,cex=1.5,lwd=3,lty=4)

points(wangenv[wangenv$day==518,"dist"],wangenv[wangenv$day==518,"no3"],type="b",col="red",pch=18,cex=2,lwd=3,lty=5)

legend("topright",inset=c(0,0),legend=c("Dec 13", "April 14", "Aug 2014", "Nov 2014","May 2015"),lty=c(1,5),pch=c(19,15,8,17,18),lwd=2,col=c("black","gold2","blue","green3","red"),ncol=2,horiz=FALSE,cex=0.9,title="months")

abline(v=4,lty=2)

par(mar=c(5,5,4,2),cex=0.9)

plot(wangenv[wangenv$day==1,"dist"],wangenv[wangenv$day==1,"nh3"],type = "b",ylim=c(0,.7),pch=19,cex=1.5,xlab="Distance(km)",ylab="NH3(mg/L)",main="d) Ammonia plotted against spatial position",lwd=3,lty=4)

points(wangenv[wangenv$day==126,"dist"],wangenv[wangenv$day==126,"nh3"],type="b",col="gold2",pch=15,lwd=3,cex=1.5,lty=2)

points(wangenv[wangenv$day==260,"dist"],wangenv[wangenv$day==260,"nh3"],type="b",col="blue",pch=8,cex=1.5,lwd=3,lty=3)

points(wangenv[wangenv$day==336,"dist"],wangenv[wangenv$day==336,"nh3"],type="b",col="green3",pch=17,cex=1.5,lwd=3,lty=4)

points(wangenv[wangenv$day==518,"dist"],wangenv[wangenv$day==518,"nh3"],type="b",col="red",pch=18,cex=2,lwd=3,lty=5)

legend("topright",inset=c(0,0),legend=c("Dec 13", "April 14", "Aug 2014", "Nov 2014","May 2015"),lty=c(1,5),pch=c(19,15,8,17,18),lwd=2,col=c("black","gold2","blue","green3","red"),ncol=2,horiz=FALSE,cex=0.9,title="months")

abline(v=4,lty=2)

par(mar=c(5,5,4,2),cex=0.9)

plot(wangenv[wangenv$day==1,"dist"],wangenv[wangenv$day==1,"op"],type = "b",ylim=c(0,0.08),pch=19,cex=1.5,xlab="Distance(km)",ylab="Orthoposphate(mg/L)",main="e) Orthoposphate plotted against spatial position",lwd=3,lty=4)

points(wangenv[wangenv$day==126,"dist"],wangenv[wangenv$day==126,"op"],type="b",col="gold2",pch=15,lwd=3,cex=1.5,lty=2)

points(wangenv[wangenv$day==260,"dist"],wangenv[wangenv$day==260,"op"],type="b",col="blue",pch=8,cex=1.5,lwd=3,lty=3)

points(wangenv[wangenv$day==336,"dist"],wangenv[wangenv$day==336,"op"],type="b",col="green3",pch=17,cex=1.5,lwd=3,lty=4)

points(wangenv[wangenv$day==518,"dist"],wangenv[wangenv$day==518,"op"],type="b",col="red",pch=18,cex=2,lwd=3,lty=5)

legend("topright",inset=c(0,0),legend=c("Dec 13", "April 14", "Aug 2014", "Nov 2014","May 2015"),lty=c(1,5),pch=c(19,15,8,17,18),lwd=2,col=c("black","gold2","blue","green3","red"),ncol=2,horiz=FALSE,cex=0.9,title="months")

abline(v=4,lty=2)

par(mar=c(5,5,4,2),cex=0.9)

plot(wangenv[wangenv$day==1,"dist"],wangenv[wangenv$day==1,"tp"],type = "b",ylim=c(0,0.7),pch=19,cex=1.5,xlab="Distance(km)",ylab="TP",main="f) Total Phosphorus plotted against spatial position",lwd=3,lty=1)

points(wangenv[wangenv$day==126,"dist"],wangenv[wangenv$day==126,"tp"],type="b",col="gold2",pch=15,lwd=3,cex=1.5,lty=2)

points(wangenv[wangenv$day==260,"dist"],wangenv[wangenv$day==260,"tp"],type="b",col="blue",pch=8,cex=1.5,lwd=3,lty=3)

points(wangenv[wangenv$day==336,"dist"],wangenv[wangenv$day==336,"tp"],type="b",col="green3",pch=17,cex=1.5,lwd=3,lty=4)

points(wangenv[wangenv$day==518,"dist"],wangenv[wangenv$day==518,"tp"],type="b",col="red",pch=18,cex=2,lwd=3,lty=5)

legend("topright",inset=c(0,0),legend=c("Dec 13", "April 14", "Aug 2014", "Nov 2014","May 2015"),lty=c(1,5),pch=c(19,15,8,17,18),lwd=2,col=c("black","gold2","blue","green3","red"),ncol=2,horiz=FALSE,cex=0.9,title="months")

abline(v=4,lty=2)

dev.off()

#heavy metals, cod and antimony

tiff(file="heavy metals, cod, and antimony.tif",width=14,height=14,units="in",pointsize = 12,bg ="transparent",res=800,compression="lzw")

par(mfrow=c(3,2), mar=c(0.1,0.1,0.1,0.1),cex=0.6,cex.axis=0.8,las=1)

par(mar=c(5,5,4,2),cex=0.9)

plot(wangenv[wangenv$day==1,"dist"],wangenv[wangenv$day==1,"cd"],type = "b",ylim=c(0,1.5),pch=19,cex=1.5,xlab="Distance(km)",ylab="Cd (mg/kg)",main="a) Cadmium plotted against spatial position",lwd=3,lty=1)

points(wangenv[wangenv$day==126,"dist"],wangenv[wangenv$day==126,"cd"],type="b",col="gold2",pch=15,lwd=3,cex=1.5,lty=2)

points(wangenv[wangenv$day==260,"dist"],wangenv[wangenv$day==260,"cd"],type="b",col="blue",pch=8,cex=1.5,lwd=3,lty=3)

points(wangenv[wangenv$day==336,"dist"],wangenv[wangenv$day==336,"cd"],type="b",col="green3",pch=17,cex=1.5,lwd=3,lty=4)

points(wangenv[wangenv$day==518,"dist"],wangenv[wangenv$day==518,"cd"],type="b",col="red",pch=18,cex=2,lwd=3,lty=5)

legend("topright",inset=c(0,0),legend=c("Dec 13", "April 14", "Aug 2014", "Nov 2014","May 2015"),lty=c(1,5),pch=c(19,15,8,17,18),lwd=2,col=c("black","gold2","blue","green3","red"),ncol=2,horiz=FALSE,cex=0.9,title="months")

abline(v=4,lty=2)

par(mar=c(5,5,4,2),cex=0.9)

plot(wangenv[wangenv$day==1,"dist"],wangenv[wangenv$day==1,"cr"],type = "b",ylim=c(0,10),pch=19,cex=1.5,xlab="Distance(km)",ylab="Cr (mg/kg)",main="b) Chromium plotted against spatial position",lwd=3,lty=1)

points(wangenv[wangenv$day==126,"dist"],wangenv[wangenv$day==126,"cr"],type="b",col="gold2",pch=15,lwd=3,cex=1.5,lty=2)

points(wangenv[wangenv$day==260,"dist"],wangenv[wangenv$day==260,"cr"],type="b",col="blue",pch=8,cex=1.5,lwd=3,lty=3)

points(wangenv[wangenv$day==336,"dist"],wangenv[wangenv$day==336,"cr"],type="b",col="green3",pch=17,cex=1.5,lwd=3,lty=4)

points(wangenv[wangenv$day==518,"dist"],wangenv[wangenv$day==518,"cr"],type="b",col="red",pch=18,cex=2,lwd=3,lty=5)

legend("topright",inset=c(0,0),legend=c("Dec 13", "April 14", "Aug 2014", "Nov 2014","May 2015"),lty=c(1,5),pch=c(19,15,8,17,18),lwd=2,col=c("black","gold2","blue","green3","red"),ncol=2,horiz=FALSE,cex=0.9,title="months")

abline(v=4,lty=2)

par(mar=c(5,5,4,2),cex=0.9)

plot(wangenv[wangenv$day==1,"dist"],wangenv[wangenv$day==1,"cu"],type = "b",ylim=c(0,16),pch=19,cex=1.5,xlab="Distance(km)",ylab="Cu (mg/kg)",main="c) Copper plotted against spatial position",lwd=3,lty=1)

points(wangenv[wangenv$day==126,"dist"],wangenv[wangenv$day==126,"cu"],type="b",col="gold2",pch=15,lwd=3,cex=1.5,lty=2)

points(wangenv[wangenv$day==260,"dist"],wangenv[wangenv$day==260,"cu"],type="b",col="blue",pch=8,cex=1.5,lwd=3,lty=3)

points(wangenv[wangenv$day==336,"dist"],wangenv[wangenv$day==336,"cu"],type="b",col="green3",pch=17,cex=1.5,lwd=3,lty=4)

points(wangenv[wangenv$day==518,"dist"],wangenv[wangenv$day==518,"cu"],type="b",col="red",pch=18,cex=2,lwd=3,lty=5)

legend("topright",inset=c(0,0),legend=c("Dec 13", "April 14", "Aug 2014", "Nov 2014","May 2015"),lty=c(1,5),pch=c(19,15,8,17,18),lwd=2,col=c("black","gold2","blue","green3","red"),ncol=2,horiz=FALSE,cex=0.9,title="months")

abline(v=4,lty=2)

par(mar=c(5,5,4,2),cex=0.9)

plot(wangenv[wangenv$day==1,"dist"],wangenv[wangenv$day==1,"zn"],type = "b",ylim=c(0,120),pch=19,cex=1.5,xlab="Distance(km)",ylab="Zn (mg/kg)",main="d) Zinc plotted against spatial position",lwd=3,lty=4)

points(wangenv[wangenv$day==126,"dist"],wangenv[wangenv$day==126,"zn"],type="b",col="gold2",pch=15,lwd=3,cex=1.5,lty=2)

points(wangenv[wangenv$day==260,"dist"],wangenv[wangenv$day==260,"zn"],type="b",col="blue",pch=8,cex=1.5,lwd=3,lty=3)

points(wangenv[wangenv$day==336,"dist"],wangenv[wangenv$day==336,"zn"],type="b",col="green3",pch=17,cex=1.5,lwd=3,lty=4)

points(wangenv[wangenv$day==518,"dist"],wangenv[wangenv$day==518,"zn"],type="b",col="red",pch=18,cex=2,lwd=3,lty=5)

legend("topright",inset=c(0,0),legend=c("Dec 13", "April 14", "Aug 2014", "Nov 2014","May 2015"),lty=c(1,5),pch=c(19,15,8,17,18),lwd=2,col=c("black","gold2","blue","green3","red"),ncol=2,horiz=FALSE,cex=0.9,title="months")

abline(v=4,lty=2)

par(mar=c(5,5,4,2),cex=0.9)

plot(wangenv[wangenv$day==1,"dist"],wangenv[wangenv$day==1,"sb"],type = "b",ylim=c(0,0.2),pch=19,cex=1.5,xlab="Distance(km)",ylab="Antimony (mg/L)",main="e) Antimony plotted against spatial position",lwd=3,lty=4)

points(wangenv[wangenv$day==126,"dist"],wangenv[wangenv$day==126,"sb"],type="b",col="gold2",pch=15,lwd=3,cex=1.5)

points(wangenv[wangenv$day==260,"dist"],wangenv[wangenv$day==260,"sb"],type="b",col="blue",pch=8,cex=1.5,lwd=3,lty=3)

points(wangenv[wangenv$day==336,"dist"],wangenv[wangenv$day==336,"sb"],type="b",col="green3",pch=17,cex=1.5,lwd=3)

points(wangenv[wangenv$day==518,"dist"],wangenv[wangenv$day==518,"sb"],type="b",col="red",pch=18,cex=2,lwd=3,lty=5)

legend("topright",inset=c(0,0),legend=c("Dec 13", "April 14", "Aug 2014", "Nov 2014","May 2015"),lty=c(1,5),pch=c(19,15,8,17,18),lwd=2,col=c("black","gold2","blue","green3","red"),ncol=2,horiz=FALSE,cex=0.9,title="months")

abline(v=4,lty=2)

par(mar=c(5,5,4,2),cex=0.9)

plot(wangenv[wangenv$day==1,"dist"],wangenv[wangenv$day==1,"cod"],type = "b",ylim=c(0,100),pch=19,cex=1.5,xlab="Distance(km)",ylab="COD",main="f) COD (mg/L) plotted against spatial position",lwd=3,lty=4)

points(wangenv[wangenv$day==126,"dist"],wangenv[wangenv$day==126,"cod"],type="b",col="gold2",pch=15,lwd=3,cex=1.5)

points(wangenv[wangenv$day==260,"dist"],wangenv[wangenv$day==260,"cod"],type="b",col="blue",pch=8,cex=1.5,lwd=3,lty=3)

points(wangenv[wangenv$day==336,"dist"],wangenv[wangenv$day==336,"cod"],type="b",col="green3",pch=17,cex=1.5,lwd=3)

points(wangenv[wangenv$day==518,"dist"],wangenv[wangenv$day==518,"cod"],type="b",col="red",pch=18,cex=2,lwd=3,lty=5)

legend("topright",inset=c(0,0),legend=c("Dec 13", "April 14", "Aug 2014", "Nov 2014","May 2015"),lty=c(1,5),pch=c(19,15,8,17,18),lwd=2,col=c("black","gold2","blue","green3","red"),ncol=2,horiz=FALSE,cex=0.9,title="months")

abline(v=4,lty=2)

dev.off()

#### ph, temperature, conductivity, alkalinity, toc, and chla

tiff(file="ph, temperature, conductivity, alkalinity, toc, and chla.jpg",width=14,height=14,units="in",pointsize = 12,bg ="transparent",res=800,compression="lzw")

par(mfrow=c(3,2), mar=c(0.1,0.1,0.1,0.1),cex=0.6,cex.axis=0.8,las=1)

par(mar=c(5,5,4,2),cex=0.9)

par(mar=c(5,5,4,2),cex=0.9)

plot(wangenv[wangenv$day==1,"dist"],wangenv[wangenv$day==1,"temp"],type = "b",ylim=c(5,25),pch=19,cex=1.5,xlab="Distance(km)",ylab="Temperature (°C)",main="a) Temperature plotted against spatial position",lwd=3,lty=1)

points(wangenv[wangenv$day==126,"dist"],wangenv[wangenv$day==126,"temp"],type="b",col="gold2",pch=15,lwd=3,cex=1.5,lty=2)

points(wangenv[wangenv$day==260,"dist"],wangenv[wangenv$day==260,"temp"],type="b",col="blue",pch=8,cex=1.5,lwd=3,lty=3)

points(wangenv[wangenv$day==336,"dist"],wangenv[wangenv$day==336,"temp"],type="b",col="green3",pch=17,cex=1.5,lwd=3,lty=4)

points(wangenv[wangenv$day==518,"dist"],wangenv[wangenv$day==518,"temp"],type="b",col="red",pch=18,cex=2,lwd=3,lty=5)

legend("topright",inset=c(0,0),legend=c("Dec 13", "April 14", "Aug 2014", "Nov 2014","May 2015"),lty=c(1,5),pch=c(19,15,8,17,18),lwd=2,col=c("black","gold2","blue","green3","red"),ncol=2,horiz=FALSE,cex=0.9,title="months")

abline(v=4,lty=2)

par(mar=c(5,5,4,2),cex=0.9)

plot(wangenv[wangenv$day==1,"dist"],wangenv[wangenv$day==1,"ph2"],type = "b",ylim=c(6.5,8.5),pch=19,cex=1.5,xlab="Distance(km)",ylab="pH",main="b) pH plotted against spatial position",lwd=3,lty=1)

points(wangenv[wangenv$day==126,"dist"],wangenv[wangenv$day==126,"ph2"],type="b",col="gold2",pch=15,lwd=3,cex=1.5,lty=2)

points(wangenv[wangenv$day==260,"dist"],wangenv[wangenv$day==260,"ph2"],type="b",col="blue",pch=8,cex=1.5,lwd=3,lty=3)

points(wangenv[wangenv$day==336,"dist"],wangenv[wangenv$day==336,"ph2"],type="b",col="green3",pch=17,cex=1.5,lwd=3,lty=4)

points(wangenv[wangenv$day==518,"dist"],wangenv[wangenv$day==518,"ph2"],type="b",col="red",pch=18,cex=2,lwd=3,lty=5)

legend("topright",inset=c(0,0),legend=c("Dec 13", "April 14", "Aug 2014", "Nov 2014","May 2015"),lty=c(1,5),pch=c(19,15,8,17,18),lwd=2,col=c("black","gold2","blue","green3","red"),ncol=2,horiz=FALSE,cex=0.9,title="months")

abline(v=4,lty=2)

par(mar=c(5,5,4,2),cex=0.9)

plot(wangenv[wangenv$day==1,"dist"],wangenv[wangenv$day==1,"alk"],type = "b",ylim=c(0,200),pch=19,cex=1.5,xlab="Distance(km)",ylab="Alkalinity (mg/L)",main="c) Alkalinity plotted against spatial position",lwd=3,lty=1)

points(wangenv[wangenv$day==126,"dist"],wangenv[wangenv$day==126,"alk"],type="b",col="gold2",pch=15,lwd=3,cex=1.5,lty=2)

points(wangenv[wangenv$day==260,"dist"],wangenv[wangenv$day==260,"alk"],type="b",col="blue",pch=8,cex=1.5,lwd=3,lty=3)

points(wangenv[wangenv$day==336,"dist"],wangenv[wangenv$day==336,"alk"],type="b",col="green3",pch=17,cex=1.5,lwd=3,lty=4)

points(wangenv[wangenv$day==518,"dist"],wangenv[wangenv$day==518,"alk"],type="b",col="red",pch=18,cex=2,lwd=3,lty=5)

legend("topright",inset=c(0,0),legend=c("Dec 13", "April 14", "Aug 2014", "Nov 2014","May 2015"),lty=c(1,5),pch=c(19,15,8,17,18),lwd=2,col=c("black","gold2","blue","green3","red"),ncol=2,horiz=FALSE,cex=0.9,title="months")

abline(v=4,lty=2)

par(mar=c(5,5,4,2),cex=0.9)

plot(wangenv[wangenv$day==1,"dist"],wangenv[wangenv$day==1,"chla"],type = "b",ylim=c(0,40),pch=19,cex=1.5,xlab="Distance(km)",ylab="Chlorophyll A (mg/L)",main="d) Chlorophyll A plotted against spatial position",lwd=3,lty=1)

points(wangenv[wangenv$day==126,"dist"],wangenv[wangenv$day==126,"chla"],type="b",col="gold2",pch=15,lwd=3,cex=1.5,lty=2)

points(wangenv[wangenv$day==260,"dist"],wangenv[wangenv$day==260,"chla"],type="b",col="blue",pch=8,cex=1.5,lwd=3,lty=3)

points(wangenv[wangenv$day==336,"dist"],wangenv[wangenv$day==336,"chla"],type="b",col="green3",pch=17,cex=1.5,lwd=3,lty=4)

points(wangenv[wangenv$day==518,"dist"],wangenv[wangenv$day==518,"chla"],type="b",col="red",pch=18,cex=2,lwd=3,lty=5)

legend("topright",inset=c(0,0),legend=c("Dec 13", "April 14", "Aug 2014", "Nov 2014","May 2015"),lty=c(1,5),pch=c(19,15,8,17,18),lwd=2,col=c("black","gold2","blue","green3","red"),ncol=2,horiz=FALSE,cex=0.9,title="months")

abline(v=4,lty=2)

par(mar=c(5,5,4,2),cex=0.9)

plot(wangenv[wangenv$day==1,"dist"],wangenv[wangenv$day==1,"toc"],type = "b",ylim=c(0,12),pch=19,cex=1.5,xlab="Distance(km)",ylab="TOC(mg/L)",main="e) Total Organic Carbon plotted against spatial position",lwd=3,lty=1)

points(wangenv[wangenv$day==126,"dist"],wangenv[wangenv$day==126,"toc"],type="b",col="gold2",pch=15,lwd=3,cex=1.5,lty=2)

points(wangenv[wangenv$day==260,"dist"],wangenv[wangenv$day==260,"toc"],type="b",col="blue",pch=8,cex=1.5,lwd=3,lty=3)

points(wangenv[wangenv$day==336,"dist"],wangenv[wangenv$day==336,"toc"],type="b",col="green3",pch=17,cex=1.5,lwd=3,lty=4)

points(wangenv[wangenv$day==518,"dist"],wangenv[wangenv$day==518,"toc"],type="b",col="red",pch=18,cex=2,lwd=3,lty=5)

legend("topright",inset=c(0,0),legend=c("Dec 13", "April 14", "Aug 2014", "Nov 2014","May 2015"),lty=c(1,5),pch=c(19,15,8,17,18),lwd=2,col=c("black","gold2","blue","green3","red"),ncol=2,horiz=FALSE,cex=0.9,title="months")

abline(v=4,lty=2)

par(mar=c(5,5,4,2),cex=0.9)

plot(wangenv[wangenv$day==1,"dist"],wangenv[wangenv$day==1,"cond"],type = "b",ylim=c(0,700),pch=19,cex=1.5,xlab="Distance(km)",ylab="Conductivity (mg/L)",main="f) Conductivity plotted against spatial position",lwd=3,lty=1)

points(wangenv[wangenv$day==126,"dist"],wangenv[wangenv$day==126,"cond"],type="b",col="gold2",pch=15,lwd=3,cex=1.5,lty=2)

points(wangenv[wangenv$day==260,"dist"],wangenv[wangenv$day==260,"cond"],type="b",col="blue",pch=8,cex=1.5,lwd=3,lty=3)

points(wangenv[wangenv$day==336,"dist"],wangenv[wangenv$day==336,"cond"],type="b",col="green3",pch=17,cex=1.5,lwd=3,lty=4)

points(wangenv[wangenv$day==518,"dist"],wangenv[wangenv$day==518,"cond"],type="b",col="red",pch=18,cex=2,lwd=3,lty=5)

legend("topright",inset=c(0,0),legend=c("Dec 13", "April 14", "Aug 2014", "Nov 2014","May 2015"),lty=c(1,5),pch=c(19,15,8,17,18),lwd=2,col=c("black","gold2","blue","green3","red"),ncol=2,horiz=FALSE,cex=0.9,title="months")

abline(v=4,lty=2)

dev.off()

### DO, canop, velocity and turbidity

tiff(file="DO, canop, velocity and turbidity.jpg",width=14,height=14,units="in",pointsize = 12,bg ="transparent",res=800,compression="lzw")

par(mfrow=c(3,2), mar=c(0.1,0.1,0.1,0.1),cex=0.6,cex.axis=0.8,las=1)

par(mar=c(5,5,4,2),cex=0.9)

plot(wangenv[wangenv$day==1,"dist"],wangenv[wangenv$day==1,"vel"],type = "b",ylim=c(0,0.8),pch=19,cex=1.5,xlab="Distance(km)",ylab="Velocity (m/s)",main="a) Velocity plotted against spatial position",lwd=3,lty=4)

points(wangenv[wangenv$day==126,"dist"],wangenv[wangenv$day==126,"vel"],type="b",col="gold2",pch=15,lwd=3,cex=1.5)

points(wangenv[wangenv$day==260,"dist"],wangenv[wangenv$day==260,"vel"],type="b",col="blue",pch=8,cex=1.5,lwd=3,lty=3)

points(wangenv[wangenv$day==336,"dist"],wangenv[wangenv$day==336,"vel"],type="b",col="green3",pch=17,cex=1.5,lwd=3)

points(wangenv[wangenv$day==518,"dist"],wangenv[wangenv$day==518,"vel"],type="b",col="red",pch=18,cex=2,lwd=3,lty=5)

legend("topright",inset=c(0,0),legend=c("Dec 2013", "April 2014", "Aug 2014", "Nov 2014","May 2015"),lty=1,lwd=2,col=c("black","gold2","blue","green3","red"),ncol=5,horiz=FALSE,cex=0.8,title="months")

abline(v=4,lty=2)

par(mar=c(5,5,4,2),cex=0.9)

plot(wangenv[wangenv$day==1,"dist"],wangenv[wangenv$day==1,"turb"],type = "b",ylim=c(0,100),pch=19,cex=1.5,xlab="Distance(km)",ylab="Turbidity (NTU)",main="b) Turbidity plotted against spatial position",lwd=3,lty=4)

points(wangenv[wangenv$day==126,"dist"],wangenv[wangenv$day==126,"turb"],type="b",col="gold2",pch=15,lwd=3,cex=1.5)

points(wangenv[wangenv$day==260,"dist"],wangenv[wangenv$day==260,"turb"],type="b",col="blue",pch=8,cex=1.5,lwd=3,lty=3)

points(wangenv[wangenv$day==336,"dist"],wangenv[wangenv$day==336,"turb"],type="b",col="green3",pch=17,cex=1.5,lwd=3)

points(wangenv[wangenv$day==518,"dist"],wangenv[wangenv$day==518,"turb"],type="b",col="red",pch=18,cex=2,lwd=3,lty=5)

legend("topright",inset=c(0,0),legend=c("Dec 2013", "April 2014", "Aug 2014", "Nov 2014","May 2015"),lty=1,lwd=2,col=c("black","gold2","blue","green3","red"),ncol=5,horiz=FALSE,cex=0.8,title="months")

abline(v=4,lty=2)

par(mar=c(5,5,4,2),cex=0.9)

plot(wangenv[wangenv$day==1,"dist"],wangenv[wangenv$day==1,"sed"],type = "b",ylim=c(0.5,2.5),pch=19,cex=1.5,xlab="Distance(km)",ylab="Sediment size (mm)",main="c) Sediment size plotted against spatial position",lwd=3,lty=4)

points(wangenv[wangenv$day==126,"dist"],wangenv[wangenv$day==126,"sed"],type="b",col="gold2",pch=15,lwd=3,cex=1.5)

points(wangenv[wangenv$day==260,"dist"],wangenv[wangenv$day==260,"sed"],type="b",col="blue",pch=8,cex=1.5,lwd=3,lty=3)

points(wangenv[wangenv$day==336,"dist"],wangenv[wangenv$day==336,"sed"],type="b",col="green3",pch=17,cex=1.5,lwd=3)

points(wangenv[wangenv$day==518,"dist"],wangenv[wangenv$day==518,"sed"],type="b",col="red",pch=18,cex=2,lwd=3,lty=5)

legend("topright",inset=c(0,0),legend=c("Dec 2013", "April 2014", "Aug 2014", "Nov 2014","May 2015"),lty=1,lwd=2,col=c("black","gold2","blue","green3","red"),ncol=5,horiz=FALSE,cex=0.8,title="months")

abline(v=4,lty=2)

par(mar=c(5,5,4,2),cex=0.9)

plot(wangenv[wangenv$day==1,"dist"],wangenv[wangenv$day==1,"canop"],type = "b",ylim=c(0,100),pch=19,cex=1.5,xlab="Distance(km)",ylab="Canopy cover (%)",main="d) Canopy cover plotted against spatial position",lwd=3,lty=4)

points(wangenv[wangenv$day==126,"dist"],wangenv[wangenv$day==126,"canop"],type="b",col="gold2",pch=15,lwd=3,cex=1.5)

points(wangenv[wangenv$day==260,"dist"],wangenv[wangenv$day==260,"canop"],type="b",col="blue",pch=8,cex=1.5,lwd=3,lty=3)

points(wangenv[wangenv$day==336,"dist"],wangenv[wangenv$day==336,"canop"],type="b",col="green3",pch=17,cex=1.5,lwd=3)

points(wangenv[wangenv$day==518,"dist"],wangenv[wangenv$day==518,"canop"],type="b",col="red",pch=18,cex=2,lwd=3,lty=5)

legend("topright",inset=c(0,0),legend=c("Dec 13", "April 14", "Aug 2014", "Nov 2014","May 2015"),lty=c(1,5),pch=c(19,15,8,17,18),lwd=2,col=c("black","gold2","blue","green3","red"),ncol=2,horiz=FALSE,cex=0.9,title="months")

abline(v=4,lty=2)

plot(wangenv[wangenv$day==1,"dist"],wangenv[wangenv$day==1,"do"],type = "b",ylim=c(4,20),pch=19,cex=1.5,xlab="Distance(km)",ylab="Dissolved Oxygen (mg/L)",main="e) DO plotted against spatial position",lwd=3,lty=4)

points(wangenv[wangenv$day==126,"dist"],wangenv[wangenv$day==126,"do"],type="b",col="gold2",pch=15,lwd=3,cex=1.5,lty=2)

points(wangenv[wangenv$day==260,"dist"],wangenv[wangenv$day==260,"do"],type="b",col="blue",pch=8,cex=1.5,lwd=3,lty=3)

points(wangenv[wangenv$day==336,"dist"],wangenv[wangenv$day==336,"do"],type="b",col="green3",pch=17,cex=1.5,lwd=3,lty=4)

points(wangenv[wangenv$day==518,"dist"],wangenv[wangenv$day==518,"do"],type="b",col="red",pch=18,cex=2,lwd=3,lty=5)

legend("topright",inset=c(0,0),legend=c("Dec 13", "April 14", "Aug 2014", "Nov 2014","May 2015"),lty=c(1,5),pch=c(19,15,8,17,18),lwd=2,col=c("black","gold2","blue","green3","red"),ncol=2,horiz=FALSE,cex=0.9,title="months")

abline(v=4,lty=2)

par(mar=c(5,5,4,2),cex=0.9)

plot(wangenv[wangenv$day==1,"dist"],wangenv[wangenv$day==1,"cfpom"],type = "b",ylim=c(0,50),pch=19,cex=1.5,xlab="Distance(km)",ylab="CPOM/ FPOM",main="f) CPOM/FPOM plotted against spatial position",lwd=3,lty=4)

points(wangenv[wangenv$day==126,"dist"],wangenv[wangenv$day==126,"cfpom"],type="b",col="gold2",pch=15,lwd=3,cex=1.5)

points(wangenv[wangenv$day==260,"dist"],wangenv[wangenv$day==260,"cfpom"],type="b",col="blue",pch=8,cex=1.5,lwd=3,lty=3)

points(wangenv[wangenv$day==336,"dist"],wangenv[wangenv$day==336,"cfpom"],type="b",col="green3",pch=17,cex=1.5,lwd=3)

points(wangenv[wangenv$day==518,"dist"],wangenv[wangenv$day==518,"cfpom"],type="b",col="red",pch=18,cex=2,lwd=3,lty=5)

legend("topright",inset=c(0,0),legend=c("Dec 13", "April 14", "Aug 2014", "Nov 2014","May 2015"),lty=c(1,5),pch=c(19,15,8,17,18),lwd=2,col=c("black","gold2","blue","green3","red"),ncol=2,horiz=FALSE,cex=0.9,title="months")

abline(v=4,lty=2)

dev.off()

#############

# Ordered community table

vegemite(wangbug,1:40,scale="log") # Functions vegemite displays compact community tables

### PCO (Principal coordinate analysis) #######

wangbug.BC<-vegdist(sqrt(wangbug)) #Bray-Curtis dissimilarity matrix of square-root transformed abundances

n<-dim(wangbug)[1]

p<-n-1

#cmdscale is multidimensional scaling, also known as principal coordinates analysis (Gower, 1966)

wangbug.mds<-cmdscale(wangbug.BC, k = p, eig = TRUE, add = TRUE, x.ret = FALSE)# PCoA using the Bray-Curtis dissimilarity measure on square-root transformed abundances, and a correction for negative eigenvalues

# add=TRUE, Logical indicating if an additive constant should be computed, and added to the non-diagonal dissimilarities such that all eigenvalues are non-negative in the underlying Principal Co-ordinates Analysis (see cmdscale for details)

pco.varpercent<-round(wangbug.mds$eig/sum(wangbug.mds$eig)*100,digits=1) # Percentage of variation explained by each successive PCO axis

round(cumsum(wangbug.mds$eig)/sum(wangbug.mds$eig)*100,digits=2)

edit(wangbug.mds$points)

pwangbug<-wangbug.mds$points

dim(pwangbug)

colnames(pwangbug)<-c(paste("pco",sep="",1:p))

edit(pwangbug)

## Diagnostics for PCO

wangeigens<-wangbug.mds$eig # eigenvalues

D=as.matrix(vegdist(sqrt(wangbug),diag=TRUE,upper=TRUE)) # symmetric BC dissimilarity matrix

n=dim(D)[1] # sample size

p=n-1 # no of PCO axes

nmax=20 #max no. of axes to plot

Y=sqrt(wangbug)

## Percentage of variation explained by each successive PCO axis:

round ( 100*wangeigens/sum(wangeigens) , digits=2)

###

## Scree plot with expectations for eigenvalues under the broken stick model

plot(1:n, 100*wangeigens/sum(wangeigens), type="b", xlab="PCO axis number",

ylab="Percent variation explained", pch=19, las=1,

main="a) Broken stick model", xlim=c(0,nmax))

abline(h=0)

broken.stick=rep(0,n)

for (k in 1:p) broken.stick[k] = sum(1/(k:p))

broken.stick.perc = 100*broken.stick/sum(broken.stick)

points(1:n,broken.stick.perc, type="b", lty="dotted", cex=1.3,

xlim=c(0,nmax)) # Figure 3a

## Bootstrap eigenvalue method (e.g., see Jackson 1993 ).

nboot = 999

wangeigens.boot=matrix(rep(0,nboot*n),nrow=nboot, ncol=n, byrow=T)

wangeigens.perc.boot=matrix(rep(0,nboot*n),nrow=nboot, ncol=n, byrow=T)

for (iboot in 1:nboot) {

index = sample(1:n, replace=TRUE)

D.boot = D[index,index]

# Do the PCO and get eigenvalues from the bootstrap data

wangeigens.boot[iboot,1:n]= cmdscale(D.boot, k = p, eig = TRUE, add = TRUE, x.ret = FALSE)$eig

wangeigens.perc.boot[iboot,1:n] = 100*wangeigens.boot[iboot,1:n]/sum(wangeigens.boot[iboot,1:n])

}

# Get the empirical 95% confidence interval on the bootstrap eigenvalues

low <- function(x) quantile(x,probs=0.025)

high <- function(x) quantile(x,probs=0.975)

lower = apply(wangeigens.perc.boot, MARGIN = 2, low)

upper = apply(wangeigens.perc.boot, MARGIN = 2, high)

centre = apply(wangeigens.perc.boot, MARGIN = 2, mean)

# Scree plot with the bootstrap results

plot(1:n, centre, type="b", xlab="PCO axis number",

ylab="Percent variation explained", las=1, pch=19, ylim = c(0,30),

main="b) Bootstrap eigenvector method", xlim=c(0,nmax)) #

abline(0,0)

arrows(1:n,lower,1:n,upper, code=3, length=0.1, angle = 90, xlim=c(0,nmax)) # Figure 3b

## Permutation method (e.g., McCune et al. 2002; Clarke et al. 2008) with variation explained by each axis as either:

## 1. a fraction of the total (holistically), or

## 2. a fraction of the variation remaining given prior axes (conditionally)

nperm = 999

wangeigens.perm=matrix(rep(0,nperm*n),nrow=nperm, ncol=n, byrow=T)

wangeigens.perc.perm1=matrix(rep(0,nperm*n),nrow=nperm, ncol=n, byrow=T)

wangeigens.perc.perm2=matrix(rep(0,nperm*n),nrow=nperm, ncol=n, byrow=T)

nvars = dim(Y)[2] # no. of variables in original Y matrix

for (iperm in 1:nperm) {

Y.perm = matrix(rep(0,n*nvars), nrow=n, ncol=nvars, byrow=T)

for (k in 1:nvars) {

index = sample(1:n, replace=FALSE)

Y.perm[1:n,k] = Y[index,k]

}

D.perm = as.matrix(vegdist(Y.perm,diag=TRUE,upper=FALSE))

# Do the PCO and get eigenvalues from the permuted data

wangeigens.perm[iperm,1:n]= cmdscale(D.perm, k = p, eig = TRUE, add = TRUE,

x.ret = FALSE)$eig

wangeigens.perc.perm1[iperm,1:n] =

100*wangeigens.perm[iperm,1:n]/sum(wangeigens.perm[iperm,1:n])

for (k in 1:n) {

wangeigens.perc.perm2[iperm,k] =

100*wangeigens.perm[iperm,k]/sum(wangeigens.perm[iperm,k:n])

}

}

# Get the empirical 95% confidence interval on the permutation eigenvalues, in

# each case

lower.p1 = apply(wangeigens.perc.perm1, MARGIN = 2, low)

upper.p1 = apply(wangeigens.perc.perm1, MARGIN = 2, high)

middle.p1 = apply(wangeigens.perc.perm1, MARGIN = 2, median)

lower.p2 = apply(wangeigens.perc.perm2, MARGIN = 2, low)

upper.p2 = apply(wangeigens.perc.perm2, MARGIN = 2, high)

middle.p2 = apply(wangeigens.perc.perm2, MARGIN = 2, median)

# Scree plot with the permutation results, holistic approach

plot(1:n, 100*wangeigens/sum(wangeigens), type="b", xlab="PCO axis number",

ylab="Percent variation explained", las=1, pch=19,

ylim = c(0,max(110*wangeigens/sum(wangeigens))),xlim=c(0,nmax),

main="c) Permutation method - holistic approach")

abline(0,0)

points(1:n, middle.p1, type="b", lty="dotted", cex=1.3,xlim=c(0,nmax))

arrows(1:n,lower.p1,1:n,upper.p1, code=3, length=0.1, angle = 90) # # Figure 3c

# Scree plot with the permutation results, conditional approach

real=rep(0,n)

for (k in 1:n) {real[k] = 100*wangeigens[k]/sum(wangeigens[k:n])}

plot(1:(n-2), real[1:(n-2)], type="b", xlab="PCO axis number",

ylab="Percent variation explained", las=1, pch=19,

ylim = c(0,max(120*wangeigens/sum(wangeigens))), xlim=c(0,nmax),

main="d) Permutation method - conditional approach")

abline(0,0)

points(1:(n-2), middle.p2[1:(n-2)], type="b", lty="dotted", cex=1.3)

arrows(1:(n-2),lower.p2[1:(n-2)],1:(n-2),upper.p2[1:(n-2)], code=3, length=0.1,

angle = 90) # Figure 3d

dev.off()

#################################################

#***** Figure 3*****#

tiff(file="Figure 3.jpg",width=6,height=6,units="in",pointsize = 12,bg = "transparent",res=800,compression="lzw") # Save the following graph as a tif image

par(mfrow=c(2,2),mar=c(4,4,2.5,2)+0.1,cex=0.6,cex.axis=0.8,las=1)

# Scree plot with expectations for eigenvalues under the broken stick model

plot(1:n, 100*wangeigens/sum(wangeigens), type="b", xlab="PCO axis number",

ylab="Percent variation explained", pch=19, las=1, xlim=c(0,nmax))

abline(h=0)

broken.stick=rep(0,n)

for (k in 1:p) broken.stick[k] = sum(1/(k:p))

broken.stick.perc = 100*broken.stick/sum(broken.stick)

points(1:n,broken.stick.perc, type="b", lty="dotted", cex=1.3,

xlim=c(0,nmax)) # Figure 3a

mtext("a",3,-5,cex=1.8)

# Scree plot with the bootstrap results

plot(1:n, centre, type="b", xlab="PCO axis number",

ylab="Percent variation explained", las=1, pch=19, ylim = c(0,max(upper)),

xlim=c(0,nmax))

abline(0,0)

arrows(1:n,lower,1:n,upper, code=3, length=0.1, angle = 90,

xlim=c(0,nmax)) # Figure 3b

mtext("b",3,-5,cex=1.8)

# Scree plot with the permutation results, holistic approach

plot(1:n, 100*wangeigens/sum(wangeigens), type="b", xlab="PCO axis number",

ylab="Percent variation explained", las=1, pch=19,

ylim = c(0,max(110*wangeigens/sum(wangeigens))),xlim=c(0,nmax))

abline(0,0)

points(1:n, middle.p1, type="b", lty="dotted", cex=1.3,xlim=c(0,nmax))

arrows(1:n,lower.p1,1:n,upper.p1, code=3, length=0.1, angle = 90) # Figure 3c

mtext("c",3,-5,cex=1.8)

# Scree plot with the permutation results, conditional approach

real=rep(0,n)

for (k in 1:n) {real[k] = 100*wangeigens[k]/sum(wangeigens[k:n])}

plot(1:(n-2), real[1:(n-2)], type="b", xlab="PCO axis number",

ylab="Percent variation explained", las=1, pch=19,

ylim = c(0,max(110*wangeigens/sum(wangeigens))), xlim=c(0,nmax))

abline(0,0)

points(1:(n-2), middle.p2[1:(n-2)], type="b", lty="dotted", cex=1.3)

arrows(1:(n-2),lower.p2[1:(n-2)],1:(n-2),upper.p2[1:(n-2)], code=3, length=0.1,

angle = 90) # Figure 3d

mtext("d",3,-5,cex=1.8)

dev.off()

####PCO1 plotted against spatial position #

#***** Figure 1 - Appendix A *****#

tiff(file="Figure 1 - Appendix A.tif",width=14,height=14,units="in",pointsize = 12,bg ="transparent",res=800,compression="lzw")

par(mfrow=c(3,2), mar=c(0.1,0.1,0.1,0.1),cex=0.6,cex.axis=0.8,las=1)

##PCO1 against distance

par(mar=c(5,5,4,2),cex=0.9)

plot(bugenv[bugenv$day==1,"dist"],bugenv[bugenv$day==1,"pco1"],type = "b",ylim=c(-.6,1),pch=19,cex=1.5,xlab="Distance(km)",ylab="PCO1 score",main="PCO1 plotted against spatial position",lwd=3,lty=1)

points(bugenv[bugenv$day==126,"dist"],bugenv[bugenv$day==126,"pco1"],type="b",col="gold2",pch=15,lwd=3,cex=1.5,lty=2)

points(bugenv[bugenv$day==260,"dist"],bugenv[bugenv$day==260,"pco1"],type="b",col="blue",pch=8,cex=1.5,lwd=3,lty=3)

points(bugenv[bugenv$day==336,"dist"],bugenv[bugenv$day==336,"pco1"],type="b",col="green3",pch=17,cex=1.5,lwd=3,lty=4)

points(bugenv[bugenv$day==518,"dist"],bugenv[bugenv$day==518,"pco1"],type="b",col="red",pch=18,cex=2,lwd=3,lty=5)

legend("topright",inset=c(0,0),legend=c("Dec 13", "April 14", "Aug 2014", "Nov 2014","May 2015"),lty=c(1,5),pch=c(19,15,8,17,18),lwd=2,col=c("black","gold2","blue","green3","red"),ncol=2,horiz=FALSE,cex=0.9,title="months")

abline(v=4,lty=2)

##PCO2 against distance

par(mar=c(5,5,4,2),cex=0.9)

plot(bugenv[bugenv$day==1,"dist"],bugenv[bugenv$day==1,"pco2"],type = "b",ylim=c(-.6,1),pch=19,cex=1.5,xlab="Distance(km)",ylab="PCO2",main="b) PCO2 plotted against spatial position",lwd=3,lty=1)

points(bugenv[bugenv$day==126,"dist"],bugenv[bugenv$day==126,"pco2"],type="b",col="gold2",pch=15,lwd=3,cex=1.5,lty=2)

points(bugenv[bugenv$day==260,"dist"],bugenv[bugenv$day==260,"pco2"],type="b",col="blue",pch=8,cex=1.5,lwd=3,lty=3)

points(bugenv[bugenv$day==336,"dist"],bugenv[bugenv$day==336,"pco2"],type="b",col="green3",pch=17,cex=1.5,lwd=3,lty=4)

points(bugenv[bugenv$day==518,"dist"],bugenv[bugenv$day==518,"pco2"],type="b",col="red",pch=18,cex=2,lwd=3,lty=5)

legend("topright",inset=c(0,0),legend=c("Dec 13", "April 14", "Aug 2014", "Nov 2014","May 2015"),lty=c(1,5),pch=c(19,15,8,17,18),lwd=2,col=c("black","gold2","blue","green3","red"),ncol=2,horiz=FALSE,cex=0.9,title="months")

abline(v=4,lty=2)

##PCO3 against distance

par(mar=c(5,5,4,2),cex=0.9)

plot(bugenv[bugenv$day==1,"dist"],bugenv[bugenv$day==1,"pco3"],type = "b",ylim=c(-.6,1),pch=19,cex=1.5,xlab="Distance(km)",ylab="PCO3",main="c) PCO3 plotted against spatial position",lwd=3,lty=1)

points(bugenv[bugenv$day==126,"dist"],bugenv[bugenv$day==126,"pco3"],type="b",col="gold2",pch=15,lwd=3,cex=1.5,lty=2)

points(bugenv[bugenv$day==260,"dist"],bugenv[bugenv$day==260,"pco3"],type="b",col="blue",pch=8,cex=1.5,lwd=3,lty=3)

points(bugenv[bugenv$day==336,"dist"],bugenv[bugenv$day==336,"pco3"],type="b",col="green3",pch=17,cex=1.5,lwd=3,lty=4)

points(bugenv[bugenv$day==518,"dist"],bugenv[bugenv$day==518,"pco3"],type="b",col="red",pch=18,cex=2,lwd=3,lty=5)

legend("topright",inset=c(0,0),legend=c("Dec 13", "April 14", "Aug 2014", "Nov 2014","May 2015"),lty=c(1,5),pch=c(19,15,8,17,18),lwd=2,col=c("black","gold2","blue","green3","red"),ncol=2,horiz=FALSE,cex=0.9,title="months")

abline(v=4,lty=2)

##PCO4 against distance

par(mar=c(5,5,4,2),cex=0.9)

plot(bugenv[bugenv$day==1,"dist"],bugenv[bugenv$day==1,"pco4"],type = "b",ylim=c(-.6,1),pch=19,cex=1.5,xlab="Distance(km)",ylab="PCO4",main="d) PCO4 plotted against spatial position",lwd=3,lty=1)

points(bugenv[bugenv$day==126,"dist"],bugenv[bugenv$day==126,"pco4"],type="b",col="gold2",pch=15,lwd=3,cex=1.5,lty=2)

points(bugenv[bugenv$day==260,"dist"],bugenv[bugenv$day==260,"pco4"],type="b",col="blue",pch=8,cex=1.5,lwd=3,lty=3)

points(bugenv[bugenv$day==336,"dist"],bugenv[bugenv$day==336,"pco4"],type="b",col="green3",pch=17,cex=1.5,lwd=3,lty=4)

points(bugenv[bugenv$day==518,"dist"],bugenv[bugenv$day==518,"pco4"],type="b",col="red",pch=18,cex=2,lwd=3,lty=5)

legend("topright",inset=c(0,0),legend=c("Dec 13", "April 14", "Aug 2014", "Nov 2014","May 2015"),lty=c(1,5),pch=c(19,15,8,17,18),lwd=2,col=c("black","gold2","blue","green3","red"),ncol=2,horiz=FALSE,cex=0.9,title="months")

abline(v=4,lty=2)

##PCO5 against distance

par(mar=c(5,5,4,2),cex=0.9)

plot(bugenv[bugenv$day==1,"dist"],bugenv[bugenv$day==1,"pco5"],type = "b",ylim=c(-.6,1),pch=19,cex=1.5,xlab="Distance(km)",ylab="PCO5",main="e) PCO5 plotted against spatial position",lwd=3,lty=1)

points(bugenv[bugenv$day==126,"dist"],bugenv[bugenv$day==126,"pco5"],type="b",col="gold2",pch=15,lwd=3,cex=1.5,lty=2)

points(bugenv[bugenv$day==260,"dist"],bugenv[bugenv$day==260,"pco5"],type="b",col="blue",pch=8,cex=1.5,lwd=3,lty=3)

points(bugenv[bugenv$day==336,"dist"],bugenv[bugenv$day==336,"pco5"],type="b",col="green3",pch=17,cex=1.5,lwd=3,lty=4)

points(bugenv[bugenv$day==518,"dist"],bugenv[bugenv$day==518,"pco5"],type="b",col="red",pch=18,cex=2,lwd=3,lty=5)

legend("topright",inset=c(0,0),legend=c("Dec 13", "April 14", "Aug 2014", "Nov 2014","May 2015"),lty=c(1,5),pch=c(19,15,8,17,18),lwd=2,col=c("black","gold2","blue","green3","red"),ncol=2,horiz=FALSE,cex=0.9,title="months")

abline(v=4,lty=2)

##PCO6 against distance

par(mar=c(5,5,4,2),cex=0.9)

plot(bugenv[bugenv$day==1,"dist"],bugenv[bugenv$day==1,"pco6"],type = "b",ylim=c(-.6,1),pch=19,cex=1.5,xlab="Distance(km)",ylab="PCO6",main="f) PCO6 plotted against spatial position",lwd=3,lty=1)

points(bugenv[bugenv$day==126,"dist"],bugenv[bugenv$day==126,"pco6"],type="b",col="gold2",pch=15,lwd=3,cex=1.5,lty=2)

points(bugenv[bugenv$day==260,"dist"],bugenv[bugenv$day==260,"pco6"],type="b",col="blue",pch=8,cex=1.5,lwd=3,lty=3)

points(bugenv[bugenv$day==336,"dist"],bugenv[bugenv$day==336,"pco6"],type="b",col="green3",pch=17,cex=1.5,lwd=3,lty=4)

points(bugenv[bugenv$day==518,"dist"],bugenv[bugenv$day==518,"pco6"],type="b",col="red",pch=18,cex=2,lwd=3,lty=5)

legend("topright",inset=c(0,0),legend=c("Dec 13", "April 14", "Aug 2014", "Nov 2014","May 2015"),lty=c(1,5),pch=c(19,15,8,17,18),lwd=2,col=c("black","gold2","blue","green3","red"),ncol=2,horiz=FALSE,cex=0.9,title="months")

abline(v=4,lty=2)

dev.off()

################ displaying date labels on x-axis for Figure 2 - Appendix A

edit(bugenv)

as.character(bugenv$date)

bugenv$date<-as.Date(as.character(bugenv$date), format="%d-%m-%Y")

mydates<-bugenv[bugenv$dist==0,"date"]

mydaterange=c(as.POSIXlt(min(mydates)),as.POSIXlt(max(mydates)))

########PCOs plotted against time  ##***** Figure 2 - Appendix A *****#

##PCO1 against time

tiff(file="Figure 2 - Appendix A.jpg",width=14,height=14,units="in",pointsize = 12,bg ="transparent",res=800,compression="lzw")

par(mfrow=c(3,2), mar=c(2,4.5,2,0.5),cex=1.5,cex.axis=0.7,las=1,cex.main=0.8)

#par(mar=c(5,5,4,2),cex=0.9)

plot(mydates,bugenv[bugenv$dist==0,"pco1"],type = "b",ylim=c(-.8,.8),pch=19,cex=1,xlab="day",ylab="PCO1",main="a) PCO1 plotted against time",lwd=3,lty=2,cex.main=1,xaxt="n")

points(mydates,bugenv[bugenv$dist==1.8,"pco1"],type="b",col="deep pink",pch=15,lwd=3,cex=1,lty=3)

points(mydates,bugenv[bugenv$dist==3.2,"pco1"],type="b",col="blue",pch=8,cex=1,lwd=3,lty=4)

points(mydates,bugenv[bugenv$dist==3.9,"pco1"],type="b",col="green",pch=17,cex=1,lwd=3,lty=5)

points(mydates,bugenv[bugenv$dist==4.08,"pco1"],type="b",col="red",pch=18,cex=1.5,lwd=3,lty=6)

points(mydates,bugenv[bugenv$dist==4.83,"pco1"],type="b",col="gold2",pch=18,cex=1.52,lwd=3,lty=7)

points(mydates,bugenv[bugenv$dist==6.53,"pco1"],type="b",col="lightseagreen",pch=18,cex=1.5,lwd=3,lty=6)

points(mydates,bugenv[bugenv$dist==7.08,"pco1"],type="b",col="sienna2",pch=18,cex=1.5,lwd=3,lty=6)

labels<-axis.Date(side=1,mydates,at=seq(mydaterange[1],mydaterange[2],by="month"),format="%b-%y")

legend("bottomright",inset=c(0,0),legend=levels(as.factor(bugenv$dist)),lty=1,lwd=2,col=c("black","deep pink","blue","green","red","gold2","lightseagreen","sienna2"),ncol=4,horiz=FALSE,cex=0.6,title="distance(km)")

##PCO2 against time

#tiff(file="pco2-time.tif",width=6,height=3.5,units="in",pointsize = 12,bg = "transparent",res=800,compression="lzw") # Save the following graph as a tif image

#par(mar=c(4,4,2.5,2)+0.1,cex=0.6,cex.axis=0.8,las=1)

plot(mydates,bugenv[bugenv$dist==0,"pco2"],type = "b",ylim=c(-.8,.8),pch=19,cex=1,xlab="day",ylab="PCO2",main="b) PCO2 plotted against time",lwd=3,lty=2,cex.main=1,xaxt="n")

points(mydates,bugenv[bugenv$dist==1.8,"pco2"],type="b",col="deep pink",pch=15,lwd=3,cex=1,lty=3)

points(mydates,bugenv[bugenv$dist==3.2,"pco2"],type="b",col="blue",pch=8,cex=1,lwd=3,lty=4)

points(mydates,bugenv[bugenv$dist==3.9,"pco2"],type="b",col="green",pch=17,cex=1,lwd=3,lty=5)

points(mydates,bugenv[bugenv$dist==4.08,"pco2"],type="b",col="red",pch=18,cex=1.5,lwd=3,lty=6)

points(mydates,bugenv[bugenv$dist==4.83,"pco2"],type="b",col="gold2",pch=18,cex=1.5,lwd=3,lty=7)

points(mydates,bugenv[bugenv$dist==6.53,"pco2"],type="b",col="lightseagreen",pch=18,cex=1.5,lwd=3,lty=6)

points(mydates,bugenv[bugenv$dist==7.08,"pco2"],type="b",col="sienna2",pch=18,cex=1.5,lwd=3,lty=6)

labels<-axis.Date(side=1,mydates,at=seq(mydaterange[1],mydaterange[2],by="month"),format="%b-%y")

legend("bottomright",inset=c(0,0),legend=levels(as.factor(bugenv$dist)),lty=1,lwd=2,col=c("black","deep pink","blue","green","red","gold2","lightseagreen","sienna2"),ncol=4,horiz=FALSE,cex=0.6,title="distance(km)")

##PCO3 against time

#tiff(file="pco3-time.tif",width=6,height=3.5,units="in",pointsize = 12,bg = "transparent",res=800,compression="lzw") # Save the following graph as a tif image

plot(mydates,bugenv[bugenv$dist==0,"pco3"],type = "b",ylim=c(-.8,.8),pch=19,cex=1,xlab="day",ylab="PCO3",main="c) PCO3 plotted against time",lwd=3,lty=2,cex.main=1,xaxt="n")

points(mydates,bugenv[bugenv$dist==1.8,"pco3"],type="b",col="deep pink",pch=15,lwd=3,cex=1,lty=3)

points(mydates,bugenv[bugenv$dist==3.2,"pco3"],type="b",col="blue",pch=8,cex=1,lwd=3,lty=4)

points(mydates,bugenv[bugenv$dist==3.9,"pco3"],type="b",col="green",pch=17,cex=1,lwd=3,lty=5)

points(mydates,bugenv[bugenv$dist==4.08,"pco3"],type="b",col="red",pch=18,cex=1.5,lwd=3,lty=6)

points(mydates,bugenv[bugenv$dist==4.83,"pco3"],type="b",col="gold2",pch=18,cex=1.5,lwd=3,lty=7)

points(mydates,bugenv[bugenv$dist==6.53,"pco3"],type="b",col="lightseagreen",pch=18,cex=1.5,lwd=3,lty=6)

points(mydates,bugenv[bugenv$dist==7.08,"pco3"],type="b",col="sienna2",pch=18,cex=1.5,lwd=3,lty=6)

labels<-axis.Date(side=1,mydates,at=seq(mydaterange[1],mydaterange[2],by="month"),format="%b-%y")

legend("bottomright",inset=c(0,0),legend=levels(as.factor(bugenv$dist)),lty=1,lwd=2,col=c("black","deep pink","blue","green","red","gold2","lightseagreen","sienna2"),ncol=4,horiz=FALSE,cex=0.6,title="distance(km)")

#dev.off()

##PCO4 against time

#tiff(file="pco4-time.tif",width=6,height=3.5,units="in",pointsize = 12,bg = "transparent",res=800,compression="lzw") # Save the following graph as a tif image

plot(mydates,bugenv[bugenv$dist==0,"pco4"],type = "b",ylim=c(-.8,.8),pch=19,cex=1,xlab="day",ylab="PCO4",main="d) PCO4 plotted against time",lwd=3,lty=2,cex.main=1,xaxt="n")

points(mydates,bugenv[bugenv$dist==1.8,"pco4"],type="b",col="deep pink",pch=15,lwd=3,cex=1,lty=3)

points(mydates,bugenv[bugenv$dist==3.2,"pco4"],type="b",col="blue",pch=8,cex=1,lwd=3,lty=4)

points(mydates,bugenv[bugenv$dist==3.9,"pco4"],type="b",col="green",pch=17,cex=1,lwd=3,lty=5)

points(mydates,bugenv[bugenv$dist==4.08,"pco4"],type="b",col="red",pch=18,cex=1.5,lwd=3,lty=6)

points(mydates,bugenv[bugenv$dist==4.83,"pco4"],type="b",col="gold2",pch=18,cex=1.5,lwd=3,lty=7)

points(mydates,bugenv[bugenv$dist==6.53,"pco4"],type="b",col="lightseagreen",pch=18,cex=1.5,lwd=3,lty=6)

points(mydates,bugenv[bugenv$dist==7.08,"pco4"],type="b",col="sienna2",pch=18,cex=1.5,lwd=3,lty=6)

labels<-axis.Date(side=1,mydates,at=seq(mydaterange[1],mydaterange[2],by="month"),format="%b-%y")

legend("bottomright",inset=c(0,0),legend=levels(as.factor(bugenv$dist)),lty=1,lwd=2,col=c("black","deep pink","blue","green","red","gold2","lightseagreen","sienna2"),ncol=4,horiz=FALSE,cex=0.6,title="distance(km)")

#dev.off()

##PCO5 against time

#tiff(file="pco5-time.tif",width=6,height=3.5,units="in",pointsize = 12,bg = "transparent",res=800,compression="lzw") # Save the following graph as a tif image

plot(mydates,bugenv[bugenv$dist==0,"pco5"],type = "b",ylim=c(-.8,.8),pch=19,cex=1,xlab="day",ylab="PCO5",main="e) PCO5 plotted against time",lwd=3,lty=2,cex.main=1,xaxt="n")

points(mydates,bugenv[bugenv$dist==1.8,"pco5"],type="b",col="deep pink",pch=15,lwd=3,cex=1.5,lty=3)

points(mydates,bugenv[bugenv$dist==3.2,"pco5"],type="b",col="blue",pch=8,cex=1,lwd=3,lty=4)

points(mydates,bugenv[bugenv$dist==3.9,"pco5"],type="b",col="green",pch=17,cex=1,lwd=3,lty=5)

points(mydates,bugenv[bugenv$dist==4.08,"pco5"],type="b",col="red",pch=18,cex=1.5,lwd=3,lty=6)

points(mydates,bugenv[bugenv$dist==4.83,"pco5"],type="b",col="gold2",pch=18,cex=1.5,lwd=3,lty=7)

points(mydates,bugenv[bugenv$dist==6.53,"pco5"],type="b",col="lightseagreen",pch=18,cex=1.5,lwd=3,lty=6)

points(mydates,bugenv[bugenv$dist==7.08,"pco5"],type="b",col="sienna2",pch=18,cex=1.5,lwd=3,lty=6)

labels<-axis.Date(side=1,mydates,at=seq(mydaterange[1],mydaterange[2],by="month"),format="%b-%y")

legend("bottomright",inset=c(0,0),legend=levels(as.factor(bugenv$dist)),lty=1,lwd=2,col=c("black","deep pink","blue","green","red","gold2","lightseagreen","sienna2"),ncol=4,horiz=FALSE,cex=0.6,title="distance(km)")

#dev.off()

##PCO6 against time

#tiff(file="pco6-time.tif",width=6,height=3.5,units="in",pointsize = 12,bg = "transparent",res=800,compression="lzw") # Save the following graph as a tif image

#par(mar=c(4,4,2.5,2)+0.1,cex=0.6,cex.axis=0.8,las=1)

plot(mydates,bugenv[bugenv$dist==0,"pco6"],type = "b",ylim=c(-.8,.8),pch=19,cex=1,xlab="day",ylab="PCO6",main="f) PCO6 plotted against time",lwd=3,lty=2,cex.main=1,xaxt="n")

points(mydates,bugenv[bugenv$dist==1.8,"pco6"],type="b",col="deep pink",pch=15,lwd=3,cex=1,lty=3)

points(mydates,bugenv[bugenv$dist==3.2,"pco6"],type="b",col="blue",pch=8,cex=1,lwd=3,lty=4)

points(mydates,bugenv[bugenv$dist==3.9,"pco6"],type="b",col="green",pch=17,cex=1,lwd=3,lty=5)

points(mydates,bugenv[bugenv$dist==4.08,"pco6"],type="b",col="red",pch=18,cex=1.5,lwd=3,lty=6)

points(mydates,bugenv[bugenv$dist==4.83,"pco6"],type="b",col="gold2",pch=18,cex=1.5,lwd=3,lty=7)

points(mydates,bugenv[bugenv$dist==6.53,"pco6"],type="b",col="lightseagreen",pch=18,cex=1.5,lwd=3,lty=6)

points(mydates,bugenv[bugenv$dist==7.08,"pco6"],type="b",col="sienna2",pch=18,cex=1.5,lwd=3,lty=6)

labels<-axis.Date(side=1,mydates,at=seq(mydaterange[1],mydaterange[2],by="month"),format="%b-%y")

legend("bottomright",inset=c(0,0),legend=levels(as.factor(bugenv$dist)),lty=1,lwd=2,col=c("black","deep pink","blue","green","red","gold2","lightseagreen","sienna2"),ncol=4,horiz=FALSE,cex=0.6,title="distance(km)")

dev.off()

##PCO7 against time

tiff(file="pco7-time.tif",width=6,height=3.5,units="in",pointsize = 12,bg = "transparent",res=800,compression="lzw") # Save the following graph as a tif image

par(mar=c(4,4,2.5,2)+0.1,cex=0.6,cex.axis=0.8,las=1)

plot(mydates,bugenv[bugenv$dist==0,"pco7"],type = "b",ylim=c(-.7,.7),pch=19,cex=1.5,xlab="day",ylab="PCO7",main="PCO7 plotted against time",lwd=3,lty=2,xaxt="n")

points(mydates,bugenv[bugenv$dist==1.8,"pco7"],type="b",col="deep pink",pch=15,lwd=3,cex=1.5,lty=3)

points(mydates,bugenv[bugenv$dist==3.2,"pco7"],type="b",col="blue",pch=8,cex=1.5,lwd=3,lty=4)

points(mydates,bugenv[bugenv$dist==3.9,"pco7"],type="b",col="green",pch=17,cex=1.5,lwd=3,lty=5)

points(mydates,bugenv[bugenv$dist==4.08,"pco7"],type="b",col="red",pch=18,cex=2,lwd=3,lty=6)

points(mydates,bugenv[bugenv$dist==4.83,"pco7"],type="b",col="gold2",pch=18,cex=2,lwd=3,lty=7)

points(mydates,bugenv[bugenv$dist==6.53,"pco7"],type="b",col="lightseagreen",pch=18,cex=2,lwd=3,lty=6)

points(mydates,bugenv[bugenv$dist==7.08,"pco7"],type="b",col="sienna2",pch=18,cex=2,lwd=3,lty=6)

labels<-axis.Date(side=1,mydates,at=seq(mydaterange[1],mydaterange[2],by="month"),format="%b-%y")

legend("bottomright",inset=c(0,0),legend=levels(as.factor(bugenv$dist)),lty=1,lwd=2,col=c("black","deep pink","blue","green","red","gold2","lightseagreen","sienna2"),ncol=4,horiz=FALSE,cex=0.8,title="distance(km)")

dev.off()

## Further exploratory analysis #combine environmental data and benthos data

edit(wangenv)

bugenv<-cbind(wangenv,pwangbug)# combine environmental data and PCO matrix

edit(bugenv)

dim(bugenv)

edit(pwangbug)

################ displaying date labels on x-axis for Rain and flow rate figures

edit(bugenv)

as.character(bugenv$date)

bugenv$date<-as.Date(as.character(bugenv$date), format="%d-%m-%Y")

mydates<-bugenv[bugenv$dist==0,"date"]

mydaterange=c(as.POSIXlt(min(mydates)),as.POSIXlt(max(mydates)))

#***** Rain and flow rate figure*****#

#####rainfall, discharge and creek flow rate graphs

tiff(file="rain and flow rate.tif",width=12,height=14,units="in",pointsize = 12,bg ="transparent",res=800,compression="lzw")

par(mfrow=c(3,1), mar=c(5,5,4,2),cex=1.2,cex.axis=0.8,las=1)

plot(mydates,bugenv[bugenv$dist==0,"dayflow"],type = "p",ylim=c(0,200),pch=19,cex=1.5,xlab="day",ylab="Creek flow rate (ML/day)",main="Average daily Creek flow rate plotted against time",lwd=3,lty=2,xaxt="n")

axis.Date(side=1,mydates,at=seq(mydaterange[1],mydaterange[2],by="month"),format="%b-%y")

plot(mydates,wangenv[wangenv$dist==0,"dflow"],type = "p",ylim=c(0,10),pch=19,cex=1.5,xlab="day",ylab="Discharge flow rate (ML/day)",main="Daily effluent discharge flow rate plotted against time",lwd=3,lty=2,xaxt="n",col="blue")

axis.Date(side=1,mydates,at=seq(mydaterange[1],mydaterange[2],by="month"),format="%b-%y")

plot(mydates,bugenv[bugenv$dist==0,"rain3"],type = "p",ylim=c(0,10),pch=19,cex=1.5,xlab="day",ylab="Rainfall(mm)",main="Three-month average rainfall plotted against time",lwd=3,lty=2,xaxt="n",col="red")

axis.Date(side=1,mydates,at=seq(mydaterange[1],mydaterange[2],by="month"),format="%b-%y")

dev.off()

tiff(file="rainfall (a month).tif",width=6,height=3.5,units="in",pointsize = 12,bg = "transparent",res=800,compression="lzw")

par(mar=c(5,5,4,2),cex=0.9)

plot(mydates,bugenv[bugenv$dist==0,"rain2"],type = "p",ylim=c(0,10),pch=19,cex=1.5,xlab="day",ylab="Rainfall(mm)",main="Average rainfall (a month) plotted against time",lwd=3,lty=2,xaxt="n",col="blue")

axis.Date(side=1,mydates,at=seq(mydaterange[1],mydaterange[2],by="month"),format="%b-%y")

dev.off()

#***** prediction with Temperature and conductivity*****#

tiff(file="prediction with Temperature and conductivity.jpg",width=14,height=14,units="in",pointsize = 12,bg ="transparent",res=800,compression="lzw")

par(mfrow=c(3,2), mar=c(2,4.5,2,0.5),cex=1.5,cex.axis=0.9,las=1,cex.main=1.5)

####prediction with Temperature

tempo<-lm(pco1~temp,data=bugenv)

summary(tempo)

fitted(tempo)

tempo1<-cbind(cbind(fitted(tempo),residuals(tempo)),bugenv)#

attributes(tempo1)

names(tempo1[,1:2])<-c("fitted","residual")

par(mar=c(5,5,4,2),cex=0.9)

plot(tempo1[tempo1$day==1,"dist"],tempo1[tempo1$day==1,"1"],type = "b",ylim=c(-.6,0.6),pch=19,cex=1.5,xlab="Distance(km)",ylab="PCO1",main="a) Prediction with temperature",lwd=3,lty=1)

points(tempo1[tempo1$day==126,"dist"],tempo1[tempo1$day==126,"1"],type="b",col="gold2",pch=15,lwd=3,cex=1.5,lty=2)

points(tempo1[tempo1$day==260,"dist"],tempo1[tempo1$day==260,"1"],type="b",col="blue",pch=8,cex=1.5,lwd=3,lty=3)

points(tempo1[tempo1$day==336,"dist"],tempo1[tempo1$day==336,"1"],type="b",col="green3",pch=17,cex=1.5,lwd=3,lty=4)

points(tempo1[tempo1$day==518,"dist"],tempo1[tempo1$day==518,"1"],type="b",col="red",pch=18,cex=2,lwd=3,lty=5)

legend("topright",inset=c(0,0),legend=c("Dec 13", "April 14", "Aug 2014", "Nov 2014","May 2015"),lty=c(1,5),pch=c(19,15,8,17,18),lwd=2,col=c("black","gold2","blue","green3","red"),ncol=2,horiz=FALSE,cex=0.8,title="months")

abline(v=4,lty=2)

####prediction with conductivity

tempo<-lm(pco1~cond,data=bugenv)

summary(tempo)

fitted(tempo)

tempo1<-cbind(cbind(fitted(tempo),residuals(tempo)),bugenv[complete.cases(bugenv[,c("temp","cond")]),])

attributes(tempo1)

names(tempo1[,1:2])<-c("fitted","residual")

par(mar=c(5,5,4,2),cex=0.9)

plot(tempo1[tempo1$day==126,"dist"],tempo1[tempo1$day==126,"1"],type = "b",ylim=c(-.6,0.6),pch=19,col="gold2",cex=1.5,xlab="Distance(km)",ylab="PCO1",main="b) Prediction with conductivity",lwd=3,lty=1)

#points(tempo1[tempo1$day==126,"dist"],tempo1[tempo1$day==126,"1"],type="b",col="gold2",pch=15,lwd=3,cex=1.5,lty=2)

points(tempo1[tempo1$day==260,"dist"],tempo1[tempo1$day==260,"1"],type="b",col="blue",pch=8,cex=1.5,lwd=3,lty=3)

points(tempo1[tempo1$day==336,"dist"],tempo1[tempo1$day==336,"1"],type="b",col="green3",pch=17,cex=1.5,lwd=3,lty=4)

points(tempo1[tempo1$day==518,"dist"],tempo1[tempo1$day==518,"1"],type="b",col="red",pch=18,cex=2,lwd=3,lty=5)

legend("topright",inset=c(0,0),legend=c("Dec 13", "April 14", "Aug 2014", "Nov 2014","May 2015"),lty=c(1,5),pch=c(19,15,8,17,18),lwd=2,col=c("black","gold2","blue","green3","red"),ncol=2,horiz=FALSE,cex=0.8,title="months")

abline(v=4,lty=2)

####prediction with Temperature and Conductivity

tempo<-lm(pco1~temp+cond,data=bugenv)

summary(tempo)

fitted(tempo)

tempo1<-cbind(cbind(fitted(tempo),residuals(tempo)),bugenv[complete.cases(bugenv[,c("temp","cond")]),])

attributes(tempo1)

names(tempo1[,1:2])<-c("fitted","residual")

par(mar=c(5,5,4,2),cex=0.9)

plot(tempo1[tempo1$day==126,"dist"],tempo1[tempo1$day==126,"1"],type = "b",ylim=c(-.6,0.6),pch=19,col="gold2",cex=1.5,xlab="Distance(km)",ylab="PCO1",main="c) Prediction with Temperature and Conductivity",lwd=3,lty=1)

#points(tempo1[tempo1$day==126,"dist"],tempo1[tempo1$day==126,"1"],type="b",col="gold2",pch=15,lwd=3,cex=1.5,lty=2)

points(tempo1[tempo1$day==260,"dist"],tempo1[tempo1$day==260,"1"],type="b",col="blue",pch=8,cex=1.5,lwd=3,lty=3)

points(tempo1[tempo1$day==336,"dist"],tempo1[tempo1$day==336,"1"],type="b",col="green3",pch=17,cex=1.5,lwd=3,lty=4)

points(tempo1[tempo1$day==518,"dist"],tempo1[tempo1$day==518,"1"],type="b",col="red",pch=18,cex=2,lwd=3,lty=5)

legend("topright",inset=c(0,0),legend=c("Dec 13", "April 14", "Aug 2014", "Nov 2014","May 2015"),lty=c(1,5),pch=c(19,15,8,17,18),lwd=2,col=c("black","gold2","blue","green3","red"),ncol=2,horiz=FALSE,cex=0.8,title="months")

abline(v=4,lty=2)

####prediction with Temperature * Conductivity interaction

tempo<-lm(pco1~temp+cond+temp:cond,data=bugenv)

anova(tempo) #*********#

summary(tempo) #*********#

fitted(tempo)

tempo1<-cbind(cbind(fitted(tempo),residuals(tempo)),bugenv[complete.cases(bugenv[,c("temp","cond")]),])

attributes(tempo1)

names(tempo1[,1:2])<-c("fitted","residual")

par(mar=c(5,5,4,2),cex=0.9)

plot(tempo1[tempo1$day==126,"dist"],tempo1[tempo1$day==126,"1"],type = "b",ylim=c(-.6,0.6),pch=19,col="gold2",cex=1.5,xlab="Distance(km)",ylab="PCO1",main="d) Prediction with Temperature * Conductivity interaction",lwd=3,lty=1)

#points(tempo1[tempo1$day==126,"dist"],tempo1[tempo1$day==126,"1"],type="b",col="gold2",pch=15,lwd=3,cex=1.5,lty=2)

points(tempo1[tempo1$day==260,"dist"],tempo1[tempo1$day==260,"1"],type="b",col="blue",pch=8,cex=1.5,lwd=3,lty=3)

points(tempo1[tempo1$day==336,"dist"],tempo1[tempo1$day==336,"1"],type="b",col="green3",pch=17,cex=1.5,lwd=3,lty=4)

points(tempo1[tempo1$day==518,"dist"],tempo1[tempo1$day==518,"1"],type="b",col="red",pch=18,cex=2,lwd=3,lty=5)

legend("topright",inset=c(0,0),legend=c("Dec 13", "April 14", "Aug 2014", "Nov 2014","May 2015"),lty=c(1,5),pch=c(19,15,8,17,18),lwd=2,col=c("black","gold2","blue","green3","red"),ncol=2,horiz=FALSE,cex=0.8,title="months")

abline(v=4,lty=2)

###### fitted and residual for environmental variables

tempo<-lm(pco1~tn+op+zn+temp+cond+temp:cond,data=bugenv)

summary(tempo)

fitted(tempo)

tempo1<-cbind(cbind(fitted(tempo),residuals(tempo)),bugenv[complete.cases(bugenv[,c("temp","cond")]),])

attributes(tempo1)

names(tempo1[,1:2])<-c("fitted","residual")

par(mar=c(5,5,4,2),cex=0.9)

plot(tempo1[tempo1$day==126,"dist"],tempo1[tempo1$day==126,"1"],type = "b",ylim=c(-.6,0.6),pch=19,col="gold2",cex=1.5,xlab="Distance(km)",ylab="PCO1",main="e) Prediction with Temperature * Conductivity interaction\n and other water quality parameters",lwd=3,lty=1)

#points(tempo1[tempo1$day==126,"dist"],tempo1[tempo1$day==126,"1"],type="b",col="gold2",pch=15,lwd=3,cex=1.5,lty=2)

points(tempo1[tempo1$day==260,"dist"],tempo1[tempo1$day==260,"1"],type="b",col="blue",pch=8,cex=1.5,lwd=3,lty=3)

points(tempo1[tempo1$day==336,"dist"],tempo1[tempo1$day==336,"1"],type="b",col="green3",pch=17,cex=1.5,lwd=3,lty=4)

points(tempo1[tempo1$day==518,"dist"],tempo1[tempo1$day==518,"1"],type="b",col="red",pch=18,cex=2,lwd=3,lty=5)

legend("topright",inset=c(0,0),legend=c("Dec 13", "April 14", "Aug 2014", "Nov 2014","May 2015"),lty=c(1,5),pch=c(19,15,8,17,18),lwd=2,col=c("black","gold2","blue","green3","red"),ncol=2,horiz=FALSE,cex=0.8,title="months")

abline(v=4,lty=2)

##PCO1 against distance

par(mar=c(5,5,4,2),cex=0.9)

plot(bugenv[bugenv$day==1,"dist"],bugenv[bugenv$day==1,"pco1"],type = "b",ylim=c(-.6,0.68),pch=19,cex=1.5,xlab="Distance(km)",ylab="PCO1",main="f) PCO1 plotted against spatial position",lwd=3,lty=1)

points(bugenv[bugenv$day==126,"dist"],bugenv[bugenv$day==126,"pco1"],type="b",col="gold2",pch=15,lwd=3,cex=1.5,lty=2)

points(bugenv[bugenv$day==260,"dist"],bugenv[bugenv$day==260,"pco1"],type="b",col="blue",pch=8,cex=1.5,lwd=3,lty=3)

points(bugenv[bugenv$day==336,"dist"],bugenv[bugenv$day==336,"pco1"],type="b",col="green3",pch=17,cex=1.5,lwd=3,lty=4)

points(bugenv[bugenv$day==518,"dist"],bugenv[bugenv$day==518,"pco1"],type="b",col="red",pch=18,cex=2,lwd=3,lty=5)

legend("topright",inset=c(0,0),legend=c("Dec 13", "April 14", "Aug 2014", "Nov 2014","May 2015"),lty=c(1,5),pch=c(19,15,8,17,18),lwd=2,col=c("black","gold2","blue","green3","red"),ncol=2,horiz=FALSE,cex=0.9,title="months")

abline(v=4,lty=2)

dev.off()

# residuals

par(mar=c(5,5,4,2),cex=0.9)

plot(tempo1[tempo1$day==126,"dist"],tempo1[tempo1$day==126,"2"],type = "b",ylim=c(-.6,0.6),pch=19,col="gold2",cex=1.5,xlab="Distance(km)",ylab="PCO1",main="residuals",lwd=3,lty=1)

#points(tempo1[tempo1$day==126,"dist"],tempo1[tempo1$day==126,"2"],type="b",col="gold2",pch=15,lwd=3,cex=1.5,lty=2)

points(tempo1[tempo1$day==260,"dist"],tempo1[tempo1$day==260,"2"],type="b",col="blue",pch=8,cex=1.5,lwd=3,lty=3)

points(tempo1[tempo1$day==336,"dist"],tempo1[tempo1$day==336,"2"],type="b",col="green3",pch=17,cex=1.5,lwd=3,lty=4)

points(tempo1[tempo1$day==518,"dist"],tempo1[tempo1$day==518,"2"],type="b",col="red",pch=18,cex=2,lwd=3,lty=5)

legend("topright",inset=c(0,0),legend=c("Dec 13", "April 14", "Aug 2014", "Nov 2014","May 2015"),lty=c(1,5),pch=c(19,15,8,17,18),lwd=2,col=c("black","gold2","blue","green3","red"),ncol=2,horiz=FALSE,cex=0.8,title="months")

abline(v=4,lty=2)

#####causal model 1 (Model building)##### Modelling macroinvertebrate community data as a function of space and time

#### dbRDA

##### Simulations to generate effluent and time vectors and add them to the data frame

wangenv$eff<-ifelse(wangenv$dist<4,0,1)

wangenv$time<-as.factor(wangenv$time)

#sim.pco2<-wangenv$eff

edit(wangenv)

#to subset times after time 1 can use wangenv$time%in%c("2","3","4","5")

#wang.cap1<-capscale(formula=wangbug.BC~sin(2*pi*day/365)+cos(2*pi*day/365)+sin(2*pi*day/365):dist+cos(2*pi*day/365):dist+eff:time+eff:time:dist,data=wangenv,comm=wangbug,add=TRUE,na.action=na.omit)

#wang.cap1<-capscale(formula=wangbug.BC~as.factor(eff)*time*dist,data=wangenv,comm=wangbug,add=TRUE,na.action=na.omit)

#wang.cap1<-capscale(formula=wangbug.BC~as.factor(eff)+time+dist+as.factor(eff):time,data=wangenv,comm=wangbug,add=TRUE,na.action=na.omit)

#wang.cap1<-capscale(formula=wangbug.BC~dist+time+dist:time+eff+eff:time+eff:dist+eff:dist:time,data=wangenv,comm=wangbug,add=TRUE,na.action=na.omit)

#wang.cap1<-capscale(formula=wangbug.BC~dist+time+dist:time+eff+eff:time+eff:dist:time,data=wangenv,comm=wangbug,add=TRUE,na.action=na.omit)

#wang.cap1<-capscale(formula=wangbug.BC~dist+time+dist:time+eff+eff:time+eff:dist:time,data=wangenv,comm=wangbug,add=TRUE,na.action=na.omit)

#wang.cap1<-capscale(formula=wangbug.BC~dist+time+dist:time+eff:time+eff:dist:time,data=wangenv,comm=wangbug,add=TRUE,na.action=na.omit)

#wang.cap1<-capscale(formula=wangbug.BC~eff+time+dist+eff:time+eff:dist+time:dist+eff:dist:time,data=wangenv,comm=wangbug,add=TRUE,na.action=na.omit) #

wang.cap1<-capscale(formula=wangbug.BC~dist+time+dist:time+eff+eff:time+eff:dist:time,data=wangenv,comm=wangbug,add=TRUE,na.action=na.omit)

summary(wang.cap1)

wang.anova1<-anova(wang.cap1,by="term",permutations = how(nperm=9999))

print(wang.anova1) #***** Table 1 *****# (ANOVA table for dbRDA model with space and time as predictors)

wang.cap1b<-capscale(formula=wangbug.BC~dist+time+dist:time,data=wangenv,comm=wangbug,add=TRUE,na.action=na.omit)

summary(wang.cap1b)

wang.anova1b<-anova(wang.cap1b,by="term")

print(wang.anova1b) #*********# Spatiotemporal model without effluent terms

anova(wang.cap1b,wang.cap1,permutations = how(nperm=9999))

# Calculate fitted and residual PCOs

#design<-scale(model.matrix(~-1+sin(2*pi*day/365)+cos(2*pi*day/365)+sin(2*pi*day/365):dist+cos(2*pi*day/365):dist+eff:time+eff:time*dist,data=wangenv),center=TRUE,scale=FALSE) #BIC=-119.6

#design<-scale(model.matrix(~-1+as.factor(eff)+time+dist+as.factor(eff):time,data=wangenv),center=FALSE,scale=FALSE) #BIC=-119.6 # construct centred desin matrix

#design<-scale(model.matrix(~-1+dist+time+dist:time+eff+eff:dist+eff:time,data=wangenv),center=FALSE,scale=FALSE) #BIC=-119.6 # construct centred desin matrix

#design<-scale(model.matrix(~-1+dist+time+eff+dist:time+eff:time+eff:dist:time,data=wangenv),center=FALSE,scale=FALSE) #BIC=-119.6 # construct centred desin matrix#Final1

#design<-scale(model.matrix(~-1+dist+time+dist:time,data=wangenv),center=FALSE,scale=FALSE) #BIC=-10.0192 # construct centred desin matrix

#design<-scale(model.matrix(~-1+as.factor(eff)+time+dist+as.factor(eff):time,data=wangenv),center=FALSE,scale=FALSE) #

b<-model.matrix(~-1+dist+time+dist:time+eff+eff:time+eff:dist:time,data=wangenv)

design<-scale(model.matrix(~-1+dist+time+dist:time+eff+eff:time+eff:dist:time,data=wangenv),center=FALSE,scale=FALSE) #

edit(design)

edit(b)

pco.beta<-qr.coef(qr(design),pwangbug)

edit(pco.beta)

dim(pco.beta)

pco.predict<-qr.fitted(qr(design),pwangbug)

pco.resid<-pwangbug-pco.predict # Compute PCO residuals

dim(pco.resid)

colnames(pco.resid)<-c(paste("res",sep="",1:p))

colnames(pco.predict)<-c(paste("pred",sep="",1:p))

envpcores<-cbind(wangenv,pco.resid)

envpcopred<-cbind(wangenv,pco.predict)

##compute AIC and BIC

sum(diag(var(pwangbug)))*p

sum(diag(var(pco.predict)))*p

100*(sum(diag(var(pco.predict)))*p)/(sum(diag(var(pwangbug)))*p)

SS.res<-sum(diag(var(pco.resid)))*p

SS.res

AIC<-n*log(SS.res/n)+2*dim(design)[2]

AIC# -26.26407

BIC<-n*log(SS.res/n)+log(n)*dim(design)[2]

BIC

edit(pco.resid)

edit(envpcopred)

alldata<-cbind(envpcopred,pwangbug)#

####plot predicted and residual values vs. distance and time

##pred1 ###

par(mar=c(5,5,4,2),cex=0.9)

plot(alldata[alldata$day==1,"dist"],alldata[alldata$day==1,"pco1"],type = "p",ylim=c(-.6,1),pch=19,cex=1.5,xlab="Distance(km)",ylab="Pred1 score",main="Predicted values plotted against spatial position",lwd=3,lty=5)

points(alldata[alldata$day==126,"dist"],alldata[alldata$day==126,"pco1"],type="p",col="gold",pch=8,lwd=3,cex=1.5,lty=4)

points(alldata[alldata$day==260,"dist"],alldata[alldata$day==260,"pco1"],type="p",col="blue",pch=15,cex=1.5,lwd=3,lty=3)

points(alldata[alldata$day==336,"dist"],alldata[alldata$day==336,"pco1"],type="p",col="green",pch=17,cex=1.5,lwd=3,lty=2)

points(alldata[alldata$day==518,"dist"],alldata[alldata$day==518,"pco1"],type="p",col="red",pch=17,cex=1.5,lwd=3,lty=2)

points(alldata[alldata$day==1,"dist"],envpcopred[envpcopred$day==1,"pred1"],type = "l",ylim=c(-.6,1),pch=19,cex=1.5,xlab="Distance(km)",ylab="Pred1 score",main="Predicted values plotted against spatial position",lwd=3,lty=5)

points(alldata[alldata$day==126,"dist"],envpcopred[envpcopred$day==126,"pred1"],type="l",col="gold",pch=8,lwd=3,cex=1.5,lty=4)

points(alldata[alldata$day==260,"dist"],envpcopred[envpcopred$day==260,"pred1"],type="l",col="blue",pch=15,cex=1.5,lwd=3,lty=3)

points(alldata[alldata$day==336,"dist"],envpcopred[envpcopred$day==336,"pred1"],type="l",col="green",pch=17,cex=1.5,lwd=3,lty=2)

points(alldata[alldata$day==518,"dist"],envpcopred[envpcopred$day==518,"pred1"],type="l",col="red",pch=17,cex=1.5,lwd=3,lty=2)

legend("topright",inset=c(0,0),legend=c("Feb 14", "May 14", "Sep 2014", "Dec 2014"),lty=c(1,5),pch=c(19,15,8,17),lwd=2,col=c("black","blue","green3","red"),ncol=2,horiz=FALSE,cex=0.9,title="months")

abline(v=10.9,lty=2)

xyplot(pred1~time,groups=dist,data=envpcopred,type="b",pch=c(1:6),auto.key=list(columns=4))

xyplot(pred1~dist,groups=time,data=envpcopred,type="b",auto.key=list(columns=5))

xyplot(res1~time,groups=dist,data=envpcores,type="b",pch=c(1:6),auto.key=list(columns=4))

xyplot(res1~dist,groups=time,data=envpcores,type="b",auto.key=list(columns=5))

xyplot(pred2~time,groups=dist,data=envpcopred,type="b",pch=c(1:6),auto.key=list(columns=4))

xyplot(pred2~dist,groups=time,data=envpcopred,type="b",auto.key=list(columns=5))

xyplot(res2~time,groups=dist,data=envpcores,type="b",pch=c(1:6),auto.key=list(columns=4))

xyplot(res2~dist,groups=time,data=envpcores,type="b",auto.key=list(columns=5))

#####

tempo<-lm(pco1~tn+op+zn+temp+cond+temp:cond,data=alldata)

summary(tempo)

fitted(tempo)

tempo1<-cbind(cbind(fitted(tempo),residuals(tempo)),alldata[complete.cases(alldata[,c("temp","cond")]),])

attributes(tempo1)

names(tempo1[,1:2])<-c("fitted","residual")

tempo3<-lm(tempo1[,2]~tempo1$pred1) ### first we need to caculate the predicted values for pcos by modelling them as a function of time and space

summary(tempo3)#p-value: 0.07102 # Fitted values in the model above explain all the variation seen in predicted values of pcos

#########################################

# Residual diagnostics

resid.d<-vegdist(pco.resid,method="euclidean") #Distance matrix of residuals for Mantel correlogram

dist.d<-dist(scale(wangenv[,c("day")],scale=FALSE),method="euclidean") #Distance matrix of sampling times for Mantel correlogram

plot(mgram(resid.d,dist.d),xlab="Lag") # mantel correlogram with function "mgram" in ecodist package

plot(mantel.correlog(resid.d,dist.d,nperm=99,n.class=16,cutoff=FALSE)) # mantel correlogram with function "mantel.correlog" in vegan package

dev.off()

## Plot predictions

max(wangenv$dist)

#Generate predictions

xygrid<-expand.grid(seq(1,5,1),seq(0,7.1,0.1))

colnames(xygrid)<-c("time","dist")

edit(xygrid)

#days are 1,125,260,336,518

for (i in 1:dim(xygrid)[1]) {

if (xygrid$time[i]==1) xygrid$day[i]<-1

if (xygrid$time[i]==2) xygrid$day[i]<-125

if (xygrid$time[i]==3) xygrid$day[i]<-260

if (xygrid$time[i]==4) xygrid$day[i]<-336

if (xygrid$time[i]==5) xygrid$day[i]<-518

}

#xygrid$eff<-as.factor(ifelse(xygrid$dist<4,0,1))

xygrid$eff<-ifelse(xygrid$dist<4,0,1)

colnames(xygrid)<-c("time","dist","day","eff")

xygrid$time<-as.factor(xygrid$time)

edit(xygrid) #use xygrid as data for design

#design2<-scale(model.matrix(~-1+sin(2*pi*day/365)+cos(2*pi*day/365)+sin(2*pi*day/365):dist+cos(2*pi*day/365):dist+eff:time+eff:time*dist,data=xygrid),center=TRUE,scale=FALSE)

#design2<-scale(model.matrix(~-1+dist+time+dist:time+eff+eff:time+eff:dist:time,data=xygrid),center=FALSE,scale=FALSE)#

#design2<-scale(model.matrix(~-1+as.factor(eff)+time+dist+as.factor(eff):time,data=xygrid),center=FALSE,scale=FALSE) ## construct centred desin matrix

design2<-scale(model.matrix(~-1+dist+time+dist:time+as.factor(eff)+as.factor(eff):time+as.factor(eff):dist:time,data=xygrid),center=FALSE,scale=FALSE)

edit(design2)

dim(design2)

dim(pco.beta)

pco.predict2<-as.matrix(design2)%*%pco.beta # Compute fitted PCOs

edit(pco.predict2)

colnames(pco.predict2)<-c(paste("pred",sep="",1:p))

dim(xygrid)

dim(pco.predict2)

pco.predict2<-cbind(xygrid,pco.predict2)

edit(pco.predict2)

tempo<-lm(pco1~dist+time+dist:time+eff+eff:time+eff:dist:time,data=alldata)

summary(tempo) #***** Table 2*****#

#pco plots with spatiotemporal model predictions### #***** Figure 3 - Appendix A *****#

tiff(file="Figure 3 - Appendix A.jpg",width=14,height=14,units="in",pointsize = 12,bg ="transparent",res=800,compression="lzw")

par(mfrow=c(3,2), mar=c(4.5,4.5,2,0.5),cex=1.5,cex.axis=0.7,las=1,cex.main=0.8)

num<-1

#fig.name<-"wangpco1.tif"

pco.no<-paste("pco",num,sep="")

PCO.NO<-paste("PCO",num," (",pco.varpercent[num],"% of total variation)",sep="")

pred.no<-paste("pred",num,sep="")

#tiff(file=fig.name,width=7,height=5,units="in",pointsize = 12,bg = "transparent",res=300,compression="lzw")

#par(mar=c(5,5,4,2),cex=0.9)

plot(wangenv$dist[wangenv$time==1],pwangbug[wangenv$time==1,pco.no],type="p",pch=19,col="black",xlab="Distance (km)",ylab=PCO.NO,ylim=c(-.6,1),las=1,cex.lab=0.7,main="PCO1 with spatiotemporal model predictions")

points(wangenv$dist[wangenv$time==1],pwangbug[wangenv$time==2,pco.no],col="gold2",pch=15)

points(wangenv$dist[wangenv$time==1],pwangbug[wangenv$time==3,pco.no],col="blue",pch=8)

points(wangenv$dist[wangenv$time==1],pwangbug[wangenv$time==4,pco.no],col="green3",pch=17)

points(wangenv$dist[wangenv$time==1],pwangbug[wangenv$time==5,pco.no],col="red",pch=18)

lines(xygrid$dist[xygrid$time==1],pco.predict2[pco.predict2$time==1,pred.no],type="l",lty=1,col="black",lwd=2)

lines(xygrid$dist[xygrid$time==1],pco.predict2[pco.predict2$time==2,pred.no],type="l",lty=2,col="gold2",lwd=2)

lines(xygrid$dist[xygrid$time==1],pco.predict2[pco.predict2$time==3,pred.no],type="l",lty=3,col="blue",lwd=2)

lines(xygrid$dist[xygrid$time==1],pco.predict2[pco.predict2$time==4,pred.no],type="l",lty=4,col="green3",lwd=2)

lines(xygrid$dist[xygrid$time==1],pco.predict2[pco.predict2$time==5,pred.no],type="l",lty=5,col="red",lwd=2)

legend("topright",inset=c(0,0),legend=c("Dec 2013", "April 2014", "Aug 2014", "Nov 2014","May 2015"),lty=c(1,5),pch=c(19,15,8,17,18),lwd=2,col=c("black","gold2","blue","green3","red"),

ncol=2,horiz=FALSE,cex=0.5,title="months")

num<-2

#fig.name<-"wangpco2.tif"

pco.no<-paste("pco",num,sep="")

PCO.NO<-paste("PCO",num," (",pco.varpercent[num],"% of total variation)",sep="")

pred.no<-paste("pred",num,sep="")

#tiff(file=fig.name,width=7,height=5,units="in",pointsize = 12,bg = "transparent",res=300,compression="lzw")

#par(mar=c(5,5,4,2),cex=0.9)

plot(wangenv$dist[wangenv$time==1],pwangbug[wangenv$time==1,pco.no],type="p",pch=19,col="black",xlab="Distance (km)",ylab=PCO.NO,ylim=c(-.6,1),las=1,cex.lab=0.7,main="PCO2 with spatiotemporal model predictions")

points(wangenv$dist[wangenv$time==1],pwangbug[wangenv$time==2,pco.no],col="gold2",pch=15)

points(wangenv$dist[wangenv$time==1],pwangbug[wangenv$time==3,pco.no],col="blue",pch=8)

points(wangenv$dist[wangenv$time==1],pwangbug[wangenv$time==4,pco.no],col="green3",pch=17)

points(wangenv$dist[wangenv$time==1],pwangbug[wangenv$time==5,pco.no],col="red",pch=18)

lines(xygrid$dist[xygrid$time==1],pco.predict2[pco.predict2$time==1,pred.no],type="l",lty=1,col="black",lwd=2)

lines(xygrid$dist[xygrid$time==1],pco.predict2[pco.predict2$time==2,pred.no],type="l",lty=2,col="gold2",lwd=2)

lines(xygrid$dist[xygrid$time==1],pco.predict2[pco.predict2$time==3,pred.no],type="l",lty=3,col="blue",lwd=2)

lines(xygrid$dist[xygrid$time==1],pco.predict2[pco.predict2$time==4,pred.no],type="l",lty=4,col="green3",lwd=2)

lines(xygrid$dist[xygrid$time==1],pco.predict2[pco.predict2$time==5,pred.no],type="l",lty=5,col="red",lwd=2)

legend("topright",inset=c(0,0),legend=c("Dec 2013", "April 2014", "Aug 2014", "Nov 2014","May 2015"),lty=c(1,5),pch=c(19,15,8,17,18),lwd=2,col=c("black","gold2","blue","green3","red"),

ncol=2,horiz=FALSE,cex=0.5,title="months")

num<-3

#fig.name<-"wangpco3.tif"

pco.no<-paste("pco",num,sep="")

PCO.NO<-paste("PCO",num," (",pco.varpercent[num],"% of total variation)",sep="")

pred.no<-paste("pred",num,sep="")

#tiff(file=fig.name,width=7,height=5,units="in",pointsize = 12,bg = "transparent",res=300,compression="lzw")

#par(mar=c(5,5,4,2),cex=0.9)

plot(wangenv$dist[wangenv$time==1],pwangbug[wangenv$time==1,pco.no],type="p",pch=19,col="black",xlab="Distance (km)",ylab=PCO.NO,ylim=c(-.6,1),las=1,cex.lab=0.7,main="PCO3 with spatiotemporal model predictions")

points(wangenv$dist[wangenv$time==1],pwangbug[wangenv$time==2,pco.no],col="gold2",pch=15)

points(wangenv$dist[wangenv$time==1],pwangbug[wangenv$time==3,pco.no],col="blue",pch=8)

points(wangenv$dist[wangenv$time==1],pwangbug[wangenv$time==4,pco.no],col="green3",pch=17)

points(wangenv$dist[wangenv$time==1],pwangbug[wangenv$time==5,pco.no],col="red",pch=18)

lines(xygrid$dist[xygrid$time==1],pco.predict2[pco.predict2$time==1,pred.no],type="l",lty=1,col="black",lwd=2)

lines(xygrid$dist[xygrid$time==1],pco.predict2[pco.predict2$time==2,pred.no],type="l",lty=2,col="gold2",lwd=2)

lines(xygrid$dist[xygrid$time==1],pco.predict2[pco.predict2$time==3,pred.no],type="l",lty=3,col="blue",lwd=2)

lines(xygrid$dist[xygrid$time==1],pco.predict2[pco.predict2$time==4,pred.no],type="l",lty=4,col="green3",lwd=2)

lines(xygrid$dist[xygrid$time==1],pco.predict2[pco.predict2$time==5,pred.no],type="l",lty=5,col="red",lwd=2)

legend("topright",inset=c(0,0),legend=c("Dec 2013", "April 2014", "Aug 2014", "Nov 2014","May 2015"),lty=c(1,5),pch=c(19,15,8,17,18),lwd=2,col=c("black","gold2","blue","green3","red"),

ncol=2,horiz=FALSE,cex=0.5,title="months")

num<-4

#fig.name<-"wangpco4.tif"

pco.no<-paste("pco",num,sep="")

PCO.NO<-paste("PCO",num," (",pco.varpercent[num],"% of total variation)",sep="")

pred.no<-paste("pred",num,sep="")

#tiff(file=fig.name,width=7,height=5,units="in",pointsize = 12,bg = "transparent",res=300,compression="lzw")

#par(mar=c(5,5,4,2),cex=0.9)

plot(wangenv$dist[wangenv$time==1],pwangbug[wangenv$time==1,pco.no],type="p",pch=19,col="black",xlab="Distance (km)",ylab=PCO.NO,ylim=c(-.6,1),las=1,cex.lab=0.7,main="PCO4 with spatiotemporal model predictions")

points(wangenv$dist[wangenv$time==1],pwangbug[wangenv$time==2,pco.no],col="gold2",pch=15)

points(wangenv$dist[wangenv$time==1],pwangbug[wangenv$time==3,pco.no],col="blue",pch=8)

points(wangenv$dist[wangenv$time==1],pwangbug[wangenv$time==4,pco.no],col="green3",pch=17)

points(wangenv$dist[wangenv$time==1],pwangbug[wangenv$time==5,pco.no],col="red",pch=18)

lines(xygrid$dist[xygrid$time==1],pco.predict2[pco.predict2$time==1,pred.no],type="l",lty=1,col="black",lwd=2)

lines(xygrid$dist[xygrid$time==1],pco.predict2[pco.predict2$time==2,pred.no],type="l",lty=2,col="gold2",lwd=2)

lines(xygrid$dist[xygrid$time==1],pco.predict2[pco.predict2$time==3,pred.no],type="l",lty=3,col="blue",lwd=2)

lines(xygrid$dist[xygrid$time==1],pco.predict2[pco.predict2$time==4,pred.no],type="l",lty=4,col="green3",lwd=2)

lines(xygrid$dist[xygrid$time==1],pco.predict2[pco.predict2$time==5,pred.no],type="l",lty=5,col="red",lwd=2)

legend("topright",inset=c(0,0),legend=c("Dec 2013", "April 2014", "Aug 2014", "Nov 2014","May 2015"),lty=c(1,5),pch=c(19,15,8,17,18),lwd=2,col=c("black","gold2","blue","green3","red"),

ncol=2,horiz=FALSE,cex=0.5,title="months")

num<-5

#fig.name<-"wangpco5.tif"

pco.no<-paste("pco",num,sep="")

PCO.NO<-paste("PCO",num," (",pco.varpercent[num],"% of total variation)",sep="")

pred.no<-paste("pred",num,sep="")

#tiff(file=fig.name,width=7,height=5,units="in",pointsize = 12,bg = "transparent",res=300,compression="lzw")

#par(mar=c(5,5,4,2),cex=0.9)

plot(wangenv$dist[wangenv$time==1],pwangbug[wangenv$time==1,pco.no],type="p",pch=19,col="black",xlab="Distance (km)",ylab=PCO.NO,ylim=c(-.6,1),las=1,cex.lab=0.7,main="PCO5 with spatiotemporal model predictions")

points(wangenv$dist[wangenv$time==1],pwangbug[wangenv$time==2,pco.no],col="gold2",pch=15)

points(wangenv$dist[wangenv$time==1],pwangbug[wangenv$time==3,pco.no],col="blue",pch=8)

points(wangenv$dist[wangenv$time==1],pwangbug[wangenv$time==4,pco.no],col="green3",pch=17)

points(wangenv$dist[wangenv$time==1],pwangbug[wangenv$time==5,pco.no],col="red",pch=18)

lines(xygrid$dist[xygrid$time==1],pco.predict2[pco.predict2$time==1,pred.no],type="l",lty=1,col="black",lwd=2)

lines(xygrid$dist[xygrid$time==1],pco.predict2[pco.predict2$time==2,pred.no],type="l",lty=2,col="gold2",lwd=2)

lines(xygrid$dist[xygrid$time==1],pco.predict2[pco.predict2$time==3,pred.no],type="l",lty=3,col="blue",lwd=2)

lines(xygrid$dist[xygrid$time==1],pco.predict2[pco.predict2$time==4,pred.no],type="l",lty=4,col="green3",lwd=2)

lines(xygrid$dist[xygrid$time==1],pco.predict2[pco.predict2$time==5,pred.no],type="l",lty=5,col="red",lwd=2)

legend("topright",inset=c(0,0),legend=c("Dec 2013", "April 2014", "Aug 2014", "Nov 2014","May 2015"),lty=c(1,5),pch=c(19,15,8,17,18),lwd=2,col=c("black","gold2","blue","green3","red"),

ncol=2,horiz=FALSE,cex=0.5,title="months")

num<-6

#fig.name<-"wangpco6.tif"

pco.no<-paste("pco",num,sep="")

PCO.NO<-paste("PCO",num," (",pco.varpercent[num],"% of total variation)",sep="")

pred.no<-paste("pred",num,sep="")

#tiff(file=fig.name,width=7,height=5,units="in",pointsize = 12,bg = "transparent",res=300,compression="lzw")

#par(mar=c(5,5,4,2),cex=0.9)

plot(wangenv$dist[wangenv$time==1],pwangbug[wangenv$time==1,pco.no],type="p",pch=19,col="black",xlab="Distance (km)",ylab=PCO.NO,ylim=c(-.6,1),las=1,cex.lab=0.7,main="PCO6 with spatiotemporal model predictions")

points(wangenv$dist[wangenv$time==1],pwangbug[wangenv$time==2,pco.no],col="gold2",pch=15)

points(wangenv$dist[wangenv$time==1],pwangbug[wangenv$time==3,pco.no],col="blue",pch=8)

points(wangenv$dist[wangenv$time==1],pwangbug[wangenv$time==4,pco.no],col="green3",pch=17)

points(wangenv$dist[wangenv$time==1],pwangbug[wangenv$time==5,pco.no],col="red",pch=18)

lines(xygrid$dist[xygrid$time==1],pco.predict2[pco.predict2$time==1,pred.no],type="l",lty=1,col="black",lwd=2)

lines(xygrid$dist[xygrid$time==1],pco.predict2[pco.predict2$time==2,pred.no],type="l",lty=2,col="gold2",lwd=2)

lines(xygrid$dist[xygrid$time==1],pco.predict2[pco.predict2$time==3,pred.no],type="l",lty=3,col="blue",lwd=2)

lines(xygrid$dist[xygrid$time==1],pco.predict2[pco.predict2$time==4,pred.no],type="l",lty=4,col="green3",lwd=2)

lines(xygrid$dist[xygrid$time==1],pco.predict2[pco.predict2$time==5,pred.no],type="l",lty=5,col="red",lwd=2)

legend("topright",inset=c(0,0),legend=c("Dec 2013", "April 2014", "Aug 2014", "Nov 2014","May 2015"),lty=c(1,5),pch=c(19,15,8,17,18),lwd=2,col=c("black","gold2","blue","green3","red"),

ncol=2,horiz=FALSE,cex=0.5,title="months")

dev.off()

## predicted and residuals plots

bugenvres<-cbind(bugenv,pco.resid)

edit(pco.resid)

xyplot(res1~day,groups=dist,data=envpcores,type="b",pch=c(1:6),auto.key=list(columns=2))

xyplot(res1~dist,groups=day,data=envpcores,type="b",auto.key=list(columns=5))

edit(design)

edit(bugenvres)

###residuals after modeling pco as a function of time, distance, and effluent #***** Residuals after modeling pco *****#

tiff(file="residuals after modeling pco.jpg",width=8,height=10,units="in",pointsize = 16,bg ="transparent",res=800,compression="lzw")

par(mfrow=c(2,1),cex.axis=0.7,las=1,cex.main=1)

par(mar=c(5,5,4,4),cex=0.9)

plot(bugenvres[bugenvres$day==1,"dist"],bugenvres[bugenvres$day==1,"res1"],type = "b",ylim=c(-.8,.88),pch=19,cex=1.5,xlab="Distance(km)",ylab="Residuals",main="Residual plotted against spatial position",lwd=3,lty=1)

points(bugenvres[bugenvres$day==126,"dist"],bugenvres[bugenvres$day==126,"res1"],type="b",col="gold2",pch=15,lwd=3,cex=1.5,lty=2)

points(bugenvres[bugenvres$day==260,"dist"],bugenvres[bugenvres$day==260,"res1"],type="b",col="blue",pch=8,cex=1.5,lwd=3,lty=3)

points(bugenvres[bugenvres$day==336,"dist"],bugenvres[bugenvres$day==336,"res1"],type="b",col="green3",pch=17,cex=1.5,lwd=3,lty=4)

points(bugenvres[bugenvres$day==518,"dist"],bugenvres[bugenvres$day==518,"res1"],type="b",col="red",pch=18,cex=2,lwd=3,lty=5)

legend("topright",inset=c(0,0),legend=c("Dec 13", "April 14", "Aug 2014", "Nov 2014","May 2015"),lty=c(1,5),pch=c(19,15,8,17,18),lwd=2,col=c("black","gold2","blue","green3","red"),ncol=2,horiz=FALSE,cex=0.8,title="months")

abline(v=4,lty=2)

par(mar=c(5,5,5,5),cex=0.9)

plot(mydates,bugenvres[bugenvres$dist==0,"res1"],type = "b",ylim=c(-.8,.8),pch=19,cex=1.5,xlab="day",ylab="Residuals",main="Residuals plotted against time",lwd=3,lty=2,cex.main=1,xaxt="n")

points(mydates,bugenvres[bugenvres$dist==1.8,"res1"],type="b",col="deep pink",pch=15,lwd=3,cex=1,lty=3)

points(mydates,bugenvres[bugenvres$dist==3.2,"res1"],type="b",col="blue",pch=8,cex=1,lwd=3,lty=4)

points(mydates,bugenvres[bugenvres$dist==3.9,"res1"],type="b",col="green",pch=17,cex=1,lwd=3,lty=5)

points(mydates,bugenvres[bugenvres$dist==4.08,"res1"],type="b",col="red",pch=18,cex=1.5,lwd=3,lty=6)

points(mydates,bugenvres[bugenvres$dist==4.83,"res1"],type="b",col="gold2",pch=18,cex=1.52,lwd=3,lty=7)

points(mydates,bugenvres[bugenvres$dist==6.53,"res1"],type="b",col="lightseagreen",pch=18,cex=1.5,lwd=3,lty=6)

points(mydates,bugenvres[bugenvres$dist==7.08,"res1"],type="b",col="sienna2",pch=18,cex=1.5,lwd=3,lty=6)

labels<-axis.Date(side=1,mydates,at=seq(mydaterange[1],mydaterange[2],by="month"),format="%b-%y")

legend("bottomright",inset=c(0,0),legend=levels(as.factor(bugenv$dist)),lty=1,lwd=2,col=c("black","deep pink","blue","green","red","gold2","lightseagreen","sienna2"),ncol=4,horiz=FALSE,cex=0.8,title="distance(km)")

dev.off()

### Before using dbRDA for modlelling bugs as a function of environmental variables, we check all the possible realtionships using correlation and scatterplots

########dbRDA for environmental variables ########

#wang.cap1<-capscale(formula=wangbug.BC~as.factor(eff)+time+dist+as.factor(eff):time,data=wangenv,comm=wangbug,add=TRUE,na.action=na.omit)

wang.cap1<-capscale(formula=wangbug.BC~dist+time+dist:time+eff+eff:time+eff:dist:time,data=wangenv,comm=wangbug,add=TRUE,na.action=na.omit)

summary(wang.cap1)

wang.anova1<-anova(wang.cap1,by="term")

print(wang.anova1)

edit(same.sites(wangbug,wangenv1))

wangenv1<-wangenv[complete.cases(wangenv[,c("vel","alk","sed","ph2","turb","toc","cfpom","temp","no2","nh3","chla","cond","on")]),]#omit season1

edit(wangenv1)

wangenv1<- removeNAenv(wangenv1,"vel")

wangenv1<- removeNAenv(wangenv1,"alk")

wangenv1<- removeNAenv(wangenv1,"sed")

wangenv1<- removeNAenv(wangenv1,"ph2")

wangenv1<- removeNAenv(wangenv1,"turb")

wangenv1<- removeNAenv(wangenv1,"toc")

wangenv1<- removeNAenv(wangenv1,"cfpom")

wangenv1<- removeNAenv(wangenv1,"temp")

wangenv1<- removeNAenv(wangenv1,"no2")

wangenv1<- removeNAenv(wangenv1,"nh3")

wangenv1<- removeNAenv(wangenv1,"chla")

wangenv1<- removeNAenv(wangenv1,"cond")

wangenv1<- removeNAenv(wangenv1,"on")

### same number of sites for both macroinvertebrate and environmental data because of missing dada

wangbug1<-same.sites(wangbug,wangenv1)

edit(wangbug1)

check.datasets(wangbug1,wangenv1)

edit(wangenv1)

##### before dbRDA , first any possible relationship between environmental variables and PCOs is examined using correlation and scatter plots

#####catter plot and more plots

#####Scatterplot Matrices/first two PCOs and environmental variables #**********#

tiff(file="scatterplot.tif",width=20,height=14,units="in",pointsize = 12,bg = "transparent",res=1200,compression="lzw")

par(mar=c(5,5,4,2),cex=0.9)

panel.cor <- function(x, y, digits = 2, cex.cor, ...)

{

usr <- par("usr"); on.exit(par(usr))

par(usr = c(0, 1, 0, 1))

# correlation coefficient

r <- cor(x, y)

method=c("spearman")

txt <- format(c(r, 0.123456789), digits = digits)[1]

txt <- paste("r= ", txt, sep = "")

text(0.5, 0.8, txt)

# p-value calculation

p <- cor.test(x, y)$p.value

txt2 <- format(c(p, 0.123456789), digits = digits)[1]

txt2 <- paste("p= ", txt2, sep = "")

if(p<0.01) txt2 <- paste("p= ", "<0.01", sep = "")

text(0.5, 0.3, txt2)

}

pairs(~cd+cu+cr+log(zn)+alk+temp+log(cond)+do+log(toc)+log(cfpom)+log(chla)+ph2+turb+sed+vel+log(tp)+log(nh3)+no3+no2+on+op+pco1+pco2,data=alldata,upper.panel=panel.cor,pch=20,na.action = na.omit)

dev.off()

#########first two PCOs and environmental variables (log transformed)

tiff(file="scatterplot.tif",width=20,height=14,units="in",pointsize = 12,bg = "transparent",res=1200,compression="lzw")

par(mar=c(5,5,4,2),cex=0.9)

panel.cor <- function(x, y, digits = 2, cex.cor, ...)

{

usr <- par("usr"); on.exit(par(usr))

par(usr = c(0, 1, 0, 1))

# correlation coefficient

r <- cor(x, y)

method=c("spearman")

txt <- format(c(r, 0.123456789), digits = digits)[1]

txt <- paste("r= ", txt, sep = "")

text(0.5, 0.8, txt)

# p-value calculation

p <- cor.test(x, y)$p.value

txt2 <- format(c(p, 0.123456789), digits = digits)[1]

txt2 <- paste("p= ", txt2, sep = "")

if(p<0.01) txt2 <- paste("p= ", "<0.01", sep = "")

text(0.5, 0.3, txt2)

}

pairs(~log1p(cd)+log1p(cu)+log1p(cr)+log1p(zn)+log1p(alk)+log1p(temp)+log1p(cond)+log1p(do)+log1p(toc)+log1p(cfpom)+log1p(chla)+ph2+log1p(turb)+log1p(sed)+log1p(vel)+log1p(tp)+log1p(nh3)+log1p(no3)+log1p(no2)+log1p(on)+log1p(op)+pco1+pco2,data=alldata,upper.panel=panel.cor,pch=20,na.action = na.omit)

dev.off()

######### more exploratory analysis

tiff(file="first two PCOs, pH, salinity, and DO",width=14,height=14,units="in",pointsize = 12,bg ="transparent",res=800,compression="lzw")

par(mar=c(5,5,4,2),cex=0.9)

panel.cor <- function(x, y, digits = 2, cex.cor, ...)

{

usr <- par("usr"); on.exit(par(usr))

par(usr = c(0, 1, 0, 1))

# correlation coefficient

r <- cor(x, y)

method=c("spearman")

txt <- format(c(r, 0.123456789), digits = digits)[1]

txt <- paste("r= ", txt, sep = "")

text(0.5, 0.8, txt)

# p-value calculation

p <- cor.test(x, y)$p.value

txt2 <- format(c(p, 0.123456789), digits = digits)[1]

txt2 <- paste("p= ", txt2, sep = "")

if(p<0.01) txt2 <- paste("p= ", "<0.01", sep = "")

text(0.5, 0.3, txt2)

}

pairs(~ph1+sali+do+pco1+pco2,data=alldata,upper.panel=panel.cor,pch=20,na.action = na.omit)

dev.off()

#########

tiff(file="first two PCOs and environmental variables (transformed)",width=14,height=14,units="in",pointsize = 12,bg ="transparent",res=800,compression="lzw")

par(mar=c(5,5,4,2),cex=0.9)

panel.cor <- function(x, y, digits = 2, cex.cor, ...)

{

usr <- par("usr"); on.exit(par(usr))

par(usr = c(0, 1, 0, 1))

# correlation coefficient

r <- cor(x, y)

method=c("spearman")

txt <- format(c(r, 0.123456789), digits = digits)[1]

txt <- paste("r= ", txt, sep = "")

text(0.5, 0.8, txt)

# p-value calculation

p <- cor.test(x, y)$p.value

txt2 <- format(c(p, 0.123456789), digits = digits)[1]

txt2 <- paste("p= ", txt2, sep = "")

if(p<0.01) txt2 <- paste("p= ", "<0.01", sep = "")

text(0.5, 0.3, txt2)

}

pairs(~log(cd)+log(cu)+log(cr)+log(zn)+temp+sqrt(cod)+log(cfpom)+log(chla)+log(ph2)+log(turb)+asin(vel)+pco1+pco2,data=alldata,upper.panel=panel.cor,pch=20)

dev.off()

#########

tiff(file="first two PCOs , pH, salinity and DO(transformed)",width=14,height=14,units="in",pointsize = 12,bg ="transparent",res=800,compression="lzw")

par(mar=c(5,5,4,2),cex=0.9)

panel.cor <- function(x, y, digits = 2, cex.cor, ...)

{

usr <- par("usr"); on.exit(par(usr))

par(usr = c(0, 1, 0, 1))

# correlation coefficient

r <- cor(x, y)

method=c("spearman")

txt <- format(c(r, 0.123456789), digits = digits)[1]

txt <- paste("r= ", txt, sep = "")

text(0.5, 0.8, txt)

# p-value calculation

p <- cor.test(x, y)$p.value

txt2 <- format(c(p, 0.123456789), digits = digits)[1]

txt2 <- paste("p= ", txt2, sep = "")

if(p<0.01) txt2 <- paste("p= ", "<0.01", sep = "")

text(0.5, 0.3, txt2)

}

pairs(~log(ph1)+log(sali)+sqrt(do)+pco1+pco2,data=alldata,upper.panel=panel.cor,pch=20,na.action = na.omit)

dev.off()

#######Scatterplot of Pred1 against velocity, ...

tiff(file="first two predicted PCOs and environmental variables)",width=14,height=14,units="in",pointsize = 12,bg ="transparent",res=800,compression="lzw")

par(mar=c(5,5,4,2),cex=0.9)

panel.cor <- function(x, y, digits = 2, cex.cor, ...)

{

usr <- par("usr"); on.exit(par(usr))

par(usr = c(0, 1, 0, 1))

# correlation coefficient

r <- cor(x, y)

method=c("spearman")

txt <- format(c(r, 0.123456789), digits = digits)[1]

txt <- paste("r= ", txt, sep = "")

text(0.5, 0.8, txt)

# p-value calculation

p <- cor.test(x, y)$p.value

txt2 <- format(c(p, 0.123456789), digits = digits)[1]

txt2 <- paste("p= ", txt2, sep = "")

if(p<0.01) txt2 <- paste("p= ", "<0.01", sep = "")

text(0.5, 0.3, txt2)

}

pairs(~cd+cu+cr+zn+cod+temp+cfpom+chla+vel+ph2+turb+sed+pred1+pred2,data=alldata,upper.panel=panel.cor,pch=20)

dev.off()

######

tiff(file="first two predicted PCOs and environmental variables(TRANSFORMED))",width=14,height=14,units="in",pointsize = 12,bg ="transparent",res=800,compression="lzw")

par(mar=c(5,5,4,2),cex=0.9)

panel.cor <- function(x, y, digits = 2, cex.cor, ...)

{

usr <- par("usr"); on.exit(par(usr))

par(usr = c(0, 1, 0, 1))

# correlation coefficient

r <- cor(x, y)

method=c("spearman")

txt <- format(c(r, 0.123456789), digits = digits)[1]

txt <- paste("r= ", txt, sep = "")

text(0.5, 0.8, txt)

# p-value calculation

p <- cor.test(x, y)$p.value

txt2 <- format(c(p, 0.123456789), digits = digits)[1]

txt2 <- paste("p= ", txt2, sep = "")

if(p<0.01) txt2 <- paste("p= ", "<0.01", sep = "")

text(0.5, 0.3, txt2)

}

pairs(~log(cd)+log(cu)+log(cr)+log(zn)+cod+temp+log(cfpom)+log(chla)+vel+log(ph2)+turb+log(sed)+pred1+pred2,data=alldata,upper.panel=panel.cor,pch=20)

dev.off()

######

tiff(file="first two predicted PCOs , salinity, ph and DO",width=14,height=14,units="in",pointsize = 12,bg ="transparent",res=800,compression="lzw")

par(mar=c(5,5,4,2),cex=0.9)

panel.cor <- function(x, y, digits = 2, cex.cor, ...)

{usr <- par("usr"); on.exit(par(usr))

par(usr = c(0, 1, 0, 1))

# correlation coefficient

r <- cor(x, y)

method=c("spearman")

txt <- format(c(r, 0.123456789), digits = digits)[1]

txt <- paste("r= ", txt, sep = "")

text(0.5, 0.8, txt)

# p-value calculation

p <- cor.test(x, y)$p.value

txt2 <- format(c(p, 0.123456789), digits = digits)[1]

txt2 <- paste("p= ", txt2, sep = "")

if(p<0.01) txt2 <- paste("p= ", "<0.01", sep = "")

text(0.5, 0.3, txt2)}

pairs(~sali+do+ph1+ph2+pred1+pred2,data=alldata,upper.panel=panel.cor,pch=20,na.action = na.omit)

dev.off()

###########velocity and other factors

tiff(file="velocity and other factors",width=14,height=14,units="in",pointsize = 12,bg ="transparent",res=800,compression="lzw")

par(mar=c(5,5,4,2),cex=0.9)

panel.cor <- function(x, y, digits = 2, cex.cor, ...)

{usr <- par("usr"); on.exit(par(usr))

par(usr = c(0, 1, 0, 1))

# correlation coefficient

r <- cor(x, y)

method=c("spearman")

txt <- format(c(r, 0.123456789), digits = digits)[1]

txt <- paste("r= ", txt, sep = "")

text(0.5, 0.8, txt)

# p-value calculation

p <- cor.test(x, y)$p.value

txt2 <- format(c(p, 0.123456789), digits = digits)[1]

txt2 <- paste("p= ", txt2, sep = "")

if(p<0.01) txt2 <- paste("p= ", "<0.01", sep = "")

text(0.5, 0.3, txt2)}

pairs(~log(vel)+log(dflow)+log(depth)+log(width)+turb+log(chla)+log(sed)+log(ph2),data=alldata,upper.panel=panel.cor,pch=20)

dev.off()

########### water temperature and other factors

tiff(file="water temperature and other factors.tiff",width=14,height=14,units="in",pointsize = 12,bg ="transparent",res=800,compression="lzw")

par(mar=c(5,5,4,2),cex=0.9)

panel.cor <- function(x, y, digits = 2, cex.cor, ...)

{

usr <- par("usr"); on.exit(par(usr))

par(usr = c(0, 1, 0, 1))

# correlation coefficient

r <- cor(x, y)

method=c("spearman")

txt <- format(c(r, 0.123456789), digits = digits)[1]

txt <- paste("r= ", txt, sep = "")

text(0.5, 0.8, txt)

# p-value calculation

p <- cor.test(x, y)$p.value

txt2 <- format(c(p, 0.123456789), digits = digits)[1]

txt2 <- paste("p= ", txt2, sep = "")

if(p<0.01) txt2 <- paste("p= ", "<0.01", sep = "")

text(0.5, 0.3, txt2)

}

pairs(~temp+airtemp+eff+canop+veg30m+veg60m+veg90m,data=wangalldata,upper.panel=panel.cor,pch=20,na.action = na.omit)

dev.off()

######### pH and other factors

tiff(file="pH and other factors",width=14,height=14,units="in",pointsize = 12,bg ="transparent",res=800,compression="lzw")

par(mar=c(5,5,4,2),cex=0.9)

panel.cor <- function(x, y, digits = 2, cex.cor, ...)

{

usr <- par("usr"); on.exit(par(usr))

par(usr = c(0, 1, 0, 1))

# correlation coefficient

r <- cor(x, y)

method=c("spearman")

txt <- format(c(r, 0.123456789), digits = digits)[1]

txt <- paste("r= ", txt, sep = "")

text(0.5, 0.8, txt)

# p-value calculation

p <- cor.test(x, y)$p.value

txt2 <- format(c(p, 0.123456789), digits = digits)[1]

txt2 <- paste("p= ", txt2, sep = "")

if(p<0.01) txt2 <- paste("p= ", "<0.01", sep = "")

text(0.5, 0.3, txt2)

}

pairs(~ph1+ph2+chla+alk+eff+dflow,data=wangalldata,upper.panel=panel.cor,pch=20,na.action = na.omit)

dev.off()

##########

tiff(file="COD and other factors",width=14,height=14,units="in",pointsize = 12,bg ="transparent",res=800,compression="lzw")

par(mar=c(5,5,4,2),cex=0.9)

panel.cor <- function(x, y, digits = 2, cex.cor, ...)

{

usr <- par("usr"); on.exit(par(usr))

par(usr = c(0, 1, 0, 1))

# correlation coefficient

r <- cor(x, y)

method=c("spearman")

txt <- format(c(r, 0.123456789), digits = digits)[1]

txt <- paste("r= ", txt, sep = "")

text(0.5, 0.8, txt)

# p-value calculation

p <- cor.test(x, y)$p.value

txt2 <- format(c(p, 0.123456789), digits = digits)[1]

txt2 <- paste("p= ", txt2, sep = "")

if(p<0.01) txt2 <- paste("p= ", "<0.01", sep = "")

text(0.5, 0.3, txt2)

}

pairs(~cod+dcod+eff+dflow+canop+veg30m+veg60m+veg90m+graz1000+ind100+dtoc+toc+rain1,data=wangalldata,upper.panel=panel.cor,pch=20,na.action = na.omit)

dev.off()

######### Chl a and other factors

tiff(file="Chl a and other factors",width=14,height=14,units="in",pointsize = 12,bg ="transparent",res=800,compression="lzw")

par(mar=c(5,5,4,2),cex=0.9)

panel.cor <- function(x, y, digits = 2, cex.cor, ...)

{

usr <- par("usr"); on.exit(par(usr))

par(usr = c(0, 1, 0, 1))

# correlation coefficient

r <- cor(x, y)

method=c("spearman")

txt <- format(c(r, 0.123456789), digits = digits)[1]

txt <- paste("r= ", txt, sep = "")

text(0.5, 0.8, txt)

# p-value calculation

p <- cor.test(x, y)$p.value

txt2 <- format(c(p, 0.123456789), digits = digits)[1]

txt2 <- paste("p= ", txt2, sep = "")

if(p<0.01) txt2 <- paste("p= ", "<0.01", sep = "")

text(0.5, 0.3, txt2)

}

pairs(~chla+eff+dchla+dflow+canop+no3+tp+op+dtp+dop+dno3+no2+dno2+turb+veg30m+veg60m+veg90m+vel+temp,data=alldata,upper.panel=panel.cor,pch=20,na.action = na.omit)

dev.off()

##########CPOM/FPOM and other factors

tiff(file="CPOM and other factors",width=14,height=14,units="in",pointsize = 12,bg ="transparent",res=800,compression="lzw")

par(mar=c(5,5,4,2),cex=0.9)

panel.cor <- function(x, y, digits = 2, cex.cor, ...)

{

usr <- par("usr"); on.exit(par(usr))

par(usr = c(0, 1, 0, 1))

# correlation coefficient

r <- cor(x, y)

method=c("spearman")

txt <- format(c(r, 0.123456789), digits = digits)[1]

txt <- paste("r= ", txt, sep = "")

text(0.5, 0.8, txt)

# p-value calculation

p <- cor.test(x, y)$p.value

txt2 <- format(c(p, 0.123456789), digits = digits)[1]

txt2 <- paste("p= ", txt2, sep = "")

if(p<0.01) txt2 <- paste("p= ", "<0.01", sep = "")

text(0.5, 0.3, txt2)

}

pairs(~cfpom+dayflow+weekflow+monflow+eff+dtoc+dflow+canop+veg30m+veg60m+veg90m+graz1000+ind100+dtoc+toc+rain1,data=wangalldata,upper.panel=panel.cor,pch=20,na.action = na.omit)

dev.off()

##########Alkalinity and other factors

tiff(file="Alkalinity and other factors",width=14,height=14,units="in",pointsize = 12,bg ="transparent",res=800,compression="lzw")

par(mar=c(5,5,4,2),cex=0.9)

panel.cor <- function(x, y, digits = 2, cex.cor, ...)

{

usr <- par("usr"); on.exit(par(usr))

par(usr = c(0, 1, 0, 1))

# correlation coefficient

r <- cor(x, y)

method=c("spearman")

txt <- format(c(r, 0.123456789), digits = digits)[1]

txt <- paste("r= ", txt, sep = "")

text(0.5, 0.8, txt)

# p-value calculation

p <- cor.test(x, y)$p.value

txt2 <- format(c(p, 0.123456789), digits = digits)[1]

txt2 <- paste("p= ", txt2, sep = "")

if(p<0.01) txt2 <- paste("p= ", "<0.01", sep = "")

text(0.5, 0.3, txt2)

}

pairs(~alk+dflow+dalk+dcond+cond+dtemp+temp,data=wangalldata,upper.panel=panel.cor,pch=20,na.action = na.omit)

dev.off()

##########Nitrate and other factors

tiff(file="Nitrate and other factors",width=14,height=14,units="in",pointsize = 12,bg ="transparent",res=800,compression="lzw")

par(mar=c(5,5,4,2),cex=0.9)

panel.cor <- function(x, y, digits = 2, cex.cor, ...)

{

usr <- par("usr"); on.exit(par(usr))

par(usr = c(0, 1, 0, 1))

# correlation coefficient

r <- cor(x, y)

method=c("spearman")

txt <- format(c(r, 0.123456789), digits = digits)[1]

txt <- paste("r= ", txt, sep = "")

text(0.5, 0.8, txt)

# p-value calculation

p <- cor.test(x, y)$p.value

txt2 <- format(c(p, 0.123456789), digits = digits)[1]

txt2 <- paste("p= ", txt2, sep = "")

if(p<0.01) txt2 <- paste("p= ", "<0.01", sep = "")

text(0.5, 0.3, txt2)

}

pairs(~no3+tn+nh3+no2+dtn+dnh3+dno2+dno3+dflow+graz1000+ind100+eff,data=wangalldata,upper.panel=panel.cor,pch=20,na.action = na.omit)

dev.off()

##########

tiff(file="Phosphate and other factors",width=14,height=14,units="in",pointsize = 12,bg ="transparent",res=800,compression="lzw")

par(mar=c(5,5,4,2),cex=0.9)

panel.cor <- function(x, y, digits = 2, cex.cor, ...)

{

usr <- par("usr"); on.exit(par(usr))

par(usr = c(0, 1, 0, 1))

# correlation coefficient

r <- cor(x, y)

method=c("spearman")

txt <- format(c(r, 0.123456789), digits = digits)[1]

txt <- paste("r= ", txt, sep = "")

text(0.5, 0.8, txt)

# p-value calculation

p <- cor.test(x, y)$p.value

txt2 <- format(c(p, 0.123456789), digits = digits)[1]

txt2 <- paste("p= ", txt2, sep = "")

if(p<0.01) txt2 <- paste("p= ", "<0.01", sep = "")

text(0.5, 0.3, txt2)

}

pairs(~op+tp+graz1000+ind100+dflow+dop+dtp+eff,data=wangalldata,upper.panel=panel.cor,pch=20,na.action = na.omit)

dev.off()

########salinity and other factors

tiff(file="salinity and other factors",width=14,height=14,units="in",pointsize = 12,bg ="transparent",res=800,compression="lzw")

par(mar=c(5,5,4,2),cex=0.9)

panel.cor <- function(x, y, digits = 2, cex.cor, ...)

{

usr <- par("usr"); on.exit(par(usr))

par(usr = c(0, 1, 0, 1))

# correlation coefficient

r <- cor(x, y)

method=c("spearman")

txt <- format(c(r, 0.123456789), digits = digits)[1]

txt <- paste("r= ", txt, sep = "")

text(0.5, 0.8, txt)

# p-value calculation

p <- cor.test(x, y)$p.value

txt2 <- format(c(p, 0.123456789), digits = digits)[1]

txt2 <- paste("p= ", txt2, sep = "")

if(p<0.01) txt2 <- paste("p= ", "<0.01", sep = "")

text(0.5, 0.3, txt2)

}

pairs(~sali+graz1000+ind100+dflow+dsali+rain1+eff,data=wangalldata,upper.panel=panel.cor,pch=20,na.action = na.omit)

dev.off()

##########Heavy metals and other factors

tiff(file="Heavy metals and other factors",width=14,height=14,units="in",pointsize = 12,bg ="transparent",res=800,compression="lzw")

par(mar=c(5,5,4,2),cex=0.9)

panel.cor <- function(x, y, digits = 2, cex.cor, ...)

{

usr <- par("usr"); on.exit(par(usr))

par(usr = c(0, 1, 0, 1))

# correlation coefficient

r <- cor(x, y)

method=c("spearman")

txt <- format(c(r, 0.123456789), digits = digits)[1]

txt <- paste("r= ", txt, sep = "")

text(0.5, 0.8, txt)

# p-value calculation

p <- cor.test(x, y)$p.value

txt2 <- format(c(p, 0.123456789), digits = digits)[1]

txt2 <- paste("p= ", txt2, sep = "")

if(p<0.01) txt2 <- paste("p= ", "<0.01", sep = "")

text(0.5, 0.3, txt2)

}

pairs(~cr+cu+zn+cd+dzn+eff+dflow,data=wangalldata,upper.panel=panel.cor,pch=20,na.action = na.omit)

dev.off()

##########Antimony and other factors

tiff(file="Antimony and other factors",width=14,height=14,units="in",pointsize = 12,bg ="transparent",res=800,compression="lzw")

par(mar=c(5,5,4,2),cex=0.9)

panel.cor <- function(x, y, digits = 2, cex.cor, ...)

{

usr <- par("usr"); on.exit(par(usr))

par(usr = c(0, 1, 0, 1))

# correlation coefficient

r <- cor(x, y)

method=c("spearman")

txt <- format(c(r, 0.123456789), digits = digits)[1]

txt <- paste("r= ", txt, sep = "")

text(0.5, 0.8, txt)

# p-value calculation

p <- cor.test(x, y)$p.value

txt2 <- format(c(p, 0.123456789), digits = digits)[1]

txt2 <- paste("p= ", txt2, sep = "")

if(p<0.01) txt2 <- paste("p= ", "<0.01", sep = "")

text(0.5, 0.3, txt2)

}

pairs(~sb+dsb+eff+dflow,data=wangalldata,upper.panel=panel.cor,pch=20,na.action = na.omit)

dev.off()

########Turbidity and other factors

tiff(file="Turbidity and other factors",width=14,height=14,units="in",pointsize = 12,bg ="transparent",res=800,compression="lzw")

par(mar=c(5,5,4,2),cex=0.9)

panel.cor <- function(x, y, digits = 2, cex.cor, ...)

{

usr <- par("usr"); on.exit(par(usr))

par(usr = c(0, 1, 0, 1))

# correlation coefficient

r <- cor(x, y)

method=c("spearman")

txt <- format(c(r, 0.123456789), digits = digits)[1]

txt <- paste("r= ", txt, sep = "")

text(0.5, 0.8, txt)

# p-value calculation

p <- cor.test(x, y)$p.value

txt2 <- format(c(p, 0.123456789), digits = digits)[1]

txt2 <- paste("p= ", txt2, sep = "")

if(p<0.01) txt2 <- paste("p= ", "<0.01", sep = "")

text(0.5, 0.3, txt2)

}

pairs(~turb+vel+eff+dflow+rain1+graz1000+ind100,data=alldata,upper.panel=panel.cor,pch=20,na.action = na.omit)

dev.off()

########sediment and other factors

tiff(file="Turbidity and other factors",width=14,height=14,units="in",pointsize = 12,bg ="transparent",res=800,compression="lzw")

par(mar=c(5,5,4,2),cex=0.9)

panel.cor <- function(x, y, digits = 2, cex.cor, ...)

{

usr <- par("usr"); on.exit(par(usr))

par(usr = c(0, 1, 0, 1))

# correlation coefficient

r <- cor(x, y)

method=c("spearman")

txt <- format(c(r, 0.123456789), digits = digits)[1]

txt <- paste("r= ", txt, sep = "")

text(0.5, 0.8, txt)

# p-value calculation

p <- cor.test(x, y)$p.value

txt2 <- format(c(p, 0.123456789), digits = digits)[1]

txt2 <- paste("p= ", txt2, sep = "")

if(p<0.01) txt2 <- paste("p= ", "<0.01", sep = "")

text(0.5, 0.3, txt2)

}

pairs(~sed+vel+eff+dflow,data=wangalldata,upper.panel=panel.cor,pch=20,na.action = na.omit)

dev.off()

###########Scatterplot Matrices/first two PCOs and environmental variables

tiff(file="first two PCOs and environmental variables.jpg",width=14,height=14,units="in",pointsize = 12,bg ="transparent",res=800,compression="lzw")

par(mar=c(5,5,4,2),cex=0.9)

panel.cor <- function(x, y, digits = 2, cex.cor, ...)

{

usr <- par("usr"); on.exit(par(usr))

par(usr = c(0, 1, 0, 1))

# correlation coefficient

r <- cor(x, y)

method=c("spearman")

txt <- format(c(r, 0.123456789), digits = digits)[1]

txt <- paste("r= ", txt, sep = "")

text(0.5, 0.8, txt)

# p-value calculation

p <- cor.test(x, y)$p.value

txt2 <- format(c(p, 0.123456789), digits = digits)[1]

txt2 <- paste("p= ", txt2, sep = "")

if(p<0.01) txt2 <- paste("p= ", "<0.01", sep = "")

text(0.5, 0.3, txt2)

}

pairs(~temp+cod+cfpom+chla+ph2+turb+sed+vel+alk+cond+no3+op+no2+nh3+tp+tn+on+pco1+pco2,data=alldata,upper.panel=panel.cor,pch=20,na.action = na.omit)

dev.off()

##

tiff(file="first two PCOs ,important variables",width=14,height=14,units="in",pointsize = 12,bg ="transparent",res=800,compression="lzw")

par(mar=c(5,5,4,2),cex=0.9)

panel.cor <- function(x, y, digits = 2, cex.cor, ...)

{

usr <- par("usr"); on.exit(par(usr))

par(usr = c(0, 1, 0, 1))

# correlation coefficient

r <- cor(x, y)

method=c("spearman")

txt <- format(c(r, 0.123456789), digits = digits)[1]

txt <- paste("r= ", txt, sep = "")

text(0.5, 0.8, txt)

# p-value calculation

p <- cor.test(x, y)$p.value

txt2 <- format(c(p, 0.123456789), digits = digits)[1]

txt2 <- paste("p= ", txt2, sep = "")

if(p<0.01) txt2 <- paste("p= ", "<0.01", sep = "")

text(0.5, 0.3, txt2)

}

pairs(~log(vel)+log(sali)+temp+log(chla)+log(do)+pco1+pco2,data=alldata,upper.panel=panel.cor,pch=20,na.action = na.omit)

dev.off()

####

tiff(file="first two PCOs , nutrients",width=14,height=14,units="in",pointsize = 12,bg ="transparent",res=800,compression="lzw")

par(mar=c(5,5,4,2),cex=0.9)

panel.cor <- function(x, y, digits = 2, cex.cor, ...)

{

usr <- par("usr"); on.exit(par(usr))

par(usr = c(0, 1, 0, 1))

# correlation coefficient

r <- cor(x, y)

method=c("spearman")

txt <- format(c(r, 0.123456789), digits = digits)[1]

txt <- paste("r= ", txt, sep = "")

text(0.5, 0.8, txt)

# p-value calculation

p <- cor.test(x, y)$p.value

txt2 <- format(c(p, 0.123456789), digits = digits)[1]

txt2 <- paste("p= ", txt2, sep = "")

if(p<0.01) txt2 <- paste("p= ", "<0.01", sep = "")

text(0.5, 0.3, txt2)

}

pairs(~alk+vel+sed+ph2+ph1+turb+sali+do+cod+toc+cfpom+temp+no3+no2+nh3+tp+tn+op+chla+cond+pco1+pco2,data=alldata,upper.panel=panel.cor,pch=20,na.action = na.omit)

dev.off()

##########Checking correlation between variables

edit(alldata)

plot(alldata$zn,alldata$pco1)

cor.test(alldata$zn,alldata$pco1)

plot(log10(alldata$zn),alldata$pco1)

cor.test(log10(alldata$zn),alldata$pco1)

plot(alldata$cu,alldata$pco1)

cor.test(alldata$cu,alldata$pco1)

plot(log(alldata$cu),alldata$pco1)

cor.test(log(alldata$cu),alldata$pco1)

plot(alldata$sed,alldata$pco1)

cor.test(alldata$sed,alldata$pco1)

plot(log(alldata$sed),alldata$pco1)

cor.test(log(alldata$sed),alldata$pco1)

plot(alldata$alk,alldata$pco1)

cor.test(alldata$alk,alldata$pco1)

plot(log(alldata$alk),alldata$pco1)

cor.test(log(alldata$vel),alldata$pco1)

plot(alldata$cod,alldata$pco1)

cor.test(alldata$cod,alldata$pco1)

plot(alldata$cod[alldata$cod<30],alldata$pco1[alldata$cod<30])

cor.test(alldata$cod[alldata$cod<30],alldata$pco1[alldata$cod<30])

plot(log(alldata$cod[alldata$cod<30]),alldata$pco1[alldata$cod<30])

cor.test(log(alldata$cod[alldata$cod<30]),alldata$pco1[alldata$cod<30])

plot(alldata$toc,alldata$pco1)

cor.test(alldata$toc,alldata$pco1)

plot(alldata$temp,alldata$pco1)

cor.test(alldata$temp,alldata$pco1)

plot(log(alldata$temp),alldata$pco1)

cor.test(log(alldata$temp),alldata$pco1)

plot(alldata$sali,alldata$pco1)

cor.test(alldata$sali,alldata$pco1)

plot(alldata$sali[alldata$sali<0.4],alldata$pco1[alldata$sali<0.4])

cor.test(alldata$sali[alldata$sali<0.4],alldata$pco1[alldata$sali<0.4])

plot(log(alldata$sali[alldata$sali<0.4]),alldata$pco1[alldata$sali<0.4])

cor.test(log(alldata$sali[alldata$sali<0.4]),alldata$pco1[alldata$sali<0.4])

plot(alldata$cfpom,alldata$pco1)

cor.test(alldata$cfpom,alldata$pco1)

plot(alldata$cfpom[alldata$cfpom<25],alldata$pco1[alldata$cfpom<25])

cor.test(alldata$cfpom[alldata$cfpom<25],alldata$pco1[alldata$cfpom<25])

plot(log(alldata$cfpom[alldata$cfpom<25]),alldata$pco1[alldata$cfpom<25])

cor.test(log(alldata$cfpom[alldata$cfpom<25]),alldata$pco1[alldata$cfpom<25])

plot(alldata$turb,alldata$pco1)

cor.test(alldata$turb,alldata$pco1)

plot(log(alldata$turb),alldata$pco1)

cor.test(log(alldata$turb),alldata$pco1)

plot(alldata$no3,alldata$pco1)

cor.test(alldata$no3,alldata$pco1)

plot(alldata$no3[alldata$no3<0.2],alldata$pco1[alldata$no3<0.2])

cor.test(alldata$no3[alldata$no3<0.2],alldata$pco1[alldata$no3<0.2])

plot(log1p(alldata$no3[alldata$no3<0.2]),alldata$pco1[alldata$no3<0.2])

cor.test(log1p(alldata$no3[alldata$no3<0.2]),alldata$pco1[alldata$no3<0.2])

plot(alldata$do,alldata$pco2)

cor.test(alldata$do,alldata$pco2)

plot(sqrt(alldata$do),alldata$pco1)

cor.test(sqrt(alldata$do),alldata$pco1)

plot(alldata$nh3,alldata$pco1)

cor.test(alldata$nh3,alldata$pco1)

plot(log1p(alldata$nh3),alldata$pco1)

cor.test(log1p(alldata$nh3),alldata$pco1)

plot(alldata$op,alldata$pco1)

cor.test(alldata$op,alldata$pco1)

plot(log1p(alldata$op),alldata$pco1)

cor.test(log1p(alldata$op),alldata$pco1)

plot(alldata$tp,alldata$pco1)

cor.test(alldata$tp,alldata$pco1)

plot(log(alldata$tp),alldata$pco1)

cor.test(log(alldata$tp),alldata$pco1)

plot(sqrt(alldata$tp),alldata$pco1)

cor.test(sqrt(alldata$tp),alldata$pco1)

plot(alldata$chla,alldata$pco1)

cor.test(alldata$chla,alldata$pco1)

plot(log(alldata$chla),alldata$pco1)

cor.test(log(alldata$chla),alldata$pco1)

plot(alldata$cond,alldata$pco1)

cor.test(alldata$cond,alldata$pco1)

plot(log(alldata$cond),alldata$pco1)

cor.test(log(alldata$cond),alldata$pco1)

plot(alldata$ph1,alldata$pco1)

cor.test(alldata$ph1,alldata$pco1)

plot(log(alldata$ph1),alldata$pco1)

cor.test(log(alldata$ph1),alldata$pco1)

plot(alldata$ph2,alldata$pco1)

cor.test(alldata$ph2,alldata$pco1)

plot(log(alldata$ph2),alldata$pco1)

cor.test(log(alldata$ph2),alldata$pco1)

plot(alldata$do,alldata$pco1)

cor.test(alldata$do,alldata$pco1)

plot(log(alldata$do),alldata$pco1)

cor.test(log(alldata$do),alldata$pco1)

plot(alldata$pred2,alldata$pco2)

cor.test(alldata$pred1,alldata$pco1)

plot(alldata$cod,alldata$toc)

identify(alldata$cod,alldata$toc)

cor.test(alldata$cod,alldata$toc)

plot(alldata$cr,alldata$pc2)

cor.test(alldata$cr,alldata$pco2)

plot(log(alldata$tn),alldata$pco1)

cor.test(log(alldata$tn),alldata$pco1)

plot(alldata$no2,alldata$pco1)

cor.test(alldata$no2,alldata$pco1)

plot(alldata$alk,alldata$pco1)

cor.test(alldata$alk,alldata$pco1)

plot(log(alldata$alk),alldata$pco1)

cor.test(log(alldata$alk),alldata$pco1)

plot(alldata$turb,alldata$toc)

cor.test(alldata$turb,alldata$toc)

plot(alldata$tp,alldata$ind1000)

cor.test(alldata$tp,alldata$ind1000)

plot(log(alldata$tp),alldata$ind1000)

cor.test(log(alldata$tp),alldata$ind1000)

plot(alldata$dayflow,alldata$chla)

cor.test(alldata$dayflow,alldata$chla)

plot(alldata$dayflow,log(alldata$chla))

cor.test(alldata$dayflow,log(alldata$chla))

plot(alldata$dflow,alldata$dayflow)

cor.test(alldata$dflow,alldata$dayflow)

plot(alldata$dayflow,log(alldata$chla))

cor.test(alldata$dayflow,log(alldata$chla))

###########Testing the variables before dbRDA --PCO1

#### pco1 as a function of environmental variables

library(mass)

wangenvOutliers<-read.csv("wangenvOutliers.csv",header=TRUE,nrows=40)

bugenv1<-cbind(wangenvOutliers,pwangbug)

summary(tempo)

edit(alldata)

tempo<-lm(pco1~temp,data=alldata)

tempo<-lm(pco1~cond,data=alldata)

tempo<-lm(pco1~temp+cond+temp:cond,data=alldata)

tempo<-lm(pco1~log(cr),data=alldata)

tempo<-lm(pco1~temp+cr+temp:cr,data=alldata)

tempo<-lm(pco1~temp+cond+temp:cond+cr+temp:cr,data=alldata)

tempo<-lm(pco1~log(zn),data=alldata)

tempo<-lm(pco1~temp+log(zn)+log(zn):temp,data=alldata)

tempo<-lm(pco1~temp+cond+temp:cond+log(zn)+log(zn):temp,data=alldata)

tempo<-lm(pco1~log(cd),data=alldata)

tempo<-lm(pco1~temp+log(cd)+log(cd):temp,data=alldata)

tempo<-lm(pco1~temp+cond+temp:cond+cd+cd:temp,data=alldata)

tempo<-lm(pco1~cu,data=alldata)

tempo<-lm(pco1~temp+cu+cu:temp,data=alldata)

tempo<-lm(pco1~temp+cond+temp:cond+cu+cu:temp,data=alldata)

tempo<-lm(pco1~log(chla),data=alldata)

tempo<-lm(pco1~temp+log(chla)+log(chla):temp,data=alldata)

tempo<-lm(pco1~temp+cond+temp:cond+log(chla)+temp:log(chla),data=alldata)

tempo<-lm(pco1~do,data=alldata)

tempo<-lm(pco1~temp+do+do:temp,data=alldata)

tempo<-lm(pco1~temp+cond+temp:cond+do+do:temp,data=alldata)

tempo<-lm(pco1~cfpom,data=alldata)

tempo<-lm(pco1~cfpom+temp+temp:cfpom,data=alldata)

tempo<-lm(pco1~temp+cond+temp:cond+cfpom+cfpom:temp,data=alldata)

tempo<-lm(pco1~cod,data=bugenv1)

tempo<-lm(pco1~cod+temp+temp:cod,data=alldata)

tempo<-lm(pco1~temp+cond+temp:cond+cod+cod:temp,data=alldata)

tempo<-lm(pco1~turb,data=alldata)

tempo<-lm(pco1~temp+turb+turb:temp,data=alldata)

tempo<-lm(pco1~temp+cond+temp:cond+turb+turb:temp,data=alldata)

tempo<-lm(pco1~vel,data=alldata)

tempo<-lm(pco1~temp+vel+vel:temp,data=alldata)

tempo<-lm(pco1~temp+cond+temp:cond+vel+vel:temp,data=alldata)

tempo<-lm(pco1~nh3,data=alldata)

tempo<-lm(pco1~temp+nh3+nh3:temp,data=alldata)

tempo<-lm(pco1~temp+cond+temp:cond+nh3+nh3:temp,data=alldata)

tempo<-lm(pco1~toc,data=alldata)

tempo<-lm(pco1~temp+toc+toc:temp,data=alldata)

tempo<-lm(pco1~temp+cond+temp:cond+toc+toc:temp,data=alldata)

tempo<-lm(pco1~ph2,data=alldata)

tempo<-lm(pco1~temp+ph2+ph2:temp,data=alldata)

tempo<-lm(pco1~temp+cond+temp:cond+ph2+ph2:temp,data=alldata)

tempo<-lm(pco1~sed,data=alldata)

tempo<-lm(pco1~temp+sed+sed:temp,data=alldata)

tempo<-lm(pco1~on,data=alldata)

tempo<-lm(pco1~temp+on+on:temp,data=alldata)

## final models

tempo<-lm(pco1~log(cd),data=alldata)###Adjusted R-squared: 0.1135

tempo<-lm(pco1~log(chla),data=alldata)###chla--->Adjusted R-squared: 0.1301

tempo<-lm(pco1~log(zn),data=alldata)###zn--->Adjusted R-squared: 0.1044

tempo<-lm(pco1~temp,data=alldata)##temp---->Adjusted R-squared: 0.3996

tempo<-lm(pco1~temp+cod+temp:cod,data=alldata)##temp:cod---> Adjusted R-squared: 0.5219

tempo<-lm(pco1~temp+cond+temp:cond,data=alldata)##temp:cond---> Adjusted R-squared: 0.4243

tempo<-lm(pco1~temp+toc+temp:toc,data=alldata)##temp:toc---> Adjusted R-squared: 0.4826

tempo<-lm(pco1~temp+log(zn)+temp:log(zn),data=alldata)##temp:zn---> Adjusted R-squared: 0.5448

tempo<-lm(pco1~temp+cond+temp:cond+cod+temp:cod,data=alldata)##temp:cod, temp:cond---> Adjusted R-squared: 0.4966

tempo<-lm(pco1~temp+cod+temp:cod+log(zn)+temp:log(zn),data=alldata)#temp:cod,temp:zn --->Adjusted R-squared: 0.6461

tempo<-lm(pco1~temp+cod+temp:cod+log(zn)+temp:log(zn)+log(chla),data=alldata)#temp:cod,temp:zn, chla --->Adjusted R-squared: 0.6355

tempo<-lm(pco1~temp+cond+temp:cond+cod+temp:cod+log(zn)+temp:log(zn),data=alldata)#temp:cond,temp:cod,temp:zn --->Adjusted R-squared: 0.6613

tempo<-lm(pco1~temp+cod+temp:cod+log(zn),data=alldata)##Adjusted R-squared: 0.6254

tempo<-lm(pco1~temp+cond+temp:cond+cod+temp:cod+log(zn)+temp:log(zn)+log(chla)+log(cd),data=alldata)

tempo<-lm(pco1~temp+cond+toc+temp:cond+log(zn)+temp:toc+log(chla)+log(cr),data=alldata)##final model ##

tempo1<-lm(pco1~temp+cond+log(zn),data=alldata)##final model

anova(tempo, tempo1)

summary(tempo)##

anova(tempo)

avPlots(tempo)

tempo1<-cbind(cbind(fitted(tempo),residuals(tempo)),alldata[complete.cases(alldata[,c("temp","cond","chla","cod")]),])

attributes(tempo1)

names(tempo1[,1:2])<-c("fitted","residual")

edit(tempo1)

tempo1[,"2"]

tempo2<-lm(tempo1[,"2"]~pred1,data=tempo1)

summary(tempo2)

par(mar=c(5,5,4,2),cex=0.9)

plot(tempo1[tempo1$day==126,"dist"],tempo1[tempo1$day==126,"1"],type = "b",ylim=c(-.6,0.6),pch=19,col="gold2",cex=1.5,xlab="Distance(km)",ylab="PCO1",main="Prediction with Zinc, Chromium, Chlorophyll A, Temperature, TOC, Conductivity,\n Temperature*TOC, and Temperature*Conductivity interaction",lwd=3,lty=1)

#points(tempo1[tempo1$day==126,"dist"],tempo1[tempo1$day==126,"1"],type="b",col="gold2",pch=15,lwd=3,cex=1.5,lty=2)

points(tempo1[tempo1$day==260,"dist"],tempo1[tempo1$day==260,"1"],type="b",col="blue",pch=8,cex=1.5,lwd=3,lty=3)

points(tempo1[tempo1$day==336,"dist"],tempo1[tempo1$day==336,"1"],type="b",col="green3",pch=17,cex=1.5,lwd=3,lty=4)

points(tempo1[tempo1$day==518,"dist"],tempo1[tempo1$day==518,"1"],type="b",col="red",pch=18,cex=2,lwd=3,lty=5)

legend("topright",inset=c(0,0),legend=c("Dec 13", "April 14", "Aug 2014", "Nov 2014","May 2015"),lty=c(1,5),pch=c(19,15,8,17,18),lwd=2,col=c("black","gold2","blue","green3","red"),ncol=2,horiz=FALSE,cex=0.8,title="months")

abline(v=4,lty=2)

xyplot(fitted(tempo)~dist, groups=day,data=tempo1,type="l",auto.key=TRUE)

xyplot(residuals(tempo)~dist, groups=day,data=tempo1,type="l",auto.key=TRUE)

tempo3<-lm(tempo1[,2]~tempo1$pred1)

summary(tempo3)

#***** Figure 4 *****#

tiff(file="Figure 4.jpg",width=10,height=14,units="in",pointsize = 12,bg ="transparent",res=800,compression="lzw")

par(mfrow=c(3,1), mar=c(4.5,4.5,2.5,3),cex=1.5,cex.axis=0.9,las=1,cex.main=1,cex.lab=0.8)

## pco plot against distance

plot(bugenv[bugenv$day==1,"dist"],bugenv[bugenv$day==1,"pco1"],type = "b",ylim=c(-.6,1),pch=19,xlab="",ylab="PCO1",main="a) PCO1 plotted against spatial position",lwd=3,lty=1)

points(bugenv[bugenv$day==126,"dist"],bugenv[bugenv$day==126,"pco1"],type="b",col="gold2",pch=15,lwd=3,cex=1.2,lty=2)

points(bugenv[bugenv$day==260,"dist"],bugenv[bugenv$day==260,"pco1"],type="b",col="blue",pch=8,cex=1.2,lwd=3,lty=3)

points(bugenv[bugenv$day==336,"dist"],bugenv[bugenv$day==336,"pco1"],type="b",col="green3",pch=17,cex=1.2,lwd=3,lty=4)

points(bugenv[bugenv$day==518,"dist"],bugenv[bugenv$day==518,"pco1"],type="b",col="red",pch=18,cex=1.4,lwd=3,lty=5)

legend("topright",inset=c(0,0),legend=c("Dec 13", "April 14", "Aug 2014", "Nov 2014","May 2015"),lty=c(1,5),pch=c(19,15,8,17,18),lwd=2,col=c("black","gold2","blue","green3","red"),ncol=2,horiz=FALSE,cex=0.6,title="months")

abline(v=4,lty=2)

### predicted pcos

num<-1

#fig.name<-"wangpco1.tif"

pco.no<-paste("pco",num,sep="")

PCO.NO<-paste("PCO",num," (",pco.varpercent[num],"% of total variation)",sep="")

pred.no<-paste("pred",num,sep="")

#tiff(file=fig.name,width=7,height=5,units="in",pointsize = 12,bg = "transparent",res=300,compression="lzw")

#par(mar=c(5,5,4,2),cex=0.9)

plot(wangenv$dist[wangenv$time==1],pwangbug[wangenv$time==1,pco.no],type="p",pch=19,col="black",xlab="",ylab=PCO.NO,ylim=c(-.6,1),main="b) PCO1 with spatiotemporal model predictions",cex=1,lwd=3,lty=1)

points(wangenv$dist[wangenv$time==1],pwangbug[wangenv$time==2,pco.no],col="gold2",pch=15,lwd=3,cex=1.2,lty=2)

points(wangenv$dist[wangenv$time==1],pwangbug[wangenv$time==3,pco.no],col="blue",pch=8, cex=1.2,lwd=3,lty=3)

points(wangenv$dist[wangenv$time==1],pwangbug[wangenv$time==4,pco.no],col="green3",pch=17,cex=1.2,lwd=3,lty=4)

points(wangenv$dist[wangenv$time==1],pwangbug[wangenv$time==5,pco.no],col="red",pch=18,cex=1.4,lwd=3,lty=5)

lines(xygrid$dist[xygrid$time==1],pco.predict2[pco.predict2$time==1,pred.no],type="l",lty=1,col="black",lwd=2)

lines(xygrid$dist[xygrid$time==1],pco.predict2[pco.predict2$time==2,pred.no],type="l",lty=2,col="gold2",lwd=2)

lines(xygrid$dist[xygrid$time==1],pco.predict2[pco.predict2$time==3,pred.no],type="l",lty=3,col="blue",lwd=2)

lines(xygrid$dist[xygrid$time==1],pco.predict2[pco.predict2$time==4,pred.no],type="l",lty=4,col="green3",lwd=2)

lines(xygrid$dist[xygrid$time==1],pco.predict2[pco.predict2$time==5,pred.no],type="l",lty=5,col="red",lwd=2)

legend("topright",inset=c(0,0),legend=c("Dec 2013", "April 2014", "Aug 2014", "Nov 2014","May 2015"),lty=c(1,5),pch=c(19,15,8,17,18),lwd=2,col=c("black","gold2","blue","green3","red"),

ncol=2,horiz=FALSE,cex=0.6,title="months")

## pco plot for fitted values

tempo<-lm(pco1~temp+cond+toc+temp:cond+log(zn)+temp:toc+log(chla)+log(cr),bugenv)#[complete.cases(bugenv[,c("temp","no3","tp")]),])

tempo1<-cbind(cbind(fitted(tempo),residuals(tempo)),alldata[complete.cases(alldata[,c("temp","cond")]),])

attributes(tempo1)

names(tempo1[,1:2])<-c("fitted","residual")

plot(tempo1[tempo1$day==126,"dist"],tempo1[tempo1$day==126,"1"],type = "l",ylim=c(-.6,1),pch=19,col="gold2",xlab="Distance(km)",ylab="PCO1",main="c) Prediction with zinc, chlorophyll A, temperature, TOC, conductivity,\n temperature*TOC, and temperature*Conductivity interaction",lwd=3,lty=2)

#points(tempo1[tempo1$day==126,"dist"],tempo1[tempo1$day==126,"1"],type="l",col="gold2",pch=15,lwd=3,cex=1.5,lty=2)

points(tempo1[tempo1$day==260,"dist"],tempo1[tempo1$day==260,"1"],type="l",col="blue",pch=8,cex=1.2,lwd=3,lty=3)

points(tempo1[tempo1$day==336,"dist"],tempo1[tempo1$day==336,"1"],type="l",col="green3",pch=17,cex=1.2,lwd=3,lty=4)

points(tempo1[tempo1$day==518,"dist"],tempo1[tempo1$day==518,"1"],type="l",col="red",pch=18,cex=1.4,lwd=3,lty=5)

points(bugenv[bugenv$day==1,"dist"],bugenv[bugenv$day==1,"pco1"],type = "p",ylim=c(-.6,1),pch=19,xlab="",ylab="PCO1",main="a) PCO1 plotted against spatial position",lwd=3,lty=1)

points(bugenv[bugenv$day==126,"dist"],bugenv[bugenv$day==126,"pco1"],type="p",col="gold2",pch=15,lwd=3,cex=1.2,lty=2)

points(bugenv[bugenv$day==260,"dist"],bugenv[bugenv$day==260,"pco1"],type="p",col="blue",pch=8,cex=1.2,lwd=3,lty=3)

points(bugenv[bugenv$day==336,"dist"],bugenv[bugenv$day==336,"pco1"],type="p",col="green3",pch=17,cex=1.2,lwd=3,lty=4)

points(bugenv[bugenv$day==518,"dist"],bugenv[bugenv$day==518,"pco1"],type="p",col="red",pch=18,cex=1.4,lwd=3,lty=5)

legend("topright",inset=c(0,0),legend=c("Dec 13", "April 14", "Aug 2014", "Nov 2014","May 2015"),lty=c(1,5),pch=c(19,15,8,17,18),lwd=2,col=c("black","gold2","blue","green3","red"),ncol=2,horiz=FALSE,cex=0.6,title="months")

abline(v=4,lty=2)

dev.off()

########### scatterplot for pco1 and res1 against physicochemical variables

par(mar=c(5,5,4,2),cex=0.9)

panel.cor <- function(x, y, digits = 2, cex.cor, ...)

{

usr <- par("usr"); on.exit(par(usr))

par(usr = c(0, 1, 0, 1))

# correlation coefficient

r <- cor(x, y)

method=c("spearman")

txt <- format(c(r, 0.123456789), digits = digits)[1]

txt <- paste("r= ", txt, sep = "")

text(0.5, 0.8, txt)

# p-value calculation

p <- cor.test(x, y)$p.value

txt2 <- format(c(p, 0.123456789), digits = digits)[1]

txt2 <- paste("p= ", txt2, sep = "")

if(p<0.01) txt2 <- paste("p= ", "<0.01", sep = "")

text(0.5, 0.3, txt2)

}

pairs(pco1~do+log(cr)+log(toc)+cod+log(cfpom)+log(chla)+log(turb)+log(vel)+log(temp)+log(ph2)+log(cond)+log(do)+log(sb)+log(cu)+log(cr)+log(cd)+log(zn)+sed,data=alldata,upper.panel=panel.cor,pch=20,na.action = na.omit)

#######

par(mar=c(5,5,4,2),cex=0.9)

panel.cor <- function(x, y, digits = 2, cex.cor, ...)

{

usr <- par("usr"); on.exit(par(usr))

par(usr = c(0, 1, 0, 1))

# correlation coefficient

r <- cor(x, y)

method=c("spearman")

txt <- format(c(r, 0.123456789), digits = digits)[1]

txt <- paste("r= ", txt, sep = "")

text(0.5, 0.8, txt)

# p-value calculation

p <- cor.test(x, y)$p.value

txt2 <- format(c(p, 0.123456789), digits = digits)[1]

txt2 <- paste("p= ", txt2, sep = "")

if(p<0.01) txt2 <- paste("p= ", "<0.01", sep = "")

text(0.5, 0.3, txt2)

}

pairs(res1~ph2+log(chla)+do+cfpom+nh3+cod+turb+vel+cd+log(zn)+log(cu)+cr+temp+sed+cond+toc,data=envpcores,upper.panel=panel.cor,pch=20,na.action = na.omit)

edit(envpcores)

##Testing the variables before dbRDA --PCO2

wangenvOutliers<-read.csv("wangenvOutliers.csv",header=TRUE,nrows=40)

bugenv1<-cbind(wangenvOutliers,pwangbug)

edit(alldata)

summary(tempo)

tempo<-lm(pco2~temp,data=alldata)

tempo<-lm(pco2~cond,data=alldata)

tempo<-lm(pco2~temp+cond+temp:cond,data=alldata)

tempo<-lm(pco2~cr,data=alldata)

tempo<-lm(pco2~temp+cr+temp:cr,data=alldata)

tempo<-lm(pco2~temp+cond+temp:cond+cr+temp:cr,data=alldata)

tempo<-lm(pco2~log(zn),data=alldata)

tempo<-lm(pco2~temp+log(zn)+log(zn):temp,data=alldata)

tempo<-lm(pco2~temp+cond+temp:cond+log(zn)+log(zn):temp,data=alldata)

tempo<-lm(pco2~cd,data=alldata)

tempo<-lm(pco2~temp+cd+cd:temp,data=alldata)

tempo<-lm(pco2~temp+cond+temp:cond+cd+cd:temp,data=alldata)

tempo<-lm(pco2~log(cu),data=alldata)

tempo<-lm(pco2~temp+log(cu)+log(cu):temp,data=alldata)

tempo<-lm(pco2~temp+cond+temp:cond+cu+cu:temp,data=alldata)

tempo<-lm(pco2~log(chla),data=alldata)

tempo<-lm(pco2~temp+log(chla)+log(chla):temp,data=alldata)

tempo<-lm(pco2~temp+cond+temp:cond+log(chla)+temp:log(chla),data=alldata)

tempo<-lm(pco2~do,data=alldata)

tempo<-lm(pco2~temp+do+do:temp,data=alldata)

tempo<-lm(pco2~temp+cond+temp:cond+do+do:temp,data=alldata)

tempo<-lm(pco2~cfpom,data=alldata)

tempo<-lm(pco2~cfpom+temp+temp:cfpom,data=alldata)

tempo<-lm(pco2~temp+cond+temp:cond+cfpom+cfpom:temp,data=alldata)

tempo<-lm(pco2~cod,data=bugenv1)

tempo<-lm(pco2~cod+temp+temp:cod,data=alldata)

tempo<-lm(pco2~temp+cond+temp:cond+cod+cod:temp,data=alldata)

tempo<-lm(pco2~turb,data=alldata)

tempo<-lm(pco2~temp+turb+turb:temp,data=alldata)

tempo<-lm(pco2~temp+cond+temp:cond+turb+turb:temp,data=alldata)

tempo<-lm(pco2~vel,data=alldata)

tempo<-lm(pco2~temp+vel+vel:temp,data=alldata)

tempo<-lm(pco2~temp+cond+temp:cond+vel+vel:temp,data=alldata)

tempo<-lm(pco2~nh3,data=alldata)

tempo<-lm(pco2~temp+nh3+nh3:temp,data=alldata)

tempo<-lm(pco2~temp+cond+temp:cond+nh3+nh3:temp,data=alldata)

tempo<-lm(pco2~toc,data=alldata)

tempo<-lm(pco2~temp+toc+toc:temp,data=alldata)

tempo<-lm(pco2~temp+cond+temp:cond+toc+toc:temp,data=alldata)

tempo<-lm(pco2~ph2,data=alldata)

tempo<-lm(pco2~temp+ph2+ph2:temp,data=alldata)

tempo<-lm(pco2~temp+cond+temp:cond+ph2+ph2:temp,data=alldata)

tempo<-lm(pco2~temp+ph2+ph2:temp,data=alldata)

tempo<-lm(pco2~temp+cond+temp:cond+ph2+ph2:temp,data=alldata)

tempo<-lm(pco2~sed,data=alldata)

tempo<-lm(pco2~temp+sed+sed:temp,data=alldata)

## final models for pco2

tempo<-lm(pco2~toc,data=alldata)##Adjusted R-squared: 0.1325

tempo<-lm(pco2~cr,data=alldata)##Adjusted R-squared: 0.1155

tempo<-lm(pco2~log(cu),data=alldata)##Adjusted R-squared: 0.07606

tempo<-lm(pco2~turb,data=alldata)##Adjusted R-squared: 0.3089

tempo<-lm(pco2~vel,data=alldata)

tempo<-lm(pco2~temp+ph2+temp:ph2,data=alldata)##Adjusted R-squared: 0.1524

tempo<-lm(pco2~temp+nh3+temp:nh3,data=alldata)##Adjusted R-squared: 0.2765

tempo<-lm(pco2~temp+log(chla)+temp:log(chla),data=alldata)##Adjusted R-squared: 0.06501

tempo<-lm(pco2~temp+vel+temp:vel,data=alldata)##Adjusted R-squared: 0.1899

tempo<-lm(pco2~nh3+temp:nh3+temp+ph2:temp+ph2+log(chla)+temp:log(chla)+vel+temp:vel+toc+turb+log(cu),data=alldata)##Adjusted R-squared: 0.4439

tempo<-lm(pco2~turb+log(chla)+temp:log(chla)+temp+ph2:temp+ph2,data=alldata)

tempo<-lm(pco2~turb+temp+nh3+temp:nh3+ph2:temp+ph2,data=alldata)

summary(tempo)

tempo2<-cbind(cbind(fitted(tempo),residuals(tempo)),alldata[complete.cases(alldata[,c("temp","nh3","turb","toc","ph2","vel","chla","cu","cr")]),])

attributes(tempo2)

names(tempo2[,1:2])<-c("fitted","residual")

edit(tempo2)

tempo2[,"2"]

tempo3<-lm(tempo2[,"2"]~pred2,data=tempo2)

summary(tempo3)

par(mar=c(5,5,4,2),cex=0.9)

plot(tempo2[tempo2$day==126,"dist"],tempo2[tempo2$day==126,"1"],type = "b",ylim=c(-0.6,0.6),pch=19,cex=1.5,xlab="Distance(km)",ylab="PCO2",main="prediction with other variables interaction",col="gold2",lwd=3,lty=4)

points(tempo2[tempo2$day==260,"dist"],tempo2[tempo2$day==260,"1"],type="b",col="blue",pch=8,cex=1.5,lwd=3,lty=3)

points(tempo2[tempo2$day==336,"dist"],tempo2[tempo2$day==336,"1"],type="b",col="green3",pch=17,cex=1.5,lwd=3)

points(tempo2[tempo2$day==518,"dist"],tempo2[tempo2$day==518,"1"],type="b",col="red",pch=18,cex=2,lwd=3,lty=5)

legend("topright",inset=c(0,0),legend=c("April 2014", "Aug 2014", "Nov 2014","May 2015"),lty=1,lwd=2,col=c("gold2","blue","green3","red"),ncol=4,horiz=FALSE,cex=0.7,title="months")

abline(v=4,lty=2)

par(mar=c(5,5,4,2),cex=0.9)

plot(tempo2[tempo2$day==126,"dist"],tempo2[tempo2$day==126,"2"],type = "b",ylim=c(-0.6,0.6),pch=19,cex=1.5,xlab="Distance(km)",ylab="PCO2",main="prediction with other variables interaction",col="gold2",lwd=3,lty=4)

points(tempo2[tempo2$day==260,"dist"],tempo2[tempo2$day==260,"2"],type="b",col="blue",pch=8,cex=1.5,lwd=3,lty=3)

points(tempo2[tempo2$day==336,"dist"],tempo2[tempo2$day==336,"2"],type="b",col="green3",pch=17,cex=1.5,lwd=3)

points(tempo2[tempo2$day==518,"dist"],tempo2[tempo2$day==518,"2"],type="b",col="red",pch=18,cex=2,lwd=3,lty=5)

legend("topright",inset=c(0,0),legend=c("April 2014", "Aug 2014", "Nov 2014","May 2015"),lty=1,lwd=2,col=c("gold2","blue","green3","red"),ncol=4,horiz=FALSE,cex=0.7,title="months")

abline(v=4,lty=2)

xyplot(fitted(tempo)~dist, groups=day,data=tempo2,type="l",auto.key=TRUE)

xyplot(residuals(tempo)~dist, groups=day,data=tempo2,type="l",auto.key=TRUE)

tempo3<-lm(tempo2[,2]~tempo2$pred2)

summary(tempo3)

plot(alldata$ph2~alldata$pco2)

cor.test(alldata$ph2,alldata$pco2)

plot(log(alldata$cu),alldata$pco2)

cor.test(log(alldata$cu),alldata$pco2)

##Testing the variables before dbRDA --PCO3

edit(alldata)

summary(tempo)

tempo<-lm(pco3~log(temp),data=alldata)

tempo<-lm(pco3~cond,data=alldata)

tempo<-lm(pco3~log(temp)+cond+log(temp):cond,data=alldata)

tempo<-lm(pco3~cr,data=alldata)

tempo<-lm(pco3~temp+cr+temp:cr,data=alldata)

tempo<-lm(pco3~temp+cond+temp:cond+cr+temp:cr,data=alldata)

tempo<-lm(pco3~zn,data=alldata)

tempo<-lm(pco3~temp+zn+zn:temp,data=alldata)

tempo<-lm(pco3~temp+cond+temp:cond+zn+zn:temp,data=alldata)

tempo<-lm(pco3~cd,data=alldata)

tempo<-lm(pco3~temp+cd+cd:temp,data=alldata)

tempo<-lm(pco3~temp+cond+temp:cond+cd+cd:temp,data=alldata)

tempo<-lm(pco3~cu,data=alldata)

tempo<-lm(pco3~temp+cu+cu:temp,data=alldata)

tempo<-lm(pco3~temp+cond+temp:cond+cu+cu:temp,data=alldata)

tempo<-lm(pco3~log(chla),data=alldata)

tempo<-lm(pco3~temp+log(chla)+log(chla):temp,data=alldata)

tempo<-lm(pco3~temp+cond+temp:cond+log(chla)+temp:log(chla),data=alldata)

tempo<-lm(pco3~do,data=alldata)

tempo<-lm(pco3~temp+do+do:temp,data=alldata)

tempo<-lm(pco3~temp+cond+temp:cond+do+do:temp,data=alldata)

tempo<-lm(pco3~cfpom,data=alldata)

tempo<-lm(pco3~cfpom+temp+temp:cfpom,data=alldata)

tempo<-lm(pco3~temp+cond+temp:cond+cfpom+cfpom:temp,data=alldata)

tempo<-lm(pco3~cod,data=alldata)

tempo<-lm(pco3~cod+temp+temp:cod,data=alldata)

tempo<-lm(pco3~temp+cond+temp:cond+cod+cod:temp,data=alldata)

tempo<-lm(pco3~turb,data=alldata)

tempo<-lm(pco3~temp+turb+turb:temp,data=alldata)

tempo<-lm(pco3~temp+cond+temp:cond+turb+turb:temp,data=alldata)

tempo<-lm(pco3~vel,data=alldata)

tempo<-lm(pco3~temp+vel+vel:temp,data=alldata)

tempo<-lm(pco3~temp+cond+temp:cond+vel+vel:temp,data=alldata)

tempo<-lm(pco3~nh3,data=alldata)

tempo<-lm(pco3~temp+nh3+nh3:temp,data=alldata)

tempo<-lm(pco3~temp+cond+temp:cond+nh3+nh3:temp,data=alldata)

tempo<-lm(pco3~toc,data=alldata)

tempo<-lm(pco3~temp+toc+toc:temp,data=alldata)

tempo<-lm(pco3~temp+cond+temp:cond+toc+toc:temp,data=alldata)

tempo<-lm(pco3~ph2,data=alldata)

tempo<-lm(pco3~temp+ph2+ph2:temp,data=alldata)

tempo<-lm(pco3~temp+cond+temp:cond+ph2+ph2:temp,data=alldata)

tempo<-lm(pco3~sed,data=alldata)

tempo<-lm(pco3~temp+sed+sed:temp,data=alldata)

## final models for pco3

tempo<-lm(pco3~temp+toc+temp:toc,data=alldata)##Adjusted R-squared: 0.2948

tempo<-lm(pco3~do,data=alldata)##Adjusted R-squared: 0.2875

tempo<-lm(pco3~temp+turb+temp:turb,data=alldata)##Adjusted R-squared: 0.2131

tempo<-lm(pco3~log(chla),data=alldata)##Adjusted R-squared: 0.2117

tempo<-lm(pco3~nh3,data=alldata)##Adjusted R-squared: 0.1153

tempo<-lm(pco3~vel,data=alldata)##Adjusted R-squared: 0.1092

tempo<-lm(pco3~log(temp),data=alldata)## Adjusted R-squared: 0.07468

tempo<-lm(pco3~temp+temp:toc+toc+do+turb+temp:turb,data=alldata)## Adjusted R-squared: 0.3031

tempo<-lm(pco3~temp+temp:toc+toc+do+turb+temp:turb+log(chla),data=alldata)## Adjusted R-squared: 0.2738

tempo<-lm(pco3~temp+temp:toc+toc+do+turb+temp:turb+log(chla)+nh3+vel+log(temp),data=alldata)## Adjusted R-squared: 0.2199

tempo<-lm(pco3~temp+temp:toc+toc+turb+temp:turb+log(chla)+nh3+vel+log(temp),data=alldata)## Adjusted R-squared: 0.2596

tempo<-lm(pco3~temp+temp:toc+toc+turb+temp:turb+log(chla)+vel+log(temp),data=alldata)## Adjusted R-squared: 0.317

summary(tempo)

tempo3<-cbind(cbind(fitted(tempo),residuals(tempo)),alldata[complete.cases(alldata[,c("temp","toc","turb","vel")]),])

tempo3<-cbind(cbind(fitted(tempo),residuals(tempo)),alldata[complete.cases(alldata[,c("temp","toc","turb","nh3","vel")]),])

attributes(tempo3)

names(tempo3[,1:2])<-c("fitted","residual")

edit(tempo3)

tempo3[,"2"]

tempo4<-lm(tempo3[,"2"]~pred3,data=tempo3)

summary(tempo4)

par(mar=c(5,5,4,2),cex=0.9)

plot(tempo3[tempo3$day==126,"dist"],tempo3[tempo3$day==126,"1"],type = "b",ylim=c(-0.6,0.6),pch=19,cex=1.5,xlab="Distance(km)",ylab="PCO3",main="prediction with other variables interaction",col="gold2",lwd=3,lty=4)

points(tempo3[tempo3$day==1,"dist"],tempo3[tempo3$day==1,"1"],type="b",col="black",pch=8,cex=1.5,lwd=3,lty=3)

points(tempo3[tempo3$day==260,"dist"],tempo3[tempo3$day==260,"1"],type="b",col="blue",pch=8,cex=1.5,lwd=3,lty=3)

points(tempo3[tempo3$day==336,"dist"],tempo3[tempo3$day==336,"1"],type="b",col="green3",pch=17,cex=1.5,lwd=3)

points(tempo3[tempo3$day==518,"dist"],tempo3[tempo3$day==518,"1"],type="b",col="red",pch=18,cex=2,lwd=3,lty=5)

legend("topright",inset=c(0,0),legend=c("April 2014", "Aug 2014", "Nov 2014","May 2015"),lty=1,lwd=2,col=c("gold2","blue","green3","red"),ncol=4,horiz=FALSE,cex=0.7,title="months")

abline(v=4,lty=2)

xyplot(fitted(tempo)~dist, groups=day,data=tempo3,type="l",auto.key=TRUE)

xyplot(residuals(tempo)~dist, groups=day,data=tempo3,type="l",auto.key=TRUE)

tempo4<-lm(tempo3[,2]~tempo3$pred3)

summary(tempo4)

plot(alldata$nh3~alldata$pco3)

cor.test(alldata$nh3,alldata$pco3)

plot(log(alldata$temp),alldata$pco3)

cor.test(log(alldata$temp),alldata$pco3)

##Testing the variables before dbRDA --PCO4

edit(alldata)

summary(tempo)

tempo<-lm(pco4~temp,data=alldata)

tempo<-lm(pco4~cond,data=alldata)

tempo<-lm(pco4~log(temp)+cond+log(temp):cond,data=alldata)

tempo<-lm(pco4~cr,data=alldata)

tempo<-lm(pco4~temp+cr+temp:cr,data=alldata)

tempo<-lm(pco4~temp+cond+temp:cond+cr+temp:cr,data=alldata)

tempo<-lm(pco4~zn,data=alldata)

tempo<-lm(pco4~temp+zn+zn:temp,data=alldata)

tempo<-lm(pco4~temp+cond+temp:cond+zn+zn:temp,data=alldata)

tempo<-lm(pco4~cd,data=alldata)

tempo<-lm(pco4~temp+cd+cd:temp,data=alldata)

tempo<-lm(pco4~temp+cond+temp:cond+cd+cd:temp,data=alldata)

tempo<-lm(pco4~cu,data=alldata)

tempo<-lm(pco4~temp+cu+cu:temp,data=alldata)

tempo<-lm(pco4~temp+cond+temp:cond+cu+cu:temp,data=alldata)

tempo<-lm(pco4~log(chla),data=alldata)

tempo<-lm(pco4~temp+log(chla)+log(chla):temp,data=alldata)

tempo<-lm(pco4~temp+cond+temp:cond+log(chla)+temp:log(chla),data=alldata)

tempo<-lm(pco4~do,data=alldata)

tempo<-lm(pco4~temp+do+do:temp,data=alldata)

tempo<-lm(pco4~temp+cond+temp:cond+do+do:temp,data=alldata)

tempo<-lm(pco4~cfpom,data=alldata)

tempo<-lm(pco4~cfpom+temp+temp:cfpom,data=alldata)

tempo<-lm(pco4~temp+cond+temp:cond+cfpom+cfpom:temp,data=alldata)

tempo<-lm(pco4~cod,data=alldata)

tempo<-lm(pco4~cod+temp+temp:cod,data=alldata)

tempo<-lm(pco4~temp+cond+temp:cond+cod+cod:temp,data=alldata)

tempo<-lm(pco4~turb,data=alldata)

tempo<-lm(pco4~temp+turb+turb:temp,data=alldata)

tempo<-lm(pco4~temp+cond+temp:cond+turb+turb:temp,data=alldata)

tempo<-lm(pco4~vel,data=alldata)

tempo<-lm(pco4~temp+vel+vel:temp,data=alldata)

tempo<-lm(pco4~temp+cond+temp:cond+vel+vel:temp,data=alldata)

tempo<-lm(pco4~nh3,data=alldata)

tempo<-lm(pco4~temp+nh3+nh3:temp,data=alldata)

tempo<-lm(pco4~temp+cond+temp:cond+nh3+nh3:temp,data=alldata)

tempo<-lm(pco4~toc,data=alldata)

tempo<-lm(pco4~temp+toc+toc:temp,data=alldata)

tempo<-lm(pco4~temp+cond+temp:cond+toc+toc:temp,data=alldata)

tempo<-lm(pco4~ph2,data=alldata)

tempo<-lm(pco4~temp+ph2+ph2:temp,data=alldata)

tempo<-lm(pco4~temp+cond+temp:cond+ph2+ph2:temp,data=alldata)

## final models for pco4

tempo<-lm(pco4~temp+cfpom+temp:cfpom,data=alldata) ## temp:cfpom ---> Adjusted R-squared: 0.4266

tempo<-lm(pco4~temp+toc+temp:toc,data=alldata)## temp:toc ---> Adjusted R-squared: 0.2768

tempo<-lm(pco4~do,data=alldata)##Adjusted R-squared: 0.2103

tempo<-lm(pco4~turb,data=alldata)##Adjusted R-squared: 0.1948

tempo<-lm(pco4~temp+cod+temp:cod,data=alldata)## temp:cod ---> Adjusted R-squared: 0.182

tempo<-lm(pco4~cfpom,data=alldata)##Adjusted R-squared: 0.154

tempo<-lm(pco4~temp+toc+temp:toc+cfpom+temp:cfpom,data=alldata)## temp:toc and temp:cfpom ---> Adjusted R-squared: 0.5269

tempo<-lm(pco4~temp+toc+temp:toc+cod+temp:cod+cfpom+temp:cfpom,data=alldata)## temp:toc, temp:cod and temp:cfpom --->Adjusted R-squared: 0.5242

tempo<-lm(pco4~temp+toc+temp:toc+cod+temp:cod+cfpom+temp:cfpom+do+turb,data=alldata)## temp:toc, temp:cod , temp:cfpom ,do and turb ---> Adjusted R-squared: 0.4054

tempo<-lm(pco4~temp+toc+temp:toc+cod+temp:cod+cfpom+temp:cfpom+turb,data=alldata)## temp:toc, temp:cod , temp:cfpom ,do and turb ---> Adjusted R-squared: 0.5261

summary(tempo)

tempo4<-cbind(cbind(fitted(tempo),residuals(tempo)),alldata[complete.cases(alldata[,c("temp","toc","cod","cfpom","turb")]),])

attributes(tempo4)

names(tempo4[,1:2])<-c("fitted","residual")

edit(tempo4)

tempo4[,"2"]

tempo5<-lm(tempo4[,"2"]~pred4,data=tempo4)

summary(tempo5)

par(mar=c(5,5,4,2),cex=0.9)

plot(tempo4[tempo4$day==126,"dist"],tempo4[tempo4$day==126,"1"],type = "b",ylim=c(-0.6,0.6),pch=19,cex=1.5,xlab="Distance(km)",ylab="PCO4",main="prediction with other variables interaction",col="gold2",lwd=3,lty=4)

points(tempo4[tempo4$day==1,"dist"],tempo4[tempo4$day==1,"1"],type="b",col="black",pch=8,cex=1.5,lwd=3,lty=3)

points(tempo4[tempo4$day==260,"dist"],tempo4[tempo4$day==260,"1"],type="b",col="blue",pch=8,cex=1.5,lwd=3,lty=3)

points(tempo4[tempo4$day==336,"dist"],tempo4[tempo4$day==336,"1"],type="b",col="green3",pch=17,cex=1.5,lwd=3)

points(tempo4[tempo4$day==518,"dist"],tempo4[tempo4$day==518,"1"],type="b",col="red",pch=18,cex=2,lwd=3,lty=5)

legend("topright",inset=c(0,0),legend=c("April 2014", "Aug 2014", "Nov 2014","May 2015"),lty=1,lwd=2,col=c("gold2","blue","green3","red"),ncol=4,horiz=FALSE,cex=0.7,title="months")

abline(v=4,lty=2)

xyplot(fitted(tempo)~dist, groups=day,data=tempo4,type="l",auto.key=TRUE)

xyplot(residuals(tempo)~dist, groups=day,data=tempo4,type="l",auto.key=TRUE)

tempo5<-lm(tempo4[,2]~tempo4$pred4)

summary(tempo5)

plot(alldata$turb~alldata$pco4)

cor.test(alldata$turb,alldata$pco4)

plot(log(alldata$turb),alldata$pco4)

cor.test(log(alldata$turb),alldata$pco4)

##Testing the variables before dbRDA --PCO5

edit(alldata)

summary(tempo)

tempo<-lm(pco5~temp,data=alldata)

tempo<-lm(pco5~cond,data=alldata)

tempo<-lm(pco5~temp+cond+log(temp):cond,data=alldata)

tempo<-lm(pco5~cr,data=alldata)

tempo<-lm(pco5~temp+cr+temp:cr,data=alldata)

tempo<-lm(pco5~temp+cond+temp:cond+cr+temp:cr,data=alldata)

tempo<-lm(pco5~zn,data=alldata)

tempo<-lm(pco5~temp+zn+zn:temp,data=alldata)

tempo<-lm(pco5~temp+cond+temp:cond+zn+zn:temp,data=alldata)

tempo<-lm(pco5~cd,data=alldata)

tempo<-lm(pco5~temp+cd+cd:temp,data=alldata)

tempo<-lm(pco5~temp+cond+temp:cond+cd+cd:temp,data=alldata)

tempo<-lm(pco5~cu,data=alldata)

tempo<-lm(pco5~temp+cu+cu:temp,data=alldata)

tempo<-lm(pco5~temp+cond+temp:cond+cu+cu:temp,data=alldata)

tempo<-lm(pco5~log(chla),data=alldata)

tempo<-lm(pco5~temp+log(chla)+log(chla):temp,data=alldata)

tempo<-lm(pco5~temp+cond+temp:cond+log(chla)+temp:log(chla),data=alldata)

tempo<-lm(pco5~do,data=alldata)

tempo<-lm(pco5~temp+do+do:temp,data=alldata)

tempo<-lm(pco5~temp+cond+temp:cond+do+do:temp,data=alldata)

tempo<-lm(pco5~cfpom,data=alldata)

tempo<-lm(pco5~cfpom+temp+temp:cfpom,data=alldata)

tempo<-lm(pco5~temp+cond+temp:cond+cfpom+cfpom:temp,data=alldata)

tempo<-lm(pco5~cod,data=alldata)

tempo<-lm(pco5~cod+temp+temp:cod,data=alldata)

tempo<-lm(pco5~temp+cond+temp:cond+cod+cod:temp,data=alldata)

tempo<-lm(pco5~turb,data=alldata)

tempo<-lm(pco5~temp+turb+turb:temp,data=alldata)

tempo<-lm(pco5~temp+cond+temp:cond+turb+turb:temp,data=alldata)

tempo<-lm(pco5~vel,data=alldata)

tempo<-lm(pco5~temp+vel+vel:temp,data=alldata)

tempo<-lm(pco5~temp+cond+temp:cond+vel+vel:temp,data=alldata)

tempo<-lm(pco5~nh3,data=alldata)

tempo<-lm(pco5~temp+nh3+nh3:temp,data=alldata)

tempo<-lm(pco5~temp+cond+temp:cond+nh3+nh3:temp,data=alldata)

tempo<-lm(pco5~toc,data=alldata)

tempo<-lm(pco5~temp+toc+toc:temp,data=alldata)

tempo<-lm(pco5~temp+cond+temp:cond+toc+toc:temp,data=alldata)

tempo<-lm(pco5~ph2,data=alldata)

tempo<-lm(pco5~temp+ph2+ph2:temp,data=alldata)

tempo<-lm(pco5~temp+cond+temp:cond+ph2+ph2:temp,data=alldata)

tempo<-lm(pco5~sed,data=alldata)

## final models for pco5

tempo<-lm(pco5~log(vel),data=alldata)##

tempo<-lm(pco5~ph2,data=alldata)## ---> Adjusted R-squared: 0.2372

tempo<-lm(pco5~cod,data=alldata)##---> Adjusted R-squared: 0.1677

tempo<-lm(pco5~ph2+cod,data=alldata)##Adjusted R-squared: 0.3274

summary(tempo)

tempo5<-cbind(cbind(fitted(tempo),residuals(tempo)),alldata)#[complete.cases(alldata[,c("temp","cond")]),])

attributes(tempo5)

names(tempo5[,1:2])<-c("fitted","residual")

edit(tempo5)

tempo5[,"2"]

tempo6<-lm(tempo5[,"2"]~pred5,data=tempo5)

summary(tempo6)

par(mar=c(5,5,4,2),cex=0.9)

plot(tempo5[tempo5$day==1,"dist"],tempo5[tempo5$day==1,"1"],type = "b",ylim=c(-0.6,0.6),pch=19,cex=1.5,xlab="Distance(km)",ylab="PCO4",main="prediction with other variables interaction",col="gold2",lwd=3,lty=4)

points(tempo5[tempo5$day==126,"dist"],tempo5[tempo5$day==126,"1"],type="b",col="black",pch=8,cex=1.5,lwd=3,lty=3)

points(tempo5[tempo5$day==260,"dist"],tempo5[tempo5$day==260,"1"],type="b",col="blue",pch=8,cex=1.5,lwd=3,lty=3)

points(tempo5[tempo5$day==336,"dist"],tempo5[tempo5$day==336,"1"],type="b",col="green3",pch=17,cex=1.5,lwd=3)

points(tempo5[tempo5$day==518,"dist"],tempo5[tempo5$day==518,"1"],type="b",col="red",pch=18,cex=2,lwd=3,lty=5)

legend("topright",inset=c(0,0),legend=c("April 2014", "Aug 2014", "Nov 2014","May 2015"),lty=1,lwd=2,col=c("gold2","blue","green3","red"),ncol=4,horiz=FALSE,cex=0.7,title="months")

abline(v=4,lty=2)

xyplot(fitted(tempo)~dist, groups=day,data=tempo5,type="l",auto.key=TRUE)

xyplot(residuals(tempo)~dist, groups=day,data=tempo5,type="l",auto.key=TRUE)

tempo6<-lm(tempo5[,2]~tempo5$pred5)

summary(tempo6)

plot(alldata$ph2~alldata$pco5)

cor.test(alldata$ph2,alldata$pco5)

plot(log(alldata$chla),alldata$pco5)

cor.test(log(alldata$chla),alldata$pco5)

##Testing the variables before dbRDA --PCO6

edit(alldata)

summary(tempo)

tempo<-lm(pco6~temp,data=alldata)

tempo<-lm(pco6~cond,data=alldata)

tempo<-lm(pco6~temp+cond+log(temp):cond,data=alldata)

tempo<-lm(pco6~cr,data=alldata)

tempo<-lm(pco6~temp+cr+temp:cr,data=alldata)

tempo<-lm(pco6~temp+cond+temp:cond+cr+temp:cr,data=alldata)

tempo<-lm(pco6~zn,data=alldata)

tempo<-lm(pco6~temp+zn+zn:temp,data=alldata)

tempo<-lm(pco6~temp+cond+temp:cond+zn+zn:temp,data=alldata)

tempo<-lm(pco6~cd,data=alldata)

tempo<-lm(pco6~temp+cd+cd:temp,data=alldata)

tempo<-lm(pco6~temp+cond+temp:cond+cd+cd:temp,data=alldata)

tempo<-lm(pco6~cu,data=alldata)

tempo<-lm(pco6~temp+cu+cu:temp,data=alldata)

tempo<-lm(pco6~temp+cond+temp:cond+cu+cu:temp,data=alldata)

tempo<-lm(pco6~log(chla),data=alldata)

tempo<-lm(pco6~temp+log(chla)+log(chla):temp,data=alldata)

tempo<-lm(pco6~temp+cond+temp:cond+log(chla)+temp:log(chla),data=alldata)

tempo<-lm(pco6~do,data=alldata)

tempo<-lm(pco6~temp+do+do:temp,data=alldata)

tempo<-lm(pco6~temp+cond+temp:cond+do+do:temp,data=alldata)

tempo<-lm(pco6~cfpom,data=alldata)

tempo<-lm(pco6~cfpom+temp+temp:cfpom,data=alldata)

tempo<-lm(pco6~temp+cond+temp:cond+cfpom+cfpom:temp,data=alldata)

tempo<-lm(pco6~cod,data=alldata)

tempo<-lm(pco6~cod+temp+temp:cod,data=alldata)

tempo<-lm(pco6~temp+cond+temp:cond+cod+cod:temp,data=alldata)

tempo<-lm(pco6~turb,data=alldata)

tempo<-lm(pco6~temp+turb+turb:temp,data=alldata)

tempo<-lm(pco6~temp+cond+temp:cond+turb+turb:temp,data=alldata)

tempo<-lm(pco6~vel,data=alldata)

tempo<-lm(pco6~temp+vel+vel:temp,data=alldata)

tempo<-lm(pco6~temp+cond+temp:cond+vel+vel:temp,data=alldata)

tempo<-lm(pco6~nh3,data=alldata)

tempo<-lm(pco6~temp+nh3+nh3:temp,data=alldata)

tempo<-lm(pco6~temp+cond+temp:cond+nh3+nh3:temp,data=alldata)

tempo<-lm(pco6~toc,data=alldata)

tempo<-lm(pco6~temp+toc+toc:temp,data=alldata)

tempo<-lm(pco6~temp+cond+temp:cond+toc+toc:temp,data=alldata)

tempo<-lm(pco6~ph2,data=alldata)

tempo<-lm(pco6~temp+ph2+ph2:temp,data=alldata)

tempo<-lm(pco6~temp+cond+temp:cond+ph2+ph2:temp,data=alldata)

tempo<-lm(pco6~sed,data=alldata)

## final models for pco6

tempo<-lm(pco6~do+do:temp+temp,data=alldata)## Adjusted R-squared: 0.1395

summary(tempo)

tempo6<-cbind(cbind(fitted(tempo),residuals(tempo)),alldata[complete.cases(alldata[,c("do")]),])

attributes(tempo6)

names(tempo6[,1:2])<-c("fitted","residual")

edit(tempo6)

tempo6[,"2"]

tempo7<-lm(tempo6[,"2"]~pred6,data=tempo6)

summary(tempo7)

par(mar=c(5,5,4,2),cex=0.9)

plot(tempo6[tempo6$day==126,"dist"],tempo6[tempo6$day==126,"1"],type = "b",ylim=c(-0.6,0.6),pch=19,cex=1.5,xlab="Distance(km)",ylab="PCO4",main="prediction with other variables interaction",col="gold2",lwd=3,lty=4)

points(tempo6[tempo6$day==126,"dist"],tempo6[tempo6$day==126,"1"],type="b",col="black",pch=8,cex=1.5,lwd=3,lty=3)

points(tempo6[tempo6$day==260,"dist"],tempo6[tempo6$day==260,"1"],type="b",col="blue",pch=8,cex=1.5,lwd=3,lty=3)

points(tempo6[tempo6$day==336,"dist"],tempo6[tempo6$day==336,"1"],type="b",col="green3",pch=17,cex=1.5,lwd=3)

points(tempo6[tempo6$day==518,"dist"],tempo6[tempo6$day==518,"1"],type="b",col="red",pch=18,cex=2,lwd=3,lty=5)

legend("topright",inset=c(0,0),legend=c("April 2014", "Aug 2014", "Nov 2014","May 2015"),lty=1,lwd=2,col=c("gold2","blue","green3","red"),ncol=4,horiz=FALSE,cex=0.7,title="months")

abline(v=4,lty=2)

plot(alldata$do~alldata$pco6)

cor.test(alldata$do,alldata$pco6)

plot(log(alldata$do),alldata$pco6)

cor.test(log(alldata$do),alldata$pco6)

##############dbRDA for pco1~env ## to check which variables explain time:space (spatiotemporal variation)

wang.cap1<-capscale(formula=wangbug.BC~dist+time+dist:time+eff+eff:time+eff:dist:time,data=wangenv,comm=wangbug,add=TRUE,na.action=na.omit)

summary(wang.cap1)

wang.anova1<-anova(wang.cap1,by="term")

print(wang.anova1)

edit(same.sites(wangbug,wangenv1))

wangenv1<-wangenv[complete.cases(wangenv[,c("cod","temp","zn","chla","cond")]),]#omit season1

edit(wangenv1)

wangenv1<- removeNAenv(wangenv1,"cod")

wangenv1<- removeNAenv(wangenv1,"temp")

wangenv1<- removeNAenv(wangenv1,"zn")

wangenv1<- removeNAenv(wangenv1,"chla")

wangenv1<- removeNAenv(wangenv1,"cond")

wangbug1<-same.sites(wangbug,wangenv1)

edit(wangbug1)

check.datasets(wangbug1,wangenv1)

edit(wangenv1)

wangbug1.BC<-vegdist(sqrt(wangbug1))

#wang.cap2<-capscale(formula=wangbug1.BC~temp+cond+temp:cond+cod+temp:cod+log(zn)+temp:log(zn)+toc+temp:toc+log(chla),data=wangenv1,comm=wangbug1,add=TRUE,na.action=na.omit)## all the significant variables with pco1

#wang.cap2<-capscale(formula=wangbug1.BC~temp+cond+temp:cond+cod+temp:cod+log(zn)+toc+log(chla),data=wangenv1,comm=wangbug1,add=TRUE,na.action=na.omit)## without toc:temp and zn:temp

#wang.cap2<-capscale(formula=wangbug1.BC~temp+cond+temp:cond+cod+temp:cod+log(zn)+log(chla),data=wangenv1,comm=wangbug1,add=TRUE,na.action=na.omit)## without toc, toc:temp and zn:temp

#wang.cap2<-capscale(formula=wangbug1.BC~temp+cond+temp:cond+log(zn)+temp:log(zn)+toc+temp:toc+log(chla),data=wangenv1,comm=wangbug1,add=TRUE,na.action=na.omit)## without cod

#wang.cap2<-capscale(formula=wangbug1.BC~temp+cond+temp:cond+log(zn)+toc+temp:toc+log(chla),data=wangenv1,comm=wangbug1,add=TRUE,na.action=na.omit)## without cod and zn:temp

#wang.cap2<-capscale(formula=wangbug1.BC~temp+cond+temp:cond+log(zn)+toc+temp:toc+log(chla)+nh3,data=wangenv1,comm=wangbug1,add=TRUE,na.action=na.omit)## without cod and zn:temp, including nh3

#wang.cap2<-capscale(formula=wangbug1.BC~temp+cond+temp:cond+log(zn)+toc+temp:toc+log(chla)+no3,data=wangenv1,comm=wangbug1,add=TRUE,na.action=na.omit)##without cod and zn:temp, including no3

#wang.cap2<-capscale(formula=wangbug1.BC~temp+cond+temp:cond+log(zn)+toc+temp:toc+log(chla)+tp,data=wangenv1,comm=wangbug1,add=TRUE,na.action=na.omit)## without cod and zn:temp, including tp

#wang.cap2<-capscale(formula=wangbug1.BC~temp+cond+temp:cond+log(zn)+toc+temp:toc+log(chla)+tn,data=wangenv1,comm=wangbug1,add=TRUE,na.action=na.omit)## without cod and zn:temp, including tn

#wang.cap2<-capscale(formula=wangbug1.BC~temp+cond+temp:cond+log(zn)+toc+temp:toc+log(chla)+sed,data=wangenv1,comm=wangbug1,add=TRUE,na.action=na.omit)## without cod and zn:temp, including sed

#wang.cap2<-capscale(formula=wangbug1.BC~temp+cond+temp:cond+log(zn)+toc+temp:toc+log(chla)+ph2,data=wangenv1,comm=wangbug1,add=TRUE,na.action=na.omit)## without cod and zn:temp, including ph

#wang.cap2<-capscale(formula=wangbug1.BC~temp+cond+temp:cond+log(zn)+toc+temp:toc+log(chla)+cfpom,data=wangenv1,comm=wangbug1,add=TRUE,na.action=na.omit)## without cod and zn:temp, including cfpom

#wang.cap2<-capscale(formula=wangbug1.BC~temp+cond+temp:cond+log(zn)+toc+temp:toc+log(chla)+turb,data=wangenv1,comm=wangbug1,add=TRUE,na.action=na.omit)## without cod and zn:temp, including turb

wang.cap2<-capscale(formula=wangbug1.BC~temp+cond+temp:cond+log(zn)+toc+temp:toc+log(chla)+log(cr),data=wangenv1,comm=wangbug1,add=TRUE,na.action=na.omit)## without cod and zn:temp, including cr # Final model

anova(wang.cap2)

#wang.cap2<-capscale(formula=wangbug1.BC~as.factor(eff)+time+dist+as.factor(eff):time+Condition(temp+cond+temp:cond+cod+temp:cod+log(zn)+temp:log(zn)+toc+temp:toc+log(chla)),data=wangenv1,comm=wangbug1,add=TRUE,na.action=na.omit)## condition (all the significant variables with pco1)

#wang.cap2<-capscale(formula=wangbug1.BC~as.factor(eff)+time+dist+as.factor(eff):time+Condition(temp+cond+temp:cond+cod+temp:cod+log(zn)+toc+log(chla)),data=wangenv1,comm=wangbug1,add=TRUE,na.action=na.omit)## condition (without toc:temp and zn:temp)

#wang.cap2<-capscale(formula=wangbug1.BC~as.factor(eff)+time+dist+as.factor(eff):time+Condition(temp+cond+temp:cond+cod+temp:cod+log(zn)+log(chla)),data=wangenv1,comm=wangbug1,add=TRUE,na.action=na.omit) ## condition (without toc, toc:temp and zn:temp)

#wang.cap2<-capscale(formula=wangbug1.BC~as.factor(eff)+time+dist+as.factor(eff):time+Condition(temp+cond+temp:cond+log(zn)+temp:log(zn)+toc+temp:toc+log(chla)),data=wangenv1,comm=wangbug1,add=TRUE,na.action=na.omit)## condition (without cod)

#wang.cap2<-capscale(formula=wangbug1.BC~as.factor(eff)+time+dist+as.factor(eff):time+Condition(temp+cond+temp:cond+log(zn)+toc+temp:toc+log(chla)),data=wangenv1,comm=wangbug1,add=TRUE,na.action=na.omit)## condition (without cod and temp:zn)

#wang.cap2<-capscale(formula=wangbug1.BC~as.factor(eff)+time+dist+as.factor(eff):time+Condition(temp+cond+temp:cond+log(zn)+toc+temp:toc+log(chla)+nh3),data=wangenv1,comm=wangbug1,add=TRUE,na.action=na.omit)## condition (without cod and temp:zn, including nh3)

#wang.cap2<-capscale(formula=wangbug1.BC~as.factor(eff)+time+dist+as.factor(eff):time+Condition(temp+cond+temp:cond+log(zn)+toc+temp:toc+log(chla)+no2),data=wangenv1,comm=wangbug1,add=TRUE,na.action=na.omit)## condition (without cod and temp:zn, including no2)

#wang.cap2<-capscale(formula=wangbug1.BC~as.factor(eff)+time+dist+as.factor(eff):time+Condition(temp+cond+temp:cond+log(zn)+toc+temp:toc+log(chla)+log(cr)),data=wangenv1,comm=wangbug1,add=TRUE,na.action=na.omit)## condition (without cod and temp:zn, including cr)

wang.cap3<-capscale(formula=wangbug1.BC~dist+time+dist:time+eff+eff:time+eff:dist:time+Condition(temp+cond+temp:cond+log(zn)+toc+temp:toc+log(chla)+log(cr)),data=wangenv1,comm=wangbug1,add=TRUE,na.action=na.omit)## condition (without cod and temp:zn, including cr) # Final model

summary(wang.cap2)

wang.anova2<-anova(wang.cap2,by="term",permutations=how(nperm=9999))

print(wang.anova2)##

summary(wang.cap2)

summary(wang.cap3)

wang.anova3<-anova(wang.cap3,by="term",permutations=how(nperm=9999)) # No evidence of spatiotemporal patterns

print(wang.anova3)###

wang.cap2coef<-coef(wang.cap2)

write.table(wang.cap2coef,file="wang.cap2coef.txt",sep="\t")

####Calculate fitted and residual for environmental variables

alldata<-cbind(envpcopred,pwangbug)#

edit(alldata)

tempo<-lm(pco1~temp+cond+temp:cond,data=alldata)

tempo<-lm(pco1~tn+op+alk+zn+chla+temp+cond+temp:cond,data=alldata)

tempo<-lm(pco1~tn+op+zn+temp+cond+temp:cond,data=alldata)

summary(tempo)

anova(tempo)

plot(tempo)

fitted(tempo)

edit(tempo1)

tempo1<-cbind(cbind(fitted(tempo),residuals(tempo)),alldata[complete.cases(alldata[,c("temp","cond")]),])

attributes(tempo1)

names(tempo1[,1:2])<-c("fitted","residual")

par(mar=c(5,5,4,2),cex=0.9)

plot(tempo1[tempo1$day==126,"dist"],tempo1[tempo1$day==126,"1"],type = "b",ylim=c(-0.6,0.6),pch=19,cex=1.5,xlab="Distance(km)",ylab="PCO1",main="prediction with other variables and Temperature*Conductivity interaction",col="gold2",lwd=3,lty=4)

points(tempo1[tempo1$day==260,"dist"],tempo1[tempo1$day==260,"1"],type="b",col="blue",pch=8,cex=1.5,lwd=3,lty=3)

points(tempo1[tempo1$day==336,"dist"],tempo1[tempo1$day==336,"1"],type="b",col="green3",pch=17,cex=1.5,lwd=3)

points(tempo1[tempo1$day==518,"dist"],tempo1[tempo1$day==518,"1"],type="b",col="red",pch=18,cex=2,lwd=3,lty=5)

legend("topright",inset=c(0,0),legend=c("April 2014", "Aug 2014", "Nov 2014","May 2015"),lty=1,lwd=2,col=c("gold2","blue","green3","red"),ncol=4,horiz=FALSE,cex=0.7,title="months")

abline(v=4,lty=2)

xyplot(fitted(tempo)~dist, groups=day,data=tempo1,type="l",auto.key=TRUE)

xyplot(residuals(tempo)~dist, groups=day,data=tempo1,type="l",auto.key=TRUE)

tempo3<-lm(tempo1[,2]~tempo1$pred1)

summary(tempo3)

###Shannon index

shannon<-diversity(wangbug, index = "shannon", MARGIN = 1, base = exp(1))

shanwangbug<-cbind(wangenv,shannon)

plot(wangenv$dist[1:8],shannon[1:8],xlab = "sites", ylab = "Shannon index",type="b",pch=19,ylim = c(0,3.5),col="black",lwd=3,lty=1)

points(wangenv$dist[9:16],shannon[9:16],xlab = "sites", ylab = "Shannon index",type="b",pch=15,col="gold2",lwd=3,cex=1.2,lty=2)

points(wangenv$dist[17:24],shannon[17:24],xlab = "sites", ylab = "Shannon index",type="b",pch=8,col="blue",cex=1.2,lwd=3,lty=3)

points(wangenv$dist[25:32],shannon[25:32],xlab = "sites", ylab = "Shannon index",type="b",col="green3",pch=17,cex=1.2,lwd=3,lty=4)

points(wangenv$dist[33:40],shannon[33:40],xlab = "sites", ylab = "Shannon index",type="b",col="red",pch=18,cex=1.4,lwd=3,lty=5)

legend("topright",inset=c(0,0),legend=c("Dec 13", "April 14", "Aug 2014", "Nov 2014","May 2015"),lty=c(1,5),pch=c(19,15,8,17,18),lwd=2,col=c("black","gold2","blue","green3","red"),ncol=2,horiz=FALSE,cex=0.6,title="months")

abline(v=4,lty=2)

edit(shanwangbug)

##Save shannon index into a CSV file

write.csv(shanwangbug)

write.csv(shanwangbug, file = "Shannon.csv")

######PCOs and Diversity indices

#PCO1

plot(pwangbug[,"pco1"],diversity[,"shannon"])

plot(pwangbug[,"pco1"],diversity[,"signal"])

plot(pwangbug[,"pco1"],diversity[,"taxarich"])

plot(pwangbug[,"pco1"],diversity[,"abundance"])

plot(pwangbug[,"pco1"],diversity[,"eptnum"])

plot(pwangbug[,"pco1"],diversity[,"eptrich"])

pwangdiver<-cbind(diversity,pwangbug)

model1 <- lm(pwangdiver$pco1 ~ pwangdiver$shannon)

summary(model1)

anova(model1)

plot(pwangdiver$pco1 ~ pwangdiver$shannon)

abline(model1)

model1 <- lm(pwangdiver$pco1 ~ pwangdiver$signal)

summary(model1)

plot(pwangdiver$pco1 ~ pwangdiver$signal)

abline(model1)

model1 <- lm(pwangdiver$pco1 ~ pwangdiver$taxarich)

summary(model1)

plot(pwangdiver$pco1 ~ pwangdiver$taxarich)

abline(model1)

model1 <- lm(pwangdiver$pco1 ~ pwangdiver$abundance)

summary(model1)

plot(pwangdiver$pco1 ~ pwangdiver$abundance)

abline(model1)

model1 <- lm(pwangdiver$pco1 ~ pwangdiver$eptnum)

summary(model1)

plot(pwangdiver$pco1 ~ pwangdiver$eptnum)

abline(model1)

model1 <- lm(pwangdiver$pco1 ~ pwangdiver$eptrich)

summary(model1)

plot(pwangdiver$pco1 ~ pwangdiver$eptrich)

abline(model1)wangwang<-cbind(wangenv,wangbug)

wangenv5<-cbind(wangwang,pwangbug)

plot(pwangbug[,"pco1"],wangbug[,"gripopterygidae"]) # Checks on aggregate process and heat maps

pwangdiver<-cbind(diversity,pwangbug)

model1 <- lm(pwangdiver$pco1 ~ wangbug$gripopterygidae)

summary(model1)

plot(pwangdiver$pco1 ~ wangbug$gripopterygidae)

abline(model1)

plot(pwangbug[,"pco1"],wangbug[,"oligochaeta"]) # Checks on aggregate process and heat maps

model1 <- lm(pwangdiver$pco1 ~ wangbug$oligochaeta)

summary(model1)

plot(pwangdiver$pco1 ~ wangbug$oligochaeta)

abline(model1)

plot(pwangbug[,"pco1"],wangbug[,"simulidae"]) # Checks on aggregate process and heat maps

model1 <- lm(pwangdiver$pco1 ~ wangbug$simulidae)

summary(model1)

plot(pwangdiver$pco1 ~ wangbug$simulidae)

abline(model1)

plot(pwangbug[,"pco1"],wangbug[,"chironominae"]) # Checks on aggregate process and heat maps

model1 <- lm(pwangdiver$pco1 ~ wangbug$chironominae)

summary(model1)

plot(pwangdiver$pco1 ~ wangbug$chironominae)

abline(model1)

#######macroinvertebrate taxa plotted against distance

bugandenv<-cbind(wangenv,wangbug)

plot(bugandenv$oligochaeta,bugandenv$cr)

cor.test(bugandenv$oligochaeta,bugandenv$cr)

par(mar=c(5,5,4,2),cex=0.9)

plot(bugandenv[bugandenv$day==1,"dist"],bugandenv[bugandenv$day==1,"baetidae"],type = "b",ylim=c(0,400),pch=19,cex=1.5,xlab="Distance(km)",ylab="baetidae",main="baetidae plotted against spatial position",lwd=3,lty=4)

points(bugandenv[bugandenv$day==126,"dist"],bugandenv[wangenv$day==126,"baetidae"],type="b",col="gold2",pch=15,lwd=3,cex=1.5)

points(bugandenv[bugandenv$day==260,"dist"],bugandenv[bugandenv$day==260,"baetidae"],type="b",col="blue",pch=8,cex=1.5,lwd=3,lty=3)

points(bugandenv[bugandenv$day==336,"dist"],bugandenv[bugandenv$day==336,"baetidae"],type="b",col="green3",pch=17,cex=1.5,lwd=3)

points(bugandenv[bugandenv$day==518,"dist"],bugandenv[bugandenv$day==518,"baetidae"],type="b",col="red",pch=18,cex=2,lwd=3,lty=5)

legend("topright",inset=c(0,0),legend=c("Dec 2013", "April 2014", "Aug 2014", "Nov 2014","May 2015"),lty=1,lwd=2,col=c("black","gold2","blue","green3","red"),ncol=5,horiz=FALSE,cex=0.8,title="months")

abline(v=4,lty=2)

par(mar=c(5,5,4,2),cex=0.9)

plot(bugandenv[bugandenv$day==1,"dist"],bugandenv[bugandenv$day==1,"caenidae"],type = "b",ylim=c(0,100),pch=19,cex=1.5,xlab="Distance(km)",ylab="caenidae",main="caenidae plotted against spatial position",lwd=3,lty=4)

points(bugandenv[bugandenv$day==126,"dist"],bugandenv[wangenv$day==126,"caenidae"],type="b",col="gold2",pch=15,lwd=3,cex=1.5)

points(bugandenv[bugandenv$day==260,"dist"],bugandenv[bugandenv$day==260,"caenidae"],type="b",col="blue",pch=8,cex=1.5,lwd=3,lty=3)

points(bugandenv[bugandenv$day==336,"dist"],bugandenv[bugandenv$day==336,"caenidae"],type="b",col="green3",pch=17,cex=1.5,lwd=3)

points(bugandenv[bugandenv$day==518,"dist"],bugandenv[bugandenv$day==518,"caenidae"],type="b",col="red",pch=18,cex=2,lwd=3,lty=5)

legend("topright",inset=c(0,0),legend=c("Dec 2013", "April 2014", "Aug 2014", "Nov 2014","May 2015"),lty=1,lwd=2,col=c("black","gold2","blue","green3","red"),ncol=5,horiz=FALSE,cex=0.8,title="months")

abline(v=4,lty=2)

par(mar=c(5,5,4,2),cex=0.9)

plot(bugandenv[bugandenv$day==1,"dist"],bugandenv[bugandenv$day==1,"coloburiscidae"],type = "b",ylim=c(0,10),pch=19,cex=1.5,xlab="Distance(km)",ylab="coloburiscidae",main="coloburiscidae plotted against spatial position",lwd=3,lty=4)

points(bugandenv[bugandenv$day==126,"dist"],bugandenv[wangenv$day==126,"coloburiscidae"],type="b",col="gold2",pch=15,lwd=3,cex=1.5)

points(bugandenv[bugandenv$day==260,"dist"],bugandenv[bugandenv$day==260,"coloburiscidae"],type="b",col="blue",pch=8,cex=1.5,lwd=3,lty=3)

points(bugandenv[bugandenv$day==336,"dist"],bugandenv[bugandenv$day==336,"coloburiscidae"],type="b",col="green3",pch=17,cex=1.5,lwd=3)

points(bugandenv[bugandenv$day==518,"dist"],bugandenv[bugandenv$day==518,"coloburiscidae"],type="b",col="red",pch=18,cex=2,lwd=3,lty=5)

legend("topright",inset=c(0,0),legend=c("Dec 2013", "April 2014", "Aug 2014", "Nov 2014","May 2015"),lty=1,lwd=2,col=c("black","gold2","blue","green3","red"),ncol=5,horiz=FALSE,cex=0.8,title="months")

abline(v=4,lty=2)

par(mar=c(5,5,4,2),cex=0.9)

plot(bugandenv[bugandenv$day==1,"dist"],bugandenv[bugandenv$day==1,"leptophlebiidae"],type = "b",ylim=c(0,100),pch=19,cex=1.5,xlab="Distance(km)",ylab="leptophlebiidae",main="leptophlebiidae plotted against spatial position",lwd=3,lty=4)

points(bugandenv[bugandenv$day==126,"dist"],bugandenv[wangenv$day==126,"leptophlebiidae"],type="b",col="gold2",pch=15,lwd=3,cex=1.5)

points(bugandenv[bugandenv$day==260,"dist"],bugandenv[bugandenv$day==260,"leptophlebiidae"],type="b",col="blue",pch=8,cex=1.5,lwd=3,lty=3)

points(bugandenv[bugandenv$day==336,"dist"],bugandenv[bugandenv$day==336,"leptophlebiidae"],type="b",col="green3",pch=17,cex=1.5,lwd=3)

points(bugandenv[bugandenv$day==518,"dist"],bugandenv[bugandenv$day==518,"leptophlebiidae"],type="b",col="red",pch=18,cex=2,lwd=3,lty=5)

legend("topright",inset=c(0,0),legend=c("Dec 2013", "April 2014", "Aug 2014", "Nov 2014","May 2015"),lty=1,lwd=2,col=c("black","gold2","blue","green3","red"),ncol=5,horiz=FALSE,cex=0.8,title="months")

abline(v=4,lty=2)

par(mar=c(5,5,4,2),cex=0.9)

plot(bugandenv[bugandenv$day==1,"dist"],bugandenv[bugandenv$day==1,"gripopterygidae"],type = "b",ylim=c(0,40),pch=19,cex=1.5,xlab="Distance(km)",ylab="gripopterygidae",main="gripopterygidae plotted against spatial position",lwd=3,lty=4)

points(bugandenv[bugandenv$day==126,"dist"],bugandenv[wangenv$day==126,"gripopterygidae"],type="b",col="gold2",pch=15,lwd=3,cex=1.5)

points(bugandenv[bugandenv$day==260,"dist"],bugandenv[bugandenv$day==260,"gripopterygidae"],type="b",col="blue",pch=8,cex=1.5,lwd=3,lty=3)

points(bugandenv[bugandenv$day==336,"dist"],bugandenv[bugandenv$day==336,"gripopterygidae"],type="b",col="green3",pch=17,cex=1.5,lwd=3)

points(bugandenv[bugandenv$day==518,"dist"],bugandenv[bugandenv$day==518,"gripopterygidae"],type="b",col="red",pch=18,cex=2,lwd=3,lty=5)

legend("topright",inset=c(0,0),legend=c("Dec 2013", "April 2014", "Aug 2014", "Nov 2014","May 2015"),lty=1,lwd=2,col=c("black","gold2","blue","green3","red"),ncol=5,horiz=FALSE,cex=0.8,title="months")

abline(v=4,lty=2)

par(mar=c(5,5,4,2),cex=0.9)

plot(bugandenv[bugandenv$day==1,"dist"],bugandenv[bugandenv$day==1,"hydroptilidae"],type = "b",ylim=c(0,10),pch=19,cex=1.5,xlab="Distance(km)",ylab="hydroptilidae",main="hydroptilidae plotted against spatial position",lwd=3,lty=4)

points(bugandenv[bugandenv$day==126,"dist"],bugandenv[wangenv$day==126,"hydroptilidae"],type="b",col="gold2",pch=15,lwd=3,cex=1.5)

points(bugandenv[bugandenv$day==260,"dist"],bugandenv[bugandenv$day==260,"hydroptilidae"],type="b",col="blue",pch=8,cex=1.5,lwd=3,lty=3)

points(bugandenv[bugandenv$day==336,"dist"],bugandenv[bugandenv$day==336,"hydroptilidae"],type="b",col="green3",pch=17,cex=1.5,lwd=3)

points(bugandenv[bugandenv$day==518,"dist"],bugandenv[bugandenv$day==518,"hydroptilidae"],type="b",col="red",pch=18,cex=2,lwd=3,lty=5)

legend("topright",inset=c(0,0),legend=c("Dec 2013", "April 2014", "Aug 2014", "Nov 2014","May 2015"),lty=1,lwd=2,col=c("black","gold2","blue","green3","red"),ncol=5,horiz=FALSE,cex=0.8,title="months")

abline(v=4,lty=2)

par(mar=c(5,5,4,2),cex=0.9)

plot(bugandenv[bugandenv$day==1,"dist"],bugandenv[bugandenv$day==1,"leptoceridae"],type = "b",ylim=c(0,10),pch=19,cex=1.5,xlab="Distance(km)",ylab="leptoceridae",main="leptoceridae plotted against spatial position",lwd=3,lty=4)

points(bugandenv[bugandenv$day==126,"dist"],bugandenv[wangenv$day==126,"leptoceridae"],type="b",col="gold2",pch=15,lwd=3,cex=1.5)

points(bugandenv[bugandenv$day==260,"dist"],bugandenv[bugandenv$day==260,"leptoceridae"],type="b",col="blue",pch=8,cex=1.5,lwd=3,lty=3)

points(bugandenv[bugandenv$day==336,"dist"],bugandenv[bugandenv$day==336,"leptoceridae"],type="b",col="green3",pch=17,cex=1.5,lwd=3)

points(bugandenv[bugandenv$day==518,"dist"],bugandenv[bugandenv$day==518,"leptoceridae"],type="b",col="red",pch=18,cex=2,lwd=3,lty=5)

legend("topright",inset=c(0,0),legend=c("Dec 2013", "April 2014", "Aug 2014", "Nov 2014","May 2015"),lty=1,lwd=2,col=c("black","gold2","blue","green3","red"),ncol=5,horiz=FALSE,cex=0.8,title="months")

abline(v=4,lty=2)

par(mar=c(5,5,4,2),cex=0.9)

plot(bugandenv[bugandenv$day==1,"dist"],bugandenv[bugandenv$day==1,"calocidae"],type = "b",ylim=c(0,10),pch=19,cex=1.5,xlab="Distance(km)",ylab="calocidae",main="calocidae plotted against spatial position",lwd=3,lty=4)

points(bugandenv[bugandenv$day==126,"dist"],bugandenv[wangenv$day==126,"calocidae"],type="b",col="gold2",pch=15,lwd=3,cex=1.5)

points(bugandenv[bugandenv$day==260,"dist"],bugandenv[bugandenv$day==260,"calocidae"],type="b",col="blue",pch=8,cex=1.5,lwd=3,lty=3)

points(bugandenv[bugandenv$day==336,"dist"],bugandenv[bugandenv$day==336,"calocidae"],type="b",col="green3",pch=17,cex=1.5,lwd=3)

points(bugandenv[bugandenv$day==518,"dist"],bugandenv[bugandenv$day==518,"calocidae"],type="b",col="red",pch=18,cex=2,lwd=3,lty=5)

legend("topright",inset=c(0,0),legend=c("Dec 2013", "April 2014", "Aug 2014", "Nov 2014","May 2015"),lty=1,lwd=2,col=c("black","gold2","blue","green3","red"),ncol=5,horiz=FALSE,cex=0.8,title="months")

abline(v=4,lty=2)

par(mar=c(5,5,4,2),cex=0.9)

plot(bugandenv[bugandenv$day==1,"dist"],bugandenv[bugandenv$day==1,"hydrobiosidae"],type = "b",ylim=c(0,10),pch=19,cex=1.5,xlab="Distance(km)",ylab="hydrobiosidae",main="hydrobiosidae plotted against spatial position",lwd=3,lty=4)

points(bugandenv[bugandenv$day==126,"dist"],bugandenv[wangenv$day==126,"hydrobiosidae"],type="b",col="gold2",pch=15,lwd=3,cex=1.5)

points(bugandenv[bugandenv$day==260,"dist"],bugandenv[bugandenv$day==260,"hydrobiosidae"],type="b",col="blue",pch=8,cex=1.5,lwd=3,lty=3)

points(bugandenv[bugandenv$day==336,"dist"],bugandenv[bugandenv$day==336,"hydrobiosidae"],type="b",col="green3",pch=17,cex=1.5,lwd=3)

points(bugandenv[bugandenv$day==518,"dist"],bugandenv[bugandenv$day==518,"hydrobiosidae"],type="b",col="red",pch=18,cex=2,lwd=3,lty=5)

legend("topright",inset=c(0,0),legend=c("Dec 2013", "April 2014", "Aug 2014", "Nov 2014","May 2015"),lty=1,lwd=2,col=c("black","gold2","blue","green3","red"),ncol=5,horiz=FALSE,cex=0.8,title="months")

abline(v=4,lty=2)

par(mar=c(5,5,4,2),cex=0.9)

plot(bugandenv[bugandenv$day==1,"dist"],bugandenv[bugandenv$day==1,"hydropsychidae"],type = "b",ylim=c(0,10),pch=19,cex=1.5,xlab="Distance(km)",ylab="hydropsychidae",main="hydropsychidae plotted against spatial position",lwd=3,lty=4)

points(bugandenv[bugandenv$day==126,"dist"],bugandenv[wangenv$day==126,"hydropsychidae"],type="b",col="gold2",pch=15,lwd=3,cex=1.5)

points(bugandenv[bugandenv$day==260,"dist"],bugandenv[bugandenv$day==260,"hydropsychidae"],type="b",col="blue",pch=8,cex=1.5,lwd=3,lty=3)

points(bugandenv[bugandenv$day==336,"dist"],bugandenv[bugandenv$day==336,"hydropsychidae"],type="b",col="green3",pch=17,cex=1.5,lwd=3)

points(bugandenv[bugandenv$day==518,"dist"],bugandenv[bugandenv$day==518,"hydropsychidae"],type="b",col="red",pch=18,cex=2,lwd=3,lty=5)

legend("topright",inset=c(0,0),legend=c("Dec 2013", "April 2014", "Aug 2014", "Nov 2014","May 2015"),lty=1,lwd=2,col=c("black","gold2","blue","green3","red"),ncol=5,horiz=FALSE,cex=0.8,title="months")

abline(v=4,lty=2)

par(mar=c(5,5,4,2),cex=0.9)

plot(bugandenv[bugandenv$day==1,"dist"],bugandenv[bugandenv$day==1,"ecnomidae"],type = "b",ylim=c(0,10),pch=19,cex=1.5,xlab="Distance(km)",ylab="ecnomidae",main="ecnomidae plotted against spatial position",lwd=3,lty=4)

points(bugandenv[bugandenv$day==126,"dist"],bugandenv[wangenv$day==126,"ecnomidae"],type="b",col="gold2",pch=15,lwd=3,cex=1.5)

points(bugandenv[bugandenv$day==260,"dist"],bugandenv[bugandenv$day==260,"ecnomidae"],type="b",col="blue",pch=8,cex=1.5,lwd=3,lty=3)

points(bugandenv[bugandenv$day==336,"dist"],bugandenv[bugandenv$day==336,"ecnomidae"],type="b",col="green3",pch=17,cex=1.5,lwd=3)

points(bugandenv[bugandenv$day==518,"dist"],bugandenv[bugandenv$day==518,"ecnomidae"],type="b",col="red",pch=18,cex=2,lwd=3,lty=5)

legend("topright",inset=c(0,0),legend=c("Dec 2013", "April 2014", "Aug 2014", "Nov 2014","May 2015"),lty=1,lwd=2,col=c("black","gold2","blue","green3","red"),ncol=5,horiz=FALSE,cex=0.8,title="months")

abline(v=4,lty=2)

par(mar=c(5,5,4,2),cex=0.9)

plot(bugandenv[bugandenv$day==1,"dist"],bugandenv[bugandenv$day==1,"ceratopogonidae"],type = "b",ylim=c(0,30),pch=19,cex=1.5,xlab="Distance(km)",ylab="ceratopogonidae ",main="ceratopogonidae plotted against spatial position",lwd=3,lty=4)

points(bugandenv[bugandenv$day==126,"dist"],bugandenv[wangenv$day==126,"ceratopogonidae"],type="b",col="gold2",pch=15,lwd=3,cex=1.5)

points(bugandenv[bugandenv$day==260,"dist"],bugandenv[bugandenv$day==260,"ceratopogonidae"],type="b",col="blue",pch=8,cex=1.5,lwd=3,lty=3)

points(bugandenv[bugandenv$day==336,"dist"],bugandenv[bugandenv$day==336,"ceratopogonidae"],type="b",col="green3",pch=17,cex=1.5,lwd=3)

points(bugandenv[bugandenv$day==518,"dist"],bugandenv[bugandenv$day==518,"ceratopogonidae"],type="b",col="red",pch=18,cex=2,lwd=3,lty=5)

legend("topright",inset=c(0,0),legend=c("Dec 2013", "April 2014", "Aug 2014", "Nov 2014","May 2015"),lty=1,lwd=2,col=c("black","gold2","blue","green3","red"),ncol=5,horiz=FALSE,cex=0.8,title="months")

abline(v=4,lty=2)

par(mar=c(5,5,4,2),cex=0.9)

plot(bugandenv[bugandenv$day==1,"dist"],bugandenv[bugandenv$day==1,"chrysomelidae"],type = "b",ylim=c(0,30),pch=19,cex=1.5,xlab="Distance(km)",ylab="chrysomelidae",main="chrysomelidae plotted against spatial position",lwd=3,lty=4)

points(bugandenv[bugandenv$day==126,"dist"],bugandenv[wangenv$day==126,"chrysomelidae"],type="b",col="gold2",pch=15,lwd=3,cex=1.5)

points(bugandenv[bugandenv$day==260,"dist"],bugandenv[bugandenv$day==260,"chrysomelidae"],type="b",col="blue",pch=8,cex=1.5,lwd=3,lty=3)

points(bugandenv[bugandenv$day==336,"dist"],bugandenv[bugandenv$day==336,"chrysomelidae"],type="b",col="green3",pch=17,cex=1.5,lwd=3)

points(bugandenv[bugandenv$day==518,"dist"],bugandenv[bugandenv$day==518,"chrysomelidae"],type="b",col="red",pch=18,cex=2,lwd=3,lty=5)

legend("topright",inset=c(0,0),legend=c("Dec 2013", "April 2014", "Aug 2014", "Nov 2014","May 2015"),lty=1,lwd=2,col=c("black","gold2","blue","green3","red"),ncol=5,horiz=FALSE,cex=0.8,title="months")

abline(v=4,lty=2)

par(mar=c(5,5,4,2),cex=0.9)

plot(bugandenv[bugandenv$day==1,"dist"],bugandenv[bugandenv$day==1,"diamesinae"],type = "b",ylim=c(0,10),pch=19,cex=1.5,xlab="Distance(km)",ylab="diamesinae",main="diamesinae plotted against spatial position",lwd=3,lty=4)

points(bugandenv[bugandenv$day==126,"dist"],bugandenv[wangenv$day==126,"diamesinae"],type="b",col="gold2",pch=15,lwd=3,cex=1.5)

points(bugandenv[bugandenv$day==260,"dist"],bugandenv[bugandenv$day==260,"diamesinae"],type="b",col="blue",pch=8,cex=1.5,lwd=3,lty=3)

points(bugandenv[bugandenv$day==336,"dist"],bugandenv[bugandenv$day==336,"diamesinae"],type="b",col="green3",pch=17,cex=1.5,lwd=3)

points(bugandenv[bugandenv$day==518,"dist"],bugandenv[bugandenv$day==518,"diamesinae"],type="b",col="red",pch=18,cex=2,lwd=3,lty=5)

legend("topright",inset=c(0,0),legend=c("Dec 2013", "April 2014", "Aug 2014", "Nov 2014","May 2015"),lty=1,lwd=2,col=c("black","gold2","blue","green3","red"),ncol=5,horiz=FALSE,cex=0.8,title="months")

abline(v=4,lty=2)

par(mar=c(5,5,4,2),cex=0.9)

plot(bugandenv[bugandenv$day==1,"dist"],bugandenv[bugandenv$day==1,"empididae"],type = "b",ylim=c(0,20),pch=19,cex=1.5,xlab="Distance(km)",ylab="empididae",main="empididae plotted against spatial position",lwd=3,lty=4)

points(bugandenv[bugandenv$day==126,"dist"],bugandenv[wangenv$day==126,"empididae"],type="b",col="gold2",pch=15,lwd=3,cex=1.5)

points(bugandenv[bugandenv$day==260,"dist"],bugandenv[bugandenv$day==260,"empididae"],type="b",col="blue",pch=8,cex=1.5,lwd=3,lty=3)

points(bugandenv[bugandenv$day==336,"dist"],bugandenv[bugandenv$day==336,"empididae"],type="b",col="green3",pch=17,cex=1.5,lwd=3)

points(bugandenv[bugandenv$day==518,"dist"],bugandenv[bugandenv$day==518,"empididae"],type="b",col="red",pch=18,cex=2,lwd=3,lty=5)

legend("topright",inset=c(0,0),legend=c("Dec 2013", "April 2014", "Aug 2014", "Nov 2014","May 2015"),lty=1,lwd=2,col=c("black","gold2","blue","green3","red"),ncol=5,horiz=FALSE,cex=0.8,title="months")

abline(v=4,lty=2)

par(mar=c(5,5,4,2),cex=0.9)

plot(bugandenv[bugandenv$day==1,"dist"],bugandenv[bugandenv$day==1,"micronectidae"],type = "b",ylim=c(0,40),pch=19,cex=1.5,xlab="Distance(km)",ylab="micronectidae",main="micronectidae plotted against spatial position",lwd=3,lty=4)

points(bugandenv[bugandenv$day==126,"dist"],bugandenv[wangenv$day==126,"micronectidae"],type="b",col="gold2",pch=15,lwd=3,cex=1.5)

points(bugandenv[bugandenv$day==260,"dist"],bugandenv[bugandenv$day==260,"micronectidae"],type="b",col="blue",pch=8,cex=1.5,lwd=3,lty=3)

points(bugandenv[bugandenv$day==336,"dist"],bugandenv[bugandenv$day==336,"micronectidae"],type="b",col="green3",pch=17,cex=1.5,lwd=3)

points(bugandenv[bugandenv$day==518,"dist"],bugandenv[bugandenv$day==518,"micronectidae"],type="b",col="red",pch=18,cex=2,lwd=3,lty=5)

legend("topright",inset=c(0,0),legend=c("Dec 2013", "April 2014", "Aug 2014", "Nov 2014","May 2015"),lty=1,lwd=2,col=c("black","gold2","blue","green3","red"),ncol=5,horiz=FALSE,cex=0.8,title="months")

abline(v=4,lty=2)

par(mar=c(5,5,4,2),cex=0.9)

plot(bugandenv[bugandenv$day==1,"dist"],bugandenv[bugandenv$day==1,"orthocladiinae"],type = "b",ylim=c(0,100),pch=19,cex=1.5,xlab="Distance(km)",ylab="orthocladiinae",main="orthocladiinae plotted against spatial position",lwd=3,lty=4)

points(bugandenv[bugandenv$day==126,"dist"],bugandenv[wangenv$day==126,"orthocladiinae"],type="b",col="gold2",pch=15,lwd=3,cex=1.5)

points(bugandenv[bugandenv$day==260,"dist"],bugandenv[bugandenv$day==260,"orthocladiinae"],type="b",col="blue",pch=8,cex=1.5,lwd=3,lty=3)

points(bugandenv[bugandenv$day==336,"dist"],bugandenv[bugandenv$day==336,"orthocladiinae"],type="b",col="green3",pch=17,cex=1.5,lwd=3)

points(bugandenv[bugandenv$day==518,"dist"],bugandenv[bugandenv$day==518,"orthocladiinae"],type="b",col="red",pch=18,cex=2,lwd=3,lty=5)

legend("topright",inset=c(0,0),legend=c("Dec 2013", "April 2014", "Aug 2014", "Nov 2014","May 2015"),lty=1,lwd=2,col=c("black","gold2","blue","green3","red"),ncol=5,horiz=FALSE,cex=0.8,title="months")

abline(v=4,lty=2)

par(mar=c(5,5,4,2),cex=0.9)

plot(bugandenv[bugandenv$day==1,"dist"],bugandenv[bugandenv$day==1,"simulidae"],type = "b",ylim=c(0,100),pch=19,cex=1.5,xlab="Distance(km)",ylab="simulidae",main="simulidae plotted against spatial position",lwd=3,lty=4)

points(bugandenv[bugandenv$day==126,"dist"],bugandenv[wangenv$day==126,"simulidae"],type="b",col="gold2",pch=15,lwd=3,cex=1.5)

points(bugandenv[bugandenv$day==260,"dist"],bugandenv[bugandenv$day==260,"simulidae"],type="b",col="blue",pch=8,cex=1.5,lwd=3,lty=3)

points(bugandenv[bugandenv$day==336,"dist"],bugandenv[bugandenv$day==336,"simulidae"],type="b",col="green3",pch=17,cex=1.5,lwd=3)

points(bugandenv[bugandenv$day==518,"dist"],bugandenv[bugandenv$day==518,"simulidae"],type="b",col="red",pch=18,cex=2,lwd=3,lty=5)

legend("topright",inset=c(0,0),legend=c("Dec 2013", "April 2014", "Aug 2014", "Nov 2014","May 2015"),lty=1,lwd=2,col=c("black","gold2","blue","green3","red"),ncol=5,horiz=FALSE,cex=0.8,title="months")

abline(v=4,lty=2)

par(mar=c(5,5,4,2),cex=0.9)

plot(bugandenv[bugandenv$day==1,"dist"],bugandenv[bugandenv$day==1,"tanypodinae"],type = "b",ylim=c(0,30),pch=19,cex=1.5,xlab="Distance(km)",ylab="tanypodinae",main="tanypodinae plotted against spatial position",lwd=3,lty=4)

points(bugandenv[bugandenv$day==126,"dist"],bugandenv[wangenv$day==126,"tanypodinae"],type="b",col="gold2",pch=15,lwd=3,cex=1.5)

points(bugandenv[bugandenv$day==260,"dist"],bugandenv[bugandenv$day==260,"tanypodinae"],type="b",col="blue",pch=8,cex=1.5,lwd=3,lty=3)

points(bugandenv[bugandenv$day==336,"dist"],bugandenv[bugandenv$day==336,"tanypodinae"],type="b",col="green3",pch=17,cex=1.5,lwd=3)

points(bugandenv[bugandenv$day==518,"dist"],bugandenv[bugandenv$day==518,"tanypodinae"],type="b",col="red",pch=18,cex=2,lwd=3,lty=5)

legend("topright",inset=c(0,0),legend=c("Dec 2013", "April 2014", "Aug 2014", "Nov 2014","May 2015"),lty=1,lwd=2,col=c("black","gold2","blue","green3","red"),ncol=5,horiz=FALSE,cex=0.8,title="months")

abline(v=4,lty=2)

par(mar=c(5,5,4,2),cex=0.9)

plot(bugandenv[bugandenv$day==1,"dist"],bugandenv[bugandenv$day==1,"tipulidae"],type = "b",ylim=c(0,30),pch=19,cex=1.5,xlab="Distance(km)",ylab="tipulidae",main="tipulidae plotted against spatial position",lwd=3,lty=4)

points(bugandenv[bugandenv$day==126,"dist"],bugandenv[wangenv$day==126,"tipulidae"],type="b",col="gold2",pch=15,lwd=3,cex=1.5)

points(bugandenv[bugandenv$day==260,"dist"],bugandenv[bugandenv$day==260,"tipulidae"],type="b",col="blue",pch=8,cex=1.5,lwd=3,lty=3)

points(bugandenv[bugandenv$day==336,"dist"],bugandenv[bugandenv$day==336,"tipulidae"],type="b",col="green3",pch=17,cex=1.5,lwd=3)

points(bugandenv[bugandenv$day==518,"dist"],bugandenv[bugandenv$day==518,"tipulidae"],type="b",col="red",pch=18,cex=2,lwd=3,lty=5)

legend("topright",inset=c(0,0),legend=c("Dec 2013", "April 2014", "Aug 2014", "Nov 2014","May 2015"),lty=1,lwd=2,col=c("black","gold2","blue","green3","red"),ncol=5,horiz=FALSE,cex=0.8,title="months")

abline(v=4,lty=2)

par(mar=c(5,5,4,2),cex=0.9)

plot(bugandenv[bugandenv$day==1,"dist"],bugandenv[bugandenv$day==1,"oligochaeta"],type = "b",ylim=c(0,1000),pch=19,cex=1.5,xlab="Distance(km)",ylab="oligochaeta",main="oligochaeta plotted against spatial position",lwd=3,lty=4)

points(bugandenv[bugandenv$day==126,"dist"],bugandenv[wangenv$day==126,"oligochaeta"],type="b",col="gold2",pch=15,lwd=3,cex=1.5)

points(bugandenv[bugandenv$day==260,"dist"],bugandenv[bugandenv$day==260,"oligochaeta"],type="b",col="blue",pch=8,cex=1.5,lwd=3,lty=3)

points(bugandenv[bugandenv$day==336,"dist"],bugandenv[bugandenv$day==336,"oligochaeta"],type="b",col="green3",pch=17,cex=1.5,lwd=3)

points(bugandenv[bugandenv$day==518,"dist"],bugandenv[bugandenv$day==518,"oligochaeta"],type="b",col="red",pch=18,cex=2,lwd=3,lty=5)

legend("topright",inset=c(0,0),legend=c("Dec 2013", "April 2014", "Aug 2014", "Nov 2014","May 2015"),lty=1,lwd=2,col=c("black","gold2","blue","green3","red"),ncol=5,horiz=FALSE,cex=0.8,title="months")

abline(v=4,lty=2)

par(mar=c(5,5,4,2),cex=0.9)

plot(bugandenv[bugandenv$day==1,"dist"],bugandenv[bugandenv$day==1,"chironominae"],type = "b",ylim=c(0,400),pch=19,cex=1.5,xlab="Distance(km)",ylab="chironominae",main="chironominae plotted against spatial position",lwd=3,lty=4)

points(bugandenv[bugandenv$day==126,"dist"],bugandenv[wangenv$day==126,"chironominae"],type="b",col="gold2",pch=15,lwd=3,cex=1.5)

points(bugandenv[bugandenv$day==260,"dist"],bugandenv[bugandenv$day==260,"chironominae"],type="b",col="blue",pch=8,cex=1.5,lwd=3,lty=3)

points(bugandenv[bugandenv$day==336,"dist"],bugandenv[bugandenv$day==336,"chironominae"],type="b",col="green3",pch=17,cex=1.5,lwd=3)

points(bugandenv[bugandenv$day==518,"dist"],bugandenv[bugandenv$day==518,"chironominae"],type="b",col="red",pch=18,cex=2,lwd=3,lty=5)

legend("topright",inset=c(0,0),legend=c("Dec 2013", "April 2014", "Aug 2014", "Nov 2014","May 2015"),lty=1,lwd=2,col=c("black","gold2","blue","green3","red"),ncol=5,horiz=FALSE,cex=0.8,title="months")

abline(v=4,lty=2)

par(mar=c(5,5,4,2),cex=0.9)

plot(bugandenv[bugandenv$day==1,"dist"],bugandenv[bugandenv$day==1,"psychodidae"],type = "b",ylim=c(0,30),pch=19,cex=1.5,xlab="Distance(km)",ylab="psychodidae",main="psychodidae plotted against spatial position",lwd=3,lty=4)

points(bugandenv[bugandenv$day==126,"dist"],bugandenv[wangenv$day==126,"psychodidae"],type="b",col="gold2",pch=15,lwd=3,cex=1.5)

points(bugandenv[bugandenv$day==260,"dist"],bugandenv[bugandenv$day==260,"psychodidae"],type="b",col="blue",pch=8,cex=1.5,lwd=3,lty=3)

points(bugandenv[bugandenv$day==336,"dist"],bugandenv[bugandenv$day==336,"psychodidae"],type="b",col="green3",pch=17,cex=1.5,lwd=3)

points(bugandenv[bugandenv$day==518,"dist"],bugandenv[bugandenv$day==518,"psychodidae"],type="b",col="red",pch=18,cex=2,lwd=3,lty=5)

legend("topright",inset=c(0,0),legend=c("Dec 2013", "April 2014", "Aug 2014", "Nov 2014","May 2015"),lty=1,lwd=2,col=c("black","gold2","blue","green3","red"),ncol=5,horiz=FALSE,cex=0.8,title="months")

abline(v=4,lty=2)

par(mar=c(5,5,4,2),cex=0.9)

plot(bugandenv[bugandenv$day==1,"dist"],bugandenv[bugandenv$day==1,"ancylidae"],type = "b",ylim=c(0,5),pch=19,cex=1.5,xlab="Distance(km)",ylab="ancylidae",main="ancylidae plotted against spatial position",lwd=3,lty=4)

points(bugandenv[bugandenv$day==126,"dist"],bugandenv[wangenv$day==126,"ancylidae"],type="b",col="gold2",pch=15,lwd=3,cex=1.5)

points(bugandenv[bugandenv$day==260,"dist"],bugandenv[bugandenv$day==260,"ancylidae"],type="b",col="blue",pch=8,cex=1.5,lwd=3,lty=3)

points(bugandenv[bugandenv$day==336,"dist"],bugandenv[bugandenv$day==336,"ancylidae"],type="b",col="green3",pch=17,cex=1.5,lwd=3)

points(bugandenv[bugandenv$day==518,"dist"],bugandenv[bugandenv$day==518,"ancylidae"],type="b",col="red",pch=18,cex=2,lwd=3,lty=5)

legend("topright",inset=c(0,0),legend=c("Dec 2013", "April 2014", "Aug 2014", "Nov 2014","May 2015"),lty=1,lwd=2,col=c("black","gold2","blue","green3","red"),ncol=5,horiz=FALSE,cex=0.8,title="months")

abline(v=4,lty=2)

par(mar=c(5,5,4,2),cex=0.9)

plot(bugandenv[bugandenv$day==1,"dist"],bugandenv[bugandenv$day==1,"anostraca"],type = "b",ylim=c(0,5),pch=19,cex=1.5,xlab="Distance(km)",ylab="anostraca",main="anostraca plotted against spatial position",lwd=3,lty=4)

points(bugandenv[bugandenv$day==126,"dist"],bugandenv[wangenv$day==126,"anostraca"],type="b",col="gold2",pch=15,lwd=3,cex=1.5)

points(bugandenv[bugandenv$day==260,"dist"],bugandenv[bugandenv$day==260,"anostraca"],type="b",col="blue",pch=8,cex=1.5,lwd=3,lty=3)

points(bugandenv[bugandenv$day==336,"dist"],bugandenv[bugandenv$day==336,"anostraca"],type="b",col="green3",pch=17,cex=1.5,lwd=3)

points(bugandenv[bugandenv$day==518,"dist"],bugandenv[bugandenv$day==518,"anostraca"],type="b",col="red",pch=18,cex=2,lwd=3,lty=5)

legend("topright",inset=c(0,0),legend=c("Dec 2013", "April 2014", "Aug 2014", "Nov 2014","May 2015"),lty=1,lwd=2,col=c("black","gold2","blue","green3","red"),ncol=5,horiz=FALSE,cex=0.8,title="months")

abline(v=4,lty=2)

par(mar=c(5,5,4,2),cex=0.9)

plot(bugandenv[bugandenv$day==1,"dist"],bugandenv[bugandenv$day==1,"diamesinae"],type = "b",ylim=c(0,5),pch=19,cex=1.5,xlab="Distance(km)",ylab="diamesinae",main="diamesinae plotted against spatial position",lwd=3,lty=4)

points(bugandenv[bugandenv$day==126,"dist"],bugandenv[wangenv$day==126,"diamesinae"],type="b",col="gold2",pch=15,lwd=3,cex=1.5)

points(bugandenv[bugandenv$day==260,"dist"],bugandenv[bugandenv$day==260,"diamesinae"],type="b",col="blue",pch=8,cex=1.5,lwd=3,lty=3)

points(bugandenv[bugandenv$day==336,"dist"],bugandenv[bugandenv$day==336,"diamesinae"],type="b",col="green3",pch=17,cex=1.5,lwd=3)

points(bugandenv[bugandenv$day==518,"dist"],bugandenv[bugandenv$day==518,"diamesinae"],type="b",col="red",pch=18,cex=2,lwd=3,lty=5)

legend("topright",inset=c(0,0),legend=c("Dec 2013", "April 2014", "Aug 2014", "Nov 2014","May 2015"),lty=1,lwd=2,col=c("black","gold2","blue","green3","red"),ncol=5,horiz=FALSE,cex=0.8,title="months")

abline(v=4,lty=2)

par(mar=c(5,5,4,2),cex=0.9)

plot(bugandenv[bugandenv$day==1,"dist"],bugandenv[bugandenv$day==1,"hydrochidae"],type = "b",ylim=c(0,5),pch=19,cex=1.5,xlab="Distance(km)",ylab="hydrochidae",main="hydrochidae plotted against spatial position",lwd=3,lty=4)

points(bugandenv[bugandenv$day==126,"dist"],bugandenv[wangenv$day==126,"hydrochidae"],type="b",col="gold2",pch=15,lwd=3,cex=1.5)

points(bugandenv[bugandenv$day==260,"dist"],bugandenv[bugandenv$day==260,"hydrochidae"],type="b",col="blue",pch=8,cex=1.5,lwd=3,lty=3)

points(bugandenv[bugandenv$day==336,"dist"],bugandenv[bugandenv$day==336,"hydrochidae"],type="b",col="green3",pch=17,cex=1.5,lwd=3)

points(bugandenv[bugandenv$day==518,"dist"],bugandenv[bugandenv$day==518,"hydrochidae"],type="b",col="red",pch=18,cex=2,lwd=3,lty=5)

legend("topright",inset=c(0,0),legend=c("Dec 2013", "April 2014", "Aug 2014", "Nov 2014","May 2015"),lty=1,lwd=2,col=c("black","gold2","blue","green3","red"),ncol=5,horiz=FALSE,cex=0.8,title="months")

abline(v=4,lty=2)

par(mar=c(5,5,4,2),cex=0.9)

plot(bugandenv[bugandenv$day==1,"dist"],bugandenv[bugandenv$day==1,"hydrophilidae"],type = "b",ylim=c(0,5),pch=19,cex=1.5,xlab="Distance(km)",ylab="hydrophilidae",main="hydrophilidae plotted against spatial position",lwd=3,lty=4)

points(bugandenv[bugandenv$day==126,"dist"],bugandenv[wangenv$day==126,"hydrophilidae"],type="b",col="gold2",pch=15,lwd=3,cex=1.5)

points(bugandenv[bugandenv$day==260,"dist"],bugandenv[bugandenv$day==260,"hydrophilidae"],type="b",col="blue",pch=8,cex=1.5,lwd=3,lty=3)

points(bugandenv[bugandenv$day==336,"dist"],bugandenv[bugandenv$day==336,"hydrophilidae"],type="b",col="green3",pch=17,cex=1.5,lwd=3)

points(bugandenv[bugandenv$day==518,"dist"],bugandenv[bugandenv$day==518,"hydrophilidae"],type="b",col="red",pch=18,cex=2,lwd=3,lty=5)

legend("topright",inset=c(0,0),legend=c("Dec 2013", "April 2014", "Aug 2014", "Nov 2014","May 2015"),lty=1,lwd=2,col=c("black","gold2","blue","green3","red"),ncol=5,horiz=FALSE,cex=0.8,title="months")

abline(v=4,lty=2)

par(mar=c(5,5,4,2),cex=0.9)

plot(bugandenv[bugandenv$day==1,"dist"],bugandenv[bugandenv$day==1,"muscidae"],type = "b",ylim=c(0,5),pch=19,cex=1.5,xlab="Distance(km)",ylab="muscidae",main="muscidae plotted against spatial position",lwd=3,lty=4)

points(bugandenv[bugandenv$day==126,"dist"],bugandenv[wangenv$day==126,"muscidae"],type="b",col="gold2",pch=15,lwd=3,cex=1.5)

points(bugandenv[bugandenv$day==260,"dist"],bugandenv[bugandenv$day==260,"muscidae"],type="b",col="blue",pch=8,cex=1.5,lwd=3,lty=3)

points(bugandenv[bugandenv$day==336,"dist"],bugandenv[bugandenv$day==336,"muscidae"],type="b",col="green3",pch=17,cex=1.5,lwd=3)

points(bugandenv[bugandenv$day==518,"dist"],bugandenv[bugandenv$day==518,"muscidae"],type="b",col="red",pch=18,cex=2,lwd=3,lty=5)

legend("topright",inset=c(0,0),legend=c("Dec 2013", "April 2014", "Aug 2014", "Nov 2014","May 2015"),lty=1,lwd=2,col=c("black","gold2","blue","green3","red"),ncol=5,horiz=FALSE,cex=0.8,title="months")

abline(v=4,lty=2)

par(mar=c(5,5,4,2),cex=0.9)

plot(bugandenv[bugandenv$day==1,"dist"],bugandenv[bugandenv$day==1,"physidae"],type = "b",ylim=c(0,5),pch=19,cex=1.5,xlab="Distance(km)",ylab="physidae",main="physidae plotted against spatial position",lwd=3,lty=4)

points(bugandenv[bugandenv$day==126,"dist"],bugandenv[wangenv$day==126,"physidae"],type="b",col="gold2",pch=15,lwd=3,cex=1.5)

points(bugandenv[bugandenv$day==260,"dist"],bugandenv[bugandenv$day==260,"physidae"],type="b",col="blue",pch=8,cex=1.5,lwd=3,lty=3)

points(bugandenv[bugandenv$day==336,"dist"],bugandenv[bugandenv$day==336,"physidae"],type="b",col="green3",pch=17,cex=1.5,lwd=3)

points(bugandenv[bugandenv$day==518,"dist"],bugandenv[bugandenv$day==518,"physidae"],type="b",col="red",pch=18,cex=2,lwd=3,lty=5)

legend("topright",inset=c(0,0),legend=c("Dec 2013", "April 2014", "Aug 2014", "Nov 2014","May 2015"),lty=1,lwd=2,col=c("black","gold2","blue","green3","red"),ncol=5,horiz=FALSE,cex=0.8,title="months")

abline(v=4,lty=2)

par(mar=c(5,5,4,2),cex=0.9)

plot(bugandenv[bugandenv$day==1,"dist"],bugandenv[bugandenv$day==1,"podonominae"],type = "b",ylim=c(0,10),pch=19,cex=1.5,xlab="Distance(km)",ylab="podonominae",main="podonominae plotted against spatial position",lwd=3,lty=4)

points(bugandenv[bugandenv$day==126,"dist"],bugandenv[wangenv$day==126,"podonominae"],type="b",col="gold2",pch=15,lwd=3,cex=1.5)

points(bugandenv[bugandenv$day==260,"dist"],bugandenv[bugandenv$day==260,"podonominae"],type="b",col="blue",pch=8,cex=1.5,lwd=3,lty=3)

points(bugandenv[bugandenv$day==336,"dist"],bugandenv[bugandenv$day==336,"podonominae"],type="b",col="green3",pch=17,cex=1.5,lwd=3)

points(bugandenv[bugandenv$day==518,"dist"],bugandenv[bugandenv$day==518,"podonominae"],type="b",col="red",pch=18,cex=2,lwd=3,lty=5)

legend("topright",inset=c(0,0),legend=c("Dec 2013", "April 2014", "Aug 2014", "Nov 2014","May 2015"),lty=1,lwd=2,col=c("black","gold2","blue","green3","red"),ncol=5,horiz=FALSE,cex=0.8,title="months")

abline(v=4,lty=2)

par(mar=c(5,5,4,2),cex=0.9)

plot(bugandenv[bugandenv$day==1,"dist"],bugandenv[bugandenv$day==1,"psychodidae"],type = "b",ylim=c(0,40),pch=19,cex=1.5,xlab="Distance(km)",ylab="psychodidae",main="psychodidae plotted against spatial position",lwd=3,lty=4)

points(bugandenv[bugandenv$day==126,"dist"],bugandenv[wangenv$day==126,"psychodidae"],type="b",col="gold2",pch=15,lwd=3,cex=1.5)

points(bugandenv[bugandenv$day==260,"dist"],bugandenv[bugandenv$day==260,"psychodidae"],type="b",col="blue",pch=8,cex=1.5,lwd=3,lty=3)

points(bugandenv[bugandenv$day==336,"dist"],bugandenv[bugandenv$day==336,"psychodidae"],type="b",col="green3",pch=17,cex=1.5,lwd=3)

points(bugandenv[bugandenv$day==518,"dist"],bugandenv[bugandenv$day==518,"psychodidae"],type="b",col="red",pch=18,cex=2,lwd=3,lty=5)

legend("topright",inset=c(0,0),legend=c("Dec 2013", "April 2014", "Aug 2014", "Nov 2014","May 2015"),lty=1,lwd=2,col=c("black","gold2","blue","green3","red"),ncol=5,horiz=FALSE,cex=0.8,title="months")

abline(v=4,lty=2)

par(mar=c(5,5,4,2),cex=0.9)

plot(bugandenv[bugandenv$day==1,"dist"],bugandenv[bugandenv$day==1,"scitidae"],type = "b",ylim=c(0,5),pch=19,cex=1.5,xlab="Distance(km)",ylab="scitidae",main="scitidae plotted against spatial position",lwd=3,lty=4)

points(bugandenv[bugandenv$day==126,"dist"],bugandenv[wangenv$day==126,"scitidae"],type="b",col="gold2",pch=15,lwd=3,cex=1.5)

points(bugandenv[bugandenv$day==260,"dist"],bugandenv[bugandenv$day==260,"scitidae"],type="b",col="blue",pch=8,cex=1.5,lwd=3,lty=3)

points(bugandenv[bugandenv$day==336,"dist"],bugandenv[bugandenv$day==336,"scitidae"],type="b",col="green3",pch=17,cex=1.5,lwd=3)

points(bugandenv[bugandenv$day==518,"dist"],bugandenv[bugandenv$day==518,"scitidae"],type="b",col="red",pch=18,cex=2,lwd=3,lty=5)

legend("topright",inset=c(0,0),legend=c("Dec 2013", "April 2014", "Aug 2014", "Nov 2014","May 2015"),lty=1,lwd=2,col=c("black","gold2","blue","green3","red"),ncol=5,horiz=FALSE,cex=0.8,title="months")

abline(v=4,lty=2)

par(mar=c(5,5,4,2),cex=0.9)

plot(bugandenv[bugandenv$day==1,"dist"],bugandenv[bugandenv$day==1,"staphylinidae"],type = "b",ylim=c(0,5),pch=19,cex=1.5,xlab="Distance(km)",ylab="staphylinidae",main="staphylinidae plotted against spatial position",lwd=3,lty=4)

points(bugandenv[bugandenv$day==126,"dist"],bugandenv[wangenv$day==126,"staphylinidae"],type="b",col="gold2",pch=15,lwd=3,cex=1.5)

points(bugandenv[bugandenv$day==260,"dist"],bugandenv[bugandenv$day==260,"staphylinidae"],type="b",col="blue",pch=8,cex=1.5,lwd=3,lty=3)

points(bugandenv[bugandenv$day==336,"dist"],bugandenv[bugandenv$day==336,"staphylinidae"],type="b",col="green3",pch=17,cex=1.5,lwd=3)

points(bugandenv[bugandenv$day==518,"dist"],bugandenv[bugandenv$day==518,"staphylinidae"],type="b",col="red",pch=18,cex=2,lwd=3,lty=5)

legend("topright",inset=c(0,0),legend=c("Dec 2013", "April 2014", "Aug 2014", "Nov 2014","May 2015"),lty=1,lwd=2,col=c("black","gold2","blue","green3","red"),ncol=5,horiz=FALSE,cex=0.8,title="months")

abline(v=4,lty=2)

par(mar=c(5,5,4,2),cex=0.9)

plot(bugandenv[bugandenv$day==1,"dist"],bugandenv[bugandenv$day==1,"culicidae"],type = "b",ylim=c(0,5),pch=19,cex=1.5,xlab="Distance(km)",ylab="culicidae",main="culicidae plotted against spatial position",lwd=3,lty=4)

points(bugandenv[bugandenv$day==126,"dist"],bugandenv[wangenv$day==126,"culicidae"],type="b",col="gold2",pch=15,lwd=3,cex=1.5)

points(bugandenv[bugandenv$day==260,"dist"],bugandenv[bugandenv$day==260,"culicidae"],type="b",col="blue",pch=8,cex=1.5,lwd=3,lty=3)

points(bugandenv[bugandenv$day==336,"dist"],bugandenv[bugandenv$day==336,"culicidae"],type="b",col="green3",pch=17,cex=1.5,lwd=3)

points(bugandenv[bugandenv$day==518,"dist"],bugandenv[bugandenv$day==518,"culicidae"],type="b",col="red",pch=18,cex=2,lwd=3,lty=5)

legend("topright",inset=c(0,0),legend=c("Dec 2013", "April 2014", "Aug 2014", "Nov 2014","May 2015"),lty=1,lwd=2,col=c("black","gold2","blue","green3","red"),ncol=5,horiz=FALSE,cex=0.8,title="months")

abline(v=4,lty=2)

par(mar=c(5,5,4,2),cex=0.9)

plot(bugandenv[bugandenv$day==1,"dist"],bugandenv[bugandenv$day==1,"curculionidae"],type = "b",ylim=c(0,5),pch=19,cex=1.5,xlab="Distance(km)",ylab="curculionidae",main="curculionidae plotted against spatial position",lwd=3,lty=4)

points(bugandenv[bugandenv$day==126,"dist"],bugandenv[wangenv$day==126,"curculionidae"],type="b",col="gold2",pch=15,lwd=3,cex=1.5)

points(bugandenv[bugandenv$day==260,"dist"],bugandenv[bugandenv$day==260,"curculionidae"],type="b",col="blue",pch=8,cex=1.5,lwd=3,lty=3)

points(bugandenv[bugandenv$day==336,"dist"],bugandenv[bugandenv$day==336,"curculionidae"],type="b",col="green3",pch=17,cex=1.5,lwd=3)

points(bugandenv[bugandenv$day==518,"dist"],bugandenv[bugandenv$day==518,"curculionidae"],type="b",col="red",pch=18,cex=2,lwd=3,lty=5)

legend("topright",inset=c(0,0),legend=c("Dec 2013", "April 2014", "Aug 2014", "Nov 2014","May 2015"),lty=1,lwd=2,col=c("black","gold2","blue","green3","red"),ncol=5,horiz=FALSE,cex=0.8,title="months")

abline(v=4,lty=2)

######### heat map

sptree<-hclust(vegdist(t((wangbug)^0.25), "raup"), "average")

tiff(file="heatpco1_ungrouped.tif",width=5,height=5,units="in",pointsize = 12,bg = "transparent",res=800,compression="lzw")

tabasco((wangbug)^0.25,use=pwangbug[,"pco1"],sp.ind=sptree,main="PCO1",cex.main=0.2,cexRow=0.3,cexCol=0.5)

dev.off()

cl <- function(x) quantile(x,seq(0,1,0.1))

apply(pwangbug[,c("pco1","pco2","pco3","pco4","pco5","pco6")],2,cl)

breaks1<-cut(pwangbug[,"pco1"],breaks=quantile(pwangbug[,"pco1"],seq(0,1,0.1)))

wangbug.agg1<-aggregate(wangbug,by=list(breaks1),mean)

dim(wangbug.agg1)

attributes(wangbug.agg1)

wangbug.agg1$Group.1

breaks2<-cut(pwangbug[,"pco2"],breaks=quantile(pwangbug[,"pco2"],seq(0,1,0.1)))

wangbug.agg2<-aggregate(wangbug,by=list(breaks2),mean)

breaks3<-cut(pwangbug[,"pco3"],breaks=quantile(pwangbug[,"pco3"],seq(0,1,0.1)))

wangbug.agg3<-aggregate(wangbug,by=list(breaks3),mean)

breaks4<-cut(pwangbug[,"pco4"],breaks=quantile(pwangbug[,"pco4"],seq(0,1,0.1)))

wangbug.agg4<-aggregate(wangbug,by=list(breaks4),mean)

breaks5<-cut(pwangbug[,"pco5"],breaks=quantile(pwangbug[,"pco5"],seq(0,1,0.1)))

wangbug.agg5<-aggregate(wangbug,by=list(breaks5),mean)

breaks6<-cut(pwangbug[,"pco6"],breaks=quantile(pwangbug[,"pco6"],seq(0,1,0.1)))

wangbug.agg6<-aggregate(wangbug,by=list(breaks6),mean)

#***** Figure 5 *****#

tiff(file="Figure 5",width=5,height=5,units="in",pointsize = 12,bg = "transparent",res=1200,compression="lzw")

tabasco((as.data.frame(wangbug.agg1[,-1]))^0.25,use=as.numeric(rownames(wangbug.agg1)),sp.ind=sptree,labCol=wangbug.agg1$Group.1,main="PCO1",cex.main=0.2,cexRow=0.6,cexCol=0.5)

dev.off()

tiff(file="heatpco2.tif",width=5,height=5,units="in",pointsize = 12,bg = "transparent",res=1200,compression="lzw")

tabasco((as.data.frame(wangbug.agg2[,-1]))^0.25,use=as.numeric(rownames(wangbug.agg2)),sp.ind=sptree,labCol=wangbug.agg2$Group.1,main="PCO2",cex.main=0.2,cexRow=0.6,cexCol=0.5)

dev.off()

tiff(file="heatpco3.tif",width=5,height=5,units="in",pointsize = 12,bg = "transparent",res=1200,compression="lzw")

tabasco((as.data.frame(wangbug.agg3[,-1]))^0.25,use=as.numeric(rownames(wangbug.agg3)),sp.ind=sptree,labCol=wangbug.agg3$Group.1,main="PCO3",cex.main=0.2,cexRow=0.6,cexCol=0.5)

dev.off()

tiff(file="heatpco4.tif",width=5,height=5,units="in",pointsize = 12,bg = "transparent",res=1200,compression="lzw")

tabasco((as.data.frame(wangbug.agg4[,-1]))^0.25,use=as.numeric(rownames(wangbug.agg4)),sp.ind=sptree,labCol=wangbug.agg4$Group.1,main="PCO4",cex.main=0.2,cexRow=0.6,cexCol=0.5)

dev.off()

tiff(file="heatpco5.tif",width=5,height=5,units="in",pointsize = 12,bg = "transparent",res=1200,compression="lzw")

tabasco((as.data.frame(wangbug.agg5[,-1]))^0.25,use=as.numeric(rownames(wangbug.agg5)),sp.ind=sptree,labCol=wangbug.agg5$Group.1,main="PCO5",cex.main=0.2,cexRow=0.6,cexCol=0.5)

dev.off()

tiff(file="heatpco6.tif",width=5,height=5,units="in",pointsize = 12,bg = "transparent",res=1200,compression="lzw")

tabasco((as.data.frame(wangbug.agg6[,-1]))^0.25,use=as.numeric(rownames(wangbug.agg5)),sp.ind=sptree,labCol=wangbug.agg5$Group.1,main="PCO5",cex.main=0.2,cexRow=0.6,cexCol=0.5)

dev.off()

temp1<-cbind(breaks1,wangbug[,"baetidae"])

temp1<-temp1[order(breaks1),]

colnames(temp1)<-c("a","b")

edit(temp1)

temp2<-aggregate(temp1[,"b"],by=list(temp[,"a"]),mean)

(wangbug.agg1[,"baetidae"])^0.25

#*********#

maxbug<- apply(wangbug, 2, max)

head(maxbug)

n1 <- names(which(maxbug < 5))

n1

wangbug1 <- wangbug[, -which(names(wangbug) %in% n1)]

#edit(wangbug1)

dim(wangbug1)

dim(wangbug)

sptree<-hclust(vegdist(t((wangbug1)^0.25), "raup"), "average")

cl <- function(x) quantile(x,seq(0,1,0.1))

apply(pwangbug[,c("pco1","pco2","pco3","pco4","pco5","pco6")],2,cl)

breaks1<-cut(pwangbug[,"pco1"],breaks=quantile(pwangbug[,"pco1"],seq(0,1,0.1)))

wangbug.agg1<-aggregate(wangbug1,by=list(breaks1),mean)

dim(wangbug.agg1)

attributes(wangbug.agg1)

wangbug.agg1$Group.1

breaks2<-cut(pwangbug[,"pco2"],breaks=quantile(pwangbug[,"pco2"],seq(0,1,0.1)))

wangbug.agg2<-aggregate(wangbug,by=list(breaks2),mean)

breaks3<-cut(pwangbug[,"pco3"],breaks=quantile(pwangbug[,"pco3"],seq(0,1,0.1)))

wangbug.agg3<-aggregate(wangbug,by=list(breaks3),mean)

breaks4<-cut(pwangbug[,"pco4"],breaks=quantile(pwangbug[,"pco4"],seq(0,1,0.1)))

wangbug.agg4<-aggregate(wangbug,by=list(breaks4),mean)

breaks5<-cut(pwangbug[,"pco5"],breaks=quantile(pwangbug[,"pco5"],seq(0,1,0.1)))

wangbug.agg5<-aggregate(wangbug,by=list(breaks5),mean)

breaks6<-cut(pwangbug[,"pco6"],breaks=quantile(pwangbug[,"pco6"],seq(0,1,0.1)))

wangbug.agg6<-aggregate(wangbug,by=list(breaks6),mean)

tiff(file="heatmap.jpg",width=5,height=5,units="in",pointsize = 12,bg = "transparent",res=1200,compression="lzw")

tabasco((as.data.frame(wangbug.agg1[,-1]))^0.25,use=as.numeric(rownames(wangbug.agg1)),sp.ind=sptree,labCol=wangbug.agg1$Group.1,main="PCO1",cex.main=0.2,cexRow=0.6,cexCol=0.5)

dev.off()

##########The relationships between PCOs and Diversity indices

#PCO1

plot(pwangbug[,"pco1"],diversity[,"shannon"])

plot(pwangbug[,"pco1"],diversity[,"signal"])

plot(pwangbug[,"pco1"],diversity[,"taxarich"])

plot(pwangbug[,"pco1"],diversity[,"abundance"])

plot(pwangbug[,"pco1"],diversity[,"eptnum"])

plot(pwangbug[,"pco1"],diversity[,"eptrich"])

pwangdiver<-cbind(diversity,pwangbug)

model1 <- lm(pwangdiver$pco1 ~ pwangdiver$shannon)

summary(model1)

plot(pwangdiver$pco1 ~ pwangdiver$shannon)

abline(model1)

model1 <- lm(pwangdiver$pco1 ~ pwangdiver$signal)

summary(model1)

plot(pwangdiver$pco1 ~ pwangdiver$signal)

abline(model1)

model1 <- lm(pwangdiver$pco1 ~ pwangdiver$taxarich)

summary(model1)

plot(pwangdiver$pco1 ~ pwangdiver$taxarich)

abline(model1)

model1 <- lm(pwangdiver$pco1 ~ pwangdiver$abundance)

summary(model1)

plot(pwangdiver$pco1 ~ pwangdiver$abundance)

abline(model1)

model1 <- lm(pwangdiver$pco1 ~ pwangdiver$eptnum)

summary(model1)

plot(pwangdiver$pco1 ~ pwangdiver$eptnum)

abline(model1)

model1 <- lm(pwangdiver$pco1 ~ pwangdiver$eptrich)

summary(model1)

plot(pwangdiver$pco1 ~ pwangdiver$eptrich)

abline(model1)wangwang<-cbind(wangenv,wangbug)

wangenv5<-cbind(wangwang,pwangbug)

plot(pwangbug[,"pco1"],wangbug[,"gripopterygidae"])

pwangdiver<-cbind(diversity,pwangbug)

model1 <- lm(pwangdiver$pco1 ~ wangbug$gripopterygidae)

summary(model1)

plot(pwangdiver$pco1 ~ wangbug$gripopterygidae)

abline(model1)

plot(pwangbug[,"pco1"],wangbug[,"oligochaeta"])

model1 <- lm(pwangdiver$pco1 ~ wangbug$oligochaeta)

summary(model1)

plot(pwangdiver$pco1 ~ wangbug$oligochaeta)

abline(model1)

plot(pwangbug[,"pco1"],wangbug[,"simulidae"])

model1 <- lm(pwangdiver$pco1 ~ wangbug$simulidae)

summary(model1)

plot(pwangdiver$pco1 ~ wangbug$simulidae)

abline(model1)

plot(pwangbug[,"pco1"],wangbug[,"chironominae"])

model1 <- lm(pwangdiver$pco1 ~ wangbug$chironominae)

summary(model1)

plot(pwangdiver$pco1 ~ wangbug$chironominae)

abline(model1)

#pco2

plot(pwangbug[,"pco2"],diversity[,"shannon"])

plot(pwangbug[,"pco2"],diversity[,"signal"])

plot(pwangbug[,"pco2"],diversity[,"taxarich"])

plot(pwangbug[,"pco2"],diversity[,"abundance"])

plot(pwangbug[,"pco2"],diversity[,"eptnum"])

plot(pwangbug[,"pco2"],diversity[,"eptrich"])

pwangdiver<-cbind(diversity,pwangbug)

model1 <- lm(pwangdiver$pco2 ~ pwangdiver$shannon)

summary(model1)

plot(pwangdiver$pco2 ~ pwangdiver$shannon)

abline(model1)

model1 <- lm(pwangdiver$pco2 ~ pwangdiver$signal)

summary(model1)

plot(pwangdiver$pco2 ~ pwangdiver$signal)

abline(model1)

model1 <- lm(pwangdiver$pco2 ~ pwangdiver$taxarich)

summary(model1)

plot(pwangdiver$pco2 ~ pwangdiver$taxarich)

abline(model1)

model1 <- lm(pwangdiver$pco2 ~ pwangdiver$abundance)

summary(model1)

plot(pwangdiver$pco2 ~ pwangdiver$abundance)

abline(model1)

model1 <- lm(pwangdiver$pco2 ~ pwangdiver$eptnum)

summary(model1)

plot(pwangdiver$pco2 ~ pwangdiver$eptnum)

abline(model1)

model1 <- lm(pwangdiver$pco2 ~ pwangdiver$eptrich)

summary(model1)

plot(pwangdiver$pco2 ~ pwangdiver$eptrich)

abline(model1)

plot(pwangbug[,"pco2"],wangbug[,"gripopterygidae"]) # Checks on aggregate process and heat maps

pwangdiver<-cbind(diversity,pwangbug)

model1 <- lm(pwangdiver$pco2 ~ wangbug$gripopterygidae)

summary(model1)

plot(pwangdiver$pco2 ~ wangbug$gripopterygidae)

abline(model1)

plot(pwangbug[,"pco2"],wangbug[,"oligochaeta"]) # Checks on aggregate process and heat maps

model1 <- lm(pwangdiver$pco2 ~ wangbug$oligochaeta)

summary(model1)

plot(pwangdiver$pco2~ wangbug$oligochaeta)

abline(model1)

plot(pwangbug[,"pco2"],wangbug[,"simulidae"]) # Checks on aggregate process and heat maps

model1 <- lm(pwangdiver$pco2 ~ wangbug$simulidae)

summary(model1)

plot(pwangdiver$pco2 ~ wangbug$simulidae)

abline(model1)

plot(pwangbug[,"pco2"],wangbug[,"chironominae"]) # Checks on aggregate process and heat maps

model1 <- lm(pwangdiver$pco2 ~ wangbug$chironominae)

summary(model1)

plot(pwangdiver$pco2 ~ wangbug$chironominae)

abline(model1)

#PCO3

plot(pwangbug[,"pco3"],diversity[,"shannon"])

plot(pwangbug[,"pco3"],diversity[,"signal"])

plot(pwangbug[,"pco3"],diversity[,"taxarich"])

plot(pwangbug[,"pco3"],diversity[,"abundance"])

plot(pwangbug[,"pco3"],diversity[,"eptnum"])

plot(pwangbug[,"pco3"],diversity[,"eptrich"])

model1 <- lm(pwangdiver$pco3 ~ pwangdiver$shannon)

summary(model1)

plot(pwangdiver$pco3 ~ pwangdiver$shannon)

abline(model1)

model1 <- lm(pwangdiver$pco3 ~ pwangdiver$signal)

summary(model1)

plot(pwangdiver$pco3 ~ pwangdiver$signal)

abline(model1)

model1 <- lm(pwangdiver$pco3 ~ pwangdiver$taxarich)

summary(model1)

plot(pwangdiver$pco3 ~ pwangdiver$taxarich)

abline(model1)

model1 <- lm(pwangdiver$pco3 ~ pwangdiver$abundance)

summary(model1)

plot(pwangdiver$pco3 ~ pwangdiver$abundance)

abline(model1)

model1 <- lm(pwangdiver$pco3 ~ pwangdiver$eptnum)

summary(model1)

plot(pwangdiver$pco3 ~ pwangdiver$eptnum)

abline(model1)

model1 <- lm(pwangdiver$pco3 ~ pwangdiver$eptrich)

summary(model1)

plot(pwangdiver$pco3 ~ pwangdiver$eptrich)

abline(model1)

plot(pwangbug[,"pco3"],wangbug[,"gripopterygidae"]) # Checks on aggregate process and heat maps

pwangdiver<-cbind(diversity,pwangbug)

model1 <- lm(pwangdiver$pco3 ~ wangbug$gripopterygidae)

summary(model1)

plot(pwangdiver$pco3 ~ wangbug$gripopterygidae)

abline(model1)

plot(pwangbug[,"pco3"],wangbug[,"oligochaeta"]) # Checks on aggregate process and heat maps

model1 <- lm(pwangdiver$pco3 ~ wangbug$oligochaeta)

summary(model1)

plot(pwangdiver$pco3~ wangbug$oligochaeta)

abline(model1)

plot(pwangbug[,"pco3"],wangbug[,"simulidae"]) # Checks on aggregate process and heat maps

model1 <- lm(pwangdiver$pco3 ~ wangbug$simulidae)

summary(model1)

plot(pwangdiver$pco3 ~ wangbug$simulidae)

abline(model1)

plot(pwangbug[,"pco3"],wangbug[,"chironominae"]) # Checks on aggregate process and heat maps

model1 <- lm(pwangdiver$pco3 ~ wangbug$chironominae)

summary(model1)

plot(pwangdiver$pco3 ~ wangbug$chironominae)

abline(model1)

#PCO4

plot(pwangbug[,"pco4"],diversity[,"shannon"])

plot(pwangbug[,"pco4"],diversity[,"signal"])

plot(pwangbug[,"pco4"],diversity[,"taxarich"])

plot(pwangbug[,"pco4"],diversity[,"abundance"])

plot(pwangbug[,"pco4"],diversity[,"eptnum"])

plot(pwangbug[,"pco4"],diversity[,"eptrich"])

model1 <- lm(pwangdiver$pco4 ~ pwangdiver$shannon)

summary(model1)

plot(pwangdiver$pco4 ~ pwangdiver$shannon)

abline(model1)

model1 <- lm(pwangdiver$pco4 ~ pwangdiver$signal)

summary(model1)

plot(pwangdiver$pco4 ~ pwangdiver$signal)

abline(model1)

model1 <- lm(pwangdiver$pco4 ~ pwangdiver$taxarich)

summary(model1)

plot(pwangdiver$pco4 ~ pwangdiver$taxarich)

abline(model1)

model1 <- lm(pwangdiver$pco4 ~ pwangdiver$abundance)

summary(model1)

plot(pwangdiver$pco4 ~ pwangdiver$abundance)

abline(model1)

model1 <- lm(pwangdiver$pco4 ~ pwangdiver$eptnum)

summary(model1)

plot(pwangdiver$pco4 ~ pwangdiver$eptnum)

abline(model1)

model1 <- lm(pwangdiver$pco4 ~ pwangdiver$eptrich)

summary(model1)

plot(pwangdiver$pco4 ~ pwangdiver$eptrich)

abline(model1)

plot(pwangbug[,"pco4"],wangbug[,"gripopterygidae"]) # Checks on aggregate process and heat maps

model1 <- lm(pwangdiver$pco4 ~ wangbug$gripopterygidae)

summary(model1)

plot(pwangdiver$pco4 ~ wangbug$gripopterygidae)

abline(model1)

plot(pwangbug[,"pco4"],wangbug[,"oligochaeta"]) # Checks on aggregate process and heat maps

model1 <- lm(pwangdiver$pco4 ~ wangbug$oligochaeta)

summary(model1)

plot(pwangdiver$pco4~ wangbug$oligochaeta)

abline(model1)

plot(pwangbug[,"pco4"],wangbug[,"simulidae"]) # Checks on aggregate process and heat maps

model1 <- lm(pwangdiver$pco4 ~ wangbug$simulidae)

summary(model1)

plot(pwangdiver$pco4 ~ wangbug$simulidae)

abline(model1)

plot(pwangbug[,"pco4"],wangbug[,"chironominae"]) # Checks on aggregate process and heat maps

model1 <- lm(pwangdiver$pco4 ~ wangbug$chironominae)

summary(model1)

plot(pwangdiver$pco4 ~ wangbug$chironominae)

abline(model1)

#PCO5

plot(pwangbug[,"pco5"],diversity[,"shannon"])

plot(pwangbug[,"pco5"],diversity[,"signal"])

plot(pwangbug[,"pco5"],diversity[,"taxarich"])

plot(pwangbug[,"pco5"],diversity[,"abundance"])

plot(pwangbug[,"pco5"],diversity[,"eptnum"])

plot(pwangbug[,"pco5"],diversity[,"eptrich"])

model1 <- lm(pwangdiver$pco5 ~ pwangdiver$shannon)

summary(model1)

plot(pwangdiver$pco5 ~ pwangdiver$shannon)

abline(model1)

model1 <- lm(pwangdiver$pco5 ~ pwangdiver$signal)

summary(model1)

plot(pwangdiver$pco5 ~ pwangdiver$signal)

abline(model1)

model1 <- lm(pwangdiver$pco5 ~ pwangdiver$taxarich)

summary(model1)

plot(pwangdiver$pco5 ~ pwangdiver$taxarich)

abline(model1)

model1 <- lm(pwangdiver$pco5 ~ pwangdiver$abundance)

summary(model1)

plot(pwangdiver$pco5 ~ pwangdiver$abundance)

abline(model1)

model1 <- lm(pwangdiver$pco5 ~ pwangdiver$eptnum)

summary(model1)

plot(pwangdiver$pco5 ~ pwangdiver$eptnum)

abline(model1)

model1 <- lm(pwangdiver$pco4 ~ pwangdiver$eptrich)

summary(model1)

plot(pwangdiver$pco4 ~ pwangdiver$eptrich)

abline(model1)

#PCO6

plot(pwangbug[,"pco6"],diversity[,"shannon"])

plot(pwangbug[,"pco6"],diversity[,"signal"])

plot(pwangbug[,"pco6"],diversity[,"taxarich"])

plot(pwangbug[,"pco6"],diversity[,"abundance"])

plot(pwangbug[,"pco6"],diversity[,"eptnum"])

plot(pwangbug[,"pco6"],diversity[,"eptrich"])

model1 <- lm(pwangdiver$pco6 ~ pwangdiver$shannon)

summary(model1)

plot(pwangdiver$pco6 ~ pwangdiver$shannon)

abline(model1)

model1 <- lm(pwangdiver$pco6 ~ pwangdiver$signal)

summary(model1)

plot(pwangdiver$pco6 ~ pwangdiver$signal)

abline(model1)

model1 <- lm(pwangdiver$pco6~ pwangdiver$taxarich)

summary(model1)

plot(pwangdiver$pco6 ~ pwangdiver$taxarich)

abline(model1)

model1 <- lm(pwangdiver$pco6 ~ pwangdiver$abundance)

summary(model1)

plot(pwangdiver$pco6 ~ pwangdiver$abundance)

abline(model1)

model1 <- lm(pwangdiver$pco6 ~ pwangdiver$eptnum)

summary(model1)

plot(pwangdiver$pco6 ~ pwangdiver$eptnum)

abline(model1)

model1 <- lm(pwangdiver$pco6 ~ pwangdiver$eptrich)

summary(model1)

plot(pwangdiver$pco6 ~ pwangdiver$eptrich)

abline(model1)

###

###land use

alldata<-cbind(envpcopred,pwangbug)# combine environmental and landuse data,PCO matrix and predicted pco matrix

edit(alldata)

dim(alldata)

names(alldata)

edit(wangenv)

names(wangenv)

landuse1<-wangenv[c(11,12,13,14,15,16,17,18,19,20,21,22,23,24,25,26,27,28,35,37,38,43,45,47,48,53,54,56,57,62,64,66,67,72,73,75,76,81,82,84,90,91,93,94,99,100,102,103,108,109,111,112,117,118,120,121,126,127,129,130,135,136,138,139,144,177)]# land use(grazing, forest, creational and treatment)

landuse2<-log1p(landuse1)

edit(landuse2)

names(landuse2)

dim(landuse2)

landnut <- lm(log1p(no2) ~ eff+log1p(rec300)+log1p(treat100)+log1p(graz100)+log1p(treat60w), data=wangenv,na.action=na.exclude)

landnutstep <- step(landnut, direction='forward')

summary (landnutstep, corr = TRUE )

landnut <- lm(log1p(no3) ~ eff+log1p(rec300)+log1p(treat100)+log1p(graz100)+log1p(treat60w), data=landuse1,na.action=na.exclude)

landnutstep <- step(landnut, direction='forward')

summary (landnut, corr = TRUE )

landnut <- lm(log1p(nh3) ~ eff+log1p(rec300)+log1p(treat100)+log1p(graz100)+log1p(treat60w), data=landuse1,na.action=na.exclude)

landnutstep <- step(landnut, direction='forward')

summary (landnutstep, corr = TRUE )

landnut <- lm(log1p(nh3) ~ eff+log1p(rec300)+log1p(treat100)+log1p(graz100)+log1p(treat60w), data=landuse1,na.action=na.exclude)

landnutstep <- step(landnut, direction='forward')

summary (landnutstep, corr = TRUE )

landnut <- lm(log1p(op) ~ eff+log1p(rec300)+log1p(treat100)+log1p(graz100)+log1p(treat60w), data=landuse1,na.action=na.exclude)

landnut <- step(landnut, direction='forward')

summary (landnut, corr = TRUE )

landnut <- lm(log1p(tp) ~ eff+log1p(rec300)+log1p(treat100)+log1p(graz100)+log1p(treat60w), data=landuse1,na.action=na.exclude)

landnut <- step(landnut, direction='forward')

summary (landnut, corr = TRUE )

landnut <- lm(log1p(tn) ~ eff+log1p(rec300)+log1p(treat100)+log1p(graz100)+log1p(treat60w), data=landuse1,na.action=na.exclude)

landnut <- step(landnut, direction='forward')

summary (landnut, corr = TRUE )

landnut <- lm(log1p(sali) ~ eff+log1p(rec300)+log1p(treat100)+log1p(graz100)+log1p(treat60w), data=landuse1,na.action=na.exclude)

landnut <- step(landnut, direction='forward')

summary (landnut, corr = TRUE )

landnut <- lm(log1p(cond) ~ eff+log1p(rec300)+log1p(treat100)+log1p(graz100)+log1p(treat60w), data=landuse1,na.action=na.exclude)

landnut <- step(landnut, direction='forward')

summary (landnut, corr = TRUE )

landnut <- lm(log1p(turb) ~ eff+log1p(rec300)+log1p(treat100)+log1p(graz100)+log1p(treat60w), data=landuse1,na.action=na.exclude)

landnut <- step(landnut, direction='forward')

summary (landnut, corr = TRUE )

landnut <- lm(sqrt(cod) ~ eff+log1p(rec300)+log1p(treat100)+log1p(treat100w)+log1p(graz100)+log1p(treat60w)+log1p(treat60), data=landuse1,na.action=na.exclude)

landnut <- step(landnut, direction='forward')

summary (landnut, corr = TRUE )

landnut <- lm(log1p(toc) ~ eff+log1p(rec300)+log1p(treat100)+log1p(treat100w)+log1p(graz100)+log1p(treat60w)+log1p(treat60), data=landuse1,na.action=na.exclude)

landnut <- step(landnut, direction='forward')

summary (landnut, corr = TRUE )

landnut <- lm(log1p(zn) ~ eff+log1p(res1000)+log1p(treat1000)+log1p(treat500)+log1p(road1000)+log1p(treat1000)+log1p(ind), data=wangenv,na.action=na.exclude)

landnut <- step(landnut, direction='forward')

summary (landnut, corr = TRUE )

###land use scatterplot Matrices

alldata<-cbind(envpcopred,pwangbug)# combine environmental and landuse data,PCO matrix and predicted pco matrix

edit(alldata)

dim(alldata)

names(alldata)

edit(wangenv)

names(wangenv)

#############

tiff(file="landuse variables and environmental variables",width=14,height=14,units="in",pointsize = 12,bg ="transparent",res=800,compression="lzw")

par(mar=c(0.2,0.2,0.2,0.2),cex=0.6,cex.axis=0.8,las=1)

panel.cor <- function(x, y, digits = 2, cex.cor, ...)

{

usr <- par("usr"); on.exit(par(usr))

par(usr = c(0, 1, 0, 1))

# correlation coefficient

r <- cor(x, y)

txt <- format(c(r, 0.123456789), digits = digits)[1]

txt <- paste("r= ", txt, sep = "")

text(0.5, 0.8, txt)

# p-value calculation

p <- cor.test(x, y)$p.value

method=c("spearman")

txt2 <- format(c(p, 0.123456789), digits = digits)[1]

txt2 <- paste("p= ", txt2, sep = "")

if(p<0.01) txt2 <- paste("p= ", "<0.01", sep = "")

text(0.5, 0.4, txt2)

}

pairs(~cd+cr+cu+zn+graz+ind+res+for.+rec+road+treat,data=wangenv,upper.panel=panel.cor,pch=20)

pairs(~cd+cr+cu+zn+graz1000+ind1000+res1000+for1000+rec1000+road1000+treat1000,data=wangenv,upper.panel=panel.cor,pch=20)

pairs(~cd+cr+cu+zn+graz500+ind500+res500+for500+rec500+road500+treat500,data=wangenv,upper.panel=panel.cor,pch=20)

pairs(~cd+cr+cu+zn+graz300+ind300+res300+for300+rec300+road300+treat300,data=wangenv,upper.panel=panel.cor,pch=20)

pairs(~cd+cr+cu+zn+graz100+ind100+res100+for100+rec100+road100+treat100,data=wangenv,upper.panel=panel.cor,pch=20)

pairs(~cd+cr+cu+zn+graz60+ind60+for60+road60+treat60,data=wangenv,upper.panel=panel.cor,pch=20)

pairs(~cd+cr+cu+zn+graz500w+ind500w+res500w+for500w+rec500w+road500w+treat500w,data=wangenv,upper.panel=panel.cor,pch=20)

pairs(~cd+cr+cu+zn+graz400w+ind400w+res400w+for400w+rec400w+road400w+treat400w+tra400w,data=wangenv,upper.panel=panel.cor,pch=20)

pairs(~cd+cr+cu+zn+graz300w+ind300w+res300w+for300w+rec300w+road300w+treat300w+tra300w,data=wangenv,upper.panel=panel.cor,pch=20)

pairs(~cd+cr+cu+zn+graz200w+ind200w+res200w+for200w+rec200w+road200w+treat200w+tra200w,data=wangenv,upper.panel=panel.cor,pch=20)

pairs(~cd+cr+cu+zn+graz100w+ind100w+res100w+for100w+rec100w+road100w+treat100w,data=wangenv,upper.panel=panel.cor,pch=20)

pairs(~cd+cr+cu+zn+graz60w+ind60w+res60w+for60w+rec60w+road60w+treat60w,data=wangenv,upper.panel=panel.cor,pch=20)

pairs(~no3+no2+nh3+tn+tp+op+ph2+graz+res+for.+rec+road+treat,data=alldata,upper.panel=panel.cor,pch=20,na.action = na.omit)

pairs(~no3+no2+nh3+tn+tp+op+ph2+graz1000+ind1000+res1000+for1000+rec1000+road1000+treat1000,data=wangenv,upper.panel=panel.cor,pch=20,na.action = na.omit)

pairs(~no3+no2+nh3+ph2+tn+tp+op+graz500+ind500+res500+for500+rec500+road500+treat500,data=wangenv,upper.panel=panel.cor,pch=20,na.action = na.omit)

pairs(~no3+no2+nh3+ph2+tn+tp+op+graz300+ind300+res300+for300+rec300+road300+treat300,data=wangenv,upper.panel=panel.cor,pch=20,na.action = na.omit)

pairs(~no3+no2+nh3+ph2+tn+tp+op+graz100+ind100+res100+for100+rec100+road100+treat100,data=wangenv,upper.panel=panel.cor,pch=20,na.action = na.omit)

pairs(~no3+no2+nh3+ph2+tn+tp+op+graz60+ind60+res60+for60+road60+treat60,data=wangenv,upper.panel=panel.cor,pch=20,na.action = na.omit)

pairs(~no3+no2+nh3+ph2+tn+tp+op+graz500w+ind500w+res500w+for500w+rec500w+road500w+treat500w,data=wangenv,upper.panel=panel.cor,pch=20,na.action = na.omit)

pairs(~no3+no2+nh3+ph2+tn+tp+op+graz400w+ind400w+res400w+for400w+rec400w+road400w+treat400w+tra400w,data=wangenv,upper.panel=panel.cor,pch=20,na.action = na.omit)

pairs(~no3+no2+nh3+ph2+tn+tp+op+graz300w+ind300w+res300w+for300w+rec300w+road300w+treat300w+tra300w,data=wangenv,upper.panel=panel.cor,pch=20,na.action = na.omit)

pairs(~no3+no2+nh3+ph2+tn+tp+op+graz200w+ind200w+res200w+for200w+rec200w+road200w+treat200w+tra200w,data=wangenv,upper.panel=panel.cor,pch=20,na.action = na.omit)

pairs(~no3+no2+nh3+ph2+tn+tp+op+graz100w+ind100w+res100w+for100w+rec100w+road100w+treat100w,data=wangenv,upper.panel=panel.cor,pch=20,na.action = na.omit)

pairs(~no3+no2+nh3+ph2+tn+tp+op+graz60w+ind60w+res60w+for60w+rec60w+road60w+treat60w,data=wangenv,upper.panel=panel.cor,pch=20,na.action = na.omit)

pairs(~alk+toc+cod+sb+cond+temp+sali+do+ph1+ph2+graz+res+for.+rec+road+treat,data=wangenv,upper.panel=panel.cor,pch=20,na.action = na.omit)

pairs(~alk+toc+cod+sb+cond+temp+sali+do+ph1+ph2+graz1000+ind1000+res1000+for1000+rec1000+road1000+treat1000,data=wangenv,upper.panel=panel.cor,pch=20,na.action = na.omit)

pairs(~alk+toc+cod+sb+cond+temp+sali+do+ph1+ph2+graz500+ind500+res500+for500+rec500+road500+treat500,data=wangenv,upper.panel=panel.cor,pch=20,na.action = na.omit)

pairs(~alk+toc+cod+sb+cond+temp+sali+do+ph1+ph2+graz300+ind300+res300+for300+rec300+road300+treat300,data=wangenv,upper.panel=panel.cor,pch=20,na.action = na.omit)

pairs(~alk+toc+cod+sb+cond+temp+sali+do+ph1+ph2+graz100+ind100+res100+for100+rec100+road100+treat100,data=wangenv,upper.panel=panel.cor,pch=20,na.action = na.omit)

pairs(~alk+toc+cod+sb+cond+temp+sali+do+ph1+ph2+graz60+ind60+res60+for60+road60+treat60,data=wangenv,upper.panel=panel.cor,pch=20,na.action = na.omit)

pairs(~alk+toc+cod+sb+cond+temp+sali+do+ph1+ph2+graz500w+ind500w+res500w+for500w+rec500w+road500w+treat500w,data=wangenv,upper.panel=panel.cor,pch=20,na.action = na.omit)

pairs(~alk+toc+cod+sb+cond+temp+sali+do+ph1+ph2+graz400w+ind400w+res400w+for400w+rec400w+road400w+treat400w,data=wangenv,upper.panel=panel.cor,pch=20,na.action = na.omit)

pairs(~alk+toc+cod+sb+cond+temp+sali+do+ph1+ph2+graz300w+ind300w+res300w+for300w+rec300w+road300w+treat300w,data=wangenv,upper.panel=panel.cor,pch=20,na.action = na.omit)

pairs(~alk+toc+cod+sb+cond+temp+sali+do+ph1+ph2+graz200w+ind200w+res200w+for200w+rec200w+road200w+treat200w,data=wangenv,upper.panel=panel.cor,pch=20,na.action = na.omit)

pairs(~alk+toc+cod+sb+cond+temp+sali+do+ph1+ph2+graz100w+ind100w+res100w+for100w+rec100w+road100w+treat100w,data=wangenv,upper.panel=panel.cor,pch=20,na.action = na.omit)

pairs(~alk+toc+cod+sb+cond+temp+sali+do+ph1+ph2+graz60w+ind60w+res60w+for60w+rec60w+road60w+treat60w,data=wangenv,upper.panel=panel.cor,pch=20,na.action = na.omit)

pairs(~turb+vel+chla+cfpom+sed+graz+res+for.+rec+road+treat,data=wangenv,upper.panel=panel.cor,pch=20,na.action = na.omit)

pairs(~turb+vel+chla+cfpom+sed+graz1000+ind1000+res1000+for1000+rec1000+road1000+treat1000+tra1000+un1000,data=wangenv,upper.panel=panel.cor,pch=20,na.action = na.omit)

pairs(~turb+vel+chla+cfpom+sed+graz500+ind500+res500+for500+rec500+road500+treat500+tra500+un500,data=wangenv,upper.panel=panel.cor,pch=20,na.action = na.omit)

pairs(~turb+vel+chla+cfpom+sed+graz300+ind300+res300+for300+rec300+road300+treat300+tra300+un300,data=wangenv,upper.panel=panel.cor,pch=20,na.action = na.omit)

pairs(~turb+vel+chla+cfpom+sed+graz100+ind100+res100+for100+rec100+road100+treat100+un100,data=wangenv,upper.panel=panel.cor,pch=20,na.action = na.omit)

pairs(~turb+vel+chla+cfpom+sed+graz60+ind60+res60+for60+road60+treat60+un60,data=wangenv,upper.panel=panel.cor,pch=20,na.action = na.omit)

pairs(~turb+vel+chla+cfpom+sed+graz500w+ind500w+res500w+for500w+rec500w+road500w+treat500w+un500w,data=wangenv,upper.panel=panel.cor,pch=20,na.action = na.omit)

pairs(~turb+vel+chla+cfpom+sed+graz400w+ind400w+res400w+for400w+rec400w+road400w+treat400w+un400w,data=wangenv,upper.panel=panel.cor,pch=20,na.action = na.omit)

pairs(~turb+vel+chla+cfpom+sed+graz300w+ind300w+res300w+for300w+rec300w+road300w+treat300w+un300w,data=wangenv,upper.panel=panel.cor,pch=20,na.action = na.omit)

pairs(~turb+vel+chla+cfpom+sed+graz200w+ind200w+res200w+for200w+rec200w+road200w+treat200w+un200w,data=wangenv,upper.panel=panel.cor,pch=20,na.action = na.omit)

pairs(~turb+vel+chla+cfpom+sed+graz100w+ind100w+res100w+for100w+rec100w+road100w+treat100w+un100w,data=wangenv,upper.panel=panel.cor,pch=20,na.action = na.omit)

pairs(~turb+vel+chla+cfpom+sed+graz60w+ind60w+res60w+for60w+rec60w+road60w+treat60w+un60w,data=wangenv,upper.panel=panel.cor,pch=20,na.action = na.omit)

####Scatterplot Matrices of correlation between all landuse variables ####

###and environmental variables in revised causal diagram

par(mar=c(0.2,0.2,0.2,0.2),cex=0.6,cex.axis=0.8,las=1)

panel.cor <- function(x, y, digits = 2, cex.cor, ...)

{

usr <- par("usr"); on.exit(par(usr))

par(usr = c(0, 1, 0, 1))

# correlation coefficient

r <- cor(x, y)

txt <- format(c(r, 0.123456789), digits = digits)[1]

txt <- paste("r= ", txt, sep = "")

text(0.5, 0.8, txt)

# p-value calculation

p <- cor.test(x, y)$p.value

method=c("spearman")

txt2 <- format(c(p, 0.123456789), digits = digits)[1]

txt2 <- paste("p= ", txt2, sep = "")

if(p<0.01) txt2 <- paste("p= ", "<0.01", sep = "")

text(0.5, 0.4, txt2)

}

pairs(~cond+toc+chla+zn+temp+tp+no3+alk+turb+graz+res+for.+rec+road+treat,data=wangenv,upper.panel=panel.cor,pch=20,na.action = na.omit)

pairs(~log(cond)+log(toc)+log(chla)+log(zn)+log(temp)+log(tp)+log(no3)+log(alk)+log(turb)+graz+res+for.+rec+road+treat,data=wangenv,upper.panel=panel.cor,pch=20,na.action = na.omit)

pairs(~cond+toc+chla+zn+temp+tp+no3+alk+turb+graz1000+ind1000+res1000+for1000+rec1000+road1000+treat1000,data=wangenv,upper.panel=panel.cor,pch=20,na.action = na.omit)

pairs(~log(cond)+log(toc)+log(chla)+log(zn)+log(temp)+log(tp)+log(no3)+log(alk)+log(turb)+graz1000+ind1000+res1000+for1000+rec1000+road1000+treat1000,data=wangenv,upper.panel=panel.cor,pch=20,na.action = na.omit)

pairs(~cond+toc+chla+zn+temp+tp+no3+alk+turb+graz500+ind500+res500+for500+rec500+road500+treat500,data=wangenv,upper.panel=panel.cor,pch=20,na.action = na.omit)

pairs(~log(cond)+log(toc)+log(chla)+log(zn)+log(temp)+log(tp)+log(no3)+log(alk)+log(turb)+graz500+ind500+res500+for500+rec500+road500+treat500,data=wangenv,upper.panel=panel.cor,pch=20,na.action = na.omit)

pairs(~cond+toc+chla+zn+temp+tp+no3+alk+turb+graz300+ind300+res300+for300+rec300+road300+treat300,data=wangenv,upper.panel=panel.cor,pch=20,na.action = na.omit)

pairs(~log(cond)+log(toc)+log(chla)+log(zn)+log(temp)+log(tp)+log(no3)+log(alk)+log(turb)+graz300+ind300+res300+for300+rec300+road300+treat300,data=wangenv,upper.panel=panel.cor,pch=20,na.action = na.omit)

pairs(~cond+toc+chla+zn+temp+tp+no3+alk+turb+graz100+ind100+res100+for100+rec100+road100+treat100,data=wangenv,upper.panel=panel.cor,pch=20,na.action = na.omit)

pairs(~log(cond)+log(toc)+log(chla)+log(zn)+log(temp)+log(tp)+log(no3)+log(alk)+log(turb)+graz100+ind100+res100+for100+rec100+road100+treat100,data=wangenv,upper.panel=panel.cor,pch=20,na.action = na.omit)

pairs(~cond+toc+chla+zn+temp+tp+no3+alk+turb+graz60+ind60+res60+for60+road60+treat60,data=wangenv,upper.panel=panel.cor,pch=20,na.action = na.omit)

pairs(~log(cond)+log(toc)+log(chla)+log(zn)+log(temp)+log(tp)+log(no3)+log(alk)+log(turb)+graz60+ind60+res60+for60+road60+treat60,data=wangenv,upper.panel=panel.cor,pch=20,na.action = na.omit)

pairs(~cond+toc+chla+zn+temp+tp+no3+alk+turb+graz500w+ind500w+res500w+for500w+rec500w+road500w+treat500w,data=wangenv,upper.panel=panel.cor,pch=20,na.action = na.omit)

pairs(~log(cond)+log(toc)+log(chla)+log(zn)+log(temp)+log(tp)+log(no3)+log(alk)+log(turb)+graz500w+ind500w+res500w+for500w+rec500w+road500w+treat500w,data=wangenv,upper.panel=panel.cor,pch=20,na.action = na.omit)

pairs(~cond+toc+chla+zn+temp+tp+no3+alk+turb+graz400w+ind400w+res400w+for400w+rec400w+road400w+treat400w,data=wangenv,upper.panel=panel.cor,pch=20,na.action = na.omit)

pairs(~log(cond)+log(toc)+log(chla)+log(zn)+log(temp)+log(tp)+log(no3)+log(alk)+log(turb)+graz400w+ind400w+res400w+for400w+rec400w+road400w+treat400w,data=wangenv,upper.panel=panel.cor,pch=20,na.action = na.omit)

pairs(~cond+toc+chla+zn+temp+tp+no3+alk+turb+graz300w+ind300w+res300w+for300w+rec300w+road300w+treat300w,data=wangenv,upper.panel=panel.cor,pch=20,na.action = na.omit)

pairs(~log(cond)+log(toc)+log(chla)+log(zn)+log(temp)+log(tp)+log(no3)+log(alk)+log(turb)+graz300w+ind300w+res300w+for300w+rec300w+road300w+treat300w,data=wangenv,upper.panel=panel.cor,pch=20,na.action = na.omit)

pairs(~cond+toc+chla+zn+temp+tp+no3+alk+turb+graz200w+ind200w+res200w+for200w+rec200w+road200w+treat200w,data=wangenv,upper.panel=panel.cor,pch=20,na.action = na.omit)

pairs(~log(cond)+log(toc)+log(chla)+log(zn)+log(temp)+log(tp)+log(no3)+log(alk)+log(turb)+graz200w+ind200w+res200w+for200w+rec200w+road200w+treat200w,data=wangenv,upper.panel=panel.cor,pch=20,na.action = na.omit)

pairs(~cond+toc+chla+zn+temp+tp+no3+alk+turb+graz100w+ind100w+res100w+for100w+rec100w+road100w+treat100w,data=wangenv,upper.panel=panel.cor,pch=20,na.action = na.omit)

pairs(~log(cond)+log(toc)+log(chla)+log(zn)+log(temp)+log(tp)+log(no3)+log(alk)+log(turb)+graz100w+ind100w+res100w+for100w+rec100w+road100w+treat100w,data=wangenv,upper.panel=panel.cor,pch=20,na.action = na.omit)

pairs(~cond+toc+chla+zn+temp+tp+no3+alk+turb+graz60w+ind60w+res60w+for60w+rec60w+road60w+treat60w,data=wangenv,upper.panel=panel.cor,pch=20,na.action = na.omit)

pairs(~log(cond)+log(toc)+log(chla)+log(zn)+log(temp)+log(tp)+log(no3)+log(alk)+log(turb)+graz60w+ind60w+res60w+for60w+rec60w+road60w+treat60w,data=wangenv,upper.panel=panel.cor,pch=20,na.action = na.omit)

dev.off()

###correlation between landuse and some environmental variables

plot(wangenv$cd,wangenv$graz60)

cor.test(wangenv$cd,wangenv$graz60)

plot(wangenv$cd,log1p(wangenv$graz60))

cor.test(wangenv$cd,log1p(wangenv$graz60))

plot(wangenv$cd,wangenv$ind60)

cor.test(wangenv$cd,wangenv$ind60)

plot(wangenv$cd,log1p(wangenv$ind60))

cor.test(wangenv$cd,log1p(wangenv$ind60))

plot(wangenv$cd,wangenv$res60)

cor.test(wangenv$cd,wangenv$res60)

plot(wangenv$cd,log1p(wangenv$res60))

cor.test(wangenv$cd,log1p(wangenv$res60))

plot(wangenv$cd,wangenv$for60)

cor.test(wangenv$cd,wangenv$for60)

plot(wangenv$cd,log1p(wangenv$for60))

cor.test(wangenv$cd,log1p(wangenv$for60))

plot(wangenv$cd,wangenv$rec60)

cor.test(wangenv$cd,wangenv$rec60)

plot(wangenv$cd,log1p(wangenv$rec60))

cor.test(wangenv$cd,log1p(wangenv$rec60))

plot(wangenv$cd,wangenv$road60)

cor.test(wangenv$cd,wangenv$road60)

plot(wangenv$cd,log1p(wangenv$road60))

cor.test(wangenv$cd,log1p(wangenv$road60))

plot(wangenv$cd,wangenv$treat60)

cor.test(wangenv$cd,wangenv$treat60)

plot(wangenv$cd,log1p(wangenv$road60))

cor.test(wangenv$cd,log1p(wangenv$road60))

plot(wangenv$cd,wangenv$qua500)

cor.test(wangenv$cd,wangenv$qua500)

plot(wangenv$cd,wangenv$tra60)

cor.test(wangenv$cd,wangenv$tra60)

plot(wangenv$cd,wangenv$un60)

cor.test(wangenv$cd,wangenv$un60)

plot(wangenv$cd,log1p(wangenv$un60))

cor.test(wangenv$cd,log1p(wangenv$un60))

plot(wangenv$turb,wangenv$res300)

cor.test(wangenv$turb,wangenv$res300)

plot(log(wangenv$turb),log(wangenv$res300))

cor.test(log(wangenv$turb),log(wangenv$res300))

##

#####Testing other variables for causal diagram

edit(alldata)

#

# Chlorophyl A

#check the scatterplot for chla

edit(alldata)

par(mar=c(5,5,4,2),cex=0.9)

panel.cor <- function(x, y, digits = 2, cex.cor, ...)

{

usr <- par("usr"); on.exit(par(usr))

par(usr = c(0, 1, 0, 1))

# correlation coefficient

r <- cor(x, y)

method=c("spearman")

txt <- format(c(r, 0.123456789), digits = digits)[1]

txt <- paste("r= ", txt, sep = "")

text(0.5, 0.8, txt)

# p-value calculation

p <- cor.test(x, y)$p.value

txt2 <- format(c(p, 0.123456789), digits = digits)[1]

txt2 <- paste("p= ", txt2, sep = "")

if(p<0.01) txt2 <- paste("p= ", "<0.01", sep = "")

text(0.5, 0.3, txt2)

}

pairs(dchla~eff+solar+temp+no3+tp+turb+don+dtoc+canop+toc+log(chla)+chla,data=alldata,upper.panel=panel.cor,pch=20,na.action = na.omit)

pairs(log(I(dchla/dayflow))~eff+solar+temp+no3+tp+turb+don+dtoc+canop+toc+log(chla)+chla,data=alldata[alldata$eff==1,],pch=20,na.action = na.omit)

pairs(~eff+solar+temp+no3+tn+tp+log(turb)+don+dtoc+canop+toc+vel+I(log(dchla/dayflow))+dayflow+log(chla)+residuals(tempo),data=alldata[complete.cases(alldata[,c("temp","no3","tp")]),],upper.panel=panel.cor,pch=20,na.action = na.omit)

#chla regression with other variables

tempo<-lm(log(chla)~temp,data=alldata)##Adjusted R-squared: 0.5416

tempo<-lm(log(chla)~on,data=alldata)

tempo<-lm(log(chla)~eff,data=alldata)##Adjusted R-squared: 0.1782

tempo<-lm(log(chla)~on+on:temp,data=alldata) ##Adjusted R-squared: 0.6483

tempo<-lm(log(chla)~no3,data=alldata)##Adjusted R-squared: 0.09357

tempo<-lm(log(chla)~no3+temp:no3,data=alldata)##Adjusted R-squared: 0.6231

tempo<-lm(log(chla)~vel,data=alldata)

tempo<-lm(log(chla)~vel+vel:temp,data=alldata)##Adjusted R-squared: 0.3877

tempo<-lm(log(chla)~nh3,data=alldata)

tempo<-lm(log(chla)~nh3+temp:nh3,data=alldata)##Adjusted R-squared: 0.1902

tempo<-lm(log(chla)~no2,data=alldata)

tempo<-lm(log(chla)~no2+no2:temp,data=alldata)##Adjusted R-squared: 0.5032

tempo<-lm(log(chla)~tp,data=alldata)##Adjusted R-squared: 0.3512

tempo<-lm(log(chla)~tp+tp:temp,data=alldata)##Adjusted R-squared: 0.6564

tempo<-lm(log(chla)~op,data=alldata)

tempo<-lm(log(chla)~op+op:temp,data=alldata)

tempo<-lm(log(chla)~solar,data=alldata)##Adjusted R-squared: 0.4824

tempo<-lm(log(chla)~solar+solar:temp,data=alldata)

tempo<-lm(log(chla)~turb,data=alldata)##Adjusted R-squared: 0.1113

tempo<-lm(log(chla)~turb+turb:temp,data=alldata)##Adjusted R-squared: 0.5947

tempo<-lm(log(chla)~dchla,data=alldata)##Adjusted R-squared: 0.2251

tempo<-lm(log(chla)~canop,data=alldata)

tempo<-lm(log(chla)~canop+canop:temp,data=alldata)##Adjusted R-squared: 0.6161

tempo<-lm(log(chla)~dayflow,data=alldata)

tempo<-lm(log(chla)~don,data=alldata)

tempo<-lm(log(chla)~dtoc,data=alldata)##Adjusted R-squared: 0.1068

tempo<-lm(log(chla)~cfpom,data=alldata)

tempo<-lm(log(chla)~toc,data=alldata)

tempo<-lm(log(chla)~dtoc+dtoc:temp,data=alldata)

tempo<-lm(log(chla)~don,data=alldata)

tempo<-lm(log(chla)~don+don:temp,data=alldata)

tempo<-lm(log(chla)~tn,data=alldata)

tempo<-lm(log(chla)~tn+dtn:temp,data=alldata)

tempo<-lm(log(chla)~cond,data=alldata)

tempo<-lm(log(chla)~cond+cond:temp,data=alldata)

summary(tempo)

plot(alldata$chla~alldata$temp)

cor.test(alldata$chla,alldata$temp)

plot(log(alldata$chla),alldata$cod)

cor.test(log(alldata$chla),alldata$cod)

#Chlorophyll A plotted against spatial position

plot(bugenv[bugenv$day==1,"dist"],bugenv[bugenv$day==1,"chla"],type = "b",ylim=c(0,40),pch=19,xlab="Distance(km)",ylab="Chlorophyll A (mg/L)",main="Chlorophyll A plotted against spatial position",cex=1,lwd=3,lty=1)

points(bugenv[bugenv$day==126,"dist"],bugenv[bugenv$day==126,"chla"],type="b",col="gold2",pch=15,lwd=3,cex=1.2,lty=2)

points(bugenv[bugenv$day==260,"dist"],bugenv[bugenv$day==260,"chla"],type="b",col="blue",pch=8,cex=1.2,lwd=3,lty=3)

points(bugenv[bugenv$day==336,"dist"],bugenv[bugenv$day==336,"chla"],type="b",col="green3",pch=17,cex=1.2,lwd=3,lty=4)

points(bugenv[bugenv$day==518,"dist"],bugenv[bugenv$day==518,"chla"],type="b",col="red",pch=18,cex=1.4,lwd=3,lty=5)

legend("topleft",inset=c(0,0),legend=c("Dec 13", "April 14", "Aug 2014", "Nov 2014","May 2015"),lty=c(1,5),pch=c(19,15,8,17,18),lwd=2,col=c("black","gold2","blue","green3","red"),ncol=2,horiz=FALSE,cex=0.6,title="months")

abline(v=4,lty=2)

# Multiple regression analysis for chla as afunction of time, distance and eff to check spatiotemporal variation of Chla

# Chlorophyll A with spatiotemporal prediction

tempo<-lm(log(chla)~dist+time+eff+eff:time+eff:dist+eff:time:dist+eff:time:I(dist^2),data=alldata[complete.cases(alldata[,c("temp","no3","tp")]),])# final model# ##*********##

anova(tempo) ##***** Table 4 *****##

summary(tempo) ##***** Table 5 *****##

tempo1<-cbind(cbind(exp(fitted(tempo)),residuals(tempo)),alldata[complete.cases(alldata[,c("temp","no3","tp")]),])#

plot(tempo1[tempo1$day==1,"dist"],tempo1[tempo1$day==1,"chla"],type="p",pch=19,xlab="Distance (km)",ylab="Chlorophyll A (mg/L)",main="Chlorophyll A with spatiotemporal prediction",ylim=c(0,40),cex=1,lwd=3,lty=1)

points(tempo1[tempo1$day==126,"dist"],tempo1[tempo1$day==126,"chla"],col="gold2",pch=15,cex=1.2,lwd=3,lty=2)

points(tempo1[tempo1$day==260,"dist"],tempo1[tempo1$day==260,"chla"],col="blue",pch=8,cex=1.2,lwd=3,lty=3)

points(tempo1[tempo1$day==336,"dist"],tempo1[tempo1$day==336,"chla"],col="green3",pch=17,cex=1.2,lwd=3,lty=4)

points(tempo1[tempo1$day==518,"dist"],tempo1[tempo1$day==518,"chla"],col="red",pch=18,cex=1.4,lwd=3,lty=5)

points(tempo1[tempo1$day==1,"dist"],tempo1[tempo1$day==1,"1"],type="l",col="black",pch=19,cex=1.5,lwd=3,lty=1)

points(tempo1[tempo1$day==126,"dist"],tempo1[tempo1$day==126,"1"],type="l",col="gold2",pch=15,cex=1.5,lwd=3,lty=2)

points(tempo1[tempo1$day==260,"dist"],tempo1[tempo1$day==260,"1"],type="l",col="blue",pch=8,cex=1.5,lwd=3,lty=3)

points(tempo1[tempo1$day==336,"dist"],tempo1[tempo1$day==336,"1"],type="l",col="green3",pch=17,cex=1.5,lwd=3,lty=4)

points(tempo1[tempo1$day==518,"dist"],tempo1[tempo1$day==518,"1"],type="l",col="red",pch=18,cex=2,lwd=3,lty=5)

legend("topleft",inset=c(0,0),legend=c("Dec 13", "April 14", "Aug 2014", "Nov 2014","May 2015"),lty=c(1,5),pch=c(19,15,8,17,18),lwd=2,col=c("black","gold2","blue","green3","red"),ncol=2,horiz=FALSE,cex=0.6,title="months")

abline(v=4,lty=2)

xyplot(log(chla)~dist,groups=time,data=alldata,type="b",lwd=2,col=c("black","gold2","blue","green3","red"))

xyplot(dayflow~dist,groups=time,data=alldata,type="b",lwd=2,col=c("black","gold2","blue","green3","red"))

xyplot(turb~dist,groups=time,data=alldata,type="b",lwd=2,col=c("black","gold2","blue","green3","red"))

a<-xyplot(chla~dist,group=time,data=alldata[complete.cases(alldata[,c("temp","no3","tp")]),],type="b",pch=19,lty=2,col=c("black","gold2","blue","green3","red"))

b<-xyplot(exp(fitted(tempo))~dist,group=time,data=alldata[complete.cases(alldata[,c("temp","no3","tp")]),],type="l",lwd=2,col=c("black","gold2","blue","green3","red"))

c<-xyplot(toc~dist,data=alldata[complete.cases(alldata[,c("temp","no3","tp")]),],panel = function(x,y) {panel.abline(v=4,lty=2)})

a+as.layer(b)+as.layer(c)

legend("topleft",inset=c(0,0),legend=c("Dec 13", "April 14", "Aug 2014", "Nov 2014","May 2015"),lty=c(1,5),pch=c(19,15,8,17,18),lwd=2,col=c("black","gold2","blue","green3","red"),ncol=2,horiz=FALSE,cex=0.6,title="months")

###Chlorophyll A linear model with other environmental variables

#tempo<-lm(log(chla)~solar+temp+tp+temp:tp+dchla+no3+dtoc+turb,data=wangenv)##

#tempo<-lm(log(chla)~solar+temp2+tp+no3+dayflow+eff+eff:dist+eff:I(dist^2)+eff:dchla+eff:dchla:dist+eff:dchla:I(dist^2)+eff:dayflow+eff:dayflow:dist+eff:dayflow:I(dist^2)+eff:temp2+eff:temp2:dist+eff:temp2:I(dist^2),data=alldata)## final model

#tempo<-lm(log(chla)~solar+temp2+tp+no3+dayflow+eff+eff:dchla+eff:temp2+eff:temp2:dist,data=alldata)

#tempo<-lm(log(chla)~solar+temp2+tp+no3+dayflow+eff:dchla,data=alldata)## without non-significant ones

#tempo<-lm(log(chla)~solar+temp2+tp+no3+turb+dayflow,data=alldata)

#tempo<-lm(log(chla)~solar+temp+tp+dchla+no3+dtoc+turb+dist+temp:tp+dchla:eff,data=alldata)

#tempo<-lm(log(chla)~solar+temp2+tp+no3+dtoc+turb+dayflow+eff:dist+eff:I(dchla/dayflow)+eff:I(dchla/dayflow):dist+eff:dist:temp2 #RH

#tempo<-lm(log(chla)~solar+temp+tp+no3+turb+dayflow+solar:temp+eff:log(I(dchla/dayflow)),data=alldata[complete.cases(alldata[,c("temp","no3","tp")]),])#

tempo<-lm(log(chla)~solar+temp+tp+no3+turb+solar:temp+eff:log(I(dchla/dayflow)),data=alldata[complete.cases(alldata[,c("temp","no3","tp")]),])# final model

tempo1<-cbind(cbind(exp(fitted(tempo)),exp(residuals(tempo))),alldata[complete.cases(alldata[,c("temp","no3","tp")]),])

attributes(tempo1)

names(tempo1[,1:2])<-c("fitted","residual")

tempo1[,"2"]

anova(tempo) ##***** Table 6 - appendix*****##

summary(tempo) ##***** Table 7 - appendix*****##

plot(tempo)

avPlots(tempo)

plot(tempo1[tempo1$day==126,"dist"],tempo1[tempo1$day==126,"1"],type = "l",ylim=c(0,40),pch=15,col="gold2",xlab="Distance(km)",ylab="Chlorophyll A (mg/L)",main="Prediction with environmental variables",lwd=3,lty=2)

#points(tempo1[tempo1$day==126,"dist"],tempo1[tempo1$day==126,"1"],type="l",col="gold2",pch=15,lwd=3,cex=1.5,lty=2)

points(tempo1[tempo1$day==260,"dist"],tempo1[tempo1$day==260,"1"],type="l",col="blue",pch=8,cex=1.2,lwd=3,lty=3)

points(tempo1[tempo1$day==336,"dist"],tempo1[tempo1$day==336,"1"],type="l",col="green3",pch=17,cex=1.2,lwd=3,lty=4)

points(tempo1[tempo1$day==518,"dist"],tempo1[tempo1$day==518,"1"],type="l",col="red",pch=18,cex=1.4,lwd=3,lty=5)

legend("topleft",inset=c(0,0),legend=c("Dec 13", "April 14", "Aug 2014", "Nov 2014","May 2015"),lty=c(1,5),pch=c(19,15,8,17,18),lwd=2,col=c("black","gold2","blue","green3","red"),ncol=2,horiz=FALSE,cex=0.6,title="months")

abline(v=4,lty=2)

tempo3<-lm(tempo1[,2]~tempo1$pred1)

summary(tempo3)

summary(tempo)

anova(tempo)

sqrt(vif(tempo)) #values > 2 indicate a problem, which is due to the correlation between temperature and solar, but literature suggests both are important, together with their interaction

plot(tempo)

# The p-value=0.1492 for this model, i.e., there is no evidence of spatiotemporal patterns in the residuals.

tempo.check<-lm(residuals(tempo)~dist+time+eff+eff:time+eff:dist+eff:time:dist+eff:time:I(dist^2),data=alldata[complete.cases(alldata[,c("temp","no3","tp")]),])

summary(tempo.check)

anova(tempo.check)

xyplot(residuals(tempo)~dist,groups=time,data=alldata[complete.cases(alldata[,c("temp","no3","tp")]),],type="b",pch=19,lwd=2,col=c("black","gold2","blue","green3","red"))

a<-xyplot(chla~dist,group=time,data=alldata[complete.cases(alldata[,c("temp","no3","tp")]),],type="b",pch=19,lty=2,col=c("black","gold2","blue","green3","red"))

b<-xyplot(exp(fitted(tempo))~dist,group=time,data=alldata[complete.cases(alldata[,c("temp","no3","tp")]),],type="l",lwd=2,col=c("black","gold2","blue","green3","red"))

c<-xyplot(toc~dist,data=alldata[complete.cases(alldata[,c("temp","no3","tp")]),],panel = function(x,y) {panel.abline(v=4,lty=2)})

a+as.layer(b)+as.layer(c)

legend("topleft",inset=c(0,0),legend=c("Dec 13", "April 14", "Aug 2014", "Nov 2014","May 2015"),lty=c(1,5),pch=c(19,15,8,17,18),lwd=2,col=c("black","gold2","blue","green3","red"),ncol=2,horiz=FALSE,cex=0.6,title="months")

plot.new()

dev.off()

##***** Chlorophyll A plot (Figure 69) *****#

tiff(file="Figure 6.jpg",width=10,height=14,units="in",pointsize = 12,bg ="transparent",res=800,compression="lzw")

par(mfrow=c(3,1), mar=c(4.5,4.5,2.5,3),cex=1.5,cex.axis=0.9,las=1,cex.main=1,cex.lab=0.8)

#Chlorophyll A plotted against spatial position

plot(bugenv[bugenv$day==1,"dist"],bugenv[bugenv$day==1,"chla"],type = "b",ylim=c(0,40),pch=19,xlab="",ylab="Chlorophyll A (mg/L)",main="a) Chlorophyll A plotted against spatial position",lwd=3,lty=1)

points(bugenv[bugenv$day==126,"dist"],bugenv[bugenv$day==126,"chla"],type="b",col="gold2",pch=15,lwd=3,cex=1.2,lty=2)

points(bugenv[bugenv$day==260,"dist"],bugenv[bugenv$day==260,"chla"],type="b",col="blue",pch=8,cex=1.2,lwd=3,lty=3)

points(bugenv[bugenv$day==336,"dist"],bugenv[bugenv$day==336,"chla"],type="b",col="green3",pch=17,cex=1.2,lwd=3,lty=4)

points(bugenv[bugenv$day==518,"dist"],bugenv[bugenv$day==518,"chla"],type="b",col="red",pch=18,cex=1.4,lwd=3,lty=5)

legend("topleft",inset=c(0,0),legend=c("Dec 13", "April 14", "Aug 2014", "Nov 2014","May 2015"),lty=c(1,5),pch=c(19,15,8,17,18),lwd=2,col=c("black","gold2","blue","green3","red"),ncol=2,horiz=FALSE,cex=0.6,title="months")

abline(v=4,lty=2)

# Chlorophyll A with spatiotemporal prediction

tempo<-lm(log(chla)~dist+time+eff+eff:time+eff:dist+eff:time:dist+eff:time:I(dist^2),data=alldata)

tempo1<-cbind(cbind(exp(fitted(tempo)),residuals(tempo)),alldata)#[complete.cases(alldata[,c("temp","cond")]),])

plot(tempo1$dist[tempo1$time==1],tempo1[tempo1$time==1,"chla"],type="p",pch=19,col="black",xlab="",ylab="Chlorophyll A (mg/L)",main="b) Chlorophyll A with spatiotemporal prediction",ylim=c(0,40),cex=1,lwd=3,lty=1)

points(tempo1$dist[tempo1$time==1],tempo1[tempo1$time==2,"chla"],col="gold2",pch=15,cex=1.2,lwd=3,lty=2)

points(tempo1$dist[tempo1$time==1],tempo1[tempo1$time==3,"chla"],col="blue",pch=8,cex=1.2,lwd=3,lty=3)

points(tempo1$dist[tempo1$time==1],tempo1[tempo1$time==4,"chla"],col="green3",pch=17,cex=1.2,lwd=3,lty=4)

points(tempo1$dist[tempo1$time==1],tempo1[tempo1$time==5,"chla"],col="red",pch=18,cex=1.4,lwd=3,lty=5)

points(tempo1[tempo1$day==1,"dist"],tempo1[tempo1$day==1,"1"],type="l",col="black",pch=19,cex=1.5,lwd=3,lty=1)

points(tempo1[tempo1$day==126,"dist"],tempo1[tempo1$day==126,"1"],type="l",col="gold2",pch=15,cex=1.5,lwd=3,lty=2)

points(tempo1[tempo1$day==260,"dist"],tempo1[tempo1$day==260,"1"],type="l",col="blue",pch=8,cex=1.5,lwd=3,lty=3)

points(tempo1[tempo1$day==336,"dist"],tempo1[tempo1$day==336,"1"],type="l",col="green3",pch=17,cex=1.5,lwd=3,lty=4)

points(tempo1[tempo1$day==518,"dist"],tempo1[tempo1$day==518,"1"],type="l",col="red",pch=18,cex=2,lwd=3,lty=5)

legend("topleft",inset=c(0,0),legend=c("Dec 13", "April 14", "Aug 2014", "Nov 2014","May 2015"),lty=c(1,5),pch=c(19,15,8,17,18),lwd=2,col=c("black","gold2","blue","green3","red"),ncol=2,horiz=FALSE,cex=0.6,title="months")

abline(v=4,lty=2)

#prediction with environmental variables

tempo<-lm(log(chla)~solar+temp+tp+no3+turb+solar:temp+eff:log(I(dchla/dayflow)),data=alldata[complete.cases(alldata[,c("temp","no3","tp")]),])# final model

summary(tempo)

anova(tempo)

tempo1<-cbind(cbind(exp(fitted(tempo)),exp(residuals(tempo))),alldata[complete.cases(alldata[,c("temp","no3","tp")]),])

names(tempo1[,1:2])<-c("fitted","residual")

tempo1[,"2"]

plot(tempo1[tempo1$day==126,"dist"],tempo1[tempo1$day==126,"1"],type = "b",ylim=c(0,40),pch=15,col="gold2",xlab="Distance(km)",ylab="Chlorophyll A (mg/L)",main="c) Prediction with environmental variables",lwd=3,lty=2)

#points(tempo1[tempo1$day==126,"dist"],tempo1[tempo1$day==126,"1"],type="b",col="gold2",pch=15,lwd=3,cex=1.5,lty=2)

points(tempo1[tempo1$day==260,"dist"],tempo1[tempo1$day==260,"1"],type="b",col="blue",pch=8,cex=1.2,lwd=3,lty=3)

points(tempo1[tempo1$day==336,"dist"],tempo1[tempo1$day==336,"1"],type="b",col="green3",pch=17,cex=1.2,lwd=3,lty=4)

points(tempo1[tempo1$day==518,"dist"],tempo1[tempo1$day==518,"1"],type="b",col="red",pch=18,cex=1.4,lwd=3,lty=5)

legend("topleft",inset=c(0,0),legend=c("Dec 13", "April 14", "Aug 2014", "Nov 2014","May 2015"),lty=c(1,5),pch=c(19,15,8,17,18),lwd=2,col=c("black","gold2","blue","green3","red"),ncol=2,horiz=FALSE,cex=0.6,title="months")

abline(v=4,lty=2)

dev.off()

###chla non-linear model

##chla simulation

temp3<-alldata$temp2-20

edit(alldata)

chlanlm<-0.8+1.5*(alldata$time==4)+2.8*alldata$eff*(alldata$dchla/alldata$dayflow)*exp(-0.03*0.9^temp3*alldata$dist/alldata$vel2)

chlanlm<-0.8+1.5*(alldata$time==2)+0.5*(alldata$time==3)+1.5*(alldata$time==4)+2.8*alldata$eff*(alldata$dchla/alldata$dayflow)*exp(-0.03*1.1^temp3*alldata$dist/alldata$vel2)

alldata2<-cbind(alldata,chlanlm,temp3)

par(mar=c(5,5,4,2),cex=0.9)

plot(alldata2$dist[alldata2$time==1],alldata2[alldata2$time==1,"chla"],type="p",pch=19,col="black",xlab="Distance (km)",ylab="Chla (mg/L)",main="Chla with spatiotemporal prediction",ylim=c(0,40),cex=1,lwd=3,lty=1)

points(alldata2$dist[alldata2$time==1],alldata2[alldata2$time==2,"chla"],col="gold2",pch=15,cex=1.2,lwd=3,lty=2)

points(alldata2$dist[alldata2$time==1],alldata2[alldata2$time==3,"chla"],col="blue",pch=8,cex=1.2,lwd=3,lty=3)

points(alldata2$dist[alldata2$time==1],alldata2[alldata2$time==4,"chla"],col="green3",pch=17,cex=1.2,lwd=3,lty=4)

points(alldata2$dist[alldata2$time==1],alldata2[alldata2$time==5,"chla"],col="red",pch=18,cex=1.4,lwd=3,lty=5)

points(alldata2[alldata2$day==1,"dist"],alldata2[alldata2$day==1,"chlanlm"],type="l",col="black",pch=15,lwd=3,cex=1.5,lty=1)

points(alldata2[alldata2$day==126,"dist"],alldata2[alldata2$day==126,"chlanlm"],type="l",col="gold2",pch=15,lwd=3,cex=1.5,lty=2)

points(alldata2[alldata2$day==260,"dist"],alldata2[alldata2$day==260,"chlanlm"],type="l",col="blue",pch=8,cex=1.5,lwd=3,lty=3)

points(alldata2[alldata2$day==336,"dist"],alldata2[alldata2$day==336,"chlanlm"],type="l",col="green3",pch=17,cex=1.5,lwd=3,lty=4)

points(alldata2[alldata2$day==518,"dist"],alldata2[alldata2$day==518,"chlanlm"],type="l",col="red",pch=18,cex=2,lwd=3,lty=5)

legend("topright",inset=c(0,0),legend=c("Dec 13", "April 14", "Aug 2014", "Nov 2014","May 2015"),lty=c(1,5),pch=c(19,15,8,17,18),lwd=2,col=c("black","gold2","blue","green3","red"),ncol=2,horiz=FALSE,cex=0.9,title="months")

abline(v=4,lty=2)

myalldata<- na.omit(alldata)

lmfit<-train(log(chla)~solar+temp+tp+no3+turb+solar:temp+eff:log(I(dchla/dayflow)),data=alldata[complete.cases(alldata[,c("temp","no3","tp")]),])# Checked WP+RH

print(lmfit)

##fitting the model

#Linear model works so no need for non linear model

#

# Nitrate (NO3)

# check the scatterplot for no3

par(mar=c(5,5,4,2),cex=0.9)

panel.cor <- function(x, y, digits = 2, cex.cor, ...)

{

usr <- par("usr"); on.exit(par(usr))

par(usr = c(0, 1, 0, 1))

# correlation coefficient

r <- cor(x, y)

method=c("spearman")

txt <- format(c(r, 0.123456789), digits = digits)[1]

txt <- paste("r= ", txt, sep = "")

text(0.5, 0.8, txt)

# p-value calculation

p <- cor.test(x, y)$p.value

txt2 <- format(c(p, 0.123456789), digits = digits)[1]

txt2 <- paste("p= ", txt2, sep = "")

if(p<0.01) txt2 <- paste("p= ", "<0.01", sep = "")

text(0.5, 0.3, txt2)

}

pairs(~ph2+log(alk)+log(I(dno3/dayflow))+eff+dayflow+dist+log(temp)+log(toc)+log(no3)+no3,data=alldata,upper.panel=panel.cor,pch=20,na.action = na.omit)

edit(alldata)

plot(alldata$no3,alldata$dno3)

cor.test(alldata$no3,alldata$dno3)

#Nitrate regression with other variables

tempo<-lm(no3~eff,data=alldata)##Adjusted R-squared: 0.5636

tempo<-lm(no3~dno3,data=alldata)##Adjusted R-squared: 0.2578

tempo<-lm(no3~dayflow,data=alldata)##Adjusted R-squared: 0.279

tempo<-lm(no3~dist,data=alldata)##Adjusted R-squared: 0.09122

tempo<-lm(no3~temp,data=alldata)

#Nitrate plotted against spatial position

plot(bugenv[bugenv$day==1,"dist"],bugenv[bugenv$day==1,"no3"],type = "b",ylim=c(0,0.7),pch=19,xlab="Distance(km)",ylab="Nitrate (mg/L)",main="Nitrate plotted against spatial position",lwd=3,lty=1)

points(bugenv[bugenv$day==126,"dist"],bugenv[bugenv$day==126,"no3"],type="b",col="gold2",pch=15,lwd=3,cex=1.2,lty=2)

points(bugenv[bugenv$day==260,"dist"],bugenv[bugenv$day==260,"no3"],type="b",col="blue",pch=8,cex=1.2,lwd=3,lty=3)

points(bugenv[bugenv$day==336,"dist"],bugenv[bugenv$day==336,"no3"],type="b",col="green3",pch=17,cex=1.2,lwd=3,lty=4)

points(bugenv[bugenv$day==518,"dist"],bugenv[bugenv$day==518,"no3"],type="b",col="red",pch=18,cex=1.4,lwd=3,lty=5)

legend("topleft",inset=c(0,0),legend=c("Dec 13", "April 14", "Aug 2014", "Nov 2014","May 2015"),lty=c(1,5),pch=c(19,15,8,17,18),lwd=2,col=c("black","gold2","blue","green3","red"),ncol=2,horiz=FALSE,cex=0.6,title="months")

abline(v=4,lty=2)

# Multiple regression analysis for nitrate vs time, distance and eff to check spatiotemporal variation of nitrate

# observed nitrate plots with spatiotemporal model prediction

tempo<-lm(log(no3)~time+eff:time+eff:dist+eff:dist:time+eff:I(dist^2):time,data=alldata) # final model ##*********##

anova(tempo) ##*********##

summary(tempo) ##*********##

#tempo1<-cbind(cbind((fitted(tempo)),residuals(tempo)),alldata)#[complete.cases(alldata[,c("no3","dayflow","dno3")]),])

tempo1<-cbind(cbind(exp(fitted(tempo)),exp(residuals(tempo))),alldata)#[complete.cases(alldata[,c("temp","no3","tp")]),])

attributes(tempo1)

names(tempo1[,1:2])<-c("fitted","residual")

tempo1[,"2"]

#observed no3 plots with spatiotemporal model predictions

plot(tempo1$dist[tempo1$time==1],tempo1[tempo1$time==1,"no3"],type="p",pch=19,col="black",xlab="Distance (km)",ylab="Nitrate (mg/L)",main="Nitrate with spatiotemporal prediction",ylim=c(0,0.7),cex=1,lwd=3,lty=1)

points(tempo1$dist[tempo1$time==1],tempo1[tempo1$time==2,"no3"],col="gold2",pch=15,cex=1.2,lwd=3,lty=2)

points(tempo1$dist[tempo1$time==1],tempo1[tempo1$time==3,"no3"],col="blue",pch=8,cex=1.2,lwd=3,lty=3)

points(tempo1$dist[tempo1$time==1],tempo1[tempo1$time==4,"no3"],col="green3",pch=17,cex=1.2,lwd=3,lty=4)

points(tempo1$dist[tempo1$time==1],tempo1[tempo1$time==5,"no3"],col="red",pch=18,cex=1.4,lwd=3,lty=5)

points(tempo1[tempo1$day==1,"dist"],tempo1[tempo1$day==1,"1"],type="l",col="black",pch=19,cex=1.5,lwd=3,lty=1)

points(tempo1[tempo1$day==126,"dist"],tempo1[tempo1$day==126,"1"],type="l",col="gold2",pch=15,cex=1.5,lwd=3,lty=2)

points(tempo1[tempo1$day==260,"dist"],tempo1[tempo1$day==260,"1"],type="l",col="blue",pch=8,cex=1.5,lwd=3,lty=3)

points(tempo1[tempo1$day==336,"dist"],tempo1[tempo1$day==336,"1"],type="l",col="green3",pch=17,cex=1.5,lwd=3,lty=4)

points(tempo1[tempo1$day==518,"dist"],tempo1[tempo1$day==518,"1"],type="l",col="red",pch=18,cex=2,lwd=3,lty=5)

legend("topleft",inset=c(0,0),legend=c("Dec 13", "April 14", "Aug 2014", "Nov 2014","May 2015"),lty=c(1,5),pch=c(19,15,8,17,18),lwd=2,col=c("black","gold2","blue","green3","red"),ncol=2,horiz=FALSE,cex=0.6,title="months")

abline(v=4,lty=2)

###residual plot

plot(tempo1[tempo1$day==1,"dist"],tempo1[tempo1$day==1,"2"],type = "b",ylim=c(0,2),pch=19,col="black",cex=1.5,xlab="Distance(km)",ylab="Nitrate",main="residuals",lwd=3,lty=1)

points(tempo1[tempo1$day==126,"dist"],tempo1[tempo1$day==126,"2"],type="l",col="gold2",pch=15,cex=1.5,lwd=3,lty=2)

points(tempo1[tempo1$day==260,"dist"],tempo1[tempo1$day==260,"2"],type="l",col="blue",pch=8,cex=1.5,lwd=3,lty=3)

points(tempo1[tempo1$day==336,"dist"],tempo1[tempo1$day==336,"2"],type="l",col="green3",pch=17,cex=1.5,lwd=3,lty=4)

points(tempo1[tempo1$day==518,"dist"],tempo1[tempo1$day==518,"2"],type="l",col="red",pch=18,cex=2,lwd=3,lty=5)

legend("topleft",inset=c(0,0),legend=c("Dec 13", "April 14", "Aug 2014", "Nov 2014","May 2015"),lty=c(1,5),pch=c(19,15,8,17,18),lwd=2,col=c("black","gold2","blue","green3","red"),ncol=2,horiz=FALSE,cex=0.6,title="months")

abline(v=4,lty=2)

plot(tempo) ## checking the diagnostic plots to check the multiple regresstion assumptions

qqnorm(tempo$residuals)

qqline(tempo$residuals)

hist(tempo$residuals)

# Multiple regression analysis for no3 and variables affecting it to check which variables explain spatiotemporal variation in no3)

#### Linear modelling for no3

#tempo<-lm(no3~I(time==3)+eff:dist+eff:I((dno3)/dayflow)+eff:I((dno3)/dayflow):dist+ eff:temp2:dist,data=alldata)

#tempo<-lm(log(no3)~time+eff:dist+eff:I((dno3/dayflow),data=alldata)##final model

#tempo<-lm(log(no3)~time+eff:I(log(dno3)/dayflow):time+eff:I(log(dno3)/dayflow):time:dist,data=alldata)##

#tempo<-lm(log(no3)~temp+toc+time+eff:I(log(dno3)/dayflow):time,data=alldata)#

#tempo<-lm(log(no3)~temp+time+eff:dist+eff:dist:time+eff:I(dist^2):time+eff:I(log(dno3/dayflow))+eff:I(log(dno3/dayflow)):time,data=alldata)##

tempo<-lm(log(no3)~temp+time+alk:time+eff:I(log(dno3)/dayflow)+eff:I(log(dno3)/dayflow):time,data=alldata)##

tempo<-lm(log(no3)~temp+rain2+rain3+alk:time+eff:I(log(dno3)/dayflow)+eff:I(log(dno3)/dayflow):time,data=alldata)####final model2

tempo<-lm(log(no3)~temp+rain2+rain3+eff:I(log(dno3)/dayflow):time+eff:I(log(dno3)/dayflow):I(dist==4.08):time,data=alldata)##final model5 ##*********##

anova(tempo) ##*********##

summary(tempo) ##*********##

tempo1<-cbind(cbind(exp(fitted(tempo)),exp(residuals(tempo))),alldata)

names(tempo1[,1:2])<-c("fitted","residual")

#edit(tempo1)

tempo1[,"2"]

#no3 plots with spatiotemporal model predictions (including other variables)

plot(tempo1[tempo1$day==1,"dist"],tempo1[tempo1$day==1,"1"],type = "l",ylim=c(0,0.7),pch=19,col="black",xlab="Distance(km)",ylab="Nitrate (mg/L)",main="Prediction with environmental variables",lwd=3,lty=1)

points(tempo1[tempo1$day==126,"dist"],tempo1[tempo1$day==126,"1"],type="l",col="gold2",pch=15,lwd=3,cex=1.5,lty=2)

points(tempo1[tempo1$day==260,"dist"],tempo1[tempo1$day==260,"1"],type="l",col="blue",pch=8,cex=1.2,lwd=3,lty=3)

points(tempo1[tempo1$day==336,"dist"],tempo1[tempo1$day==336,"1"],type="l",col="green3",pch=17,cex=1.2,lwd=3,lty=4)

points(tempo1[tempo1$day==518,"dist"],tempo1[tempo1$day==518,"1"],type="l",col="red",pch=18,cex=1.4,lwd=3,lty=5)

legend("topleft",inset=c(0,0),legend=c("Dec 13", "April 14", "Aug 2014", "Nov 2014","May 2015"),lty=c(1,5),pch=c(19,15,8,17,18),lwd=2,col=c("black","gold2","blue","green3","red"),ncol=2,horiz=FALSE,cex=0.6,title="months")

abline(v=4,lty=2)

options( scipen = 1 )

anova(tempo)

anova(tempo,tempo1)

summary(tempo)

avPlots(tempo)

plot(tempo) ## checking the diagnostic plots to check the multiple regresstion assumptions

qqnorm(tempo$residuals)

qqline(tempo$residuals)

hist(tempo$residuals)

tempo.check<-lm(residuals(tempo)~time+eff:time+eff:dist+eff:dist:time+eff:I(dist^2):time,data=alldata)

#tempo.check<-lm(residuals(tempo)~toc,data=alldata)

summary(tempo.check)

anova(tempo.check) # No clear evidence of spatiotemporal patters in residuals

## some more graphs

a<-xyplot(no3~dist,group=time,data=alldata,ylim=c(0,0.8),type="p",pch=19,lty=2,col=c("black","gold2","blue","green3","red"))

b<-xyplot(exp(fitted(tempo))~dist,group=time,data=alldata,type="l",lwd=2,col=c("black","gold2","blue","green3","red"))

c<-xyplot(toc~dist,data=alldata,panel = function(x,y) {panel.abline(v=4,lty=2)})

a+as.layer(b)+as.layer(c)

legend("topleft",inset=c(0,0),legend=c("Dec 13", "April 14", "Aug 2014", "Nov 2014","May 2015"),lty=c(1,5),pch=c(19,15,8,17,18),lwd=2,col=c("black","gold2","blue","green3","red"),ncol=2,horiz=FALSE,cex=0.6,title="months")

xyplot(residuals(tempo)~dist,groups=time,data=alldata,type="b",pch=19,lwd=2,col=c("black","gold2","blue","green3","red"))

xyplot(no3~dist,group=time,data=alldata,type="b",pch=19,lty=2,col=c("black","gold2","blue","green3","red"))

xyplot(nh3~dist,group=time,data=alldata,type="b",pch=19,lty=2,col=c("black","gold2","blue","green3","red"))

xyplot(do~dist,group=time,data=alldata,type="b",pch=19,lty=2,col=c("black","gold2","blue","green3","red"))

xyplot(log(no3)~toc,groups=time,data=alldata,type="p",pch=19:24,lwd=2,col=c("black","gold2","blue","green3","red"))

xyplot(do~toc,groups=time,data=alldata,type="p",pch=19:24,lwd=2,col=c("black","gold2","blue","green3","red"))

xyplot(log(no3)~log(nh3),groups=time,data=alldata,type="p",pch=19:24,lwd=2,col=c("black","gold2","blue","green3","red"))

xyplot(log(nh3)~log(toc),groups=time,data=alldata,type="p",pch=19:24,lwd=2,col=c("black","gold2","blue","green3","red"))

cor.test(alldata$do,alldata$toc)

##***** nitrate plot *****#

tiff(file="nitrate plot.jpg",width=10,height=14,units="in",pointsize = 12,bg ="transparent",res=800,compression="lzw")

par(mfrow=c(3,1), mar=c(3,4.5,2.5,3.5),cex=1.5,cex.axis=0.9,las=1,cex.main=1,cex.lab=0.8)

#Nitrate plotted against spatial position

plot(bugenv[bugenv$day==1,"dist"],bugenv[bugenv$day==1,"no3"],type = "b",ylim=c(0,0.7),pch=19,xlab="Distance(km)",ylab="Nitrate (mg/L)",main="a) Nitrate plotted against spatial position",lwd=3,lty=1)

points(bugenv[bugenv$day==126,"dist"],bugenv[bugenv$day==126,"no3"],type="b",col="gold2",pch=15,lwd=3,cex=1.2,lty=2)

points(bugenv[bugenv$day==260,"dist"],bugenv[bugenv$day==260,"no3"],type="b",col="blue",pch=8,cex=1.2,lwd=3,lty=3)

points(bugenv[bugenv$day==336,"dist"],bugenv[bugenv$day==336,"no3"],type="b",col="green3",pch=17,cex=1.2,lwd=3,lty=4)

points(bugenv[bugenv$day==518,"dist"],bugenv[bugenv$day==518,"no3"],type="b",col="red",pch=18,cex=1.4,lwd=3,lty=5)

legend("topleft",inset=c(0,0),legend=c("Dec 13", "April 14", "Aug 2014", "Nov 2014","May 2015"),lty=c(1,5),pch=c(19,15,8,17,18),lwd=2,col=c("black","gold2","blue","green3","red"),ncol=2,horiz=FALSE,cex=0.6,title="months")

abline(v=4,lty=2)

tempo<-lm(log(no3)~time+eff:time+eff:dist+eff:dist:time+eff:I(dist^2):time,data=alldata) # final model

tempo1<-cbind(cbind(exp(fitted(tempo)),exp(residuals(tempo))),alldata)#[complete.cases(alldata[,c("temp","no3","tp")]),])

names(tempo1[,1:2])<-c("fitted","residual")

tempo1[,"2"]

#no3 with spatiotemporal model predictions

plot(tempo1$dist[tempo1$time==1],tempo1[tempo1$time==1,"no3"],type="p",pch=19,col="black",xlab="Distance (km)",ylab="Nitrate (mg/L)",main="b) Nitrate with spatiotemporal prediction",ylim=c(0,0.7),cex=1,lwd=3,lty=1)

points(tempo1$dist[tempo1$time==1],tempo1[tempo1$time==2,"no3"],col="gold2",pch=15,cex=1.2,lwd=3,lty=2)

points(tempo1$dist[tempo1$time==1],tempo1[tempo1$time==3,"no3"],col="blue",pch=8,cex=1.2,lwd=3,lty=3)

points(tempo1$dist[tempo1$time==1],tempo1[tempo1$time==4,"no3"],col="green3",pch=17,cex=1.2,lwd=3,lty=4)

points(tempo1$dist[tempo1$time==1],tempo1[tempo1$time==5,"no3"],col="red",pch=18,cex=1.4,lwd=3,lty=5)

points(tempo1[tempo1$day==1,"dist"],tempo1[tempo1$day==1,"1"],type="l",col="black",pch=19,cex=1.5,lwd=3,lty=1)

points(tempo1[tempo1$day==126,"dist"],tempo1[tempo1$day==126,"1"],type="l",col="gold2",pch=15,cex=1.5,lwd=3,lty=2)

points(tempo1[tempo1$day==260,"dist"],tempo1[tempo1$day==260,"1"],type="l",col="blue",pch=8,cex=1.5,lwd=3,lty=3)

points(tempo1[tempo1$day==336,"dist"],tempo1[tempo1$day==336,"1"],type="l",col="green3",pch=17,cex=1.5,lwd=3,lty=4)

points(tempo1[tempo1$day==518,"dist"],tempo1[tempo1$day==518,"1"],type="l",col="red",pch=18,cex=2,lwd=3,lty=5)

legend("topleft",inset=c(0,0),legend=c("Dec 13", "April 14", "Aug 2014", "Nov 2014","May 2015"),lty=c(1,5),pch=c(19,15,8,17,18),lwd=2,col=c("black","gold2","blue","green3","red"),ncol=2,horiz=FALSE,cex=0.6,title="months")

abline(v=4,lty=2)

tempo<-lm(log(no3)~temp+time+eff:dist+eff:dist:time+eff:I(dist^2):time+eff:I(log(dno3/dayflow))+eff:I(log(dno3/dayflow)):time,data=alldata)##final model Checked

tempo<-lm(log(no3)~temp+time+eff:I(log(dno3)/dayflow)+eff:I(log(dno3)/dayflow):time+dist,data=alldata)##

tempo<-lm(log(no3)~temp+time+alk:time+eff:I(log(dno3)/dayflow)+eff:I(log(dno3)/dayflow):time,data=alldata)##final model2 Checked

tempo<-lm(log(no3)~temp+rain2+rain3+eff:I(log(dno3)/dayflow):time+eff:I(log(dno3)/dayflow):I(dist==4.08):time,data=alldata)##final model5 Checked

tempo1<-cbind(cbind(exp(fitted(tempo)),exp(residuals(tempo))),alldata)

names(tempo1[,1:2])<-c("fitted","residual")

tempo1[,"2"]

#no3 plots with spatiotemporal model predictions (including other variables)

plot(tempo1[tempo1$day==1,"dist"],tempo1[tempo1$day==1,"1"],type = "b",ylim=c(0,0.7),pch=19,col="black",xlab="Distance(km)",ylab="Nitrate (mg/L)",main="c) Prediction with environmental variables",lwd=3,lty=1)

points(tempo1[tempo1$day==126,"dist"],tempo1[tempo1$day==126,"1"],type="b",col="gold2",pch=15,lwd=3,cex=1.5,lty=2)

points(tempo1[tempo1$day==260,"dist"],tempo1[tempo1$day==260,"1"],type="b",col="blue",pch=8,cex=1.2,lwd=3,lty=3)

points(tempo1[tempo1$day==336,"dist"],tempo1[tempo1$day==336,"1"],type="b",col="green3",pch=17,cex=1.2,lwd=3,lty=4)

points(tempo1[tempo1$day==518,"dist"],tempo1[tempo1$day==518,"1"],type="b",col="red",pch=18,cex=1.4,lwd=3,lty=5)

legend("topleft",inset=c(0,0),legend=c("Dec 13", "April 14", "Aug 2014", "Nov 2014","May 2015"),lty=c(1,5),pch=c(19,15,8,17,18),lwd=2,col=c("black","gold2","blue","green3","red"),ncol=2,horiz=FALSE,cex=0.6,title="months")

abline(v=4,lty=2)

dev.off()

## Nonlinear modelling for no3

##simulations

temp3<-alldata$temp2-20

temp3

no3nlm<-0.01+0.33*(alldata2$time==3)+1.8*alldata2$eff*(alldata2$dno3/alldata2$dayflow)*exp(-0.01*1.1^alldata2$temp2*alldata2$dist/alldata2$vel2)

alldata2<-cbind(alldata,no3nlm,temp3)

par(mar=c(5,5,4,2),cex=0.9)

plot(alldata2$dist[alldata2$time==1],alldata2[alldata2$time==1,"no3"],type="p",pch=19,col="black",xlab="Distance (km)",ylab="Nitrate (mg/L)",main="Nitrate with spatiotemporal prediction",ylim=c(0,0.7),cex=1,lwd=3,lty=1)

points(alldata2$dist[alldata2$time==1],alldata2[alldata2$time==2,"no3"],col="gold2",pch=15,cex=1.2,lwd=3,lty=2)

points(alldata2$dist[alldata2$time==1],alldata2[alldata2$time==3,"no3"],col="blue",pch=8,cex=1.2,lwd=3,lty=3)

points(alldata2$dist[alldata2$time==1],alldata2[alldata2$time==4,"no3"],col="green3",pch=17,cex=1.2,lwd=3,lty=4)

points(alldata2$dist[alldata2$time==1],alldata2[alldata2$time==5,"no3"],col="red",pch=18,cex=1.4,lwd=3,lty=5)

points(alldata2[alldata2$day==126,"dist"],alldata2[alldata2$day==126,"no3nlm"],type="l",col="black",pch=15,lwd=3,cex=1.5,lty=1)

points(alldata2[alldata2$day==126,"dist"],alldata2[alldata2$day==126,"no3nlm"],type="l",col="gold2",pch=15,lwd=3,cex=1.5,lty=2)

points(alldata2[alldata2$day==260,"dist"],alldata2[alldata2$day==260,"no3nlm"],type="l",col="blue",pch=8,cex=1.5,lwd=3,lty=3)

points(alldata2[alldata2$day==336,"dist"],alldata2[alldata2$day==336,"no3nlm"],type="l",col="green3",pch=17,cex=1.5,lwd=3,lty=4)

points(alldata2[alldata2$day==518,"dist"],alldata2[alldata2$day==518,"no3nlm"],type="l",col="red",pch=18,cex=2,lwd=3,lty=5)

legend("topright",inset=c(0,0),legend=c("Dec 13", "April 14", "Aug 2014", "Nov 2014","May 2015"),lty=c(1,5),pch=c(19,15,8,17,18),lwd=2,col=c("black","gold2","blue","green3","red"),ncol=2,horiz=FALSE,cex=0.9,title="months")

abline(v=4,lty=2)

##fitting the non-linear model

no3nls<-c(b1=0.01,b2=0.33,b3=1.8,b4=-0.02,b5=1.05)

no3nls.fm1<-nls(no3~b1+b2*(time==3)+b3*eff*(dno3/dayflow)*exp(b4*b5^temp3*dist/vel2),data=alldata2,start=no3nls,trace=T)

edit(alldata)

summary(no3nls.fm1)

plot(no3nls.fm1)

fm2<-as.matrix(fitted(no3nls.fm1))

attributes(fm2)

edit(fm2)

tempo1<-cbind(fm2,alldata)

attributes(tempo1)

edit(tempo1)

#observed no3 plots with spatiotemporal model predictions

plot(tempo1$dist[tempo1$time==1],tempo1[tempo1$time==1,"no3"],type="p",pch=19,col="black",xlab="Distance (km)",ylab="Nitrate (mg/L)",main="Nitrate with spatiotemporal prediction",ylim=c(0,0.7),cex=1,lwd=3,lty=1)

points(tempo1$dist[tempo1$time==1],tempo1[tempo1$time==2,"no3"],col="gold2",pch=15,cex=1.2,lwd=3,lty=2)

points(tempo1$dist[tempo1$time==1],tempo1[tempo1$time==3,"no3"],col="blue",pch=8,cex=1.2,lwd=3,lty=3)

points(tempo1$dist[tempo1$time==1],tempo1[tempo1$time==4,"no3"],col="green3",pch=17,cex=1.2,lwd=3,lty=4)

points(tempo1$dist[tempo1$time==1],tempo1[tempo1$time==5,"no3"],col="red",pch=18,cex=1.4,lwd=3,lty=5)

points(tempo1[tempo1$day==1,"dist"],tempo1[tempo1$day==1,"fm2"],type="l",col="black",pch=19,cex=1.5,lwd=3,lty=1)

points(tempo1[tempo1$day==126,"dist"],tempo1[tempo1$day==126,"fm2"],type="l",col="gold2",pch=15,cex=1.5,lwd=3,lty=2)

points(tempo1[tempo1$day==260,"dist"],tempo1[tempo1$day==260,"fm2"],type="l",col="blue",pch=8,cex=1.5,lwd=3,lty=3)

points(tempo1[tempo1$day==336,"dist"],tempo1[tempo1$day==336,"fm2"],type="l",col="green3",pch=17,cex=1.5,lwd=3,lty=4)

points(tempo1[tempo1$day==518,"dist"],tempo1[tempo1$day==518,"fm2"],type="l",col="red",pch=18,cex=2,lwd=3,lty=5)

legend("topleft",inset=c(0,0),legend=c("Dec 13", "April 14", "Aug 2014", "Nov 2014","May 2015"),lty=c(1,5),pch=c(19,15,8,17,18),lwd=2,col=c("black","gold2","blue","green3","red"),ncol=2,horiz=FALSE,cex=0.6,title="months")

abline(v=4,lty=2)

#residuals

par(mar=c(5,5,4,2),cex=0.9)

plot(tempo1[tempo1$day==1,"dist"],tempo1[tempo1$day==1,"2"],type = "b",ylim=c(-0.6,0.6),pch=19,cex=1.5,xlab="Distance(km)",ylab="residulas",main="prediction with space and time",lwd=3,lty=4)

points(tempo1[tempo1$day==126,"dist"],tempo1[tempo1$day==126,"2"],type="b",col="gold2",pch=8,cex=1.5,lwd=3,lty=3)

points(tempo1[tempo1$day==260,"dist"],tempo1[tempo1$day==260,"2"],type="b",col="blue",pch=8,cex=1.5,lwd=3,lty=3)

points(tempo1[tempo1$day==336,"dist"],tempo1[tempo1$day==336,"2"],type="b",col="green3",pch=17,cex=1.5,lwd=3)

points(tempo1[tempo1$day==518,"dist"],tempo1[tempo1$day==518,"2"],type="b",col="red",pch=18,cex=2,lwd=3,lty=5)

legend("topright",inset=c(0,0),legend=c("Dec 2013","April 2014", "Aug 2014", "Nov 2014","May 2015"),lty=1,lwd=2,col=c("black","gold2","blue","green3","red"),ncol=5,horiz=FALSE,cex=0.7,title="months")

abline(v=4,lty=2)

xyplot(fitted(tempo)~dist, groups=day,data=tempo1,type="l",auto.key=TRUE)

xyplot(residuals(tempo)~dist, groups=day,data=tempo1,type="l",auto.key=TRUE)

tempo3<-lm(tempo1[,2]~tempo1$pred1)

summary(tempo3)

## residulas against time and space

edit(residuals(tempo))

names(residuals(tempo))

rownames(alldata)

tempo<-lm(tempo1[,2]~as.factor(eff)+dist+time+as.factor(eff):time+as.factor(eff):dist,data=alldata)

summary(tempo)

plot(tempo)

anova(tempo)

###cross-validation

lmfit<-train(log(no3)~temp+time+eff:I(log(dno3)/dayflow)+eff:I(log(dno3)/dayflow):time+dist,data=alldata)##WP2+RH2

lmfit<-train(log(no3)~temp+rain2+rain3+eff:I(log(dno3)/dayflow):time+eff:I(log(dno3)/dayflow):I(dist==4.08):time,data=alldata)##WP5+RH2

print(lmfit)

#

# Total phosphorus (TP)

#check the graph (scatterplot) for tp

par(mar=c(5,5,4,2),cex=0.9)

panel.cor <- function(x, y, digits = 2, cex.cor, ...)

{

usr <- par("usr"); on.exit(par(usr))

par(usr = c(0, 1, 0, 1))

# correlation coefficient

r <- cor(x, y)

method=c("spearman")

txt <- format(c(r, 0.123456789), digits = digits)[1]

txt <- paste("r= ", txt, sep = "")

text(0.5, 0.8, txt)

# p-value calculation

p <- cor.test(x, y)$p.value

txt2 <- format(c(p, 0.123456789), digits = digits)[1]

txt2 <- paste("p= ", txt2, sep = "")

if(p<0.01) txt2 <- paste("p= ", "<0.01", sep = "")

text(0.5, 0.3, txt2)

}

pairs(~ph2+log(alk)+dayflow+log(I(dtp/dayflow))+eff+dist+temp+log(tp),data=alldata,upper.panel=panel.cor,pch=20,na.action = na.omit)

##total phosphorus regression with other variables

tempo<-lm(tp~eff,data=alldata)##Adjusted R-squared: 0.5636

tempo<-lm(tp~dtp,data=alldata)

tempo<-lm(tp~dayflow,data=alldata)

tempo<-lm(tp~dist,data=alldata)##Adjusted R-squared: 0.2565

tempo<-lm(tp~temp,data=alldata)

tempo<-lm(tp~ind1000,data=alldata)

# Multiple regression analysis for tp as a function of time, distance and eff to check spatiotemporal variation of tp

xyplot(log(tp)~dist,groups=time,data=alldata,type="b",pch=19,lwd=2,col=c("black","gold2","blue","green3","red"))

#tempo<-lm(log(tp)~dist+time+eff+eff:time+eff:dist+eff:time:dist,data=alldata)

#tempo<-lm(tp~time+eff:time+eff:dist,data=alldata) #

tempo<-lm(log(tp)~time+eff:time+eff:dist,data=alldata)# final model ##*********##

anova(tempo) ##*********##

summary(tempo) ##*********##

plot(tempo) ## checking the diagnostic plots to check the multiple regresstion assumptions

qqnorm(tempo$residuals)

qqline(tempo$residuals)

hist(tempo$residuals)

#tp with spatiotemporal prediction

tempo1<-cbind(cbind((exp(fitted(tempo))),residuals(tempo)),alldata[complete.cases(alldata[,c("tp","dayflow","dtp")]),])

names(tempo1[,1:2])<-c("fitted","residual")

tempo1[,"2"]

plot(tempo1$dist[tempo1$time==2],tempo1[tempo1$time==2,"tp"],type="p",pch=15,col="gold2",xlab="Distance (km)",ylab="TP (mg/L)",main="TP with spatiotemporal prediction",ylim=c(0,0.8),cex=1,lwd=3,lty=2)

#points(tempo1$dist[tempo1$time==1],tempo1[tempo1$time==2,"tp"],col="gold2",pch=15,cex=1.5,lwd=3,lty=2)

points(tempo1$dist[tempo1$time==2],tempo1[tempo1$time==3,"tp"],col="blue",pch=8,cex=1.2,lwd=3,lty=3)

points(tempo1$dist[tempo1$time==2],tempo1[tempo1$time==4,"tp"],col="green3",pch=17,cex=1.2,lwd=3,lty=4)

points(tempo1$dist[tempo1$time==2],tempo1[tempo1$time==5,"tp"],col="red",pch=18,cex=1.4,lwd=3,lty=5)

points(tempo1[tempo1$day==126,"dist"],tempo1[tempo1$day==126,"1"],type="l",col="gold2",pch=15,cex=1.5,lwd=3,lty=2)

#points(tempo1[tempo1$day==126,"dist"],tempo1[tempo1$day==126,"1"],type="l",col="gold2",pch=15,cex=1.5,lwd=3,lty=2)

points(tempo1[tempo1$day==260,"dist"],tempo1[tempo1$day==260,"1"],type="l",col="blue",pch=8,cex=1.5,lwd=3,lty=3)

points(tempo1[tempo1$day==336,"dist"],tempo1[tempo1$day==336,"1"],type="l",col="green3",pch=17,cex=1.5,lwd=3,lty=4)

points(tempo1[tempo1$day==518,"dist"],tempo1[tempo1$day==518,"1"],type="l",col="red",pch=18,cex=2,lwd=3,lty=5)

legend("topleft",inset=c(0,0),legend=c("Dec 13", "April 14", "Aug 2014", "Nov 2014","May 2015"),lty=c(1,5),pch=c(19,15,8,17,18),lwd=2,col=c("black","gold2","blue","green3","red"),ncol=2,horiz=FALSE,cex=0.6,title="months")

abline(v=4,lty=2)

plot.new()

#residuals plotted aginst distance

par(mar=c(5,5,4,2),cex=0.9)

plot(tempo1[tempo1$day==126,"dist"],tempo1[tempo1$day==126,"2"],col="gold2",type = "b",ylim=c(-1,1),pch=19,cex=1.5,xlab="Distance(km)",ylab="residulas",main="prediction with space and time",lwd=3,lty=4)

#points(tempo1[tempo1$day==126,"dist"],tempo1[tempo1$day==126,"2"],type="b",col="gold2",pch=8,cex=1.5,lwd=3,lty=3)

points(tempo1[tempo1$day==260,"dist"],tempo1[tempo1$day==260,"2"],type="b",col="blue",pch=8,cex=1.5,lwd=3,lty=3)

points(tempo1[tempo1$day==336,"dist"],tempo1[tempo1$day==336,"2"],type="b",col="green3",pch=17,cex=1.5,lwd=3)

points(tempo1[tempo1$day==518,"dist"],tempo1[tempo1$day==518,"2"],type="b",col="red",pch=18,cex=2,lwd=3,lty=5)

legend("topright",inset=c(0,0),legend=c("Dec 2013","April 2014", "Aug 2014", "Nov 2014","May 2015"),lty=1,lwd=2,col=c("black","gold2","blue","green3","red"),ncol=5,horiz=FALSE,cex=0.7,title="months")

abline(v=4,lty=2)

# Multiple regression analysis for tp and factors affecting it to check which variables explain spatiotemporal variation in tp)

alldata$time<-as.factor(alldata$time)

#tempo<-lm(log(tp)~dayflow+dtp+dist+eff+dist:eff,data=alldata)##final model## Adjusted R-squared: 0.8084

#tempo<-lm(log(tp)~eff+log(dtp)+log(dayflow)+I(dist/vel)+temp:I(dist/vel)+I(temp^2):I(dist/vel),data=alldata)##Adjusted R-squared: 0.7669

#tempo<-lm(log(tp)~eff+eff:log(dtp)+eff:log(dayflow)+eff:I(dist/vel)+eff:temp:I(dist/vel)+eff:I(temp^2):I(dist/vel),data=alldata)##Adjusted R-squared: 0.752

#tempo<-lm(log(tp)~dayflow+dtp+dist+eff+dist:eff+I(dist/vel),data=alldata) ##Adjusted R-squared: 0.8025

#tempo<-lm(log(tp)~dayflow+dtp+dist+eff+dist:eff+temp:I(dist/vel),data=alldata)##Adjusted R-squared: 0.8109

#tempo<-lm(log(tp)~dayflow+dtp+dist+eff+dist:eff+temp:I(dist/vel)+I(temp^2):I(dist/vel),data=alldata)##Adjusted R-squared: 0.8237

#tempo<-lm(log(tp)~dayflow+dtp+dist+eff+dist:eff+temp:I(dist/vel)+I(temp^2):I(dist/vel)+eff:log(dtp),data=alldata)##Adjusted R-squared: 0.8177

#tempo<-lm(log(tp)~dayflow+dtp+dist+eff+dist:eff+temp:I(dist/vel)+I(temp^2):I(dist/vel)+eff:log(dayflow),data=alldata)##Adjusted R-squared: 0.8176

#tempo<-lm(log(tp)~dayflow+dtp+dist+eff+dist:eff+temp:I(dist/vel)+I(temp^2):I(dist/vel)+eff:I(dist/vel),data=alldata)##Adjusted R-squared: 0.8198

#tempo<-lm(log(tp)~dayflow+dtp+dist+eff+dist:eff+temp:I(dist/vel)+I(temp^2):I(dist/vel)+eff:temp:I(dist/vel),data=alldata)##Adjusted R-squared: 0.8198

#tempo<-lm(log(tp)~dayflow+dtp+dist+eff+dist:eff+temp:I(dist/vel)+I(temp^2):I(dist/vel)+eff:I(temp^2):I(dist/vel),data=alldata)##Adjusted R-squared: 0.7939

#tempo<-lm(log(tp)~eff+log(dtp)+log(dayflow)+dist:eff+I(dist/vel)+temp:I(dist/vel)+I(temp^2):I(dist/vel)+eff:I(dist/vel),data=alldata)##Adjusted R-squared: 0.7669 ## final model

#tempo<-lm(tp~dayflow+eff+eff:dist+eff:I(dist^2)+eff:dtp+eff:dtp:dist+eff:dtp:I(dist^2)+eff:dayflow+eff:dayflow:dist+eff:dayflow:I(dist^2)+eff:vel+eff:vel:dist+eff:vel:I(dist^2)+eff:temp+eff:temp:dist+eff:temp:I(dist^2),data=alldata)

#tempo<-lm(tp~dayflow+eff+eff:dist+eff:I(dist^2)+eff:dtp+eff:dtp:dist+eff:dtp:I(dist^2)+eff:dayflow+eff:dayflow:dist+eff:vel+eff:vel:dist+eff:temp+eff:temp:dist,data=alldata)### final model## Adjusted R-squared: 0.9517

#edit(wangenv)

#tempo<-lm(log(tp)~rain2+temp+dist+time+dist:time+eff:dist+eff:log(I(dtp/dayflow))+eff:log(I(dtp/dayflow)):dist,data=alldata[complete.cases(alldata[,c("tp")]),]) #

#tempo<-lm(log(tp)~rain2+temp+dist+time+dist:time+eff:dist+eff:log(I(dtp/dayflow))+eff:time:dist,data=alldata[complete.cases(alldata[,c("tp")]),]) #

#tempo<-lm(log(tp)~rain2+temp+dist+time+dist:time+eff:dist+eff:log(I(dtp/dayflow)),data=alldata[complete.cases(alldata[,c("tp")]),]) #

#tempo<-lm(log(tp)~dist+time+eff:I(dtp/dayflow)+eff:dist+eff:dist:time,data=alldata[complete.cases(alldata[,c("tp")]),]) #

#tempo<-lm(log(tp)~time+eff:I(dtp/dayflow)+eff:dist+eff:dist:time,data=alldata[complete.cases(alldata[,c("tp")]),]) #

#tempo<-lm(log(tp)~time+eff:log(I(dtp/dayflow))+eff:time+eff:dist,data=alldata[complete.cases(alldata[,c("tp")]),]) #

#tempo<-lm(log(tp)~alk+ph2+time+eff:log(I(dtp/dayflow))+eff:time,data=alldata[complete.cases(alldata[,c("tp")]),]) #

tempo<-lm(log(tp)~alk+ph2+time+eff:log(I(dtp/dayflow))+eff:time,data=alldata[complete.cases(alldata[,c("tp")]),]) #

tempo<-lm(log(tp)~alk+ph2+ind1000+time+eff:log(I(dtp/dayflow)):time,data=alldata[complete.cases(alldata[,c("tp")]),]) #

tempo<-lm(log(tp)~alk+ph2+time+eff:log(I(dtp/dayflow)):time,data=alldata[complete.cases(alldata[,c("tp")]),]) #

tempo<-lm(tp~alk+time+toc+eff:I(dtp/dayflow)+eff:I(dtp/dayflow):time,data=alldata[complete.cases(alldata[,c("tp")]),])#

tempo<-lm(log(tp)~alk+time+toc+eff:I(dtp/dayflow)+eff:I(dtp/dayflow):time,data=alldata[complete.cases(alldata[,c("tp")]),])#

tempo<-lm(tp~alk+time+toc+eff:I(dtp/dayflow)+eff:I(dtp/dayflow):time,data=alldata[complete.cases(alldata[,c("tp")]),])#

tempo<-lm(log(tp)~alk+ph2+time+eff:I(dtp/dayflow)+eff:(I(dtp/dayflow)):time,data=alldata[complete.cases(alldata[,c("tp")]),]) #

tempo<-lm(log(tp)~alk+ph2+rain2+rain3+eff:I(log(dtp/dayflow)):time+eff:I(log(dtp/dayflow)):I(dist==4.08):time,data=myalldata)

tempo<-lm(tp~alk+ph2+rain2+rain3+eff:I(dtp/dayflow):time+eff:I(dtp/dayflow):I(dist==4.08):time,data=alldata[complete.cases(alldata[,c("tp")]),])# Final model ##*********##

anova(tempo) ##*********##

summary(tempo) ##*********##

sqrt(vif(tempo))

plot(tempo)

tempo.check<-lm(residuals(tempo)~time+eff:time+eff:dist,data=alldata[complete.cases(alldata[,c("tp")]),])

summary(tempo.check)

anova(tempo.check) # No evidence of spatiotemporal patters in residuals

#tp plot# prediction with environmental variables

tempo<-cbind(cbind((fitted(tempo)),expm1(residuals(tempo))),alldata[complete.cases(alldata[,c("tp","dayflow","dtp")]),])

#tempo1<-cbind(cbind(exp(fitted(tempo)),residuals(tempo)),alldata[complete.cases(alldata[,c("tp")]),])

names(tempo1[,1:2])<-c("fitted","residual")

tempo1[,"2"]

plot(tempo1[tempo1$day==126,"dist"],tempo1[tempo1$day==126,"1"],type = "l",ylim=c(0,0.8),pch=15,col="gold2",xlab="Distance(km)",ylab="TP (mg/L)",main="Prediction with environmental variables",lwd=3,lty=2)

#points(tempo1[tempo1$day==126,"dist"],tempo1[tempo1$day==126,"1"],type="l",col="gold2",pch=15,lwd=3,cex=1.5,lty=2)

points(tempo1[tempo1$day==260,"dist"],tempo1[tempo1$day==260,"1"],type="l",col="blue",pch=8,cex=1.2,lwd=3,lty=3)

points(tempo1[tempo1$day==336,"dist"],tempo1[tempo1$day==336,"1"],type="l",col="green3",pch=17,cex=1.2,lwd=3,lty=4)

points(tempo1[tempo1$day==518,"dist"],tempo1[tempo1$day==518,"1"],type="l",col="red",pch=18,cex=1.4,lwd=3,lty=5)

legend("topleft",inset=c(0,0),legend=c("Dec 13", "April 14", "Aug 2014", "Nov 2014","May 2015"),lty=c(1,5),pch=c(19,15,8,17,18),lwd=2,col=c("black","gold2","blue","green3","red"),ncol=2,horiz=FALSE,cex=0.6,title="months")

abline(v=4,lty=2)

#residuals plotted aginst distance

par(mar=c(5,5,4,2),cex=0.9)

#plot(tempo1[tempo1$day==126,"dist"],tempo1[tempo1$day==126,"2"],col="gold2",type = "b",ylim=c(-0.07,0.07),pch=19,cex=1.5,xlab="Distance(km)",ylab="residulas",main="prediction with space and time",lwd=3,lty=4)

plot(tempo1[tempo1$day==126,"dist"],tempo1[tempo1$day==126,"2"],type="b",col="gold2",ylim=c(-0.09,0.09),pch=8,cex=1.5,lwd=3,lty=3)

points(tempo1[tempo1$day==260,"dist"],tempo1[tempo1$day==260,"2"],type="b",col="blue",pch=8,cex=1.5,lwd=3,lty=3)

points(tempo1[tempo1$day==336,"dist"],tempo1[tempo1$day==336,"2"],type="b",col="green3",pch=17,cex=1.5,lwd=3)

points(tempo1[tempo1$day==518,"dist"],tempo1[tempo1$day==518,"2"],type="b",col="red",pch=18,cex=2,lwd=3,lty=5)

legend("topright",inset=c(0,0),legend=c("Dec 2013","April 2014", "Aug 2014", "Nov 2014","May 2015"),lty=1,lwd=2,col=c("black","gold2","blue","green3","red"),ncol=2,horiz=FALSE,cex=0.7,title="months")

abline(v=4,lty=2)

#checking the assumptions by regressing residuals against predicted values

tempo3<-lm(tempo1[,2]~tempo1$pred1)

summary(tempo3)

plot(tempo3)

## residulas against time and space

edit(residuals(tempo))

names(residuals(tempo))

rownames(alldata)

tempo<-lm(tempo1[,2]~time+eff:time+eff:dist,data=alldata[complete.cases(alldata[,c("tp")]),])#

summary(tempo)

plot(tempo)

anova(tempo)

##cross-validation

library(caret)

library(mlbench)

library(mboost)

myalldata<- na.omit(alldata)

lmfit<-train(log(tp)~alk+ph2+time+toc+eff:I(dtp/dayflow)+eff:I(dtp/dayflow):time,data=alldata[complete.cases(alldata[,c("tp")]),])#

lmfit<-train(log(tp)~alk+ph2+rain2+rain3+eff:I(log(dtp/dayflow)):time+eff:I(log(dtp/dayflow)):I(dist==4.08):time,data=myalldata)

print(lmfit)

##***** TP plot *****#

tiff(file="TP plot.jpg",width=10,height=14,units="in",pointsize = 12,bg ="transparent",res=800,compression="lzw")

par(mfrow=c(3,1), mar=c(4.5,4.5,2.5,3),cex=1.5,cex.axis=0.9,las=1,cex.main=1,cex.lab=0.8)

##total phosphorus plotted against spatial position

plot(bugenv[bugenv$day==1,"dist"],bugenv[bugenv$day==1,"tp"],type = "b",ylim=c(0,0.8),pch=19,xlab="",ylab="TP (mg/L)",main="a) TP plotted against spatial position",lwd=3,lty=1)

points(bugenv[bugenv$day==126,"dist"],bugenv[bugenv$day==126,"tp"],type="b",col="gold2",pch=15,lwd=3,cex=1.2,lty=2)

points(bugenv[bugenv$day==260,"dist"],bugenv[bugenv$day==260,"tp"],type="b",col="blue",pch=8,cex=1.2,lwd=3,lty=3)

points(bugenv[bugenv$day==336,"dist"],bugenv[bugenv$day==336,"tp"],type="b",col="green3",pch=17,cex=1.2,lwd=3,lty=4)

points(bugenv[bugenv$day==518,"dist"],bugenv[bugenv$day==518,"tp"],type="b",col="red",pch=18,cex=1.4,lwd=3,lty=5)

legend("topleft",inset=c(0,0),legend=c("Dec 13", "April 14", "Aug 2014", "Nov 2014","May 2015"),lty=c(1,5),pch=c(19,15,8,17,18),lwd=2,col=c("black","gold2","blue","green3","red"),ncol=2,horiz=FALSE,cex=0.6,title="months")

abline(v=4,lty=2)

# total phosphorus with spatiotemporal prediction

tempo<-lm(log(tp)~time+eff:time+eff:dist,data=alldata) # final model

tempo1<-cbind(cbind((exp(fitted(tempo))),residuals(tempo)),alldata[complete.cases(alldata[,c("tp","dayflow","dtp")]),])

names(tempo1[,1:2])<-c("fitted","residual")

tempo1[,"2"]

plot(tempo1$dist[tempo1$time==2],tempo1[tempo1$time==2,"tp"],type="p",pch=15,col="gold2",xlab="",ylab="TP (mg/L)",main="b) TP with spatiotemporal prediction",ylim=c(0,0.8),cex=1,lwd=3,lty=2)

#points(tempo1$dist[tempo1$time==1],tempo1[tempo1$time==2,"tp"],col="gold2",pch=15,cex=1.5,lwd=3,lty=2)

points(tempo1$dist[tempo1$time==2],tempo1[tempo1$time==3,"tp"],col="blue",pch=8,cex=1.2,lwd=3,lty=3)

points(tempo1$dist[tempo1$time==2],tempo1[tempo1$time==4,"tp"],col="green3",pch=17,cex=1.2,lwd=3,lty=4)

points(tempo1$dist[tempo1$time==2],tempo1[tempo1$time==5,"tp"],col="red",pch=18,cex=1.4,lwd=3,lty=5)

points(tempo1[tempo1$day==126,"dist"],tempo1[tempo1$day==126,"1"],type="l",col="gold2",pch=15,cex=1.5,lwd=3,lty=2)

#points(tempo1[tempo1$day==126,"dist"],tempo1[tempo1$day==126,"1"],type="l",col="gold2",pch=15,cex=1.5,lwd=3,lty=2)

points(tempo1[tempo1$day==260,"dist"],tempo1[tempo1$day==260,"1"],type="l",col="blue",pch=8,cex=1.5,lwd=3,lty=3)

points(tempo1[tempo1$day==336,"dist"],tempo1[tempo1$day==336,"1"],type="l",col="green3",pch=17,cex=1.5,lwd=3,lty=4)

points(tempo1[tempo1$day==518,"dist"],tempo1[tempo1$day==518,"1"],type="l",col="red",pch=18,cex=2,lwd=3,lty=5)

legend("topleft",inset=c(0,0),legend=c("Dec 13", "April 14", "Aug 2014", "Nov 2014","May 2015"),lty=c(1,5),pch=c(19,15,8,17,18),lwd=2,col=c("black","gold2","blue","green3","red"),ncol=2,horiz=FALSE,cex=0.6,title="months")

abline(v=4,lty=2)

#prediction with environmental variables

tempo<-lm(log(tp)~alk+ph2+rain2+rain3+eff:I(log(dtp/dayflow)):time+eff:I(log(dtp/dayflow)):I(dist==4.08):time,alldata[complete.cases(alldata[,c("tp","dayflow","dtp")]),])

tempo<-lm(log(tp)~alk+ph2+time+eff:I(dtp/dayflow)+eff:(I(dtp/dayflow)):time,data=alldata[complete.cases(alldata[,c("tp")]),]) #

tempo<-lm(tp~alk+ph2+rain2+rain3+eff:I(dtp/dayflow):time+eff:I(dtp/dayflow):I(dist==4.08):time,data=alldata[complete.cases(alldata[,c("tp")]),])#final model

tempo1<-cbind(cbind((fitted(tempo)),residuals(tempo)),alldata[complete.cases(alldata[,c("tp")]),])

names(tempo1[,1:2])<-c("fitted","residual")

tempo1[,"2"]

plot(tempo1[tempo1$day==126,"dist"],tempo1[tempo1$day==126,"1"],type = "b",ylim=c(0,0.8),pch=15,col="gold2",xlab="Distance(km)",ylab="TP (mg/L)",main="c) Prediction with environmental variables",lwd=3,lty=2)

#points(tempo1[tempo1$day==126,"dist"],tempo1[tempo1$day==126,"1"],type="b",col="gold2",pch=15,lwd=3,cex=1.5,lty=2)

points(tempo1[tempo1$day==260,"dist"],tempo1[tempo1$day==260,"1"],type="b",col="blue",pch=8,cex=1.2,lwd=3,lty=3)

points(tempo1[tempo1$day==336,"dist"],tempo1[tempo1$day==336,"1"],type="b",col="green3",pch=17,cex=1.2,lwd=3,lty=4)

points(tempo1[tempo1$day==518,"dist"],tempo1[tempo1$day==518,"1"],type="b",col="red",pch=18,cex=1.4,lwd=3,lty=5)

legend("topleft",inset=c(0,0),legend=c("Dec 13", "April 14", "Aug 2014", "Nov 2014","May 2015"),lty=c(1,5),pch=c(19,15,8,17,18),lwd=2,col=c("black","gold2","blue","green3","red"),ncol=2,horiz=FALSE,cex=0.6,title="months")

abline(v=4,lty=2)

dev.off()

#

# alkalinity

#check the scatterplot for alkalinity

par(mar=c(5,5,4,2),cex=0.9)

panel.cor <- function(x, y, digits = 2, cex.cor, ...)

{

usr <- par("usr"); on.exit(par(usr))

par(usr = c(0, 1, 0, 1))

# correlation coefficient

r <- cor(x, y)

method=c("spearman")

txt <- format(c(r, 0.123456789), digits = digits)[1]

txt <- paste("r= ", txt, sep = "")

text(0.5, 0.8, txt)

# p-value calculation

p <- cor.test(x, y)$p.value

txt2 <- format(c(p, 0.123456789), digits = digits)[1]

txt2 <- paste("p= ", txt2, sep = "")

if(p<0.01) txt2 <- paste("p= ", "<0.01", sep = "")

text(0.5, 0.3, txt2)

}

pairs(~dist+time+eff+I(log(dalk/dayflow))+log(dayflow)+rain3+log(rain3)+log(alk),data=alldata,upper.panel=panel.cor,pch=20,na.action = na.omit)

##alkalinity regression with other environmental variables

tempo<-lm(alk~eff,data=alldata)##Adjusted R-squared: 0.09709

tempo<-lm(alk~dayflow,data=alldata)##Adjusted R-squared: 0.2583

tempo<-lm(alk~dist,data=alldata)##Adjusted R-squared: 0.01987

tempo<-lm(alk~time,data=alldata)##Adjusted R-squared: 0.6881

tempo<-lm(alk~rain3,data=alldata)

#alk plotted against spatial position

plot(bugenv[bugenv$day==1,"dist"],bugenv[bugenv$day==1,"alk"],type = "b",ylim=c(0,160),pch=19,xlab="Distance(km)",ylab="Alkalinity (mg/L)",main="Alkalinity plotted against spatial position",lwd=3,lty=1)

points(bugenv[bugenv$day==126,"dist"],bugenv[bugenv$day==126,"alk"],type="b",col="gold2",pch=15,lwd=3,cex=1.2,lty=2)

points(bugenv[bugenv$day==260,"dist"],bugenv[bugenv$day==260,"alk"],type="b",col="blue",pch=8,cex=1.2,lwd=3,lty=3)

points(bugenv[bugenv$day==336,"dist"],bugenv[bugenv$day==336,"alk"],type="b",col="green3",pch=17,cex=1.2,lwd=3,lty=4)

points(bugenv[bugenv$day==518,"dist"],bugenv[bugenv$day==518,"alk"],type="b",col="red",pch=18,cex=1.4,lwd=3,lty=5)

legend("topleft",inset=c(0,0),legend=c("Dec 13", "April 14", "Aug 2014", "Nov 2014","May 2015"),lty=c(1,5),pch=c(19,15,8,17,18),lwd=2,col=c("black","gold2","blue","green3","red"),ncol=2,horiz=FALSE,cex=0.6,title="months")

abline(v=4,lty=2)

# Multiple regression analysis for alkalinity vs time, distance and eff to check spatiotemporal variation of alkalinity

#turbidity with spatiotemporal prediction

tempo<-lm(log(alk)~time+dist+eff+eff:time+eff:dist:time+eff:I(dist^2):time,data=alldata)#RH

tempo.a<-lm(log(alk)~time+dist+eff+eff:time+eff:dist:time+eff:I(dist^2):time,data=alldata)#RH

tempo.b<-lm(log(alk)~time+eff+eff:time+eff:dist:time+eff:I(dist^2):time,data=alldata)#RH

anova(tempo.b,tempo.a)

tempo<-lm(log(alk)~time+eff+eff:time+eff:dist+eff:I(dist^2)+eff:dist:time+eff:I(dist^2):time,data=alldata)#Checked final model ##*********###

anova(tempo) ##*********###

summary(tempo) ##*********###

tempo1<-cbind(cbind(exp(fitted(tempo)),residuals(tempo)),alldata)

attributes(tempo1)

names(tempo1[,1:2])<-c("fitted","residual")

tempo1[,"2"]

#observed alk plots with spatiotemporal model predictions

plot(tempo1$dist[tempo1$time==1],tempo1[tempo1$time==1,"alk"],type="p",pch=19,col="black",xlab="Distance (km)",ylab="Alkalinity (mg/L)",main="Alkalinity with spatiotemporal prediction",ylim=c(0,160),cex=1,lwd=3,lty=1)

points(tempo1$dist[tempo1$time==1],tempo1[tempo1$time==2,"alk"],col="gold2",pch=15,cex=1.2,lwd=3,lty=2)

points(tempo1$dist[tempo1$time==1],tempo1[tempo1$time==3,"alk"],col="blue",pch=8,cex=1.2,lwd=3,lty=3)

points(tempo1$dist[tempo1$time==1],tempo1[tempo1$time==4,"alk"],col="green3",pch=17,cex=1.2,lwd=3,lty=4)

points(tempo1$dist[tempo1$time==1],tempo1[tempo1$time==5,"alk"],col="red",pch=18,cex=1.4,lwd=3,lty=5)

points(tempo1[tempo1$day==1,"dist"],tempo1[tempo1$day==1,"1"],type="l",col="black",pch=19,cex=1.2,lwd=3,lty=1)

points(tempo1[tempo1$day==126,"dist"],tempo1[tempo1$day==126,"1"],type="l",col="gold2",pch=15,cex=1.5,lwd=3,lty=2)

points(tempo1[tempo1$day==260,"dist"],tempo1[tempo1$day==260,"1"],type="l",col="blue",pch=8,cex=1.5,lwd=3,lty=3)

points(tempo1[tempo1$day==336,"dist"],tempo1[tempo1$day==336,"1"],type="l",col="green3",pch=17,cex=1.5,lwd=3,lty=4)

points(tempo1[tempo1$day==518,"dist"],tempo1[tempo1$day==518,"1"],type="l",col="red",pch=18,cex=2,lwd=3,lty=5)

legend("topleft",inset=c(0,0),legend=c("Dec 13", "April 14", "Aug 2014", "Nov 2014","May 2015"),lty=c(1,5),pch=c(19,15,8,17,18),lwd=2,col=c("black","gold2","blue","green3","red"),ncol=2,horiz=FALSE,cex=0.6,title="months")

abline(v=4,lty=2)

plot(tempo) ## checking the diagnostic plots to check the multiple regresstion assumptions

qqnorm(tempo$residuals)

qqline(tempo$residuals)

hist(tempo$residuals)

#residuals against distance

par(mar=c(5,5,4,2),cex=0.9)

plot(tempo1[tempo1$day==1,"dist"],tempo1[tempo1$day==1,"2"],type = "b",ylim=c(-0.3,0.4),pch=19,cex=1.5,xlab="Distance(km)",ylab="residulas",main="prediction with space and time",lwd=3,lty=4)

points(tempo1[tempo1$day==126,"dist"],tempo1[tempo1$day==126,"2"],type="b",col="gold2",pch=8,cex=1.5,lwd=3,lty=3)

points(tempo1[tempo1$day==260,"dist"],tempo1[tempo1$day==260,"2"],type="b",col="blue",pch=8,cex=1.5,lwd=3,lty=3)

points(tempo1[tempo1$day==336,"dist"],tempo1[tempo1$day==336,"2"],type="b",col="green3",pch=17,cex=1.5,lwd=3)

points(tempo1[tempo1$day==518,"dist"],tempo1[tempo1$day==518,"2"],type="b",col="red",pch=18,cex=2,lwd=3,lty=5)

legend("topright",inset=c(0,0),legend=c("Dec 2013","April 2014", "Aug 2014", "Nov 2014","May 2015"),lty=1,lwd=2,col=c("black","gold2","blue","green3","red"),ncol=5,horiz=FALSE,cex=0.7,title="months")

abline(v=4,lty=2)

#alkalinity linear model

tempo<-lm(log(alk)~dayflow+log(dalk):dist+time+eff,data=alldata)

tempo<-lm(log(alk)~dayflow+log(dalk):time+time+eff:time:I(dalk/dayflow)+eff:I(dist^2):time+eff:dist:time,data=alldata)

tempo<-lm(log(alk)~time+dayflow+log(dalk):time+eff:time:I(dalk/dayflow)+eff:I(dist^2):time+eff:dist:time,data=alldata)

tempo<-lm(log(alk)~time+eff:time:dayflow+eff:I(dist^2):time+eff:dist:time,data=alldata)#final model# RH

tempo<-lm(log(alk)~rain3+dayflow+eff:I(dalk/dayflow)+eff:I(dalk/dayflow):time+eff:dist+eff:I(dist^2)+eff:dist:time+eff:I(dist^2):time,data=alldata) # Checked model

tempo<-lm(log(alk)~rain3+dayflow+toc+eff:I(dalk/dayflow)+eff:I(dalk/dayflow):time,data=alldata) # Checked model2

tempo<-lm(log(alk)~rain3+dayflow+eff:I(log(dalk/dayflow)):time+eff:I(log(dalk/dayflow)):I(dist==4.08):time,data=alldata) # Checked model5

anova(tempo) ##*********###

summary(tempo) ##*********###

plot(tempo)

sqrt(vif(tempo))

tempo.check<-lm(residuals(tempo)~time+eff+eff:time+eff:dist+eff:I(dist^2)+eff:dist:time+eff:I(dist^2):time,data=alldata)

summary(tempo.check)

anova(tempo.check) # No clear evidence of spatiotemporal patters in residuals

anova(tempo,tempo1)

#alkalinity prediction with environmental variables

tempo1<-cbind(cbind(exp(fitted(tempo)),residuals(tempo)),alldata)

anova(tempo)

attributes(tempo1)

names(tempo1[,1:2])<-c("fitted","residual")

tempo1[,"2"]

plot(tempo1[tempo1$day==1,"dist"],tempo1[tempo1$day==1,"1"],type = "l",ylim=c(0,160),pch=19,col="black",xlab="Distance(km)",ylab="Alkalinity (NTU)",main="Prediction with environmental parameters",lwd=3,lty=1)

points(tempo1[tempo1$day==126,"dist"],tempo1[tempo1$day==126,"1"],type="l",col="gold2",pch=15,lwd=3,cex=1.5,lty=2)

points(tempo1[tempo1$day==260,"dist"],tempo1[tempo1$day==260,"1"],type="l",col="blue",pch=8,cex=1.2,lwd=3,lty=3)

points(tempo1[tempo1$day==336,"dist"],tempo1[tempo1$day==336,"1"],type="l",col="green3",pch=17,cex=1.2,lwd=3,lty=4)

points(tempo1[tempo1$day==518,"dist"],tempo1[tempo1$day==518,"1"],type="l",col="red",pch=18,cex=1.4,lwd=3,lty=5)

legend("topleft",inset=c(0,0),legend=c("Dec 13", "April 14", "Aug 2014", "Nov 2014","May 2015"),lty=c(1,5),pch=c(19,15,8,17,18),lwd=2,col=c("black","gold2","blue","green3","red"),ncol=2,horiz=FALSE,cex=0.6,title="months")

abline(v=4,lty=2)

#residuals against distance

par(mar=c(5,5,4,2),cex=0.9)

plot(tempo1[tempo1$day==1,"dist"],tempo1[tempo1$day==1,"2"],type = "b",ylim=c(-0.3,0.6),pch=19,cex=1.5,xlab="Distance(km)",ylab="residulas",main="prediction with space and time",lwd=3,lty=4)

points(tempo1[tempo1$day==126,"dist"],tempo1[tempo1$day==126,"2"],type="b",col="gold2",pch=8,cex=1.5,lwd=3,lty=3)

points(tempo1[tempo1$day==260,"dist"],tempo1[tempo1$day==260,"2"],type="b",col="blue",pch=8,cex=1.5,lwd=3,lty=3)

points(tempo1[tempo1$day==336,"dist"],tempo1[tempo1$day==336,"2"],type="b",col="green3",pch=17,cex=1.5,lwd=3)

points(tempo1[tempo1$day==518,"dist"],tempo1[tempo1$day==518,"2"],type="b",col="red",pch=18,cex=2,lwd=3,lty=5)

legend("topright",inset=c(0,0),legend=c("Dec 2013","April 2014", "Aug 2014", "Nov 2014","May 2015"),lty=1,lwd=2,col=c("black","gold2","blue","green3","red"),ncol=5,horiz=FALSE,cex=0.7,title="months")

abline(v=4,lty=2)

## residuals regressed against predicted values

tempo3<-lm(tempo1[,2]~tempo1$pred1)

summary(tempo3)

## residulas against time and space

edit(residuals(tempo))

names(residuals(tempo))

rownames(alldata)

tempo<-lm(tempo1[,2]~time+eff+eff:time+eff:dist+eff:I(dist^2)+eff:dist:time+eff:I(dist^2):time,data=alldata)

summary(tempo)

plot(tempo)

anova(tempo)

###*********#####alkalinity figure

tiff(file="Alkalinity figure.jpg",width=10,height=14,units="in",pointsize = 12,bg ="transparent",res=800,compression="lzw")

par(mfrow=c(3,1), mar=c(3,4.5,2.5,3.5),cex=1.5,cex.axis=0.9,las=1,cex.main=1,cex.lab=0.8)

#alkalinity plotted against spatial position

plot(alldata[alldata$day==1,"dist"],alldata[alldata$day==1,"alk"],type = "b",ylim=c(0,160),pch=19,xlab="Distance(km)",ylab="Alkalinity (mg/L)",main="a) Alkalinity plotted against spatial position",lwd=3,lty=1)

points(alldata[alldata$day==126,"dist"],alldata[alldata$day==126,"alk"],type="b",col="gold2",pch=15,lwd=3,cex=1.2,lty=2)

points(alldata[alldata$day==260,"dist"],alldata[alldata$day==260,"alk"],type="b",col="blue",pch=8,cex=1.2,lwd=3,lty=3)

points(alldata[alldata$day==336,"dist"],alldata[alldata$day==336,"alk"],type="b",col="green3",pch=17,cex=1.2,lwd=3,lty=4)

points(alldata[alldata$day==518,"dist"],alldata[alldata$day==518,"alk"],type="b",col="red",pch=18,cex=1.4,lwd=3,lty=5)

legend("topleft",inset=c(0,0),legend=c("Dec 13", "April 14", "Aug 2014", "Nov 2014","May 2015"),lty=c(1,5),pch=c(19,15,8,17,18),lwd=2,col=c("black","gold2","blue","green3","red"),ncol=2,horiz=FALSE,cex=0.6,title="months")

abline(v=4,lty=2)

#alkalinity with spatiotemporal prediction

tempo<-lm(log(alk)~time+eff+eff:time+eff:dist+eff:I(dist^2)+eff:dist:time+eff:I(dist^2):time,data=alldata)#Checked model

summary(tempo)

tempo1<-cbind(cbind(exp(fitted(tempo)),residuals(tempo)),alldata)

attributes(tempo1)

names(tempo1[,1:2])<-c("fitted","residual")

tempo1[,"2"]

plot(tempo1$dist[tempo1$time==1],tempo1[tempo1$time==1,"alk"],type="p",pch=19,col="black",xlab="Distance (km)",ylab="Alkalinity (mg/L)",main="b) Alkalinity with spatiotemporal prediction",ylim=c(0,160),cex=1,lwd=3,lty=1)

points(tempo1$dist[tempo1$time==1],tempo1[tempo1$time==2,"alk"],col="gold2",pch=15,cex=1.2,lwd=3,lty=2)

points(tempo1$dist[tempo1$time==1],tempo1[tempo1$time==3,"alk"],col="blue",pch=8,cex=1.2,lwd=3,lty=3)

points(tempo1$dist[tempo1$time==1],tempo1[tempo1$time==4,"alk"],col="green3",pch=17,cex=1.2,lwd=3,lty=4)

points(tempo1$dist[tempo1$time==1],tempo1[tempo1$time==5,"alk"],col="red",pch=18,cex=1.4,lwd=3,lty=5)

points(tempo1[tempo1$day==1,"dist"],tempo1[tempo1$day==1,"1"],type="l",col="black",pch=19,cex=1.2,lwd=3,lty=1)

points(tempo1[tempo1$day==126,"dist"],tempo1[tempo1$day==126,"1"],type="l",col="gold2",pch=15,cex=1.5,lwd=3,lty=2)

points(tempo1[tempo1$day==260,"dist"],tempo1[tempo1$day==260,"1"],type="l",col="blue",pch=8,cex=1.5,lwd=3,lty=3)

points(tempo1[tempo1$day==336,"dist"],tempo1[tempo1$day==336,"1"],type="l",col="green3",pch=17,cex=1.5,lwd=3,lty=4)

points(tempo1[tempo1$day==518,"dist"],tempo1[tempo1$day==518,"1"],type="l",col="red",pch=18,cex=2,lwd=3,lty=5)

legend("topleft",inset=c(0,0),legend=c("Dec 13", "April 14", "Aug 2014", "Nov 2014","May 2015"),lty=c(1,5),pch=c(19,15,8,17,18),lwd=2,col=c("black","gold2","blue","green3","red"),ncol=2,horiz=FALSE,cex=0.6,title="months")

abline(v=4,lty=2)

#alkalinity prediction with environmental variables

tempo<-lm(log(alk)~rain3+dayflow+toc+eff:I(dalk/dayflow)+eff:I(dalk/dayflow):time,data=alldata) # Checked model2

tempo<-lm(log(alk)~rain3+dayflow+eff:I(log(dalk/dayflow)):time+eff:I(log(dalk/dayflow)):I(dist==4.08):time,data=alldata) # Checked model5

tempo1<-cbind(cbind(exp(fitted(tempo)),residuals(tempo)),alldata)

anova(tempo)

attributes(tempo1)

names(tempo1[,1:2])<-c("fitted","residual")

tempo1[,"2"]

plot(tempo1[tempo1$day==1,"dist"],tempo1[tempo1$day==1,"1"],type = "b",ylim=c(0,160),pch=19,col="black",xlab="Distance(km)",ylab="Alkalinity (NTU)",main="c) Prediction with environmental parameters",lwd=3,lty=1)

points(tempo1[tempo1$day==126,"dist"],tempo1[tempo1$day==126,"1"],type="b",col="gold2",pch=15,lwd=3,cex=1.5,lty=2)

points(tempo1[tempo1$day==260,"dist"],tempo1[tempo1$day==260,"1"],type="b",col="blue",pch=8,cex=1.2,lwd=3,lty=3)

points(tempo1[tempo1$day==336,"dist"],tempo1[tempo1$day==336,"1"],type="b",col="green3",pch=17,cex=1.2,lwd=3,lty=4)

points(tempo1[tempo1$day==518,"dist"],tempo1[tempo1$day==518,"1"],type="b",col="red",pch=18,cex=1.4,lwd=3,lty=5)

legend("topleft",inset=c(0,0),legend=c("Dec 13", "April 14", "Aug 2014", "Nov 2014","May 2015"),lty=c(1,5),pch=c(19,15,8,17,18),lwd=2,col=c("black","gold2","blue","green3","red"),ncol=2,horiz=FALSE,cex=0.6,title="months")

abline(v=4,lty=2)

dev.off()

#

# pH

tempo<-lm(ph2~time,data=alldata)##

anova(tempo) ##*********###

summary(tempo) ##*********###

tempo1<-cbind(cbind((fitted(tempo)),residuals(tempo)),alldata)

attributes(tempo1)

names(tempo1[,1:2])<-c("fitted","residual")

tempo1[,"2"]

#observed ph plots with spatiotemporal model predictions

plot(tempo1$dist[tempo1$time==1],tempo1[tempo1$time==1,"ph2"],type="p",pch=19,col="black",xlab="Distance (km)",ylab="pH",main="pH with spatiotemporal prediction",ylim=c(6.5,9),cex=1,lwd=3,lty=1)

points(tempo1$dist[tempo1$time==1],tempo1[tempo1$time==2,"ph2"],col="gold2",pch=15,cex=1.2,lwd=3,lty=2)

points(tempo1$dist[tempo1$time==1],tempo1[tempo1$time==3,"ph2"],col="blue",pch=8,cex=1.2,lwd=3,lty=3)

points(tempo1$dist[tempo1$time==1],tempo1[tempo1$time==4,"ph2"],col="green3",pch=17,cex=1.2,lwd=3,lty=4)

points(tempo1$dist[tempo1$time==1],tempo1[tempo1$time==5,"ph2"],col="red",pch=18,cex=1.4,lwd=3,lty=5)

points(tempo1[tempo1$day==1,"dist"],tempo1[tempo1$day==1,"1"],type="l",col="black",pch=19,cex=1.2,lwd=3,lty=1)

points(tempo1[tempo1$day==126,"dist"],tempo1[tempo1$day==126,"1"],type="l",col="gold2",pch=15,cex=1.5,lwd=3,lty=2)

points(tempo1[tempo1$day==260,"dist"],tempo1[tempo1$day==260,"1"],type="l",col="blue",pch=8,cex=1.5,lwd=3,lty=3)

points(tempo1[tempo1$day==336,"dist"],tempo1[tempo1$day==336,"1"],type="l",col="green3",pch=17,cex=1.5,lwd=3,lty=4)

points(tempo1[tempo1$day==518,"dist"],tempo1[tempo1$day==518,"1"],type="l",col="red",pch=18,cex=2,lwd=3,lty=5)

legend("topleft",inset=c(0,0),legend=c("Dec 13", "April 14", "Aug 2014", "Nov 2014","May 2015"),lty=c(1,5),pch=c(19,15,8,17,18),lwd=2,col=c("black","gold2","blue","green3","red"),ncol=2,horiz=FALSE,cex=0.6,title="months")

abline(v=4,lty=2)

tempo<-lm(ph2~rain2,data=alldata) # #Checked

anova(tempo) ##*********###

summary(tempo) ##*********###

tempo1<-cbind(cbind((fitted(tempo)),residuals(tempo)),alldata)

attributes(tempo1)

names(tempo1[,1:2])<-c("fitted","residual")

tempo1[,"2"]

plot(tempo1[tempo1$day==1,"dist"],tempo1[tempo1$day==1,"1"],type = "l",ylim=c(6.5,9),pch=19,col="black",xlab="Distance(km)",ylab="pH",main="Prediction with environmental parameters",lwd=3,lty=1)

points(tempo1[tempo1$day==126,"dist"],tempo1[tempo1$day==126,"1"],type="l",col="gold2",pch=15,lwd=3,cex=1.5,lty=2)

points(tempo1[tempo1$day==260,"dist"],tempo1[tempo1$day==260,"1"],type="l",col="blue",pch=8,cex=1.2,lwd=3,lty=3)

points(tempo1[tempo1$day==336,"dist"],tempo1[tempo1$day==336,"1"],type="l",col="green3",pch=17,cex=1.2,lwd=3,lty=4)

points(tempo1[tempo1$day==518,"dist"],tempo1[tempo1$day==518,"1"],type="l",col="red",pch=18,cex=1.4,lwd=3,lty=5)

legend("topleft",inset=c(0,0),legend=c("Dec 13", "April 14", "Aug 2014", "Nov 2014","May 2015"),lty=c(1,5),pch=c(19,15,8,17,18),lwd=2,col=c("black","gold2","blue","green3","red"),ncol=2,horiz=FALSE,cex=0.6,title="months")

abline(v=4,lty=2)

dev.off()

#

# turbidity

#check the graph (scatterplot) for turbidity

par(mar=c(5,5,4,2),cex=0.9)

panel.cor <- function(x, y, digits = 2, cex.cor, ...)

{

usr <- par("usr"); on.exit(par(usr))

par(usr = c(0, 1, 0, 1))

# correlation coefficient

r <- cor(x, y)

method=c("spearman")

txt <- format(c(r, 0.123456789), digits = digits)[1]

txt <- paste("r= ", txt, sep = "")

text(0.5, 0.8, txt)

# p-value calculation

p <- cor.test(x, y)$p.value

txt2 <- format(c(p, 0.123456789), digits = digits)[1]

txt2 <- paste("p= ", txt2, sep = "")

if(p<0.01) txt2 <- paste("p= ", "<0.01", sep = "")

text(0.5, 0.3, txt2)

}

pairs(~time+dayflow+weekflow+monflow+eff+dturb+vel+rain2+solar+log(rain2)+veg30m+res300+depth+log(cond)+turb+log(turb),data=alldata,upper.panel=panel.cor,pch=20,na.action = na.omit)

##turbidity regression with other environmental variables

tempo<-lm(turb~rain3,data=alldata)##Adjusted R-squared: 0.3849

tempo<-lm(turb~vel,data=alldata)##Adjusted R-squared: 0.08757

tempo<-lm(turb~dayflow,data=alldata)

tempo<-lm(turb~eff,data=alldata)

tempo<-lm(turb~dturb,data=alldata)

tempo<-lm(turb~veg30m,data=alldata)

tempo<-lm(turb~depth,data=alldata)##Adjusted R-squared: 0.1378

#turbidity plotted against spatial position

plot(bugenv[bugenv$day==1,"dist"],bugenv[bugenv$day==1,"turb"],type = "b",ylim=c(0,100),pch=19,xlab="Distance(km)",ylab="Turbidity (NTU)",main="Turbidity plotted against spatial position",lwd=3,lty=1)

points(bugenv[bugenv$day==126,"dist"],bugenv[bugenv$day==126,"turb"],type="b",col="gold2",pch=15,lwd=3,cex=1.2,lty=2)

points(bugenv[bugenv$day==260,"dist"],bugenv[bugenv$day==260,"turb"],type="b",col="blue",pch=8,cex=1.2,lwd=3,lty=3)

points(bugenv[bugenv$day==336,"dist"],bugenv[bugenv$day==336,"turb"],type="b",col="green3",pch=17,cex=1.2,lwd=3,lty=4)

points(bugenv[bugenv$day==518,"dist"],bugenv[bugenv$day==518,"turb"],type="b",col="red",pch=18,cex=1.4,lwd=3,lty=5)

legend("topleft",inset=c(0,0),legend=c("Dec 13", "April 14", "Aug 2014", "Nov 2014","May 2015"),lty=c(1,5),pch=c(19,15,8,17,18),lwd=2,col=c("black","gold2","blue","green3","red"),ncol=2,horiz=FALSE,cex=0.6,title="months")

abline(v=4,lty=2)

# Multiple regression analysis for turbidity as a function of time, distance and eff to check spatiotemporal variation of turbidity

# turbidity with spatiotemporal prediction

tempo<-lm(log(turb)~dist+time+dist:time,data=alldata) # final checked model ###*********###

anova(tempo) ###*********###

summary(tempo)###*********###

tempo1<-cbind(cbind(exp(fitted(tempo)),residuals(tempo)),alldata)

attributes(tempo1)

names(tempo1[,1:2])<-c("fitted","residual")

tempo1[,"2"]

#turbidity with spatiotemporal model predictions

plot(tempo1$dist[tempo1$time==1],tempo1[tempo1$time==1,"turb"],type="p",pch=19,col="black",xlab="Distance (km)",ylab="Turbidity (NTU)",main="Turbidity with spatiotemporal prediction",ylim=c(0,100),cex=1,lwd=3,lty=1)

points(tempo1$dist[tempo1$time==1],tempo1[tempo1$time==2,"turb"],col="gold2",pch=15,cex=1.2,lwd=3,lty=2)

points(tempo1$dist[tempo1$time==1],tempo1[tempo1$time==3,"turb"],col="blue",pch=8,cex=1.2,lwd=3,lty=3)

points(tempo1$dist[tempo1$time==1],tempo1[tempo1$time==4,"turb"],col="green3",pch=17,cex=1.2,lwd=3,lty=4)

points(tempo1$dist[tempo1$time==1],tempo1[tempo1$time==5,"turb"],col="red",pch=18,cex=1.4,lwd=3,lty=5)

points(tempo1[tempo1$day==1,"dist"],tempo1[tempo1$day==1,"1"],type="l",col="black",pch=19,cex=1.2,lwd=3,lty=1)

points(tempo1[tempo1$day==126,"dist"],tempo1[tempo1$day==126,"1"],type="l",col="gold2",pch=15,cex=1.5,lwd=3,lty=2)

points(tempo1[tempo1$day==260,"dist"],tempo1[tempo1$day==260,"1"],type="l",col="blue",pch=8,cex=1.5,lwd=3,lty=3)

points(tempo1[tempo1$day==336,"dist"],tempo1[tempo1$day==336,"1"],type="l",col="green3",pch=17,cex=1.5,lwd=3,lty=4)

points(tempo1[tempo1$day==518,"dist"],tempo1[tempo1$day==518,"1"],type="l",col="red",pch=18,cex=2,lwd=3,lty=5)

legend("topleft",inset=c(0,0),legend=c("Dec 13", "April 14", "Aug 2014", "Nov 2014","May 2015"),lty=c(1,5),pch=c(19,15,8,17,18),lwd=2,col=c("black","gold2","blue","green3","red"),ncol=2,horiz=FALSE,cex=0.6,title="months")

abline(v=4,lty=2)

anova(tempo)

summary(tempo)

plot(tempo) ## checking the diagnostic plots to check the multiple regresstion assumptions

qqnorm(tempo$residuals)

qqline(tempo$residuals)

hist(tempo$residuals)

#residuals against distance

par(mar=c(5,5,4,2),cex=0.9)

plot(tempo1[tempo1$day==1,"dist"],tempo1[tempo1$day==1,"2"],type = "b",ylim=c(-0.3,0.4),pch=19,cex=1.5,xlab="Distance(km)",ylab="residulas",main="prediction with space and time",lwd=3,lty=4)

points(tempo1[tempo1$day==126,"dist"],tempo1[tempo1$day==126,"2"],type="b",col="gold2",pch=8,cex=1.5,lwd=3,lty=3)

points(tempo1[tempo1$day==260,"dist"],tempo1[tempo1$day==260,"2"],type="b",col="blue",pch=8,cex=1.5,lwd=3,lty=3)

points(tempo1[tempo1$day==336,"dist"],tempo1[tempo1$day==336,"2"],type="b",col="green3",pch=17,cex=1.5,lwd=3)

points(tempo1[tempo1$day==518,"dist"],tempo1[tempo1$day==518,"2"],type="b",col="red",pch=18,cex=2,lwd=3,lty=5)

legend("topright",inset=c(0,0),legend=c("Dec 2013","April 2014", "Aug 2014", "Nov 2014","May 2015"),lty=1,lwd=2,col=c("black","gold2","blue","green3","red"),ncol=5,horiz=FALSE,cex=0.7,title="months")

abline(v=4,lty=2)

###turbidity linear model

# Multiple regression analysis for turbidity and factors affecting it to check which variables explain spatiotemporal variation in turbidity

alldata$time<-as.factor(alldata$time)

#tempo<-lm(turb~dturb+vel+dayflow+rain3+eff,data=alldata)## all the variables with arrows pointing to turbidity

tempo<-lm(turb~dturb+vel+dayflow+rain3+dturb:rain3,data=alldata)## all the significant variables ## eff p-value=0.7143 so I took it out##Adjusted R-squared: 0.8589

#tempo<-lm(turb~depth+vel+dayflow+rain3+dturb:rain3,data=alldata)

tempo<-lm(log(turb)~dayflow+I(dayflow^2)+graz1000+res300+graz1000:dayflow+graz1000:I(dayflow^2),data=alldata)

tempo<-lm(log(turb)~log(rain3)+log(dayflow)+log(res300)+log(rain3):log(res300),data=alldata)#final model3

tempo<-lm(log(turb)~log(rain3)+log(dayflow)+log(vel),data=alldata)##final model5

tempo1<-cbind(cbind(exp(fitted(tempo)),residuals(tempo)),alldata)

attributes(tempo1)

names(tempo1[,1:2])<-c("fitted","residual")

tempo1[,"2"]

plot(tempo1[tempo1$day==1,"dist"],tempo1[tempo1$day==1,"1"],type = "l",ylim=c(0,100),pch=19,col="black",xlab="Distance(km)",ylab="Turbidity (NTU)",main="Prediction with environmental parameters",lwd=3,lty=1)

points(tempo1[tempo1$day==126,"dist"],tempo1[tempo1$day==126,"1"],type="l",col="gold2",pch=15,lwd=3,cex=1.5,lty=2)

points(tempo1[tempo1$day==260,"dist"],tempo1[tempo1$day==260,"1"],type="l",col="blue",pch=8,cex=1.2,lwd=3,lty=3)

points(tempo1[tempo1$day==336,"dist"],tempo1[tempo1$day==336,"1"],type="l",col="green3",pch=17,cex=1.2,lwd=3,lty=4)

points(tempo1[tempo1$day==518,"dist"],tempo1[tempo1$day==518,"1"],type="l",col="red",pch=18,cex=1.4,lwd=3,lty=5)

legend("topleft",inset=c(0,0),legend=c("Dec 13", "April 14", "Aug 2014", "Nov 2014","May 2015"),lty=c(1,5),pch=c(19,15,8,17,18),lwd=2,col=c("black","gold2","blue","green3","red"),ncol=2,horiz=FALSE,cex=0.6,title="months")

abline(v=4,lty=2)

summary(tempo)

anova(tempo)

sqrt(vif(tempo))

plot(tempo)

tempo.check<-lm(residuals(tempo)~dist+time+dist:time,data=alldata)

#tempo.check<-lm(residuals(tempo)~rain2,data=alldata)

summary(tempo.check)

anova(tempo.check) # No clear evidence of spatiotemporal patters in residuals

#residuals against distance

par(mar=c(5,5,4,2),cex=0.9)

plot(tempo1[tempo1$day==1,"dist"],tempo1[tempo1$day==1,"2"],type = "b",ylim=c(-0.3,0.4),pch=19,cex=1.5,xlab="Distance(km)",ylab="residulas",main="prediction with space and time",lwd=3,lty=4)

points(tempo1[tempo1$day==126,"dist"],tempo1[tempo1$day==126,"2"],type="b",col="gold2",pch=8,cex=1.5,lwd=3,lty=3)

points(tempo1[tempo1$day==260,"dist"],tempo1[tempo1$day==260,"2"],type="b",col="blue",pch=8,cex=1.5,lwd=3,lty=3)

points(tempo1[tempo1$day==336,"dist"],tempo1[tempo1$day==336,"2"],type="b",col="green3",pch=17,cex=1.5,lwd=3)

points(tempo1[tempo1$day==518,"dist"],tempo1[tempo1$day==518,"2"],type="b",col="red",pch=18,cex=2,lwd=3,lty=5)

legend("topright",inset=c(0,0),legend=c("Dec 2013","April 2014", "Aug 2014", "Nov 2014","May 2015"),lty=1,lwd=2,col=c("black","gold2","blue","green3","red"),ncol=5,horiz=FALSE,cex=0.7,title="months")

abline(v=4,lty=2)

## residuals regressed against predicted values

tempo3<-lm(tempo1[,2]~tempo1$pred1)

summary(tempo3)

## residulas against time and space

edit(residuals(tempo))

names(residuals(tempo))

rownames(alldata)

tempo<-lm(tempo1[,2]~dist+time+dist:time,data=alldata)

summary(tempo)

plot(tempo)

anova(tempo)

##***** Turbidity Figure *****####turbidity

tiff(file="Turbidity Figure.jpg",width=10,height=14,units="in",pointsize = 12,bg ="transparent",res=800,compression="lzw")

par(mfrow=c(3,1), mar=c(3,4.5,2.5,3.5),cex=1.5,cex.axis=0.9,las=1,cex.main=1,cex.lab=0.8)

#turbidity plotted against spatial position

plot(bugenv[bugenv$day==1,"dist"],bugenv[bugenv$day==1,"turb"],type = "b",ylim=c(0,100),pch=19,xlab="Distance(km)",ylab="Turbidity (NTU)",main="a) Turbidity plotted against spatial position",lwd=3,lty=1)

points(bugenv[bugenv$day==126,"dist"],bugenv[bugenv$day==126,"turb"],type="b",col="gold2",pch=15,lwd=3,cex=1.2,lty=2)

points(bugenv[bugenv$day==260,"dist"],bugenv[bugenv$day==260,"turb"],type="b",col="blue",pch=8,cex=1.2,lwd=3,lty=3)

points(bugenv[bugenv$day==336,"dist"],bugenv[bugenv$day==336,"turb"],type="b",col="green3",pch=17,cex=1.2,lwd=3,lty=4)

points(bugenv[bugenv$day==518,"dist"],bugenv[bugenv$day==518,"turb"],type="b",col="red",pch=18,cex=1.4,lwd=3,lty=5)

legend("topleft",inset=c(0,0),legend=c("Dec 13", "April 14", "Aug 2014", "Nov 2014","May 2015"),lty=c(1,5),pch=c(19,15,8,17,18),lwd=2,col=c("black","gold2","blue","green3","red"),ncol=2,horiz=FALSE,cex=0.6,title="months")

abline(v=4,lty=2)

# Multiple regression analysis for turbidity vs time, distance and eff to check spatiotemporal variation of turbidity

# turbidity with spatiotemporal prediction

tempo<-lm(log(turb)~dist+time+dist:time,data=alldata) # Checked final model

anova(tempo)

summary(tempo)

tempo1<-cbind(cbind(exp(fitted(tempo)),residuals(tempo)),alldata)

attributes(tempo1)

names(tempo1[,1:2])<-c("fitted","residual")

tempo1[,"2"]

#turbidity with spatiotemporal model predictions

plot(tempo1$dist[tempo1$time==1],tempo1[tempo1$time==1,"turb"],type="p",pch=19,col="black",xlab="Distance (km)",ylab="Turbidity (NTU)",main="b) Turbidity with spatiotemporal prediction",ylim=c(0,100),cex=1,lwd=3,lty=1)

points(tempo1$dist[tempo1$time==1],tempo1[tempo1$time==2,"turb"],col="gold2",pch=15,cex=1.2,lwd=3,lty=2)

points(tempo1$dist[tempo1$time==1],tempo1[tempo1$time==3,"turb"],col="blue",pch=8,cex=1.2,lwd=3,lty=3)

points(tempo1$dist[tempo1$time==1],tempo1[tempo1$time==4,"turb"],col="green3",pch=17,cex=1.2,lwd=3,lty=4)

points(tempo1$dist[tempo1$time==1],tempo1[tempo1$time==5,"turb"],col="red",pch=18,cex=1.4,lwd=3,lty=5)

points(tempo1[tempo1$day==1,"dist"],tempo1[tempo1$day==1,"1"],type="l",col="black",pch=19,cex=1.2,lwd=3,lty=1)

points(tempo1[tempo1$day==126,"dist"],tempo1[tempo1$day==126,"1"],type="l",col="gold2",pch=15,cex=1.5,lwd=3,lty=2)

points(tempo1[tempo1$day==260,"dist"],tempo1[tempo1$day==260,"1"],type="l",col="blue",pch=8,cex=1.5,lwd=3,lty=3)

points(tempo1[tempo1$day==336,"dist"],tempo1[tempo1$day==336,"1"],type="l",col="green3",pch=17,cex=1.5,lwd=3,lty=4)

points(tempo1[tempo1$day==518,"dist"],tempo1[tempo1$day==518,"1"],type="l",col="red",pch=18,cex=2,lwd=3,lty=5)

legend("topleft",inset=c(0,0),legend=c("Dec 13", "April 14", "Aug 2014", "Nov 2014","May 2015"),lty=c(1,5),pch=c(19,15,8,17,18),lwd=2,col=c("black","gold2","blue","green3","red"),ncol=2,horiz=FALSE,cex=0.6,title="months")

abline(v=4,lty=2)

###turbidity linear model

#turbidity prediction with environmental variables

tempo<-lm(log(turb)~log(rain3)+log(dayflow)+log(res300)+log(rain3):log(res300),data=alldata)#checked1

tempo<-lm(log(turb)~log(rain3)+log(dayflow)+log(vel),data=alldata)#checked5

anova(tempo) ###*********###

summary(tempo) ###*********###

tempo1<-cbind(cbind(exp(fitted(tempo)),residuals(tempo)),alldata)

attributes(tempo1)

names(tempo1[,1:2])<-c("fitted","residual")

tempo1[,"2"]

plot(tempo1[tempo1$day==1,"dist"],tempo1[tempo1$day==1,"1"],type = "b",ylim=c(0,100),pch=19,col="black",xlab="Distance(km)",ylab="Turbidity (NTU)",main="c) Prediction with environmental parameters",lwd=3,lty=1)

points(tempo1[tempo1$day==126,"dist"],tempo1[tempo1$day==126,"1"],type="b",col="gold2",pch=15,lwd=3,cex=1.5,lty=2)

points(tempo1[tempo1$day==260,"dist"],tempo1[tempo1$day==260,"1"],type="b",col="blue",pch=8,cex=1.2,lwd=3,lty=3)

points(tempo1[tempo1$day==336,"dist"],tempo1[tempo1$day==336,"1"],type="b",col="green3",pch=17,cex=1.2,lwd=3,lty=4)

points(tempo1[tempo1$day==518,"dist"],tempo1[tempo1$day==518,"1"],type="b",col="red",pch=18,cex=1.4,lwd=3,lty=5)

legend("topleft",inset=c(0,0),legend=c("Dec 13", "April 14", "Aug 2014", "Nov 2014","May 2015"),lty=c(1,5),pch=c(19,15,8,17,18),lwd=2,col=c("black","gold2","blue","green3","red"),ncol=2,horiz=FALSE,cex=0.6,title="months")

abline(v=4,lty=2)

dev.off()

#

# Total organic carbon (TOC)

#check the graph (scatterplot) for toc

par(mar=c(5,5,4,2),cex=0.9)

panel.cor <- function(x, y, digits = 2, cex.cor, ...)

{

usr <- par("usr"); on.exit(par(usr))

par(usr = c(0, 1, 0, 1))

# correlation coefficient

r <- cor(x, y)

method=c("spearman")

txt <- format(c(r, 0.123456789), digits = digits)[1]

txt <- paste("r= ", txt, sep = "")

text(0.5, 0.8, txt)

# p-value calculation

p <- cor.test(x, y)$p.value

txt2 <- format(c(p, 0.123456789), digits = digits)[1]

txt2 <- paste("p= ", txt2, sep = "")

if(p<0.01) txt2 <- paste("p= ", "<0.01", sep = "")

text(0.5, 0.3, txt2)

}

pairs(~dayflow+eff+I(log(dtoc/dayflow))+temp+rain2+time+log(toc),data=alldata,upper.panel=panel.cor,pch=20,na.action = na.omit)

pairs(~dayflow+eff+I(log(dtoc/dayflow))+temp+rain2+time+log(toc),data=alldata[alldata$eff==1,],pch=20,na.action = na.omit)

unique(alldata$dist)

alldata$toc[alldata$dist==4.08]/(alldata$dtoc[alldata$dist==4.08]/alldata$dayflow[alldata$dist==4.08])

#TOC regression with other variables

tempo<-lm(toc~dayflow,data=alldata)#Adjusted R-squared: 0.1536

tempo<-lm(toc~eff,data=alldata)## Adjusted R-squared: 0.2125

tempo<-lm(toc~dtoc,data=alldata)

tempo<-lm(toc~temp,data=alldata)

tempo<-lm(toc~rain3,data=alldata)##Adjusted R-squared: 0.4314

# Multiple regression analysis for TOC vs time, distance and eff to check spatiotemporal variation of TOC

# TOC with spatiotemporal prediction

#tempo<-lm(toc~as.factor(eff)+time+dist+as.factor(eff):time+as.factor(eff):dist+as.factor(eff):time:dist,data=alldata)

#tempo<-lm(toc~as.factor(eff)+time+as.factor(eff):time+as.factor(eff):time:dist,data=alldata)# final model

tempo<-lm(log(toc)~dist+time+dist:time+eff:time+eff:dist:time+eff:I(dist^2):time,data=alldata) # final model##*********#

anova(tempo) ##***** Table 8 - appendix *****#

summary(tempo) ##***** Table 9 - appendix *****#

tempo1<-cbind(cbind(exp(fitted(tempo)),residuals(tempo)),alldata)

attributes(tempo1)

names(tempo1[,1:2])<-c("fitted","residual")

edit(tempo1)

tempo1[,"2"]

#toc with spatiotemporal predictions

plot(tempo1$dist[tempo1$time==1],tempo1[tempo1$time==1,"toc"],type="p",pch=19,col="black",xlab="Distance (km)",ylab="TOC (mg/L)",main="TOC with spatiotemporal prediction",ylim=c(0,10),cex=1,lwd=3,lty=1)

points(tempo1$dist[tempo1$time==1],tempo1[tempo1$time==2,"toc"],col="gold2",pch=15,cex=1.2,lwd=3,lty=2)

points(tempo1$dist[tempo1$time==1],tempo1[tempo1$time==3,"toc"],col="blue",pch=8,cex=1.2,lwd=3,lty=3)

points(tempo1$dist[tempo1$time==1],tempo1[tempo1$time==4,"toc"],col="green3",pch=17,cex=1.2,lwd=3,lty=4)

points(tempo1$dist[tempo1$time==1],tempo1[tempo1$time==5,"toc"],col="red",pch=18,cex=1.4,lwd=3,lty=5)

points(tempo1[tempo1$day==1,"dist"],tempo1[tempo1$day==1,"1"],type="l",col="black",pch=19,cex=1.5,lwd=3,lty=1)

points(tempo1[tempo1$day==126,"dist"],tempo1[tempo1$day==126,"1"],type="l",col="gold2",pch=15,cex=1.5,lwd=3,lty=2)

points(tempo1[tempo1$day==260,"dist"],tempo1[tempo1$day==260,"1"],type="l",col="blue",pch=8,cex=1.5,lwd=3,lty=3)

points(tempo1[tempo1$day==336,"dist"],tempo1[tempo1$day==336,"1"],type="l",col="green3",pch=17,cex=1.5,lwd=3,lty=4)

points(tempo1[tempo1$day==518,"dist"],tempo1[tempo1$day==518,"1"],type="l",col="red",pch=18,cex=2,lwd=3,lty=5)

legend("topleft",inset=c(0,0),legend=c("Dec 13", "April 14", "Aug 2014", "Nov 2014","May 2015"),lty=c(1,5),pch=c(19,15,8,17,18),lwd=2,col=c("black","gold2","blue","green3","red"),ncol=2,horiz=FALSE,cex=0.6,title="months")

abline(v=4,lty=2)

#residuals

par(mar=c(5,5,4,2),cex=0.9)

plot(tempo1[tempo1$day==1,"dist"],tempo1[tempo1$day==1,"2"],type = "b",ylim=c(-1,1),pch=19,cex=1.5,xlab="Distance(km)",ylab="residulas",main="prediction with space and time",lwd=3,lty=4)

points(tempo1[tempo1$day==126,"dist"],tempo1[tempo1$day==126,"2"],type="b",col="gold2",pch=8,cex=1.5,lwd=3,lty=3)

points(tempo1[tempo1$day==260,"dist"],tempo1[tempo1$day==260,"2"],type="b",col="blue",pch=8,cex=1.5,lwd=3,lty=3)

points(tempo1[tempo1$day==336,"dist"],tempo1[tempo1$day==336,"2"],type="b",col="green3",pch=17,cex=1.5,lwd=3)

points(tempo1[tempo1$day==518,"dist"],tempo1[tempo1$day==518,"2"],type="b",col="red",pch=18,cex=2,lwd=3,lty=5)

legend("topright",inset=c(0,0),legend=c("Dec 2013","April 2014", "Aug 2014", "Nov 2014","May 2015"),lty=1,lwd=2,col=c("black","gold2","blue","green3","red"),ncol=5,horiz=FALSE,cex=0.7,title="months")

abline(v=4,lty=2)

anova(tempo)

summary(tempo)

plot(tempo) ## checking the diagnostic plots to check the multiple regression assumptions

qqnorm(tempo$residuals)

qqline(tempo$residuals)

hist(tempo$residuals)

# Multiple regression analysis for toc with other environmental variables

## linear model for toc

#tempo<-lm(toc~time+eff:dist+eff:dist:I(dtoc/dayflow)+eff:I(dtoc/dayflow)+eff:dist:temp2,data=alldata)

#tempo<-lm(toc~time+eff:dist+eff:I(dtoc/dayflow),data=alldata)###final model

#tempo<-lm(log(toc)~temp+time+eff:I(log(dtoc/dayflow)):time,data=alldata)

#tempo<-lm(log(toc)~time+eff:I(log(dtoc/dayflow)):time+eff:dist+eff:dist:time,data=alldata)

tempo<-lm(log(toc)~rain2+dayflow+dist+time+dist:time+eff:I(log(dtoc/dayflow))+eff:I(log(dtoc/dayflow)):time+eff:time:dist+eff:I(dist^2):time,data=alldata) #Final checked model1

tempo<-lm(log(toc)~dist+time+eff:I(log(dtoc/dayflow))+eff:I(log(dtoc/dayflow)):time+eff:I(log(dtoc/dayflow)):dist,data=alldata) #Final checked model3

tempo<-lm(toc~time+eff:I(dtoc/dayflow)+eff:I(dtoc/dayflow):time+eff:I(dtoc/dayflow):dist,data=alldata)# #Final checked model3

#toc plots with spatiotemporal model predictions (including other variables)

tempo<-lm((toc)~dayflow+rain2+rain3+eff:I((dtoc/dayflow)):time+eff:I((dtoc/dayflow)):I(dist==4.08):time,data=alldata)# Final checked model5 ##*********#

anova(tempo) ##***** Table 10 - appendix *****#

summary(tempo) ##***** Table 11 - appendix *****#

#tempo<-lm(toc~dayflow+dtoc+dist+eff+rain3+eff:dtoc+eff:dtoc:dist,data=alldata)## all significant ones

#tempo<-lm(toc~dayflow+dtoc+dist+eff+eff:dist+eff:dtoc+eff:dtoc:dist+time+dtoc:time,data=alldata)# time and distance are important and it might be related to rainfall

#tempo1<-cbind(cbind((fitted(tempo)),residuals(tempo)),alldata)

tempo1<-cbind(cbind((fitted(tempo)),expm1(residuals(tempo))),alldata)

names(tempo1[,1:2])<-c("fitted","residual")

#edit(tempo1)

tempo1[,"2"]

edit(alldata)

#toc plots with spatiotemporal model predictions (including other variables)

plot(tempo1[tempo1$day==1,"dist"],tempo1[tempo1$day==1,"1"],type = "l",ylim=c(0,10),pch=19,col="black",xlab="Distance(km)",ylab="TOC (mg/L)",main="Prediction with environmental variables",lwd=3,lty=1)

points(tempo1[tempo1$day==126,"dist"],tempo1[tempo1$day==126,"1"],type="l",col="gold2",pch=15,lwd=3,cex=1.5,lty=2)

points(tempo1[tempo1$day==260,"dist"],tempo1[tempo1$day==260,"1"],type="l",col="blue",pch=8,cex=1.2,lwd=3,lty=3)

points(tempo1[tempo1$day==336,"dist"],tempo1[tempo1$day==336,"1"],type="l",col="green3",pch=17,cex=1.2,lwd=3,lty=4)

points(tempo1[tempo1$day==518,"dist"],tempo1[tempo1$day==518,"1"],type="l",col="red",pch=18,cex=1.4,lwd=3,lty=5)

legend("topleft",inset=c(0,0),legend=c("Dec 13", "April 14", "Aug 2014", "Nov 2014","May 2015"),lty=c(1,5),pch=c(19,15,8,17,18),lwd=2,col=c("black","gold2","blue","green3","red"),ncol=2,horiz=FALSE,cex=0.6,title="months")

abline(v=4,lty=2)

#residuals plotted aginst distance

par(mar=c(5,5,4,2),cex=0.9)

plot(tempo1[tempo1$day==1,"dist"],tempo1[tempo1$day==1,"2"],type = "b",ylim=c(-3,20),pch=19,cex=1.5,xlab="Distance(km)",ylab="residulas",main="prediction with space and time",lwd=3,lty=4)

points(tempo1[tempo1$day==126,"dist"],tempo1[tempo1$day==126,"2"],type="b",col="gold2",pch=8,cex=1.5,lwd=3,lty=3)

points(tempo1[tempo1$day==260,"dist"],tempo1[tempo1$day==260,"2"],type="b",col="blue",pch=8,cex=1.5,lwd=3,lty=3)

points(tempo1[tempo1$day==336,"dist"],tempo1[tempo1$day==336,"2"],type="b",col="green3",pch=17,cex=1.5,lwd=3)

points(tempo1[tempo1$day==518,"dist"],tempo1[tempo1$day==518,"2"],type="b",col="red",pch=18,cex=2,lwd=3,lty=5)

legend("topright",inset=c(0,0),legend=c("Dec 2013","April 2014", "Aug 2014", "Nov 2014","May 2015"),lty=1,lwd=2,col=c("black","gold2","blue","green3","red"),ncol=5,horiz=FALSE,cex=0.7,title="months")

abline(v=4,lty=2)

summary(tempo)

anova(tempo)

avPlots(tempo)

plot(tempo)

tempo.check<-lm(residuals(tempo)~dist+time+dist:time+eff:time+eff:dist:time,data=alldata)

#tempo.check<-lm(residuals(tempo)~rain2,data=alldata)

summary(tempo.check)

anova(tempo.check) # No clear evidence of spatiotemporal patters in residuals

xyplot(fitted(tempo)~dist, groups=day,data=tempo1,type="l",auto.key=TRUE)

xyplot(residuals(tempo)~dist, groups=day,data=tempo1,type="l",auto.key=TRUE)

tempo3<-lm(tempo1[,2]~tempo1$pred1)

summary(tempo3)

## residulas against time and space

edit(residuals(tempo))

names(residuals(tempo))

rownames(alldata)

tempo<-lm(tempo1[,2]~time,data=alldata)

summary(tempo)

plot(tempo)

anova(tempo)

##***** Figure 7 *****#######TOC plot as in Figure 7

tiff(file="Figure 7.jpg",width=10,height=14,units="in",pointsize = 12,bg ="transparent",res=800,compression="lzw")

par(mfrow=c(3,1), mar=c(3,4.5,2.5,3.5),cex=1.5,cex.axis=0.9,las=1,cex.main=1,cex.lab=0.8)

#toc plotted against spatial position

plot(bugenv[bugenv$day==1,"dist"],bugenv[bugenv$day==1,"toc"],type = "b",ylim=c(0,10),pch=19,xlab="Distance(km)",ylab="TOC (mg/L)",main="a) TOC plotted against spatial position",lwd=3,lty=1)

points(bugenv[bugenv$day==126,"dist"],bugenv[bugenv$day==126,"toc"],type="b",col="gold2",pch=15,lwd=3,cex=1.2,lty=2)

points(bugenv[bugenv$day==260,"dist"],bugenv[bugenv$day==260,"toc"],type="b",col="blue",pch=8,cex=1.2,lwd=3,lty=3)

points(bugenv[bugenv$day==336,"dist"],bugenv[bugenv$day==336,"toc"],type="b",col="green3",pch=17,cex=1.2,lwd=3,lty=4)

points(bugenv[bugenv$day==518,"dist"],bugenv[bugenv$day==518,"toc"],type="b",col="red",pch=18,cex=1.4,lwd=3,lty=5)

legend("topleft",inset=c(0,0),legend=c("Dec 13", "April 14", "Aug 2014", "Nov 2014","May 2015"),lty=c(1,5),pch=c(19,15,8,17,18),lwd=2,col=c("black","gold2","blue","green3","red"),ncol=2,horiz=FALSE,cex=0.6,title="months")

abline(v=4,lty=2)

# TOC with spatiotemporal prediction

tempo<-lm(log(toc)~dist+time+dist:time+eff:time+eff:dist:time+eff:I(dist^2):time,data=alldata) # Checked final model

tempo1<-cbind(cbind(exp(fitted(tempo)),residuals(tempo)),alldata)

attributes(tempo1)

names(tempo1[,1:2])<-c("fitted","residual")

tempo1[,"2"]

plot(tempo1$dist[tempo1$time==1],tempo1[tempo1$time==1,"toc"],type="p",pch=19,col="black",xlab="Distance (km)",ylab="TOC (mg/L)",main="b) TOC with spatiotemporal prediction",ylim=c(0,10),cex=1,lwd=3,lty=1)

points(tempo1$dist[tempo1$time==1],tempo1[tempo1$time==2,"toc"],col="gold2",pch=15,cex=1.2,lwd=3,lty=2)

points(tempo1$dist[tempo1$time==1],tempo1[tempo1$time==3,"toc"],col="blue",pch=8,cex=1.2,lwd=3,lty=3)

points(tempo1$dist[tempo1$time==1],tempo1[tempo1$time==4,"toc"],col="green3",pch=17,cex=1.2,lwd=3,lty=4)

points(tempo1$dist[tempo1$time==1],tempo1[tempo1$time==5,"toc"],col="red",pch=18,cex=1.4,lwd=3,lty=5)

points(tempo1[tempo1$day==1,"dist"],tempo1[tempo1$day==1,"1"],type="l",col="black",pch=19,cex=1.5,lwd=3,lty=1)

points(tempo1[tempo1$day==126,"dist"],tempo1[tempo1$day==126,"1"],type="l",col="gold2",pch=15,cex=1.5,lwd=3,lty=2)

points(tempo1[tempo1$day==260,"dist"],tempo1[tempo1$day==260,"1"],type="l",col="blue",pch=8,cex=1.5,lwd=3,lty=3)

points(tempo1[tempo1$day==336,"dist"],tempo1[tempo1$day==336,"1"],type="l",col="green3",pch=17,cex=1.5,lwd=3,lty=4)

points(tempo1[tempo1$day==518,"dist"],tempo1[tempo1$day==518,"1"],type="l",col="red",pch=18,cex=2,lwd=3,lty=5)

legend("topleft",inset=c(0,0),legend=c("Dec 13", "April 14", "Aug 2014", "Nov 2014","May 2015"),lty=c(1,5),pch=c(19,15,8,17,18),lwd=2,col=c("black","gold2","blue","green3","red"),ncol=2,horiz=FALSE,cex=0.6,title="months")

abline(v=4,lty=2)

#TOC plots with spatiotemporal model predictions (including other variables)

tempo<-lm(toc~time+eff:I(dtoc/dayflow)+eff:I(dtoc/dayflow):time+eff:I(dtoc/dayflow):dist,data=alldata)# checked final model

tempo<-lm(log(toc)~dayflow+rain2+rain3+eff:I(log(dtoc/dayflow)):time+eff:I(log(dtoc/dayflow)):I(dist==4.08):time,data=alldata)# checked final model

tempo<-lm(log(toc)~time+eff:I(dtoc/dayflow)+eff:I(dtoc/dayflow):time+eff:I(dtoc/dayflow):dist,data=alldata)# checked final model3

tempo<-lm((toc)~dayflow+rain2+rain3+eff:I((dtoc/dayflow)):time+eff:I((dtoc/dayflow)):I(dist==4.08):time,data=alldata)# checked final model5

anova(tempo)

summary(tempo)

tempo1<-cbind(cbind((fitted(tempo)),expm1(residuals(tempo))),alldata)

names(tempo1[,1:2])<-c("fitted","residual")

tempo1[,"2"]

plot(tempo1[tempo1$day==1,"dist"],tempo1[tempo1$day==1,"1"],type = "b",ylim=c(0,10),pch=19,col="black",xlab="Distance(km)",ylab="TOC (mg/L)",main="c) Prediction with environmental variables",lwd=3,lty=1)

points(tempo1[tempo1$day==126,"dist"],tempo1[tempo1$day==126,"1"],type="b",col="gold2",pch=15,lwd=3,cex=1.5,lty=2)

points(tempo1[tempo1$day==260,"dist"],tempo1[tempo1$day==260,"1"],type="b",col="blue",pch=8,cex=1.2,lwd=3,lty=3)

points(tempo1[tempo1$day==336,"dist"],tempo1[tempo1$day==336,"1"],type="b",col="green3",pch=17,cex=1.2,lwd=3,lty=4)

points(tempo1[tempo1$day==518,"dist"],tempo1[tempo1$day==518,"1"],type="b",col="red",pch=18,cex=1.4,lwd=3,lty=5)

legend("topleft",inset=c(0,0),legend=c("Dec 13", "April 14", "Aug 2014", "Nov 2014","May 2015"),lty=c(1,5),pch=c(19,15,8,17,18),lwd=2,col=c("black","gold2","blue","green3","red"),ncol=2,horiz=FALSE,cex=0.6,title="months")

abline(v=4,lty=2)

dev.off()

#

# Conductivity (cond)

#check the graph (scatterplot) for cond

par(mar=c(5,5,4,2),cex=0.9)

panel.cor <- function(x, y, digits = 2, cex.cor, ...)

{

usr <- par("usr"); on.exit(par(usr))

par(usr = c(0, 1, 0, 1))

# correlation coefficient

r <- cor(x, y)

method=c("spearman")

txt <- format(c(r, 0.123456789), digits = digits)[1]

txt <- paste("r= ", txt, sep = "")

text(0.5, 0.8, txt)

# p-value calculation

p <- cor.test(x, y)$p.value

txt2 <- format(c(p, 0.123456789), digits = digits)[1]

txt2 <- paste("p= ", txt2, sep = "")

if(p<0.01) txt2 <- paste("p= ", "<0.01", sep = "")

text(0.5, 0.3, txt2)

}

pairs(~dcond+eff+dayflow+dist+temp+cond,data=alldata,upper.panel=panel.cor,pch=20,na.action = na.omit)

##Conductivity regression with other environmental variables

tempo<-lm(cond~eff,data=alldata)##Adjusted R-squared: 0.2441

tempo<-lm(cond~dcond,data=alldata)

tempo<-lm(cond~dayflow,data=alldata)##Adjusted R-squared: 0.279

tempo<-lm(cond~dist,data=alldata) ##Adjusted R-squared: 0.09122

tempo<-lm(cond~temp,data=alldata) ##Adjusted R-squared: 0.1798

#conductivity plotted against spatial position

plot(bugenv[bugenv$day==1,"dist"],bugenv[bugenv$day==1,"cond"],type = "b",ylim=c(0,700),pch=19,xlab="Distance(km)",ylab="Conductivity (mg/L)",main="Conductivity plotted against spatial position",lwd=3,lty=1)

points(bugenv[bugenv$day==126,"dist"],bugenv[bugenv$day==126,"cond"],type="b",col="gold2",pch=15,lwd=3,cex=1.2,lty=2)

points(bugenv[bugenv$day==260,"dist"],bugenv[bugenv$day==260,"cond"],type="b",col="blue",pch=8,cex=1.2,lwd=3,lty=3)

points(bugenv[bugenv$day==336,"dist"],bugenv[bugenv$day==336,"cond"],type="b",col="green3",pch=17,cex=1.2,lwd=3,lty=4)

points(bugenv[bugenv$day==518,"dist"],bugenv[bugenv$day==518,"cond"],type="b",col="red",pch=18,cex=1.4,lwd=3,lty=5)

legend("topleft",inset=c(0,0),legend=c("Dec 13", "April 14", "Aug 2014", "Nov 2014","May 2015"),lty=c(1,5),pch=c(19,15,8,17,18),lwd=2,col=c("black","gold2","blue","green3","red"),ncol=2,horiz=FALSE,cex=0.6,title="months")

abline(v=4,lty=2)

# Multiple regression analysis for conductivity as a function of time, distance and eff to check spatiotemporal variation of conductivity

#tempo<-lm(cond~as.factor(eff)+dist+time+as.factor(eff):time+as.factor(eff):dist+as.factor(eff):dist:time,data=alldata)

tempo<-lm(log(cond)~time+eff:time+eff:dist:time+eff:I(dist^2):time,data=alldata[complete.cases(alldata[,c("cond")]),]) # Checked final model ##*********##

anova(tempo) ##***** Table 12 - appendix *****##

summary(tempo) ##***** Table 13 - appendix *****##

tempo1<-cbind(cbind(exp(fitted(tempo)),residuals(tempo)),alldata[complete.cases(alldata[,c("cond")]),])

names(tempo1[,1:2])<-c("fitted","residual")

tempo1[,"2"]

# conductivity with spatiotemporal prediction

plot(tempo1$dist[tempo1$time==2],tempo1[tempo1$time==2,"cond"],type="p",pch=15,col="gold2",xlab="Distance (km)",ylab="Conductivity (mg/L)",main="Conductivity with spatiotemporal prediction",ylim=c(0,700),cex=1,lwd=3,lty=2)

#points(tempo1$dist[tempo1$time==1],tempo1[tempo1$time==2,"cond"],col="gold2",pch=15,cex=1.5,lwd=3,lty=2)

points(tempo1$dist[tempo1$time==2],tempo1[tempo1$time==3,"cond"],col="blue",pch=8,cex=1.2,lwd=3,lty=3)

points(tempo1$dist[tempo1$time==2],tempo1[tempo1$time==4,"cond"],col="green3",pch=17,cex=1.2,lwd=3,lty=4)

points(tempo1$dist[tempo1$time==2],tempo1[tempo1$time==5,"cond"],col="red",pch=18,cex=1.4,lwd=3,lty=5)

points(tempo1[tempo1$day==126,"dist"],tempo1[tempo1$day==126,"1"],type="l",col="gold2",pch=15,cex=1.5,lwd=3,lty=2)

#points(tempo1[tempo1$day==126,"dist"],tempo1[tempo1$day==126,"1"],type="l",col="gold2",pch=15,cex=1.5,lwd=3,lty=2)

points(tempo1[tempo1$day==260,"dist"],tempo1[tempo1$day==260,"1"],type="l",col="blue",pch=8,cex=1.5,lwd=3,lty=3)

points(tempo1[tempo1$day==336,"dist"],tempo1[tempo1$day==336,"1"],type="l",col="green3",pch=17,cex=1.5,lwd=3,lty=4)

points(tempo1[tempo1$day==518,"dist"],tempo1[tempo1$day==518,"1"],type="l",col="red",pch=18,cex=2,lwd=3,lty=5)

legend("topleft",inset=c(0,0),legend=c("Dec 13", "April 14", "Aug 2014", "Nov 2014","May 2015"),lty=c(1,5),pch=c(19,15,8,17,18),lwd=2,col=c("black","gold2","blue","green3","red"),ncol=2,horiz=FALSE,cex=0.6,title="months")

abline(v=4,lty=2)

summary(tempo)

anova(tempo)

plot(tempo) ## checking the diagnostic plots to check the multiple regression assumptions

qqnorm(tempo$residuals)

qqline(tempo$residuals)

hist(tempo$residuals)

# Multiple regression analysis for cond and factors affecting it to check which variables explain spatiotemporal variation in cond

alldata$time<-as.factor(alldata$time)

#model based on variables in causal diagram

tempo<-lm(cond~eff:dist+eff:dist:I(dcond/dayflow)+eff:I(dcond/dayflow)+eff:temp2:dist,data=alldata[complete.cases(alldata[,c("cond")])])

tempo<-lm(cond~eff:dist+eff:dist:I(dcond/dayflow)+eff:I(dcond/dayflow),data=alldata[complete.cases(alldata[,c("cond")])])

tempo<-lm(log(cond)~dayflow+eff:I(dcond*dflow/dayflow)+eff:I(dcond*dflow/dayflow):time+eff:dist:time+eff:I(dist^2):time,data=alldata[complete.cases(alldata[,c("cond")]),])##final Checked model

tempo<-lm(log(cond)~dayflow+eff:I(dcond*dflow/dayflow)+eff:I(dcond*dflow/dayflow):time,data=alldata[complete.cases(alldata[,c("cond")]),])## final Checked model2

tempo<-glm(log(cond)~dayflow+toc+eff:I(log(dcond*dflow/dayflow))+eff:I(log(dcond*dflow/dayflow)):time,data=alldata, family=Gamma(link="log"))## final Checked model3

tempo<-lm(log(cond)~dayflow+toc+eff:I(log(dcond*dflow/dayflow))+eff:I(log(dcond*dflow/dayflow)):time,data=alldata[complete.cases(alldata[,c("cond")]),])## final Checked model3

tempo<-lm(log(cond)~dayflow+tp+alk+eff:I(log(dcond*dflow/dayflow))+eff:I(log(dcond*dflow/dayflow)):time,data=alldata[complete.cases(alldata[,c("cond")]),])## final Checked model5

tempo<-lm(log(cond)~tp+alk,data=alldata[complete.cases(alldata[,c("cond")]),])##model5

tempo<-lm(log(cond)~tp+alk+time+dist:time:eff,data=alldata[complete.cases(alldata[,c("cond")]),])##final model5

anova(tempo) ##***** Table 14 - appendix *****##

summary(tempo) ##***** Table 15 - appendix *****##

anova(tempo)

summary(tempo)

tempo1<-cbind(cbind(exp(fitted(tempo)),exp(residuals(tempo))),alldata[complete.cases(alldata[,c("cond")]),])

names(tempo1[,1:2])<-c("fitted","residual")

tempo1[,"2"]

plot(tempo1[tempo1$day==126,"dist"],tempo1[tempo1$day==126,"1"],type = "l",ylim=c(0,700),pch=15,col="gold2",xlab="Distance(km)",ylab="Conductivity (mg/L)",main="Prediction with environmental variables",lwd=3,lty=2)

#points(tempo1[tempo1$day==126,"dist"],tempo1[tempo1$day==126,"1"],type="l",col="gold2",pch=15,lwd=3,cex=1.5,lty=2)

points(tempo1[tempo1$day==260,"dist"],tempo1[tempo1$day==260,"1"],type="l",col="blue",pch=8,cex=1.2,lwd=3,lty=3)

points(tempo1[tempo1$day==336,"dist"],tempo1[tempo1$day==336,"1"],type="l",col="green3",pch=17,cex=1.2,lwd=3,lty=4)

points(tempo1[tempo1$day==518,"dist"],tempo1[tempo1$day==518,"1"],type="l",col="red",pch=18,cex=1.4,lwd=3,lty=5)

legend("topleft",inset=c(0,0),legend=c("Dec 13", "April 14", "Aug 2014", "Nov 2014","May 2015"),lty=c(1,5),pch=c(19,15,8,17,18),lwd=2,col=c("black","gold2","blue","green3","red"),ncol=2,horiz=FALSE,cex=0.6,title="months")

abline(v=4,lty=2)

summary(tempo)

anova(tempo)

plot(tempo)

#Partial regression plots

avPlots(tempo)

#residuals plotted aginst distance

par(mar=c(5,5,4,2),cex=0.9)

plot(tempo1[tempo1$day==126,"dist"],tempo1[tempo1$day==126,"2"],type = "b",ylim=c(0.5,1.5),pch=19,cex=1.5,xlab="Distance(km)",ylab="residulas",main="prediction with space and time",lwd=3,lty=4)

#points(tempo1[tempo1$day==126,"dist"],tempo1[tempo1$day==126,"2"],type="b",col="gold2",pch=8,cex=1.5,lwd=3,lty=3)

points(tempo1[tempo1$day==260,"dist"],tempo1[tempo1$day==260,"2"],type="b",col="blue",pch=8,cex=1.5,lwd=3,lty=3)

points(tempo1[tempo1$day==336,"dist"],tempo1[tempo1$day==336,"2"],type="b",col="green3",pch=17,cex=1.5,lwd=3)

points(tempo1[tempo1$day==518,"dist"],tempo1[tempo1$day==518,"2"],type="b",col="red",pch=18,cex=2,lwd=3,lty=5)

legend("topright",inset=c(0,0),legend=c("Dec 2013","April 2014", "Aug 2014", "Nov 2014","May 2015"),lty=1,lwd=2,col=c("black","gold2","blue","green3","red"),ncol=5,horiz=FALSE,cex=0.7,title="months")

abline(v=4,lty=2)

tempo3<-lm(tempo1[,2]~tempo1$pred1)#checking the assumptions by regressing residuals against predicted values

summary(tempo3)

## residulas modelled as a function of time and space

tempo.check<-lm(residuals(tempo)~time+eff:time+eff:dist:time+eff:I(dist^2):time,data=alldata[complete.cases(alldata[,c("cond")]),])

#tempo.check<-lm(residuals(tempo)~rain2,data=alldata)

summary(tempo.check)

anova(tempo.check) # No clear evidence of spatiotemporal patters in residuals

# some more graphs

xyplot(residuals(tempo)~dist,group=time,data=alldata[complete.cases(alldata[,c("cond")]),],type="b",pch=19,lty=2,col=c("black","gold2","blue","green3","red"))

a<-xyplot(cond~dist,groups=time,data=alldata[complete.cases(alldata[,c("cond")]),],type="b",pch=19,lty=2,col=c("gold2","blue","green3","red"))

b<-xyplot(exp(fitted(tempo))~dist,group=time,data=alldata[complete.cases(alldata[,c("cond")]),],type="l",lwd=2,col=c("gold2","blue","green3","red"))

c<-xyplot(toc~dist,data=alldata,panel = function(x,y) {panel.abline(v=4,lty=2)})

a+as.layer(b)+as.layer(c)

legend("topleft",inset=c(0,0),legend=c("April 14", "Aug 2014", "Nov 2014","May 2015"),lty=c(1,5),pch=c(19,15,8,17,18),lwd=2,col=c("gold2","blue","green3","red"),ncol=2,horiz=FALSE,cex=0.6,title="months")

# Calculate and plot the expected increase in conductivity to assess whether mixing explains pattern

cond.upstream<-aggregate(cond~time,data=alldata[complete.cases(alldata[,c("cond")]),][alldata$eff==0,],mean)

cond.discharge<-unique(with(alldata[complete.cases(alldata[,c("cond")]),],I(dcond*dflow/dayflow)))

cond.downstream<-cond.upstream$cond+cond.discharge

unique(alldata$dcond)

a<-xyplot(cond~dist,groups=time,data=alldata[complete.cases(alldata[,c("cond")]),],type="b",pch=19,lty=2,col=c("gold2","blue","green3","red"))

b<-xyplot(exp(fitted(tempo))~dist,group=time,data=alldata[complete.cases(alldata[,c("cond")]),],type="l",lwd=2,col=c("gold2","blue","green3","red"))

c<-xyplot(toc~dist,data=alldata,panel = function(x,y) {panel.abline(v=4,lty=2)})

d<-d<-xyplot(cond~dist,data=alldata[complete.cases(alldata[,c("cond")]),],panel = function(x,y) {panel.abline(h=cond.downstream,lty=2,lwd=2,col.line=c("gold2","blue","green3","red")) })

a+as.layer(b)+as.layer(c)+as.layer(d)

legend("topleft",inset=c(0,0),legend=c("April 14", "Aug 2014", "Nov 2014","May 2015"),lty=c(1,5),pch=c(19,15,8,17,18),lwd=2,col=c("gold2","blue","green3","red"),ncol=2,horiz=FALSE,cex=0.6,title="months")

# The conductivity recorded at downstream sites doesn't seem to match the expected increase, which suggests there are still inacuraies in mass discharge loads.

###### Conductivity plot as in Figure 8

##***** Figure 8 *****##

tiff(file="Figure 8.jpg",width=10,height=14,units="in",pointsize = 12,bg ="transparent",res=800,compression="lzw")

par(mfrow=c(3,1), mar=c(3,4.5,2.5,3.5),cex=1.5,cex.axis=0.9,las=1,cex.main=1,cex.lab=0.8)

#conductivity plotted against spatial position

plot(bugenv[bugenv$day==1,"dist"],bugenv[bugenv$day==1,"cond"],type = "b",ylim=c(0,700),pch=19,xlab="Distance(km)",ylab="Conductivity (mg/L)",main="a) Conductivity plotted against spatial position",lwd=3,lty=1)

points(bugenv[bugenv$day==126,"dist"],bugenv[bugenv$day==126,"cond"],type="b",col="gold2",pch=15,lwd=3,cex=1.2,lty=2)

points(bugenv[bugenv$day==260,"dist"],bugenv[bugenv$day==260,"cond"],type="b",col="blue",pch=8,cex=1.2,lwd=3,lty=3)

points(bugenv[bugenv$day==336,"dist"],bugenv[bugenv$day==336,"cond"],type="b",col="green3",pch=17,cex=1.2,lwd=3,lty=4)

points(bugenv[bugenv$day==518,"dist"],bugenv[bugenv$day==518,"cond"],type="b",col="red",pch=18,cex=1.4,lwd=3,lty=5)

legend("topleft",inset=c(0,0),legend=c("Dec 13", "April 14", "Aug 2014", "Nov 2014","May 2015"),lty=c(1,5),pch=c(19,15,8,17,18),lwd=2,col=c("black","gold2","blue","green3","red"),ncol=2,horiz=FALSE,cex=0.6,title="months")

abline(v=4,lty=2)

# conductivity with spatiotemporal prediction

tempo<-lm(log(cond)~time+eff:time+eff:dist:time+eff:I(dist^2):time,data=alldata[complete.cases(alldata[,c("cond")]),]) # final Checked model

tempo1<-cbind(cbind(exp(fitted(tempo)),residuals(tempo)),alldata[complete.cases(alldata[,c("cond")]),])

names(tempo1[,1:2])<-c("fitted","residual")

tempo1[,"2"]

plot(tempo1$dist[tempo1$time==2],tempo1[tempo1$time==2,"cond"],type="p",pch=15,col="gold2",xlab="Distance (km)",ylab="Conductivity (mg/L)",main="b) Conductivity with spatiotemporal prediction",ylim=c(0,700),cex=1,lwd=3,lty=2)

#points(tempo1$dist[tempo1$time==1],tempo1[tempo1$time==2,"cond"],col="gold2",pch=15,cex=1.5,lwd=3,lty=2)

points(tempo1$dist[tempo1$time==2],tempo1[tempo1$time==3,"cond"],col="blue",pch=8,cex=1.2,lwd=3,lty=3)

points(tempo1$dist[tempo1$time==2],tempo1[tempo1$time==4,"cond"],col="green3",pch=17,cex=1.2,lwd=3,lty=4)

points(tempo1$dist[tempo1$time==2],tempo1[tempo1$time==5,"cond"],col="red",pch=18,cex=1.4,lwd=3,lty=5)

points(tempo1[tempo1$day==126,"dist"],tempo1[tempo1$day==126,"1"],type="l",col="gold2",pch=15,cex=1.5,lwd=3,lty=2)

#points(tempo1[tempo1$day==126,"dist"],tempo1[tempo1$day==126,"1"],type="l",col="gold2",pch=15,cex=1.5,lwd=3,lty=2)

points(tempo1[tempo1$day==260,"dist"],tempo1[tempo1$day==260,"1"],type="l",col="blue",pch=8,cex=1.5,lwd=3,lty=3)

points(tempo1[tempo1$day==336,"dist"],tempo1[tempo1$day==336,"1"],type="l",col="green3",pch=17,cex=1.5,lwd=3,lty=4)

points(tempo1[tempo1$day==518,"dist"],tempo1[tempo1$day==518,"1"],type="l",col="red",pch=18,cex=2,lwd=3,lty=5)

legend("topleft",inset=c(0,0),legend=c("Dec 13", "April 14", "Aug 2014", "Nov 2014","May 2015"),lty=c(1,5),pch=c(19,15,8,17,18),lwd=2,col=c("black","gold2","blue","green3","red"),ncol=2,horiz=FALSE,cex=0.6,title="months")

abline(v=4,lty=2)

#conductivity prediction with other environmental variables

tempo<-lm(log(cond)~dayflow+tp+alk+eff:I(log(dcond*dflow/dayflow))+eff:I(log(dcond*dflow/dayflow)):time,data=alldata[complete.cases(alldata[,c("cond")]),])##final model3

tempo<-lm(log(cond)~tp+alk,data=alldata[complete.cases(alldata[,c("cond")]),])##final model5

tempo1<-cbind(cbind(exp(fitted(tempo)),exp(residuals(tempo))),alldata[complete.cases(alldata[,c("cond")]),])

names(tempo1[,1:2])<-c("fitted","residual")

tempo1[,"2"]

plot(tempo1[tempo1$day==126,"dist"],tempo1[tempo1$day==126,"1"],type = "b",ylim=c(0,700),pch=15,col="gold2",xlab="Distance(km)",ylab="Conductivity (mg/L)",main="c) Prediction with environmental variables",lwd=3,lty=2)

#points(tempo1[tempo1$day==126,"dist"],tempo1[tempo1$day==126,"1"],type="b",col="gold2",pch=15,lwd=3,cex=1.5,lty=2)

points(tempo1[tempo1$day==260,"dist"],tempo1[tempo1$day==260,"1"],type="b",col="blue",pch=8,cex=1.2,lwd=3,lty=3)

points(tempo1[tempo1$day==336,"dist"],tempo1[tempo1$day==336,"1"],type="b",col="green3",pch=17,cex=1.2,lwd=3,lty=4)

points(tempo1[tempo1$day==518,"dist"],tempo1[tempo1$day==518,"1"],type="b",col="red",pch=18,cex=1.4,lwd=3,lty=5)

legend("topleft",inset=c(0,0),legend=c("Dec 13", "April 14", "Aug 2014", "Nov 2014","May 2015"),lty=c(1,5),pch=c(19,15,8,17,18),lwd=2,col=c("black","gold2","blue","green3","red"),ncol=2,horiz=FALSE,cex=0.6,title="months")

abline(v=4,lty=2)

dev.off()

#

# water temperature

par(mar=c(5,5,4,2),cex=0.9)

panel.cor <- function(x, y, digits = 2, cex.cor, ...)

{

usr <- par("usr"); on.exit(par(usr))

par(usr = c(0, 1, 0, 1))

# correlation coefficient

r <- cor(x, y)

method=c("spearman")

txt <- format(c(r, 0.123456789), digits = digits)[1]

txt <- paste("r= ", txt, sep = "")

text(0.5, 0.8, txt)

# p-value calculation

p <- cor.test(x, y)$p.value

txt2 <- format(c(p, 0.123456789), digits = digits)[1]

txt2 <- paste("p= ", txt2, sep = "")

if(p<0.01) txt2 <- paste("p= ", "<0.01", sep = "")

text(0.5, 0.3, txt2)

}

pairs(~eff+airtemp+dtemp+solar+canop+res300+rain3+dayflow+depth+temp,data=alldata,upper.panel=panel.cor,pch=20,na.action = na.omit)

##water temperatute regression with other environmental variables

tempo<-lm(temp~eff,data=alldata)

tempo<-lm(temp~airtemp,data=alldata) ##Adjusted R-squared: 0.7661

tempo<-lm(temp~dtemp,data=alldata) ##Adjusted R-squared: 0.8383

tempo<-lm(temp~canop,data=alldata)

tempo<-lm(temp~solar,data=alldata)##Adjusted R-squared: 0.7099

summary(tempo)

# water temperatute plotted against spatial position

plot(alldata[alldata$day==1,"dist"],alldata[alldata$day==1,"temp"],type = "b",ylim=c(0,30),pch=19,xlab="Distance(km)",ylab="Temperature",main="Temperature with spatiotemporal prediction",lwd=3,lty=1)

points(alldata[alldata$day==126,"dist"],alldata[alldata$day==126,"temp"],type="b",col="gold2",pch=15,lwd=3,cex=1.2,lty=2)

points(alldata[alldata$day==260,"dist"],alldata[alldata$day==260,"temp"],type="b",col="blue",pch=8,cex=1.2,lwd=3,lty=3)

points(alldata[alldata$day==336,"dist"],alldata[alldata$day==336,"temp"],type="b",col="green3",pch=17,cex=1.2,lwd=3,lty=4)

points(alldata[alldata$day==518,"dist"],alldata[alldata$day==518,"temp"],type="b",col="red",pch=18,cex=1.4,lwd=3,lty=5)

legend("topright",inset=c(0,0),legend=c("Dec 13", "April 14", "Aug 2014", "Nov 2014","May 2015"),lty=c(1,5),pch=c(19,15,8,17,18),lwd=2,col=c("black","gold2","blue","green3","red"),ncol=2,horiz=FALSE,cex=0.6,title="months")

abline(v=4,lty=2)

# Multiple regression analysis for water temperature vs time, distance and eff to check spatiotemporal variation of water temperature

tempo<-lm(temp~dist+time+dist:time,data=alldata) # Checked modle

anova(tempo) ##***** Table 16 - appendix *****###

summary(tempo) ##***** Table 17 - appendix *****###

plot(tempo) ## checking the diagnostic plots to check the multiple regresstion assumptions

qqnorm(tempo$residuals)

qqline(tempo$residuals)

hist(tempo$residuals)

#The plot of water temperature with spatiotemporal model predictions

tempo1<-cbind(cbind((fitted(tempo)),residuals(tempo)),alldata)

attributes(tempo1)

names(tempo1[,1:2])<-c("fitted","residual")

tempo1[,"2"]

plot(tempo1$dist[tempo1$time==1],tempo1[tempo1$time==1,"temp"],type="p",pch=19,col="black",xlab="Distance (km)",ylab="Temperature",main="Temperature with spatiotemporal prediction",ylim=c(0,30),cex=1,lwd=3,lty=1)

points(tempo1$dist[tempo1$time==1],tempo1[tempo1$time==2,"temp"],col="gold2",pch=15,cex=1.2,lwd=3,lty=2)

points(tempo1$dist[tempo1$time==1],tempo1[tempo1$time==3,"temp"],col="blue",pch=8,cex=1.2,lwd=3,lty=3)

points(tempo1$dist[tempo1$time==1],tempo1[tempo1$time==4,"temp"],col="green3",pch=17,cex=1.2,lwd=3,lty=4)

points(tempo1$dist[tempo1$time==1],tempo1[tempo1$time==5,"temp"],col="red",pch=18,cex=1.4,lwd=3,lty=5)

points(tempo1[tempo1$day==1,"dist"],tempo1[tempo1$day==1,"1"],type="l",col="black",pch=19,cex=1.2,lwd=3,lty=1)

points(tempo1[tempo1$day==126,"dist"],tempo1[tempo1$day==126,"1"],type="l",col="gold2",pch=15,cex=1.5,lwd=3,lty=2)

points(tempo1[tempo1$day==260,"dist"],tempo1[tempo1$day==260,"1"],type="l",col="blue",pch=8,cex=1.5,lwd=3,lty=3)

points(tempo1[tempo1$day==336,"dist"],tempo1[tempo1$day==336,"1"],type="l",col="green3",pch=17,cex=1.5,lwd=3,lty=4)

points(tempo1[tempo1$day==518,"dist"],tempo1[tempo1$day==518,"1"],type="l",col="red",pch=18,cex=2,lwd=3,lty=5)

legend("topright",inset=c(0,0),legend=c("Dec 13", "April 14", "Aug 2014", "Nov 2014","May 2015"),lty=c(1,5),pch=c(19,15,8,17,18),lwd=2,col=c("black","gold2","blue","green3","red"),ncol=2,horiz=FALSE,cex=0.6,title="months")

abline(v=4,lty=2)

# water temperature linear model

## multiple regression

#tempo<-lm(temp~solar+dtemp+airtemp+canop+eff,data=alldata)

#tempo<-lm(temp~solar+dtemp+airtemp+canop+as.factor(eff)+dist+time+as.factor(eff):time+as.factor(eff):dist+as.factor(eff):dist:time,data=alldata)##final model

#dtemp cannot affect temp upstream of the disharge. Also, there is no evidence in the spatiotemporal model that dtemp affects temp.

#tempo<-lm(temp~solar+rain3+depth+airtemp+airtemp:depth,data=alldata) #

#tempo<-lm(temp~rain3*airtemp*dayflow*canop*res300,data=alldata) #

#tempo<-lm(temp~canop+rain3+airtemp+dayflow+res300+dayflow:res300+rain3:res300+dayflow:res300:rain3,data=alldata) #

#tempo<-lm(temp~canop+rain3+airtemp+dayflow+res300,data=alldata) #

tempo<-lm(temp~rain3+airtemp+dayflow+res300+dayflow:res300+rain3:res300+dayflow:res300:rain3,data=alldata) ##Checked model

anova(tempo) ##***** Table 18 - appendix *****###

summary(tempo) ##***** Table 19 - appendix *****###

plot(tempo)

sqrt(vif(tempo))

tempo.check<-lm(residuals(tempo)~dist+time+dist:time,data=alldata)

summary(tempo.check)

anova(tempo.check) # Still some evidence of spatiotemporal patters in residuals

avPlots(tempo)

# water temperature prediction with environmental variables

tempo1<-cbind(cbind((fitted(tempo)),residuals(tempo)),alldata)

attributes(tempo1)

names(tempo1[,1:2])<-c("fitted","residual")

tempo1[,"2"]

#Temp plots with spatiotemporal model predictions (including other variables)

plot(tempo1[tempo1$day==1,"dist"],tempo1[tempo1$day==1,"1"],type = "l",ylim=c(0,30),pch=19,col="black",xlab="Distance(km)",ylab="Temperature",main="Temperature with other variables",lwd=3,lty=1)

points(tempo1[tempo1$day==126,"dist"],tempo1[tempo1$day==126,"1"],type="l",col="gold2",pch=15,lwd=3,cex=1.5,lty=2)

points(tempo1[tempo1$day==260,"dist"],tempo1[tempo1$day==260,"1"],type="l",col="blue",pch=8,cex=1.2,lwd=3,lty=3)

points(tempo1[tempo1$day==336,"dist"],tempo1[tempo1$day==336,"1"],type="l",col="green3",pch=17,cex=1.2,lwd=3,lty=4)

points(tempo1[tempo1$day==518,"dist"],tempo1[tempo1$day==518,"1"],type="l",col="red",pch=18,cex=1.4,lwd=3,lty=5)

legend("topright",inset=c(0,0),legend=c("Dec 13", "April 14", "Aug 2014", "Nov 2014","May 2015"),lty=c(1,5),pch=c(19,15,8,17,18),lwd=2,col=c("black","gold2","blue","green3","red"),ncol=2,horiz=FALSE,cex=0.6,title="months")

abline(v=4,lty=2)

#residuals plotted against distance

par(mar=c(5,5,4,2),cex=0.9)

plot(tempo1[tempo1$day==1,"dist"],tempo1[tempo1$day==1,"2"],type = "b",ylim=c(-2,2),pch=19,cex=1.5,xlab="Distance(km)",ylab="residulas",main="prediction with space and time",lwd=3,lty=4)

points(tempo1[tempo1$day==126,"dist"],tempo1[tempo1$day==126,"2"],type="b",col="gold2",pch=8,cex=1.5,lwd=3,lty=3)

points(tempo1[tempo1$day==260,"dist"],tempo1[tempo1$day==260,"2"],type="b",col="blue",pch=8,cex=1.5,lwd=3,lty=3)

points(tempo1[tempo1$day==336,"dist"],tempo1[tempo1$day==336,"2"],type="b",col="green3",pch=17,cex=1.5,lwd=3)

points(tempo1[tempo1$day==518,"dist"],tempo1[tempo1$day==518,"2"],type="b",col="red",pch=18,cex=2,lwd=3,lty=5)

legend("topright",inset=c(0,0),legend=c("Dec 2013","April 2014", "Aug 2014", "Nov 2014","May 2015"),lty=1,lwd=2,col=c("black","gold2","blue","green3","red"),ncol=5,horiz=FALSE,cex=0.7,title="months")

abline(v=4,lty=2)

## residuals regressed against predicted values

tempo3<-lm(tempo1[,2]~tempo1$pred1)

summary(tempo3)

## residulas against time and space

edit(residuals(tempo))

names(residuals(tempo))

rownames(alldata)

tempo<-lm(tempo1[,2]~dist+time+dist:time,data=alldata)

summary(tempo)

plot(tempo)

anova(tempo)

##***** Figure 9 *****###

tiff(file="Figure 9.jpg",width=10,height=14,units="in",pointsize = 12,bg ="transparent",res=800,compression="lzw")

par(mfrow=c(3,1), mar=c(3,4.5,2.5,3.5),cex=1.5,cex.axis=0.9,las=1,cex.main=1,cex.lab=0.8)

# water temperature plotted against spatial position

plot(alldata[alldata$day==1,"dist"],alldata[alldata$day==1,"temp"],type = "b",ylim=c(0,30),pch=19,xlab="Distance(km)",ylab="Water temperature (°C)",main="a) Water temperature with spatiotemporal prediction",lwd=3,lty=1)

points(alldata[alldata$day==126,"dist"],alldata[alldata$day==126,"temp"],type="b",col="gold2",pch=15,lwd=3,cex=1.2,lty=2)

points(alldata[alldata$day==260,"dist"],alldata[alldata$day==260,"temp"],type="b",col="blue",pch=8,cex=1.2,lwd=3,lty=3)

points(alldata[alldata$day==336,"dist"],alldata[alldata$day==336,"temp"],type="b",col="green3",pch=17,cex=1.2,lwd=3,lty=4)

points(alldata[alldata$day==518,"dist"],alldata[alldata$day==518,"temp"],type="b",col="red",pch=18,cex=1.4,lwd=3,lty=5)

legend("topright",inset=c(0,0),legend=c("Dec 13", "April 14", "Aug 2014", "Nov 2014","May 2015"),lty=c(1,5),pch=c(19,15,8,17,18),lwd=2,col=c("black","gold2","blue","green3","red"),ncol=2,horiz=FALSE,cex=0.6,title="months")

abline(v=4,lty=2)

#water temperaturewith spatiotemporal prediction

tempo<-lm(temp~dist+time+dist:time,data=alldata) # Checked model

tempo1<-cbind(cbind((fitted(tempo)),residuals(tempo)),alldata)

attributes(tempo1)

names(tempo1[,1:2])<-c("fitted","residual")

tempo1[,"2"]

plot(tempo1$dist[tempo1$time==1],tempo1[tempo1$time==1,"temp"],type="p",pch=19,col="black",xlab="Distance (km)",ylab="Water temperature (°C)",main="b) Water temperature with spatiotemporal prediction",ylim=c(0,30),cex=1,lwd=3,lty=1)

points(tempo1$dist[tempo1$time==1],tempo1[tempo1$time==2,"temp"],col="gold2",pch=15,cex=1.2,lwd=3,lty=2)

points(tempo1$dist[tempo1$time==1],tempo1[tempo1$time==3,"temp"],col="blue",pch=8,cex=1.2,lwd=3,lty=3)

points(tempo1$dist[tempo1$time==1],tempo1[tempo1$time==4,"temp"],col="green3",pch=17,cex=1.2,lwd=3,lty=4)

points(tempo1$dist[tempo1$time==1],tempo1[tempo1$time==5,"temp"],col="red",pch=18,cex=1.4,lwd=3,lty=5)

points(tempo1[tempo1$day==1,"dist"],tempo1[tempo1$day==1,"1"],type="l",col="black",pch=19,cex=1.2,lwd=3,lty=1)

points(tempo1[tempo1$day==126,"dist"],tempo1[tempo1$day==126,"1"],type="l",col="gold2",pch=15,cex=1.5,lwd=3,lty=2)

points(tempo1[tempo1$day==260,"dist"],tempo1[tempo1$day==260,"1"],type="l",col="blue",pch=8,cex=1.5,lwd=3,lty=3)

points(tempo1[tempo1$day==336,"dist"],tempo1[tempo1$day==336,"1"],type="l",col="green3",pch=17,cex=1.5,lwd=3,lty=4)

points(tempo1[tempo1$day==518,"dist"],tempo1[tempo1$day==518,"1"],type="l",col="red",pch=18,cex=2,lwd=3,lty=5)

legend("topright",inset=c(0,0),legend=c("Dec 13", "April 14", "Aug 2014", "Nov 2014","May 2015"),lty=c(1,5),pch=c(19,15,8,17,18),lwd=2,col=c("black","gold2","blue","green3","red"),ncol=2,horiz=FALSE,cex=0.6,title="months")

abline(v=4,lty=2)

#water temperature prediction with environmental variables

tempo<-lm(temp~rain3+airtemp+dayflow+res300+dayflow:res300+rain3:res300+dayflow:res300:rain3,data=alldata) ##Checked model

tempo1<-cbind(cbind((fitted(tempo)),residuals(tempo)),alldata)

attributes(tempo1)

names(tempo1[,1:2])<-c("fitted","residual")

tempo1[,"2"]

plot(tempo1[tempo1$day==1,"dist"],tempo1[tempo1$day==1,"1"],type = "b",ylim=c(0,30),pch=19,col="black",xlab="Distance(km)",ylab="Water temperature (°C)",main="c) Water temperature with other variables",lwd=3,lty=1)

points(tempo1[tempo1$day==126,"dist"],tempo1[tempo1$day==126,"1"],type="b",col="gold2",pch=15,lwd=3,cex=1.5,lty=2)

points(tempo1[tempo1$day==260,"dist"],tempo1[tempo1$day==260,"1"],type="b",col="blue",pch=8,cex=1.2,lwd=3,lty=3)

points(tempo1[tempo1$day==336,"dist"],tempo1[tempo1$day==336,"1"],type="b",col="green3",pch=17,cex=1.2,lwd=3,lty=4)

points(tempo1[tempo1$day==518,"dist"],tempo1[tempo1$day==518,"1"],type="b",col="red",pch=18,cex=1.4,lwd=3,lty=5)

legend("topright",inset=c(0,0),legend=c("Dec 13", "April 14", "Aug 2014", "Nov 2014","May 2015"),lty=c(1,5),pch=c(19,15,8,17,18),lwd=2,col=c("black","gold2","blue","green3","red"),ncol=2,horiz=FALSE,cex=0.6,title="months")

abline(v=4,lty=2)

dev.off()

#

# Zinc (zn)

#check the graph (scatterplot) for zn

par(mar=c(5,5,4,2),cex=0.9)

panel.cor <- function(x, y, digits = 2, cex.cor, ...)

{

usr <- par("usr"); on.exit(par(usr))

par(usr = c(0, 1, 0, 1))

# correlation coefficient

r <- cor(x, y)

method=c("spearman")

txt <- format(c(r, 0.123456789), digits = digits)[1]

txt <- paste("r= ", txt, sep = "")

text(0.5, 0.8, txt)

# p-value calculation

p <- cor.test(x, y)$p.value

txt2 <- format(c(p, 0.123456789), digits = digits)[1]

txt2 <- paste("p= ", txt2, sep = "")

if(p<0.01) txt2 <- paste("p= ", "<0.01", sep = "")

text(0.5, 0.3, txt2)

}

pairs(~dzn+eff+dist+log(zn)+ph1,data=alldata,upper.panel=panel.cor,pch=20,na.action = na.omit)

##regression with other variables

tempo<-lm(log(zn)~eff,data=alldata)##Adjusted R-squared: 0.1463

tempo<-lm(log(zn)~dist,data=alldata)##Adjusted R-squared: 0.09895

tempo<-lm(log(zn)~dzn,data=alldata)

##***** Figure *****###zinc plotted against spatial position

tiff(file="Figure.jpg",width=10,height=10,units="in",pointsize = 12,bg ="transparent",res=800,compression="lzw")

par(mfrow=c(2,1), mar=c(3,4.5,2.5,3.5),cex=1.5,cex.axis=0.9,las=1,cex.main=1,cex.lab=0.8)

plot(alldata[alldata$day==1,"dist"],alldata[alldata$day==1,"zn"],type = "b",ylim=c(0,120),pch=19,xlab="Distance(km)",ylab="Zinc (mg/L)",main="a) Zn plotted against spatial position",lwd=3,lty=1)

points(alldata[alldata$day==126,"dist"],alldata[alldata$day==126,"zn"],type="b",col="gold2",pch=15,lwd=3,cex=1.2,lty=2)

points(alldata[alldata$day==260,"dist"],alldata[alldata$day==260,"zn"],type="b",col="blue",pch=8,cex=1.2,lwd=3,lty=3)

points(alldata[alldata$day==336,"dist"],alldata[alldata$day==336,"zn"],type="b",col="green3",pch=17,cex=1.2,lwd=3,lty=4)

points(alldata[alldata$day==518,"dist"],alldata[alldata$day==518,"zn"],type="b",col="red",pch=18,cex=1.4,lwd=3,lty=5)

legend("topleft",inset=c(0,0),legend=c("Dec 13", "April 14", "Aug 2014", "Nov 2014","May 2015"),lty=c(1,5),pch=c(19,15,8,17,18),lwd=2,col=c("black","gold2","blue","green3","red"),ncol=2,horiz=FALSE,cex=0.6,title="months")

abline(v=4,lty=2)

plot(alldata[alldata$day==1,"dist"],alldata[alldata$day==1,"zn1"],type = "b",ylim=c(0,30),pch=19,xlab="Distance(km)",ylab="Zinc (mg/L)",main="b) Zn plotted against spatial position/n (without outliers)",lwd=3,lty=1)

points(alldata[alldata$day==126,"dist"],alldata[alldata$day==126,"zn1"],type="b",col="gold2",pch=15,lwd=3,cex=1.2,lty=2)

points(alldata[alldata$day==260,"dist"],alldata[alldata$day==260,"zn1"],type="b",col="blue",pch=8,cex=1.2,lwd=3,lty=3)

points(alldata[alldata$day==336,"dist"],alldata[alldata$day==336,"zn1"],type="b",col="green3",pch=17,cex=1.2,lwd=3,lty=4)

points(alldata[alldata$day==518,"dist"],alldata[alldata$day==518,"zn1"],type="b",col="red",pch=18,cex=1.4,lwd=3,lty=5)

legend("topleft",inset=c(0,0),legend=c("Dec 13", "April 14", "Aug 2014", "Nov 2014","May 2015"),lty=c(1,5),pch=c(19,15,8,17,18),lwd=2,col=c("black","gold2","blue","green3","red"),ncol=2,horiz=FALSE,cex=0.6,title="months")

abline(v=4,lty=2)

dev.off()

# Multiple regression analysis for zn as a function of time, distance and eff to check spatiotemporal variation of log(zn)

tempo<-lm(log(zn)~eff+eff:dist+eff:I(dist^2)+eff:dist:time+eff:I(dist^2):time,data=alldata)

tempo<-lm(log(zn)~eff+eff:dist+eff:I(dist^2),data=alldata)## significant ones ##*********##

anova(tempo) ##*********##

tempo<-lm(log(zn)~eff+eff:I(dist^2),data=alldata)

tempo<-lm(log(zn)~eff,data=alldata)

summary(tempo) ##***** Table 20 - appendix *****##

anova(tempo) ##***** Table 21 - appendix *****##

plot(tempo) ## checking the diagnostic plots to check the multiple regresstion assumptions

qqnorm(tempo$residuals)

qqline(tempo$residuals)

hist(tempo$residuals)

summary(tempo)

tempo1<-cbind(cbind(exp(fitted(tempo)),residuals(tempo)),alldata)

attributes(tempo1)

names(tempo1[,1:2])<-c("fitted","residual")

edit(tempo1)

tempo1[,"2"]

#observed zn plots with spatiotemporal model predictions

plot(tempo1$dist[tempo1$time==1],tempo1[tempo1$time==1,"zn"],type="p",pch=19,col="black",xlab="Distance (km)",ylab="Zn",main="Zinc with spatiotemporal prediction",ylim=c(0,20),cex=1,lwd=3,lty=1)

points(tempo1$dist[tempo1$time==1],tempo1[tempo1$time==2,"zn"],col="gold2",pch=15,cex=1.5,lwd=3,lty=2)

points(tempo1$dist[tempo1$time==1],tempo1[tempo1$time==3,"zn"],col="blue",pch=8,cex=1.5,lwd=3,lty=3)

points(tempo1$dist[tempo1$time==1],tempo1[tempo1$time==4,"zn"],col="green3",pch=17,cex=1.5,lwd=3,lty=4)

points(tempo1$dist[tempo1$time==1],tempo1[tempo1$time==5,"zn"],col="red",pch=18,cex=2,lwd=3,lty=5)

points(tempo1[tempo1$day==1,"dist"],tempo1[tempo1$day==1,"1"],type="l",col="black",pch=19,cex=1.5,lwd=3,lty=1)

points(tempo1[tempo1$day==126,"dist"],tempo1[tempo1$day==126,"1"],type="l",col="gold2",pch=15,cex=1.5,lwd=3,lty=2)

points(tempo1[tempo1$day==260,"dist"],tempo1[tempo1$day==260,"1"],type="l",col="blue",pch=8,cex=1.5,lwd=3,lty=3)

points(tempo1[tempo1$day==336,"dist"],tempo1[tempo1$day==336,"1"],type="l",col="green3",pch=17,cex=1.5,lwd=3,lty=4)

points(tempo1[tempo1$day==518,"dist"],tempo1[tempo1$day==518,"1"],type="l",col="red",pch=18,cex=2,lwd=3,lty=5)

legend("topleft",inset=c(0,0),legend=c("Dec 13", "April 14", "Aug 2014", "Nov 2014","May 2015"),lty=c(1,5),pch=c(19,15,8,17,18),lwd=2,col=c("black","gold2","blue","green3","red"),ncol=2,horiz=FALSE,cex=0.6,title="months")

abline(v=4,lty=2)

# Multiple regression analysis for zn and factors affecting it to check which variables explain time:space (spatiotemporal variation in zn

tempo<-lm(log(zn1)~eff:I(dzn/dayflow)+eff:dist+eff:temp2:dist+eff:dist:I(dzn/dayflow)+eff:I(dist^2)+eff:temp2:I(dist^2)+eff:I(dist^2):I(dzn/dayflow),data=alldata)### final

tempo<-lm(log(zn)~road1000+res1000+treat1000,data=alldata)### final

tempo<-lm(log(zn1)~road1000+res1000+treat1000,data=alldata)### final ##*********###

summary(tempo)

anova(tempo)##*********###

tempo<-lm(log(zn1)~treat1000,data=alldata)###

summary(tempo)

anova(tempo)

tempo<-lm(log(zn1)~eff,data=alldata)

summary(tempo)

anova(tempo)

plot(tempo)

tempo1<-cbind(cbind(exp(fitted(tempo)),residuals(tempo)),alldata)

names(tempo1[,1:2])<-c("fitted","residual")

edit(tempo1)

tempo1[,"2"]

#zn plots with spatiotemporal model predictions (including other variables)

plot(tempo1[tempo1$day==1,"dist"],tempo1[tempo1$day==1,"1"],type = "l",ylim=c(0,40),pch=19,col="black",xlab="Distance(km)",ylab="Zinc",main="Prediction with environmental parameters",lwd=3,lty=1)

points(tempo1[tempo1$day==126,"dist"],tempo1[tempo1$day==126,"1"],type="l",col="gold2",pch=15,lwd=3,cex=1.5,lty=2)

points(tempo1[tempo1$day==260,"dist"],tempo1[tempo1$day==260,"1"],type="l",col="blue",pch=8,cex=1.2,lwd=3,lty=3)

points(tempo1[tempo1$day==336,"dist"],tempo1[tempo1$day==336,"1"],type="l",col="green3",pch=17,cex=1.2,lwd=3,lty=4)

points(tempo1[tempo1$day==518,"dist"],tempo1[tempo1$day==518,"1"],type="l",col="red",pch=18,cex=1.4,lwd=3,lty=5)

legend("topleft",inset=c(0,0),legend=c("Dec 13", "April 14", "Aug 2014", "Nov 2014","May 2015"),lty=c(1,5),pch=c(19,15,8,17,18),lwd=2,col=c("black","gold2","blue","green3","red"),ncol=2,horiz=FALSE,cex=0.6,title="months")

abline(v=4,lty=2)

#residuals

par(mar=c(5,5,4,2),cex=0.9)

plot(tempo1[tempo1$day==1,"dist"],tempo1[tempo1$day==1,"2"],type = "b",ylim=c(-1,20),pch=19,cex=1.5,xlab="Distance(km)",ylab="residulas",main="prediction with space and time",lwd=3,lty=4)

points(tempo1[tempo1$day==126,"dist"],tempo1[tempo1$day==126,"2"],type="b",col="gold2",pch=8,cex=1.5,lwd=3,lty=3)

points(tempo1[tempo1$day==260,"dist"],tempo1[tempo1$day==260,"2"],type="b",col="blue",pch=8,cex=1.5,lwd=3,lty=3)

points(tempo1[tempo1$day==336,"dist"],tempo1[tempo1$day==336,"2"],type="b",col="green3",pch=17,cex=1.5,lwd=3)

points(tempo1[tempo1$day==518,"dist"],tempo1[tempo1$day==518,"2"],type="b",col="red",pch=18,cex=2,lwd=3,lty=5)

legend("topright",inset=c(0,0),legend=c("Dec 2013","April 2014", "Aug 2014", "Nov 2014","May 2015"),lty=1,lwd=2,col=c("black","gold2","blue","green3","red"),ncol=5,horiz=FALSE,cex=0.7,title="months")

abline(v=4,lty=2)

xyplot(fitted(tempo)~dist, groups=day,data=tempo1,type="l",auto.key=TRUE)

xyplot(residuals(tempo)~dist, groups=day,data=tempo1,type="l",auto.key=TRUE)

tempo3<-lm(tempo1[,2]~tempo1$pred1)

summary(tempo3)

## residulas against time and space

edit(residuals(tempo))

names(residuals(tempo))

rownames(alldata)

tempo<-lm(tempo1[,2]~as.factor(eff)+dist+time+as.factor(eff):time+as.factor(eff):dist,data=alldata)

summary(tempo)

plot(tempo)

anova(tempo)

#

# chla in discharge

## We didn't have enough data for time, but in studies with data available for years, dischrge variables can be modeled as a function of time

par(mar=c(5,5,4,2),cex=0.9)

panel.cor <- function(x, y, digits = 2, cex.cor, ...)

{

usr <- par("usr"); on.exit(par(usr))

par(usr = c(0, 1, 0, 1))

# correlation coefficient

r <- cor(x, y)

method=c("spearman")

txt <- format(c(r, 0.123456789), digits = digits)[1]

txt <- paste("r= ", txt, sep = "")

text(0.5, 0.8, txt)

# p-value calculation

p <- cor.test(x, y)$p.value

txt2 <- format(c(p, 0.123456789), digits = digits)[1]

txt2 <- paste("p= ", txt2, sep = "")

if(p<0.01) txt2 <- paste("p= ", "<0.01", sep = "")

text(0.5, 0.3, txt2)

}

pairs(~solar+airtemp+time+dchla,data=alldata,upper.panel=panel.cor,pch=20,na.action = na.omit)

##regression with other variables

time1<-2*pi*alldata$day/365

tempo<-lm(dchla~solar+airtemp+time1,data=alldata)

tempo<-lm(dchla~airtemp+solar,data=alldata)

tempo<-lm(dchla~solar,data=alldata)## final

summary(tempo)

anova(tempo)

plot(tempo) ## checking the diagnostic plots to check the multiple regresstion assumptions

qqnorm(tempo$residuals)

qqline(tempo$residuals)

hist(tempo$residuals)

#

# air temperature

par(mar=c(5,5,4,2),cex=0.9)

panel.cor <- function(x, y, digits = 2, cex.cor, ...)

{

usr <- par("usr"); on.exit(par(usr))

par(usr = c(0, 1, 0, 1))

# correlation coefficient

r <- cor(x, y)

method=c("spearman")

txt <- format(c(r, 0.123456789), digits = digits)[1]

txt <- paste("r= ", txt, sep = "")

text(0.5, 0.8, txt)

# p-value calculation

p <- cor.test(x, y)$p.value

txt2 <- format(c(p, 0.123456789), digits = digits)[1]

txt2 <- paste("p= ", txt2, sep = "")

if(p<0.01) txt2 <- paste("p= ", "<0.01", sep = "")

text(0.5, 0.3, txt2)

}

pairs(~airtemp+solar+time,data=alldata,upper.panel=panel.cor,pch=20,na.action = na.omit)

##regression with other variables

time1<-2*pi*alldata$day/365

time1

tempo<-lm(airtemp~time1,data=alldata)

summary(tempo)

summary(tempo)

anova(tempo)

plot(tempo) ## checking the diagnostic plots to check the multiple regresstion assumptions

qqnorm(tempo$residuals)

qqline(tempo$residuals)

hist(tempo$residuals)

#

# solar

par(mar=c(5,5,4,2),cex=0.9)

panel.cor <- function(x, y, digits = 2, cex.cor, ...)

{

usr <- par("usr"); on.exit(par(usr))

par(usr = c(0, 1, 0, 1))

# correlation coefficient

r <- cor(x, y)

method=c("spearman")

txt <- format(c(r, 0.123456789), digits = digits)[1]

txt <- paste("r= ", txt, sep = "")

text(0.5, 0.8, txt)

# p-value calculation

p <- cor.test(x, y)$p.value

txt2 <- format(c(p, 0.123456789), digits = digits)[1]

txt2 <- paste("p= ", txt2, sep = "")

if(p<0.01) txt2 <- paste("p= ", "<0.01", sep = "")

text(0.5, 0.3, txt2)

}

pairs(~solar+time,data=alldata,upper.panel=panel.cor,pch=20,na.action = na.omit)

#

# canopy cover

#check the graph (scatterplot) for canopy cover

par(mar=c(5,5,4,2),cex=0.9)

panel.cor <- function(x, y, digits = 2, cex.cor, ...)

{

usr <- par("usr"); on.exit(par(usr))

par(usr = c(0, 1, 0, 1))

# correlation coefficient

r <- cor(x, y)

method=c("spearman")

txt <- format(c(r, 0.123456789), digits = digits)[1]

txt <- paste("r= ", txt, sep = "")

text(0.5, 0.8, txt)

# p-value calculation

p <- cor.test(x, y)$p.value

txt2 <- format(c(p, 0.123456789), digits = digits)[1]

txt2 <- paste("p= ", txt2, sep = "")

if(p<0.01) txt2 <- paste("p= ", "<0.01", sep = "")

text(0.5, 0.3, txt2)

}

pairs(~dist+canop+veg30m+log(veg30m),data=alldata,upper.panel=panel.cor,pch=20,na.action = na.omit)

#multiple regression with time and space

tempo<-lm(log(canop)~time+dist+time:dist,data=alldata) #Checked model, no clear spatiotempral patterns

summary(tempo)

anova(tempo)

plot(tempo) ## checking the diagnostic plots to check the multiple regresstion assumptions

qqnorm(tempo$residuals)

qqline(tempo$residuals)

hist(tempo$residuals)

#Canopy plotted against spatial position

plot(bugenv[bugenv$day==1,"dist"],bugenv[bugenv$day==1,"canop"],type = "b",ylim=c(0,100),pch=19,xlab="Distance(km)",ylab="Alkalinity (mg/L)",main="Canopy cover plotted against spatial position",lwd=3,lty=1)

points(bugenv[bugenv$day==126,"dist"],bugenv[bugenv$day==126,"canop"],type="b",col="gold2",pch=15,lwd=3,cex=1.2,lty=2)

points(bugenv[bugenv$day==260,"dist"],bugenv[bugenv$day==260,"canop"],type="b",col="blue",pch=8,cex=1.2,lwd=3,lty=3)

points(bugenv[bugenv$day==336,"dist"],bugenv[bugenv$day==336,"canop"],type="b",col="green3",pch=17,cex=1.2,lwd=3,lty=4)

points(bugenv[bugenv$day==518,"dist"],bugenv[bugenv$day==518,"canop"],type="b",col="red",pch=18,cex=1.4,lwd=3,lty=5)

legend("topright",inset=c(0,0),legend=c("Dec 13", "April 14", "Aug 2014", "Nov 2014","May 2015"),lty=c(1,5),pch=c(19,15,8,17,18),lwd=2,col=c("black","gold2","blue","green3","red"),ncol=2,horiz=FALSE,cex=0.6,title="months")

abline(v=4,lty=2)

##canopy cover regression with other variables

tempo<-lm(canop~veg30m,data=alldata) ##Adjusted R-squared: 0.1836

summary(tempo)

anova(tempo)

plot(tempo) ## checking the diagnostic plots to check the regresstion assumptions

qqnorm(tempo$residuals)

qqline(tempo$residuals)

hist(tempo$residuals)

#

### riparian vegetation

par(mar=c(5,5,4,2),cex=0.9)

panel.cor <- function(x, y, digits = 2, cex.cor, ...)

{

usr <- par("usr"); on.exit(par(usr))

par(usr = c(0, 1, 0, 1))

# correlation coefficient

r <- cor(x, y)

method=c("spearman")

txt <- format(c(r, 0.123456789), digits = digits)[1]

txt <- paste("r= ", txt, sep = "")

text(0.5, 0.8, txt)

# p-value calculation

p <- cor.test(x, y)$p.value

txt2 <- format(c(p, 0.123456789), digits = digits)[1]

txt2 <- paste("p= ", txt2, sep = "")

if(p<0.01) txt2 <- paste("p= ", "<0.01", sep = "")

text(0.5, 0.3, txt2)

}

pairs(~dist+veg30m,data=alldata,upper.panel=panel.cor,pch=20,na.action = na.omit)

##regression with other variables

tempo<-lm(veg30m~dist,data=alldata)

summary(tempo)

anova(tempo)

plot(tempo) ## checking the diagnostic plots to check the regresstion assumptions

qqnorm(tempo$residuals)

qqline(tempo$residuals)

hist(tempo$residuals)

#

## creek flow rate

#check the graph (scatterplot) for creek flow rate

par(mar=c(5,5,4,2),cex=0.9)

panel.cor <- function(x, y, digits = 2, cex.cor, ...)

{

usr <- par("usr"); on.exit(par(usr))

par(usr = c(0, 1, 0, 1))

# correlation coefficient

r <- cor(x, y)

method=c("spearman")

txt <- format(c(r, 0.123456789), digits = digits)[1]

txt <- paste("r= ", txt, sep = "")

text(0.5, 0.8, txt)

# p-value calculation

p <- cor.test(x, y)$p.value

txt2 <- format(c(p, 0.123456789), digits = digits)[1]

txt2 <- paste("p= ", txt2, sep = "")

if(p<0.01) txt2 <- paste("p= ", "<0.01", sep = "")

text(0.5, 0.3, txt2)

}

pairs(~rain3+dflow+dayflow,data=alldata,upper.panel=panel.cor,pch=20,na.action = na.omit)

pairs(~log(rain3)+log(rain2)+log(dflow)+log(dayflow),data=alldata,upper.panel=panel.cor,pch=20,na.action = na.omit)

##regression with other variables

tempo<-lm(dayflow~dflow,data=alldata)##Adjusted R-squared: 0.5844

tempo<-lm(dayflow~rain3,data=alldata) ##Adjusted R-squared: 0.5513

summary(tempo)

# Multiple regression analysis for creek flow rate

tempo<-lm(dayflow~dflow+rain3,data=alldata) # Checked final model

anova(tempo) ##*********###

summary(tempo) ##*********###

plot(tempo) ## checking the diagnostic plots to check the multiple regresstion assumptions

qqnorm(tempo$residuals)

qqline(tempo$residuals)

hist(tempo$residuals)

##***** Flow rate figure *****##

## plot displaying how the model explains variation in creek flow rate at different time of sampling

tempo1<-cbind(cbind((fitted(tempo)),residuals(tempo)),alldata)

names(tempo1[,1:2])<-c("fitted","residual")

tempo1[,"2"]

tiff(file="Flow rate figure",width=10,height=4.6,units="in",pointsize = 12,bg ="transparent",res=800,compression="lzw")

par(mfrow=c(1,1), mar=c(3,4.5,2.5,3.5),cex=1.5,cex.axis=0.9,las=1,cex.main=1,cex.lab=0.8)

plot(mydates,bugenv[bugenv$dist==0,"dayflow"],type = "b",ylim=c(10,120),pch=16,cex=1.5,xlab="Day",ylab="Creek flow rate (ML/day)",main="Observed and predicted values of \n creek flow rate against time ",lwd=3,lty=2, col=c("red"),xaxt="n")

axis.Date(side=1,mydates,at=seq(mydaterange[1],mydaterange[2],by="month"),format="%b-%y")

points(mydates,tempo1[tempo1$dist==0,"1"],type = "l",ylim=c(0,120),pch=19,cex=1.5,xlab="day",ylab="Creek flow rate (ML/day)",main="Average daily Creek flow rate plotted against time",lwd=3,lty=1,xaxt="n")

legend("topright",inset=c(0,0),legend=c("Observed values", "Predicted values"),lty=c(1,5),pch=c(19,15,8,17,18),lwd=2,col=c("red","black"),ncol=1,horiz=FALSE,cex=0.8,title="")

dev.off()

##landuse

tempo<-lm(res300~dist,data=alldata)

summary(tempo)

anova(tempo)

plot(tempo)

##vel

tempo<-lm(log(vel)~dist,data=alldata)

summary(tempo)

anova(tempo)

plot(tempo)

##ph2

tempo<-lm(ph2~rain2,data=alldata)

summary(tempo)

anova(tempo)

plot(tempo)

##time and other factors

par(mar=c(5,5,4,2),cex=4)

panel.cor <- function(x, y, digits = 2, cex.cor, ...)

{

usr <- par("usr"); on.exit(par(usr))

par(usr = c(0, 1, 0, 1))

# correlation coefficient

r <- cor(x, y)

method=c("spearman")

txt <- format(c(r, 0.123456789), digits = digits)[1]

txt <- paste("r= ", txt, sep = "")

text(0.5, 0.8, txt)

# p-value calculation

p <- cor.test(x, y)$p.value

txt2 <- format(c(p, 0.123456789), digits = digits)[1]

txt2 <- paste("p= ", txt2, sep = "")

if(p<0.01) txt2 <- paste("p= ", "<0.01", sep = "")

text(0.5, 0.3, txt2)

}

pairs(~log(dalk)+log(dcond)+log(dtoc)+log(dno3)+log(dtp)+log(dchla)+log(dflow)+log(rain3)+log(rain2),data=alldata,upper.panel=panel.cor,pch=20,na.action = na.omit)

########d-separation statements

####

library(dagR)

library(ggm)

library(pcalg)

library(gRbase)

library(gRain)

library(igraph)

library(piecewiseSEM)

install.packages("ggm")

###adjacency matrix

sink('dsep.txt', append=TRUE)

dag.draw(mywangdag)

####basis set for the conditional independencies

## series1

mywangdag<-DAG(bugs~temp+chla+cond+toc,chla~no3+temp+dchla+tp+dayflow+eff+turb+solar,toc~dayflow+dtoc+eff+dist+time+rain3,cond~dayflow+dcond+eff+dist+time+dflow,temp~canop+rain3+dayflow+res300+airtemp, no3~dayflow+dno3+eff+temp+time+dist,tp~dayflow+dtp+eff+time+ph2+alk, dayflow~dflow+rain3,alk~rain3+dist+time+dayflow+dalk+eff, turb~dayflow+res300+rain3, eff~dist, res300~dist, veg30m~dist,canop~veg30m,ph2~rain3, solar~climate,rain3~climate,airtemp~climate,operation~airtemp+rain3+solar+time,dchla~operation,dtp~operation, dcond~operation,dalk~operation,dno3~operation,dtoc~operation,dflow~operation, climate~time, order=TRUE)

sink('DAG1.txt', append=TRUE)

print(mywangdag)

basis.set<-basiSet(mywangdag)

print(basis.set)

sink(NULL)

mywangdag<- as.matrix(mywangdag)

adjmydag<-graph.adjacency(mywangdag,(mode="directed"),weighted=TRUE,diag=FALSE)

str(adjmydag)

plot(adjmydag)

plot.igraph(adjmydag,vertex.label=V(adjmydag)$name,vertex.label.color="black",edge.color="blue",edge.width=E(adjmydag)$weight, edge.arrow.size=0.1,vertex.size=7, vertex.label.dist=0.5, vertex.color="red")

###########################################Checking conditional independencies

####basis set for the conditional independencies ## Iteration3

## Iteration3

mywangdag2<-DAG(bugs~temp+chla+cond+toc+zn,chla~no3+temp+dchla+tp+dayflow+eff+turb+solar,toc~dayflow+dtoc+eff+dist+time,cond~alk+tp+dist+time+eff,temp~rain3+dayflow+res300+airtemp,zn~eff,no3~rain2+rain3+dayflow+dno3+eff+temp+time+dist,tp~alk+ph2+dayflow+dtp+eff+time,ph2~rain2,dayflow~dflow+rain3,alk~rain3+dist+time+dayflow+dalk+eff,turb~dayflow+vel+rain3,eff~dist,res300~dist,turb~vel,vel~dist,solar~climate,rain2~climate, rain3~climate,airtemp~climate,operation~airtemp+rain3+solar+time,dchla~operation,dtp~operation,dalk~operation,dno3~operation,dtoc~operation,dflow~operation,climate~time, order=TRUE)

sink('DAG3.txt', append=TRUE)

print(mywangdag2)

basis.set2<-basiSet(mywangdag2)

print(basis.set2)

sink(NULL)

################################## ## Iteration3 of conditional independencies

#

#2# d-separation (ph2 des time| rain2)

tempo<-lm(ph2~rain2,data=alldata)

independence.test1<-lm(ph2~rain2+time,data=alldata)

summary(independence.test1)

anova(independence.test1)

anova(tempo,independence.test1)

#8# d-separation (dayflow des time| rain3, dflow)

tempo<-lm(dayflow~dflow+rain3,data=alldata)

independence.test1<-lm(dayflow~dflow+rain3+time,data=alldata)

summary(independence.test1)

anova(independence.test1)

anova(tempo,independence.test1)

# not enough data

#14# d-separation (vel des time| dist)

tempo<-lm(vel~dist,data=alldata)

independence.test1<-lm(vel~dist+time,data=alldata)

summary(independence.test1)

anova(independence.test1)

anova(tempo,independence.test1)

#15# d-separation (turb des time| rain3, dayflow, vel)

tempo<-lm(log(turb)~log(rain3)+log(dayflow)+log(vel),data=alldata)###checked model5

anova(tempo)

summary(tempo)

independence.test1<-lm(log(turb)~log(rain3)+log(dayflow)+log(vel)+time,data=alldata)

summary(independence.test1)

anova(independence.test1)

anova(tempo,independence.test1)

#16# d-separation (res300 des time| dist)

tempo<-lm(res300~dist,data=alldata)

independence.test1<-lm(res300~dist+time,data=alldata)

summary(independence.test1)

anova(independence.test1)

anova(tempo,independence.test1)

# not enough data

#17# d-separation (temp des time| airtemp, rain3, dayflow, res300)

tempo<-lm(temp~rain3+airtemp+dayflow+res300+dayflow:res300+rain3:res300+dayflow:res300:rain3,data=alldata)

anova(tempo)

summary(tempo)

independence.test1<-lm(temp~rain3+airtemp+dayflow+res300+dayflow:res300+rain3:res300+dayflow:res300:rain3+time,data=alldata)

summary(independence.test1)

anova(independence.test1)

anova(tempo,independence.test1)

#19# d-separation (chla des time| solar, dayflow, dchla, turb, temp, eff, tp, no3)

tempo<-lm(log(chla)~solar+temp+tp+no3+turb+solar:temp+eff:log(I(dchla/dayflow)),data=alldata[complete.cases(alldata[,c("temp","no3","tp")]),])###checked model+revision

independence.test1<-lm(log(chla)~solar+temp+tp+no3+turb+solar:temp+eff:log(I(dchla/dayflow))+time,data=alldata[complete.cases(alldata[,c("temp","no3","tp")]),])###checked model+revision

anova(tempo)

summary(tempo)

summary(independence.test1)

anova(independence.test1)

anova(tempo,independence.test1)

#20# d-separation (zn des time| eff)

tempo<-lm(log(zn)~eff,data=alldata)

independence.test1<-lm(log(zn)~eff+time,data=alldata)

summary(independence.test1)

anova(independence.test1)

anova(tempo,independence.test1)

#21# d-separatin (bugs des time| temp, chla, toc, cond, zn)

wang.cap3<-capscale(formula=wangbug.BC~time+Condition(temp+cond+temp:cond+toc+temp:toc+log(chla)+log(zn)),data=alldata,comm=wangbug,add=TRUE,na.action=na.omit)#

summary(wang.cap3)

wang.anova3<-anova(wang.cap3,by="term",permutations = how(nperm=9999))

print(wang.anova3)

edit(alldata)

logchla<-log(alldata$chla)

datalogchla<-cbind(logchla,alldata)

datalogchla1<-datalogchla[complete.cases(datalogchla[,c("temp","cond","toc","chla")]),]#

design<-scale(model.matrix(~temp+cond+temp:cond+toc+temp:toc+log(chla)+log(zn),data=wangenv),center=FALSE,scale=FALSE)

edit(design)

wangbug1<-wangbug[9:40,]

wangbug1.BC<-vegdist(sqrt(wangbug1))

n<-dim(wangbug1)[1]

p<-n-1

wangbug1.mds<-cmdscale(wangbug1.BC, k = p, eig = TRUE, add = TRUE, x.ret = FALSE)

edit(wangbug1)

pco.predict<-qr.fitted(qr(design),wangbug1.mds$points)

pco.resid<-wangbug1.mds$points-pco.predict##compute pco residuals

plot(datalogchla1$time, pco.resid[,1],type="b", xlab="time", ylab="pco1 residuals")

xyplot(pco.resid[,1]~datalogchla1$time, xlab="time", ylab="pco1 residuals")

independence.check<-lm(pco.resid[,1]~datalogchla1$time, data=alldata)

summary(independence.check)

anova(independence.check)

#76# d-separation (ph2 des dayflow| rain2, rain3, dflow)

tempo<-lm(ph2~rain2+rain3+dflow,data=alldata)

independence.test1<-lm(ph2~rain2+rain3+dflow+dayflow,data=alldata)

summary(independence.test1)

anova(independence.test1)

anova(tempo,independence.test1)

#80# d-separation (ph2 des dist| rain2)

tempo<-lm(ph2~rain2,data=alldata)

independence.test1<-lm(ph2~rain2+dist,data=alldata)

summary(independence.test1)

anova(independence.test1)

anova(tempo,independence.test1)

#81# d-separation (ph2 des vel| rain2,dist)

tempo<-lm(ph2~rain2+dist,data=alldata)

independence.test1<-lm(ph2~rain2+dist+vel,data=alldata)

summary(independence.test1)

anova(independence.test1)

anova(tempo,independence.test1)

#82# d-separation (ph2 des turb| rain2,rain3,dayflow, vel)

tempo<-lm(ph2~rain2+rain3+dayflow+vel,data=alldata)

independence.test1<-lm(ph2~rain2+rain3+dayflow+vel+turb,data=alldata)

summary(independence.test1)

anova(independence.test1)

anova(tempo,independence.test1)

#83# d-separation (ph2 des res300| rain2,dist)

tempo<-lm(ph2~rain2+dist,data=alldata)

independence.test1<-lm(ph2~rain2+dist+res300,data=alldata)

summary(independence.test1)

anova(independence.test1)

anova(tempo,independence.test1)

#84# d-separation (ph2 des temp| rain2,rain3,airtemp,dayflow,res300)

tempo<-lm(ph2~rain2+rain3+airtemp+dayflow+res300,data=alldata)

independence.test1<-lm(ph2~rain2+rain3+airtemp+dayflow+res300+temp,data=alldata)

summary(independence.test1)

anova(independence.test1)

anova(tempo,independence.test1)

#86# d-separation (alk des ph2| rain2,rain3,time,dalk,dayflow,dist,eff)

tempo<-lm(log(alk)~rain2+rain3+dayflow+eff:I(log(dalk/dayflow)):time+eff:I(log(dalk/dayflow)):I(dist==4.08):time,data=alldata) ##checked model5+revision

anova(tempo)

independence.test1<-lm(log(alk)~rain2+rain3+dayflow+eff:I(log(dalk/dayflow)):time+eff:I(log(dalk/dayflow)):I(dist==4.08):time+ph2,data=alldata) ##checked model5+revision

summary(independence.test1)

anova(independence.test1)

anova(tempo,independence.test1)

#87# d-separation (cond des ph2| rain2,alk,tp)

tempo<-lm(log(cond)~tp+alk+time+dist:time:eff+rain2,data=alldata[complete.cases(alldata[,c("cond")]),])##final checked model5+revision

independence.test1<-lm(log(cond)~tp+alk+time+dist:time:eff+rain2+ph2,data=alldata[complete.cases(alldata[,c("cond")]),])##final checked model5+revision

summary(independence.test1)

anova(independence.test1)

anova(tempo,independence.test1)

#88# d-separation (no3 des ph2| rain2,rain3,time,dno3,dayflow,dist,eff)

tempo<-lm(log(no3)~temp+rain2+rain3+eff:I(log(dno3)/dayflow):time+eff:I(log(dno3)/dayflow):I(dist==4.08):time,data=alldata)##final checked model5+revision

independence.test1<-lm(log(no3)~temp+rain2+rain3+eff:I(log(dno3)/dayflow):time+eff:I(log(dno3)/dayflow):I(dist==4.08):time+ph2,data=alldata)##final checked model5+revision

summary(independence.test1)

anova(independence.test1)

anova(tempo,independence.test1)

#89# d-separation (chla des ph2| rain2,solar, dayflow, dchla, turb, temp, eff, tp, no3)

tempo<-lm(log(chla)~solar+temp+tp+no3+turb+solar:temp+eff:log(I(dchla/dayflow))+rain2,data=alldata[complete.cases(alldata[,c("temp","no3","tp")]),])###final checked model5+revision

anova(tempo)

independence.test1<-lm(log(chla)~solar+temp+tp+no3+turb+solar:temp+eff:log(I(dchla/dayflow))+rain2+ph2,data=alldata[complete.cases(alldata[,c("temp","no3","tp")]),])##final checked model5+revision

summary(independence.test1)

anova(independence.test1)

anova(tempo,independence.test1)

#90# d-separation (zn des ph2| rain2,eff)

tempo<-lm(log(zn1)~rain2+eff,data=alldata)

independence.test1<-lm(log(zn1)~rain2+eff+ph2,data=alldata)

summary(independence.test1)

anova(independence.test1)

anova(tempo,independence.test1)

#91# d-separation (toc des ph2| rain2,time, rain3, dayflow, dtoc, dist, eff)

tempo<-lm((toc)~dayflow+rain2+rain3+eff:I((dtoc/dayflow)):time+eff:I((dtoc/dayflow)):I(dist==4.08):time,data=alldata)##final checked model5+revision

independence.test1<-lm((toc)~dayflow+rain2+rain3+eff:I((dtoc/dayflow)):time+eff:I((dtoc/dayflow)):I(dist==4.08):time+ph2,data=alldata)##final checked model5+revision

summary(independence.test1)

anova(independence.test1)

anova(tempo,independence.test1)

#92# d-separation (bugs des ph2| temp, chla, toc, cond, zn, rain2)

wang.cap3<-capscale(formula=wangbug.BC~ph2+Condition(temp+cond+temp:cond+toc+temp:toc+log(chla))+log(zn)+rain2,data=alldata,comm=wangbug,add=TRUE,na.action=na.omit)#

summary(wang.cap3)

wang.anova3<-anova(wang.cap3,by="term",permutations = how(nperm=9999))

print(wang.anova3)

logchla<-log(alldata$chla)

datalogchla<-cbind(logchla,alldata)

datalogchla1<-datalogchla[complete.cases(datalogchla[,c("temp","cond","toc","chla")]),]#

design<-scale(model.matrix(~cond+temp:cond+toc+temp:toc+log(chla)+log(zn)+rain2,data=alldata),center=FALSE,scale=FALSE)

#edit(design)

wangbug1<-wangbug[9:40,]

wangbug1.BC<-vegdist(sqrt(wangbug1))

n<-dim(wangbug1)[1]

p<-n-1

wangbug1.mds<-cmdscale(wangbug1.BC, k = p, eig = TRUE, add = TRUE, x.ret = FALSE)

#edit(wangbug1)

pco.predict<-qr.fitted(qr(design),wangbug1.mds$points)

pco.resid<-wangbug1.mds$points-pco.predict##compute pco residuals

plot(datalogchla1$dist, pco.resid[,1],type="p", xlab="dist", ylab="pco1 residuals")

xyplot(pco.resid[,1]~datalogchla1$ph2, xlab="ph2", ylab="pco1 residuals")

independence.check<-lm(pco.resid[,1]~datalogchla1$ph2, data=alldata)

summary(independence.check)

anova(independence.check)

#208# d-separatin (dayflow des dist| rain3, dflow)

tempo<-lm(dayflow~dflow+rain3,data=alldata)

independence.test1<-lm(dayflow~dflow+rain3+dist,data=alldata)

summary(independence.test1)

anova(independence.test1)

# not enough data

#209# d-separation (vel des dayflow| rain3,dflow, dist)

tempo<-lm(vel~rain3+dflow+dist,data=alldata)

anova(tempo)

independence.test1<-lm(vel~rain3+dflow+dist+dayflow,data=alldata)

summary(independence.test1)

anova(independence.test1)

anova(tempo,independence.test1)

#210# d-separation (dayflow des res300| rain3,dflow,dist)

tempo<-lm(dayflow~rain3+dflow+dist,data=alldata)

anova(tempo)

independence.test1<-lm(dayflow~rain3+dflow+dist+res300,data=alldata)

summary(independence.test1)

anova(independence.test1)

anova(tempo,independence.test1)

#212# d-separation (cond des dayflow| rain3,dflow,alk, tp)

tempo<-lm(log(cond)~tp+alk+time+dist:time:eff+rain3+dflow,data=alldata[complete.cases(alldata[,c("cond","tp")]),])##final checked model5+revision

anova(tempo)

independence.test1<-lm(log(cond)~tp+alk+time+dist:time:eff+rain3+dflow+dayflow,data=alldata[complete.cases(alldata[,c("cond","tp")]),])##final checked model5+revision

summary(independence.test1)

anova(independence.test1)

anova(tempo,independence.test1)

#213# d-separation (zn des dayflow| rain3, dflow, eff)

tempo<-lm(log(zn)~rain3+dflow+eff,data=alldata)

anova(tempo)

independence.test1<-lm(log(zn)~rain3+dflow+eff+dayflow,data=alldata)

summary(independence.test1)

anova(independence.test1)

anova(tempo,independence.test1)

#214# d-separation (bugs des dayflow| rain3, dflow,temp, chla, toc, cond, zn)

wang.cap3<-capscale(formula=wangbug.BC~dayflow+Condition(temp+cond+temp:cond+toc+temp:toc+log(chla)+log(zn)+rain3+dflow),data=alldata,comm=wangbug,add=TRUE,na.action=na.omit)## condition (without cod and temp:zn, including cr)# ##final checked model5+revision

summary(wang.cap3)

wang.anova3<-anova(wang.cap3,by="term",permutations = how(nperm=9999))

print(wang.anova3)

#edit(alldata)

logchla<-log(alldata$chla)

datalogchla<-cbind(logchla,alldata)

datalogchla1<-datalogchla[complete.cases(datalogchla[,c("temp","cond","toc","chla")]),]#

design<-scale(model.matrix(~temp+cond+temp:cond+toc+temp:toc+log(chla)+rain3+log(zn)+rain3+dflow,data=alldata),center=FALSE,scale=FALSE)

#edit(design)

wangbug1<-wangbug[9:40,]

wangbug1.BC<-vegdist(sqrt(wangbug1))

n<-dim(wangbug1)[1]

p<-n-1

wangbug1.mds<-cmdscale(wangbug1.BC, k = p, eig = TRUE, add = TRUE, x.ret = FALSE)

#edit(wangbug1)

pco.predict<-qr.fitted(qr(design),wangbug1.mds$points)

pco.resid<-wangbug1.mds$points-pco.predict##compute pco residuals

plot(datalogchla1$ph2, pco.resid[,1],type="p", xlab="dayflow", ylab="pco1 residuals")

xyplot(pco.resid[,1]~datalogchla1$dayflow, xlab="dayflow", ylab="pco1 residuals")

independence.check<-lm(pco.resid[,1]~datalogchla1$dayflow, data=alldata)

summary(independence.check)

anova(independence.check)

#273#(turb des dist| rain3, dayflow, vel )

tempo<-lm(log(turb)~log(rain3)+log(dayflow)+log(vel),data=alldata)##final checked model5+revision

anova(tempo)

independence.test1<-lm(log(turb)~log(rain3)+log(dayflow)+log(vel)+dist,data=alldata)##final checked model5+revision

summary(independence.test1)

anova(independence.test1)

anova(tempo,independence.test1)

#274# d-separation (temp des dist| airtemp, rain3, dayflow, res300)

tempo<-lm(temp~rain3+airtemp+dayflow+res300+dayflow:res300+rain3:res300+dayflow:res300:rain3,data=alldata)

anova(tempo)

independence.test1<-lm(temp~rain3+airtemp+dayflow+res300+dayflow:res300+rain3:res300+dayflow:res300:rain3+dist,data=alldata)

summary(independence.test1)

anova(independence.test1)

anova(tempo,independence.test1)

#275# d-separation (tp des dist| solar, dayflow, dchla, turb, temp, eff, tp, no3)

tempo<-lm(tp~alk+ph2+rain2+rain3+eff:I(dtp/dayflow):time+eff:I(dtp/dayflow):I(dist==4.08):time,data=alldata[complete.cases(alldata[,c("tp")]),])

anova(tempo)

independence.test1<-lm(tp~alk+ph2+rain2+rain3+eff:I(dtp/dayflow):time+eff:I(dtp/dayflow):I(dist==4.08):time+dist,data=alldata[complete.cases(alldata[,c("tp")]),])

summary(independence.test1)

anova(independence.test1)

anova(tempo,independence.test1)

#276# d-separation (chla des dist| solar, dayflow, dchla, turb, temp, eff, tp, no3)

tempo<-lm(log(chla)~solar+temp+tp+no3+turb+solar:temp+eff:log(I(dchla/dayflow)),data=alldata[complete.cases(alldata[,c("temp","no3","tp")]),])###final checked model+revision

anova(tempo)

independence.test1<-lm(log(chla)~solar+temp+tp+no3+turb+solar:temp+eff:log(I(dchla/dayflow))+dist,data=alldata[complete.cases(alldata[,c("temp","no3","tp")]),])##final checked model+revision

summary(independence.test1)

anova(independence.test1)

anova(tempo,independence.test1)

#277# d-separation (zn des dist| eff)

tempo<-lm(log(zn)~eff,data=alldata)

anova(tempo)

independence.test1<-lm(log(zn)~dist+eff+dayflow,data=alldata)

summary(independence.test1)

anova(independence.test1)

anova(tempo,independence.test1)

#278# d-separation (bugs des dist| temp, chla, toc, cond, zn)

wang.cap3<-capscale(formula=wangbug.BC~dist+Condition(temp+cond+temp:cond+toc+temp:toc+log(chla))+log(zn),data=alldata,comm=wangbug,add=TRUE,na.action=na.omit)#

summary(wang.cap3)

wang.anova3<-anova(wang.cap3,by="term",permutations = how(nperm=9999))

print(wang.anova3)

anova(wang.cap3)

logchla<-log(alldata$chla)

datalogchla<-cbind(logchla,alldata)

datalogchla1<-datalogchla[complete.cases(datalogchla[,c("temp","cond","toc","chla")]),]#

design<-scale(model.matrix(~temp+cond+temp:cond+toc+temp:toc+log(chla)+log(zn),data=alldata),center=FALSE,scale=FALSE)

#edit(design)

wangbug1<-wangbug[9:40,]

wangbug1.BC<-vegdist(sqrt(wangbug1))

n<-dim(wangbug1)[1]

p<-n-1

wangbug1.mds<-cmdscale(wangbug1.BC, k = p, eig = TRUE, add = TRUE, x.ret = FALSE)

#edit(wangbug1)

pco.predict<-qr.fitted(qr(design),wangbug1.mds$points)

pco.resid<-wangbug1.mds$points-pco.predict##compute pco residuals

plot(datalogchla1$dist, pco.resid[,1],type="p", xlab="dist", ylab="pco1 residuals")

xyplot(pco.resid[,1]~datalogchla1$dist, xlab="dist", ylab="pco1 residuals")

independence.check<-lm(pco.resid[,1]~datalogchla1$dist, data=alldata)

summary(independence.check)

anova(independence.check)

#279# d-separation (vel des res300| dist)

tempo<-lm(vel~dist,data=alldata)

anova(tempo)

independence.test1<-lm(vel~res300+dist+dayflow,data=alldata)

summary(independence.test1)

anova(independence.test1)

anova(tempo,independence.test1)

#280# d-separation (vel des temp| airtemp, rain3, dayflow, res300, dist)

tempo<-lm(temp~rain3+airtemp+dayflow+res300+dayflow:res300+rain3:res300+dayflow:res300:rain3+dist,data=alldata)

anova(tempo)

independence.test1<-lm(temp~rain3+airtemp+dayflow+res300+dayflow:res300+rain3:res300+dayflow:res300:rain3+dist+vel,data=alldata)

summary(independence.test1)

anova(independence.test1)

anova(tempo,independence.test1)

#282# d-separation (alk des vel| rain3,time,dalk,dayflow,dist,eff)

tempo<-lm(log(alk)~rain3+dayflow+eff:I(log(dalk/dayflow)):time+eff:I(log(dalk/dayflow)):I(dist==4.08):time+dist,data=alldata) ###final checked model5+revision

anova(tempo)

independence.test1<-lm(log(alk)~rain3+dayflow+eff:I(log(dalk/dayflow)):time+eff:I(log(dalk/dayflow)):I(dist==4.08):time+dist+vel,data=alldata)##final checked model5+revision

summary(independence.test1)

anova(independence.test1)

anova(tempo,independence.test1)

#283# d-separation (tp des vel| dist, time, rain3, rain2, ph2, dayflow, dtp, eff, alk)

tempo<-lm(tp~alk+ph2+rain2+rain3+eff:I(dtp/dayflow):time+eff:I(dtp/dayflow):I(dist==4.08):time+dist,data=alldata[complete.cases(alldata[,c("tp")]),])

anova(tempo)

independence.test1<-lm(tp~alk+ph2+rain2+rain3+eff:I(dtp/dayflow):time+eff:I(dtp/dayflow):I(dist==4.08):time+dist+vel,data=alldata[complete.cases(alldata[,c("tp")]),])##final checked model5+revision

summary(independence.test1)

anova(independence.test1)

anova(tempo,independence.test1)

#284# d-separation (cond des vel| alk,tp,dist)

tempo<-lm(log(cond)~tp+alk+time+dist:time:eff+dist,data=alldata[complete.cases(alldata[,c("cond")]),])##final checked model5+revision

anova(tempo)

independence.test1<-lm(log(cond)~tp+alk+time+dist:time:eff+dist+vel,data=alldata[complete.cases(alldata[,c("cond")]),])##final checked model5+revision

summary(independence.test1)

anova(independence.test1)

anova(tempo,independence.test1)

#285# d-separation (no3 des vel| rain2,rain3,time,dno3,dayflow,dist,eff, temp)

tempo<-lm(log(no3)~temp+rain2+rain3+eff:I(log(dno3)/dayflow):time+eff:I(log(dno3)/dayflow):I(dist==4.08):time+dist,data=alldata)##final checked model5+revision

independence.test1<-lm(log(no3)~temp+rain2+rain3+eff:I(log(dno3)/dayflow):time+eff:I(log(dno3)/dayflow):I(dist==4.08):time+dist+vel,data=alldata)##final checked model5+revision

summary(independence.test1)

anova(independence.test1)

anova(tempo,independence.test1)

#286# d-separation (chla des vel| solar, dayflow, dchla, turb, temp, eff, tp, no3, dist)

tempo<-lm(log(chla)~solar+temp+tp+no3+turb+solar:temp+eff:log(I(dchla/dayflow))+dist,data=alldata[complete.cases(alldata[,c("temp","no3","tp")]),])##final checked model+revision

anova(tempo)

independence.test1<-lm(log(chla)~solar+temp+tp+no3+turb+solar:temp+eff:log(I(dchla/dayflow))+dist+vel,data=alldata[complete.cases(alldata[,c("temp","no3","tp")]),])##final checked model5+revision

summary(independence.test1)

anova(independence.test1)

anova(tempo,independence.test1)

#287# d-separation (zn des vel| dist, eff)

tempo<-lm(log(zn1)~dist+eff,data=alldata)

independence.test1<-lm(log(zn1)~dist+eff+vel,data=alldata)

summary(independence.test1)

anova(independence.test1)

anova(tempo,independence.test1)

#288# d-separation (toc des vel| rain2,rain3, time, dist, dayflow, dtoc, eff)

tempo<-lm(log(toc)~dayflow+rain2+rain3+eff:I(log(dtoc/dayflow)):time+eff:I(log(dtoc/dayflow)):I(dist==4.08):time+dist,data=alldata)##final checked model5+revision

independence.test1<-lm(log(toc)~dayflow+rain2+rain3+eff:I(log(dtoc/dayflow)):time+eff:I(log(dtoc/dayflow)):I(dist==4.08):time+dist+vel,data=alldata)##final checked model5+revision

summary(independence.test1)

anova(independence.test1)

anova(tempo,independence.test1)

#289# d-separation (bugs des vel| temp, chla, toc, cond, zn, dist)

wang.cap3<-capscale(formula=wangbug.BC~vel+Condition(temp+cond+temp:cond+toc+temp:toc+log(chla))+log(zn)+dist,data=alldata,comm=wangbug,add=TRUE,na.action=na.omit)#

summary(wang.cap3)

wang.anova3<-anova(wang.cap3,by="term",permutations = how(nperm=9999))

print(wang.anova3)

logchla<-log(alldata$chla)

datalogchla<-cbind(logchla,alldata)

datalogchla1<-datalogchla[complete.cases(datalogchla[,c("temp","cond","toc","chla")]),]#

design<-scale(model.matrix(~temp+cond+temp:cond+toc+temp:toc+log(chla)+log(zn)+dist,data=alldata),center=FALSE,scale=FALSE)

#edit(design)

wangbug1<-wangbug[9:40,]

wangbug1.BC<-vegdist(sqrt(wangbug1))

n<-dim(wangbug1)[1]

p<-n-1

wangbug1.mds<-cmdscale(wangbug1.BC, k = p, eig = TRUE, add = TRUE, x.ret = FALSE)

#edit(wangbug1)

pco.predict<-qr.fitted(qr(design),wangbug1.mds$points)

pco.resid<-wangbug1.mds$points-pco.predict##compute pco residuals

plot(datalogchla1$dist, pco.resid[,1],type="p", xlab="vel", ylab="pco1 residuals")

xyplot(pco.resid[,1]~datalogchla1$vel, xlab="vel", ylab="pco1 residuals")

independence.check<-lm(pco.resid[,1]~datalogchla1$vel, data=alldata)

summary(independence.check)

anova(independence.check)

#290# d-separation (turb des res300| rain3, dayflow, vel, dist)

tempo<-lm(log(turb)~log(rain3)+log(dayflow)+log(vel)+dist,data=alldata)###final checked model5+revision

anova(tempo)

independence.test1<-lm(log(turb)~log(rain3)+log(dayflow)+log(vel)+dist+res300,data=alldata)##final checked model5+revision

summary(independence.test1)

anova(independence.test1)

anova(tempo,independence.test1)

#291# d-separation (turb des temp| rain3, dayflow, vel, airtemp, res300)

tempo<-lm(log(turb)~log(rain3)+log(dayflow)+log(vel)+airtemp+res300,data=alldata)##final checked model5+revision

anova(tempo)

independence.test1<-lm(log(turb)~log(rain3)+log(dayflow)+log(vel)+airtemp+res300+temp,data=alldata)##final checked model5+revision

summary(independence.test1)

anova(independence.test1)

anova(tempo,independence.test1)

#293# d-separation (alk des turb| rain3, dayflow, vel, time, dalk, dist, eff)

tempo<-lm(log(alk)~rain3+dayflow+eff:I(log(dalk/dayflow)):time+eff:I(log(dalk/dayflow)):I(dist==4.08):time+dist+vel,data=alldata) ##final checked model5+revision

anova(tempo)

independence.test1<-lm(log(alk)~rain3+dayflow+eff:I(log(dalk/dayflow)):time+eff:I(log(dalk/dayflow)):I(dist==4.08):time+dist+vel+turb,data=alldata) ##final checked model5+revision

summary(independence.test1)

anova(independence.test1)

anova(tempo,independence.test1)

#294# d-separation (tp des turb| rain3, dayflow, vel, time, rain2, ph2, dist, dtp, eff, alk)

tempo<-lm(tp~alk+ph2+rain2+rain3+eff:I(log(dtp/dayflow)):time+eff:I(log(dtp/dayflow)):I(dist==4.08):time+dist+vel,data=alldata[complete.cases(alldata[,c("tp")]),])##final checked model5+revision

anova(tempo)

independence.test1<-lm(tp~alk+ph2+rain2+rain3+eff:I(log(dtp/dayflow)):time+eff:I(log(dtp/dayflow)):I(dist==4.08):time+dist+vel+turb,data=alldata[complete.cases(alldata[,c("tp")]),])##final checked model5+revision

summary(independence.test1)

anova(independence.test1)

anova(tempo,independence.test1)

#295# d-separation (cond des turb| rain3, dayflow, vel, alk, tp)

tempo<-lm(log(cond)~tp+alk+time+dist:time:eff+vel+rain3+dayflow,data=alldata[complete.cases(alldata[,c("cond")]),])##final checked model5+revision

anova(tempo)

independence.test1<-lm(log(cond)~tp+alk+time+dist:time:eff+vel+rain3+dayflow+turb,data=alldata[complete.cases(alldata[,c("cond")]),])##final checked model5+revision

summary(independence.test1)

anova(independence.test1)

anova(tempo,independence.test1)

#296# d-separation (no3 des turb| rain3, dayflow, vel, time, rain2, dno3, dist, temp, eff)

tempo<-lm(log(no3)~temp+rain2+rain3+eff:I(log(dno3)/dayflow):time+eff:I(log(dno3)/dayflow):I(dist==4.08):time+dist,data=alldata)##final checked model5+revision

anova(tempo)

independence.test1<-lm(log(no3)~temp+rain2+rain3+eff:I(log(dno3)/dayflow):time+eff:I(log(dno3)/dayflow):I(dist==4.08):time+dist+turb,data=alldata)##final checked model5+revision

summary(independence.test1)

anova(independence.test1)

anova(tempo,independence.test1)

#297# d-separation (zn des turb| rain3, dayflow, vel, eff)

tempo<-lm(log(zn)~rain3+dayflow+vel+eff,data=alldata)

independence.test1<-lm(log(zn)~rain3+dayflow+vel+eff+turb,data=alldata)

summary(independence.test1)

anova(independence.test1)

anova(tempo,independence.test1)

#298# d-separation (toc des turb| dayflow, rain3, vel, time, rain2, dtoc, dist, eff)

tempo<-lm((toc)~dayflow+rain2+rain3+eff:I((dtoc/dayflow)):time+eff:I((dtoc/dayflow)):I(dist==4.08):time+dist+vel,data=alldata)##final checked model5+revision

anova(tempo)

independence.test1<-lm((toc)~dayflow+rain2+rain3+eff:I((dtoc/dayflow)):time+eff:I((dtoc/dayflow)):I(dist==4.08):time+dist+vel+turb,data=alldata)##final checked model5+revision

summary(independence.test1)

anova(independence.test1)

anova(tempo,independence.test1)

#299# d-separation (bugs des turb| rain3, dayflow, vel, temp, chla, toc, cond, zn)

wang.cap3<-capscale(formula=wangbug.BC~turb+Condition(temp+cond+temp:cond+toc+temp:toc+log(chla)+log(zn)+rain3+dayflow+vel),data=alldata,comm=wangbug,add=TRUE,na.action=na.omit)## condition (without cod and temp:zn, including cr) final model ##final checked model5+revision

summary(wang.cap3)

wang.anova3<-anova(wang.cap3,by="term",permutations = how(nperm=9999))

print(wang.anova3)

#edit(alldata)

logchla<-log(alldata$chla)

datalogchla<-cbind(logchla,alldata)

datalogchla1<-datalogchla[complete.cases(datalogchla[,c("temp","cond","toc","chla")]),]#

design<-scale(model.matrix(~temp+cond+temp:cond+toc+temp:toc+log(chla)+rain3+log(zn)+rain3+dflow+vel,data=alldata),center=FALSE,scale=FALSE)

#edit(design)

wangbug1<-wangbug[9:40,]

wangbug1.BC<-vegdist(sqrt(wangbug1))

n<-dim(wangbug1)[1]

p<-n-1

wangbug1.mds<-cmdscale(wangbug1.BC, k = p, eig = TRUE, add = TRUE, x.ret = FALSE)

#edit(wangbug1)

pco.predict<-qr.fitted(qr(design),wangbug1.mds$points)

pco.resid<-wangbug1.mds$points-pco.predict##compute pco residuals

plot(datalogchla1$turb, pco.resid[,1],type="p", xlab="turb", ylab="pco1 residuals")

xyplot(pco.resid[,1]~datalogchla1$turb, xlab="turb", ylab="pco1 residuals")

independence.check<-lm(pco.resid[,1]~datalogchla1$turb, data=alldata)

summary(independence.check)

anova(independence.check)

#301# d-separation (alk des res300| dist, time, rain, dalk, dayflow, eff)

tempo<-lm(log(alk)~rain3+dayflow+eff:I(log(dalk/dayflow)):time+eff:I(log(dalk/dayflow)):I(dist==4.08):time+dist,data=alldata) ###final checked model5+revision

anova(tempo)

independence.test1<-lm(log(alk)~rain3+dayflow+eff:I(log(dalk/dayflow)):time+eff:I(log(dalk/dayflow)):I(dist==4.08):time+dist+res300,data=alldata) ##final checked model5+revision

summary(independence.test1)

anova(independence.test1)

anova(tempo,independence.test1)

#302# d-separation (tp des res300| dist, time, dtp, dayflow, rain2, rain3, ph2, eff, alk)

tempo<-lm(tp~alk+ph2+rain2+rain3+eff:I(dtp/dayflow):time+eff:I(dtp/dayflow):I(dist==4.08):time+dist,data=alldata[complete.cases(alldata[,c("tp")]),])

anova(tempo)

independence.test1<-lm(tp~alk+ph2+rain2+rain3+eff:I(dtp/dayflow):time+eff:I(dtp/dayflow):I(dist==4.08):time+dist+res300,data=alldata[complete.cases(alldata[,c("tp")]),])###final checked model5+revision

summary(independence.test1)

anova(independence.test1)

anova(tempo,independence.test1)

#303# d-separation (cond des res300| dist, alk, tp)

tempo<-lm(log(cond)~tp+alk+time+dist:time:eff+dist,data=alldata[complete.cases(alldata[,c("cond")]),])##final checked model5+revision

anova(tempo)

independence.test1<-lm(log(cond)~tp+alk+time+dist:time:eff+dist+res300,data=alldata[complete.cases(alldata[,c("cond")]),])##final checked model5+revision

summary(independence.test1)

anova(independence.test1)

anova(tempo,independence.test1)

#304# d-separation (no3 des res300| dist, time, dno3, rain2, rain3, dayflow, temp, eff)

tempo<-lm(log(no3)~temp+rain2+rain3+eff:I(log(dno3)/dayflow):time+eff:I(log(dno3)/dayflow):I(dist==4.08):time+dist,data=alldata)##final checked model5+revision

anova(tempo)

independence.test1<-lm(log(no3)~temp+rain2+rain3+eff:I(log(dno3)/dayflow):time+eff:I(log(dno3)/dayflow):I(dist==4.08):time+dist+res300,data=alldata)##final checked model5+revision

summary(independence.test1)

anova(independence.test1)

anova(tempo,independence.test1)

#305# d-separation (chla des res300| dist, solar, dayflow, dchla, turb, temp, eff, tp, no3)

tempo<-lm(log(chla)~solar+temp+tp+no3+turb+solar:temp+eff:log(I(dchla/dayflow))+dist,data=alldata[complete.cases(alldata[,c("temp","no3","tp")]),])##final checked model+revision

anova(tempo)

independence.test1<-lm(log(chla)~solar+temp+tp+no3+turb+solar:temp+eff:log(I(dchla/dayflow))+dist+res300,data=alldata[complete.cases(alldata[,c("temp","no3","tp")]),])##final checked model+revision

anova(tempo)

summary(independence.test1)

anova(independence.test1)

anova(tempo,independence.test1)

#306# d-separation (zn des res300| dist, eff)

tempo<-lm(log(zn1)~ dist+eff,data=alldata)

independence.test1<-lm(log(zn1)~ dist+eff+res300, data=alldata)

summary(independence.test1)

anova(independence.test1)

anova(tempo,independence.test1)

#307# d-separation (toc des res300| dayflow, rain3, time, rain2, dtoc, dist, eff)

tempo<-lm((toc)~dayflow+rain2+rain3+eff:I((dtoc/dayflow)):time+eff:I((dtoc/dayflow)):I(dist==4.08):time+dist,data=alldata)##final checked model5+revision

anova(tempo)

independence.test1<-lm((toc)~dayflow+rain2+rain3+eff:I((dtoc/dayflow)):time+eff:I((dtoc/dayflow)):I(dist==4.08):time+dist+res300,data=alldata)##final checked model5+revision

summary(independence.test1)

anova(independence.test1)

anova(tempo,independence.test1)

#308# d-separation (bugs des res300| dist, temp, toc, cond, chla, zn)

wang.cap3<-capscale(formula=wangbug.BC~res300+Condition(temp+cond+temp:cond+toc+temp:toc+log(chla)+log(zn)+dist),data=wangenv,comm=wangbug,add=TRUE,na.action=na.omit)#

summary(wang.cap3)

wang.anova3<-anova(wang.cap3,by="term",permutations = how(nperm=9999))

print(wang.anova3)

anova(wang.cap3)

summary(wang.cap3)

logchla<-log(alldata$chla)

datalogchla<-cbind(logchla,alldata)

datalogchla1<-datalogchla[complete.cases(datalogchla[,c("temp","cond","toc","chla")]),]#

design<-scale(model.matrix(~temp+cond+temp:cond+toc+temp:toc+log(chla)+log(zn)+dist,data=wangenv),center=FALSE,scale=FALSE)

#edit(design)

wangbug1<-wangbug[9:40,]

wangbug1.BC<-vegdist(sqrt(wangbug1))

n<-dim(wangbug1)[1]

p<-n-1

wangbug1.mds<-cmdscale(wangbug1.BC, k = p, eig = TRUE, add = TRUE, x.ret = FALSE)

#edit(wangbug1)

pco.predict<-qr.fitted(qr(design),wangbug1.mds$points)

pco.resid<-wangbug1.mds$points-pco.predict##compute pco residuals

plot(datalogchla1$res300, pco.resid[,1],type="p", xlab="dist", ylab="pco1 residuals")

xyplot(pco.resid[,1]~datalogchla1$res300, xlab="res300", ylab="pco1 residuals")

independence.check<-lm(pco.resid[,1]~datalogchla1$res300, data=alldata)

summary(independence.check)

anova(independence.check)

#310# d-separation (alk des temp| airtemp,rain3, dayflow, res300, time, dalk, dist, eff)

tempo<-lm(log(alk)~rain3+dayflow+eff:I(log(dalk/dayflow)):time+eff:I(log(dalk/dayflow)):I(dist==4.08):time+dist+res300,data=alldata) ###final checked model5+revision

anova(tempo)

independence.test1<-lm(log(alk)~rain3+dayflow+eff:I(log(dalk/dayflow)):time+eff:I(log(dalk/dayflow)):I(dist==4.08):time+dist+res300+temp,data=alldata) ##final checked model5+revision

summary(independence.test1)

anova(independence.test1)

anova(tempo,independence.test1)

#311# d-separation (tp des temp| airtemp, rain3, dayflow, res300, time, rain2, ph2, dtp, eff, alk, dist)

tempo<-lm(tp~alk+ph2+rain2+rain3+eff:I(dtp/dayflow):time+eff:I(dtp/dayflow):I(dist==4.08):time+dist+airtemp,data=alldata[complete.cases(alldata[,c("tp")]),])

anova(tempo)

independence.test1<-lm(tp~alk+ph2+rain2+rain3+eff:I(dtp/dayflow):time+eff:I(dtp/dayflow):I(dist==4.08):time+dist+airtemp+temp,data=alldata[complete.cases(alldata[,c("tp")]),])

summary(independence.test1)

anova(independence.test1)

anova(tempo,independence.test1)

#312# d-separation (cond des temp| airtemp, rain3, dayflow, res300, alk, tp)

tempo<-lm(log(cond)~tp+alk+time+dist:time:eff+airtemp+rain3+dayflow+res300,data=alldata[complete.cases(alldata[,c("cond")]),])##final checked model5+revision

anova(tempo)

independence.test1<-lm(log(cond)~tp+alk+time+dist:time:eff+airtemp+rain3+dayflow+res300+temp,data=alldata[complete.cases(alldata[,c("cond")]),])##final checked model5+revision

summary(independence.test1)

anova(independence.test1)

anova(tempo,independence.test1)

#313# d-separation (zn des temp| airtemp, rain3, dayflow, res300, dist, eff)

tempo<-lm(log(zn1)~airtemp+rain3+dayflow+res300+dist+eff,data=alldata)

independence.test1<-lm(log(zn1)~airtemp+rain3+dayflow+res300+dist+eff+temp,data=alldata)

summary(independence.test1)

anova(independence.test1)

anova(tempo,independence.test1)

#314# d-separation (toc des temp| airtemp, dayflow, rain3, res300, time, rain2, dtoc, dist, eff)

tempo<-lm((toc)~dayflow+rain2+rain3+eff:I((dtoc/dayflow)):time+eff:I((dtoc/dayflow)):I(dist==4.08):time+dist+airtemp+res300,data=alldata)##final checked model5+revision

anova(tempo)

independence.test1<-lm((toc)~dayflow+rain2+rain3+eff:I((dtoc/dayflow)):time+eff:I((dtoc/dayflow)):I(dist==4.08):time+dist+airtemp+res300+temp,data=alldata)##final checked model5+revision

summary(independence.test1)

anova(independence.test1)

anova(tempo,independence.test1)

#316# d-separation (alk des no3| time, dno3, dayflow, dist, temp, eff, rain3, rain2, dalk)

tempo<-lm(log(alk)~rain3+dayflow+eff:I(log(dalk/dayflow)):time+eff:I(log(dalk/dayflow)):I(dist==4.08):time+dist+dno3+temp+rain2,data=alldata) ##final checked model5+revision

anova(tempo)

independence.test1<-lm(log(alk)~rain3+dayflow+eff:I(log(dalk/dayflow)):time+eff:I(log(dalk/dayflow)):I(dist==4.08):time+dist+dno3+temp+rain2+no3,data=alldata) ##final checked model5+revision

summary(independence.test1)

anova(independence.test1)

anova(tempo,independence.test1)

#317# d-separation (chla des alk| time, rain3, dalk, dayflow, dist, eff, solar, dchla, turb, temp, tp, no3)

tempo<-lm(log(chla)~solar+temp+tp+no3+turb+solar:temp+eff:log(I(dchla/dayflow))+dist+time+rain3+dalk,data=alldata[complete.cases(alldata[,c("temp","no3","tp")]),])##final checked model+revision

anova(tempo)

independence.test1<-lm(log(chla)~solar+temp+tp+no3+turb+solar:temp+eff:log(I(dchla/dayflow))+dist+time+rain3+dalk+alk,data=alldata[complete.cases(alldata[,c("temp","no3","tp")]),])##final checked model+revision

anova(tempo)

summary(independence.test1)

anova(independence.test1)

anova(tempo,independence.test1)

#318# d-separation (zn des alk| time, rain3, dalk, dayflow, dist, eff)

tempo<-lm(log(zn1)~airtemp+rain3+dayflow+res300+dist+eff,data=alldata)

anova(tempo)

independence.test1<-lm(log(zn1)~airtemp+rain3+dayflow+res300+dist+eff+alk,data=alldata)

summary(independence.test1)

anova(independence.test1)

anova(tempo,independence.test1)

#319# d-separation (toc des alk| rain3, rain2, time, dalk, dayflow, dtoc, dist, eff)

tempo<-lm((toc)~dayflow+rain2+rain3+eff:I((dtoc/dayflow)):time+eff:I((dtoc/dayflow)):I(dist==4.08):time+dist+dalk,data=alldata)##final checked model5+revision

anova(tempo)

independence.test1<-lm((toc)~dayflow+rain2+rain3+eff:I((dtoc/dayflow)):time+eff:I((dtoc/dayflow)):I(dist==4.08):time+dist+dalk+alk,data=alldata)##final checked model5+revision

summary(independence.test1)

anova(independence.test1)

anova(tempo,independence.test1)

#320# d-separation (bugs des alk| time, rain3, dalk, dayflow, dist, eff, temp, zn, cond, chla)

wang.cap3<-capscale(formula=wangbug.BC~alk+Condition(temp+cond+temp:cond+toc+temp:toc+log(chla)+eff+dist+dayflow+dalk+rain3+log(zn)),data=alldata,comm=wangbug,add=TRUE,na.action=na.omit)#

summary(wang.cap3)

wang.anova3<-anova(wang.cap3,by="term",permutations = how(nperm=9999))

print(wang.anova3)

anova(wang.cap3)

summary(wang.cap3)

logchla<-log(alldata$chla)

datalogchla<-cbind(logchla,alldata)

datalogchla1<-datalogchla[complete.cases(datalogchla[,c("temp","cond","toc","chla")]),]#

design<-scale(model.matrix(~temp+cond+temp:cond+toc+temp:toc+log(chla)+eff+dist+dayflow+dalk+rain3+log(zn),data=wangenv),center=FALSE,scale=FALSE)

#edit(design)

wangbug1<-wangbug[9:40,]

wangbug1.BC<-vegdist(sqrt(wangbug1))

n<-dim(wangbug1)[1]

p<-n-1

wangbug1.mds<-cmdscale(wangbug1.BC, k = p, eig = TRUE, add = TRUE, x.ret = FALSE)

#edit(wangbug1)

pco.predict<-qr.fitted(qr(design),wangbug1.mds$points)

pco.resid<-wangbug1.mds$points-pco.predict##compute pco residuals

plot(datalogchla1$alk, pco.resid[,1],type="p", xlab="alk", ylab="pco1 residuals")

xyplot(pco.resid[,1]~datalogchla1$alk, xlab="alk", ylab="pco1 residuals")

independence.check<-lm(pco.resid[,1]~datalogchla1$alk, data=alldata)

summary(independence.check)

anova(independence.check)

#321# d-separation (no3 des tp| time, rain3, rain2, ph2, dno3, dayflow, dist, temp, eff, dtp, alk)

tempo<-lm(log(no3)~temp+ph2+rain2+rain3+eff:I(log(dno3)/dayflow):time+eff:I(log(dno3)/dayflow):I(dist==4.08):time+dist+dtp+alk,data=alldata[complete.cases(alldata[,c("tp")]),])##final checked model5+revision

anova(tempo)

independence.test1<-lm(log(no3)~temp+rain2+rain3+eff:I(log(dno3)/dayflow):time+eff:I(log(dno3)/dayflow):I(dist==4.08):time+dist+dtp+alk+tp,data=alldata[complete.cases(alldata[,c("tp")]),])##final checked model5+revision

summary(independence.test1)

anova(independence.test1)

anova(tempo,independence.test1)

#322# d-separation (tp des zn | time, rain3, rain2, ph2, dayflow, dtp, dist, eff, alk)

tempo<-lm(tp~alk+ph2+rain2+rain3+eff:I(dtp/dayflow):time+eff:I(dtp/dayflow):I(dist==4.08):time+dist,data=alldata[complete.cases(alldata[,c("tp")]),])

anova(tempo)

independence.test1<-lm(tp~alk+ph2+rain2+rain3+eff:I(log(dtp/dayflow)):time+eff:I(log(dtp/dayflow)):I(dist==4.08):time+dist+zn,data=alldata[complete.cases(alldata[,c("tp")]),])##final checked model5+revision

summary(independence.test1)

anova(independence.test1)

anova(tempo,independence.test1)

#323# d-separation (toc des tp| time, rain3, rain2, ph2, dayflow, dtoc, dist, eff, dtp, alk)

tempo<-lm((toc)~dayflow+rain2+rain3+eff:I((dtoc/dayflow)):time+eff:I((dtoc/dayflow)):I(dist==4.08):time+dist+ph2+alk,data=alldata[complete.cases(alldata[,c("tp")]),])##final checked model5+revision

anova(tempo)

independence.test1<-lm((toc)~dayflow+rain2+rain3+eff:I((dtoc/dayflow)):time+eff:I((dtoc/dayflow)):I(dist==4.08):time+dist+ph2+alk+tp,data=alldata[complete.cases(alldata[,c("tp")]),])##final checked model5+revision

summary(independence.test1)

anova(independence.test1)

anova(tempo,independence.test1)

#324# d-separation (bugs des tp | time, rain3, rain2, ph2, dtp, dayflow, eff, alk, temp, dist, chla, toc, cond, zn)

wang.cap3<-capscale(formula=wangbug.BC~tp+Condition(temp+cond+temp:cond+toc+temp:toc+log(chla)+log(zn1)+time+rain3+rain2+ph2+dtp+dayflow+eff+alk+dist),data=alldata,comm=wangbug,add=TRUE,na.action=na.omit)#

summary(wang.cap3)

wang.anova3<-anova(wang.cap3,by="term",permutations = how(nperm=9999))

print(wang.anova3)

anova(wang.cap3)

summary(wang.cap3)

logchla<-log(alldata$chla)

datalogchla<-cbind(logchla,alldata)

datalogchla1<-datalogchla[complete.cases(datalogchla[,c("temp","cond","toc","chla")]),]#

design<-scale(model.matrix(~temp+cond+temp:cond+toc+temp:toc+log(chla)+log(zn1)+time+rain3+rain2+ph2+dtp+dayflow+eff+alk+dist,data=alldata),center=FALSE,scale=FALSE)

#edit(design)

wangbug1<-wangbug[9:40,]

wangbug1.BC<-vegdist(sqrt(wangbug1))

n<-dim(wangbug1)[1]

p<-n-1

wangbug1.mds<-cmdscale(wangbug1.BC, k = p, eig = TRUE, add = TRUE, x.ret = FALSE)

#edit(wangbug1)

pco.predict<-qr.fitted(qr(design),wangbug1.mds$points)

pco.resid<-wangbug1.mds$points-pco.predict##compute pco residuals

plot(datalogchla1$tp, pco.resid[,1],type="p", xlab="tp", ylab="pco1 residuals")

xyplot(pco.resid[,1]~datalogchla1$tp, xlab="tp", ylab="pco1 residuals")

independence.check<-lm(pco.resid[,1]~datalogchla1$tp, data=alldata)

summary(independence.check)

anova(independence.check)

#325# d-separation (no3 des cond| time, rain3, rain2, dno3, dayflow, dist, temp, eff, tp, alk)

tempo<-lm(log(no3)~temp+rain2+rain3+eff:I(log(dno3)/dayflow):time+eff:I(log(dno3)/dayflow):I(dist==4.08):time+alk+tp,data=alldata[complete.cases(alldata[,c("tp","cond")]),])##final checked model5+revision

anova(tempo)

independence.test1<-lm(log(no3)~temp+rain2+rain3+eff:I(log(dno3)/dayflow):time+eff:I(log(dno3)/dayflow):I(dist==4.08):time+alk+tp+cond,data=alldata[complete.cases(alldata[,c("tp","cond")]),])##final checked model5+revision

summary(independence.test1)

anova(independence.test1)

anova(tempo,independence.test1)

#326# d-separation (chla des cond | time, dayflow, eff, alk, tp, solar, dchla, turb, temp, no3)

tempo<-lm(log(chla)~solar+temp+tp+no3+turb+solar:temp+eff:log(I(dchla/dayflow))+time+rain3+alk,data=alldata[complete.cases(alldata[,c("temp","no3","tp","cond")]),])##final checked model+revision

anova(tempo)

independence.test1<-lm(log(chla)~solar+temp+tp+no3+turb+solar:temp+eff:log(I(dchla/dayflow))+time+rain3+alk+cond,data=alldata[complete.cases(alldata[,c("temp","no3","tp","cond")]),])##final checked model+revision

summary(independence.test1)

anova(independence.test1)

anova(tempo,independence.test1)

#327# d-separation (cond des zn | eff, alk, tp)

tempo<-lm(log(cond)~tp+alk+time+dist:time:eff,data=alldata[complete.cases(alldata[,c("cond")]),])##final checked model5+revision

anova(tempo)

independence.test1<-lm(log(cond)~tp+alk+time+dist:time:eff+zn,data=alldata[complete.cases(alldata[,c("cond")]),])##final checked model5+revision

summary(independence.test1)

anova(independence.test1)

anova(tempo,independence.test1)

#328# d-separation (toc des cond| time, rain3, rain2, dayflow, dtoc, dist, eff, alk, tp)

tempo<-lm(log(toc)~dayflow+rain2+rain3+eff:I(log(dtoc/dayflow)):time+eff:I(log(dtoc/dayflow)):I(dist==4.08):time+tp+alk,data=alldata[complete.cases(alldata[,c("tp","cond")]),])##final checked model5+revision

anova(tempo)

independence.test1<-lm(log(toc)~dayflow+rain2+rain3+eff:I(log(dtoc/dayflow)):time+eff:I(log(dtoc/dayflow)):I(dist==4.08):time+tp+alk+cond,data=alldata[complete.cases(alldata[,c("tp","cond")]),])##final checked model5+revision

summary(independence.test1)

anova(independence.test1)

anova(tempo,independence.test1)

#329# d-separation (zn des no3 | time, dno3, rain3, rain2, dayflow, dist, temp, eff)

tempo<-lm(log(zn1)~dist+eff+temp+time+dno3+rain3+rain2+dayflow,data=alldata)

anova(tempo)

independence.test1<-lm(log(zn1)~dist+eff+temp+time+dno3+rain3+rain2+dayflow+no3,data=alldata)

summary(independence.test1)

anova(independence.test1)

anova(tempo,independence.test1)

#330# d-separation (toc des no3| time, dno3, rain3, rain2, dayflow, dist, temp, eff, dtoc)

tempo<-lm((toc)~dayflow+rain2+rain3+eff:I((dtoc/dayflow)):time+eff:I((dtoc/dayflow)):I(dist==4.08):time+dist+dno3+temp+no3,data=alldata)###checked model5+revision

anova(tempo)

independence.test1<-lm((toc)~dayflow+rain2+rain3+eff:I((dtoc/dayflow)):time+eff:I((dtoc/dayflow)):I(dist==4.08):time+dist+dno3+temp,data=alldata)##checked model5+revision

summary(independence.test1)

anova(independence.test1)

anova(tempo,independence.test1)

#331# d-separation (bugs des no3| time, dno3, rain3, rain2, dayflow, dist, temp, eff, chla, toc, cond, zn)

wang.cap3<-capscale(formula=wangbug.BC~no3+Condition(temp+cond+temp:cond+toc+temp:toc+log(chla)+log(zn)+dist+time+dno3+dayflow+eff+rain3+rain2),data=alldata,comm=wangbug,add=TRUE,na.action=na.omit)#

summary(wang.cap3)

wang.anova3<-anova(wang.cap3,by="term",permutations = how(nperm=9999))

print(wang.anova3)

anova(wang.cap3)

summary(wang.cap3)

logchla<-log(alldata$chla)

datalogchla<-cbind(logchla,alldata)

datalogchla1<-datalogchla[complete.cases(datalogchla[,c("temp","cond","toc","chla")]),]#

design<-scale(model.matrix(~temp+cond+temp:cond+toc+temp:toc+log(chla)+log(zn)+dist+time+dno3+dayflow+eff+rain2+rain3,data=alldata),center=FALSE,scale=FALSE)

#edit(design)

wangbug1<-wangbug[9:40,]

wangbug1.BC<-vegdist(sqrt(wangbug1))

n<-dim(wangbug1)[1]

p<-n-1

wangbug1.mds<-cmdscale(wangbug1.BC, k = p, eig = TRUE, add = TRUE, x.ret = FALSE)

#edit(wangbug1)

pco.predict<-qr.fitted(qr(design),wangbug1.mds$points)

pco.resid<-wangbug1.mds$points-pco.predict##compute pco residuals

plot(datalogchla1$no3, pco.resid[,1],type="p", xlab="no3", ylab="pco1 residuals")

xyplot(pco.resid[,1]~datalogchla1$no3, xlab="no3", ylab="pco1 residuals")

independence.check<-lm(pco.resid[,1]~datalogchla1$no3, data=alldata)

summary(independence.check)

anova(independence.check)

#332# d-separation (chla des zn | eff, solar, dayflow, dchla, turb, temp, tp, no3)

tempo<-lm(log(chla)~solar+temp+tp+no3+turb+solar:temp+eff:log(I(dchla/dayflow)),data=alldata[complete.cases(alldata[,c("temp","no3","tp")]),])##final checked model+revision

anova(tempo)

independence.test1<-lm(log(chla)~solar+temp+tp+no3+turb+solar:temp+eff:log(I(dchla/dayflow))+zn,data=alldata[complete.cases(alldata[,c("temp","no3","tp")]),])##final checked model+revision

summary(independence.test1)

anova(independence.test1)

anova(tempo,independence.test1)

#333# d-separation (chla des toc| time, dayflow, dtoc, dist, eff, solar, dchla, turb, temp, no3, tp, rain2,rain3)

tempo<-lm(log(chla)~solar+temp+tp+no3+turb+solar:temp+eff:log(I(dchla/dayflow))+time+dtoc+dist+rain2+rain3,data=alldata[complete.cases(alldata[,c("temp","no3","tp")]),])##final checked model+revision

anova(tempo)

independence.test1<-lm(log(chla)~solar+temp+tp+no3+turb+solar:temp+eff:log(I(dchla/dayflow))+time+dtoc+dist+rain2+rain3+toc,data=alldata[complete.cases(alldata[,c("temp","no3","tp")]),])##final checked model+revision

summary(independence.test1)

anova(independence.test1)

anova(tempo,independence.test1)

#334# d-separation (toc des zn| dist, eff, time, dayflow, dtoc, rain2, rain3)

tempo<-lm((toc)~dayflow+rain2+rain3+eff:I((dtoc/dayflow)):time+eff:I((dtoc/dayflow)):I(dist==4.08):time+dist,data=alldata)##final checked model+revision

anova(tempo)

independence.test1<-lm((toc)~dayflow+rain2+rain3+eff:I((dtoc/dayflow)):time+eff:I((dtoc/dayflow)):I(dist==4.08):time+dist+zn,data=alldata)##final checked model+revision

summary(independence.test1)

anova(independence.test1)

anova(tempo,independence.test1)

##*************************************##

##*************************************##

C<--2*(log(0.2862)+log(0.2862)+log(0.281)+log(0.4666)+log(0.2584)+log(0.729)+log(0.9811)+log(0.9801)+log(0.8075)+log(0.08321)+log(0.768)+log(0.7981)+log(0.3135)+log(0.4091)+log(0.3185)+log(0.4001)+log(0.8362)+log(0.9707)+log(0.8159)+log(0.3239)+log(0.9299)+log(0.3545)+log(0.868)+log(0.1587)+log(0.1848)+log(0.859)+log(0.3215)+log(0.9016)+log(0.2587)+log(0.6589)+log(0.9717)+log(0.2118)+log(0.2745)+log(0.2654)+log(0.5033)+log(0.5644)+log(0.3253)+log(0.4007)+log(0.3457)+log(0.2598)+log(0.7497)+log(0.07954)+log(0.5078)+log(0.1927)+log(0.01745)+log(0.9612)+log(0.001129)+log(0.768)+log(0.197)+log(0.7472)+log(0.372)+log(0.4781)+log(0.04157)+log(0.4621)+log(0.6556)+log(0.9284)+log(0.3132)+log(0.2834)+log(0.3957)+log(0.7677)+log(0.4636)+log(0.1166)+log(0.159)+log(0.4545)+log(0.3448)+log(0.8076)+log(0.2935)+log(0.665)+log(0.2502)+log(0.9427)+log(0.3342)+log(0.365)+log(0.1427)+log(0.06621)+log(0.5555)+log(0.03393)+log(0.97)+log(0.1328)+log(0.5163)+log(0.9455)) # uses p-values from partial tests

\# (or likelihood ratio test) of conditional independencies

C

pchisq(C,df=2*80,lower.tail=FALSE) # p-value for C-test
